# Azo-Povarov Cycloaddition of *N*‑Carbonyl Aryldiazenes with *cis*,*trans*-Cycloocta-1,5-diene as a Fluorogenic Click Reaction for the Synthesis of Cinnoline Derivatives

**DOI:** 10.1021/acs.joc.6c00313

**Published:** 2026-04-30

**Authors:** Xabier Jiménez-Aberásturi, Javier Vicario, Frank Abendroth, Olalla Vázquez, Jesús M. de los Santos

**Affiliations:** † Department of Organic Chemistry I, Faculty of Pharmacy and Lascaray Research Center, 16402 University of the Basque Country (UPV/EHU), Paseo de la Universidad 7, 01006 Vitoria-Gasteiz, Spain; ‡ Faculty of Chemistry, and Chemical Biology Division, Marburg University, Hans-Meerwein-Straße 4, 35043 Marburg, Germany; § Centre for Synthetic Microbiology (SYNMIKRO), Karl-von-Frisch-Straße 14, 35043 Marburg, Germany

## Abstract

Herein, we describe a novel azo-Povarov click reaction between *N*-carbonyl aryldiazenes and *cis,trans*-cycloocta-1,5-diene. This uncatalyzed [4 + 2] cycloaddition reaction enables the synthesis of a broad series of cinnoline derivatives at room temperature, in short reaction times and excellent yields (up to 98%). The functionality of the reaction has been also evidenced through the report of a high-yielding gram-scale synthesis as well as diverse transformations of the final cinnoline substrates. Furthermore, an activatable fluorogenic version of the reaction has been developed using quenched azo-fluorophores as starting materials.

## Graphical abstract

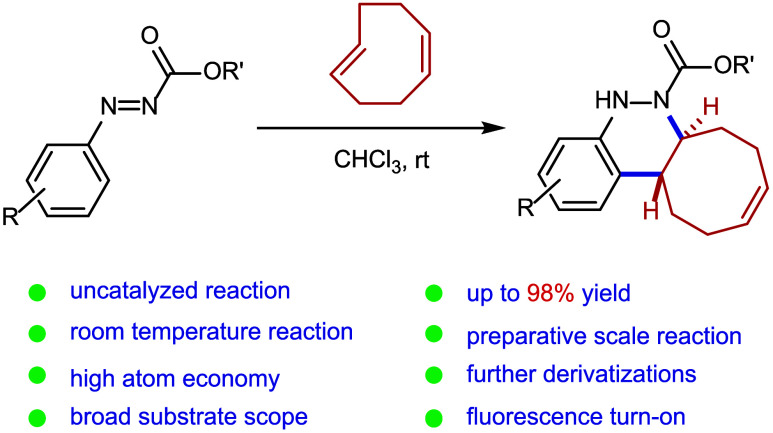

## Introduction

Since its conceptualization by Carolyn Bertozzi in 2003, bioorthogonal chemistry has emerged as one of the most fruitful areas in chemical biology. It encompasses a set of chemical reactions that can occur in biological environments without interfering with the native biomolecular processes.^1^ A reaction must fulfill several essential criteria in order to be considered bioorthogonal: it should proceed efficiently under physiological conditions, exhibit high selectivity and yield, preferably leading to a single product, and possess sufficiently fast kinetics to be practical in biological systems while avoiding any toxic effect.^2^

Initially, bioorthogonal reactions emerged as a subset of click chemistry, designed to proceed orthogonally to endogenous biochemical processes.^3^ Although early definitions emphasized the absence of byproducts, more recent perspectives acknowledge that side products may be tolerated, provided they do not interfere with native biomolecules.^4^ Due to the stringent requirements that must be met, the set of bioorthogonal reactions remains limited, highlighting the ongoing need for the development of new transformations.

Earlier, in 2002, Meldal^5^ and Sharpless^6^ independently established the concept of click chemistry through azide–alkyne cycloadditions, which had been extensively studied since 1963 (Scheme 1A).^7^ A major challenge in this area is mitigating the toxicity of Cu­(I) and Cu­(II) catalysts, which are often required to improve reaction kinetics. Subsequently, strain-promoted azide–alkyne cycloadditions (SPAAC) turned up as a superior alternative, avoiding the need for metal catalysts. Copper-free azide–alkyne cycloadditions were first reported by Bertozzi et al., who exploited the ring strain of cyclooctyne derivatives used as dipolarophiles. This bioorthogonal reaction enabled efficient biomolecule and cell surface-labeling without detectable cytotoxic effects.^8^

**Scheme 1:**
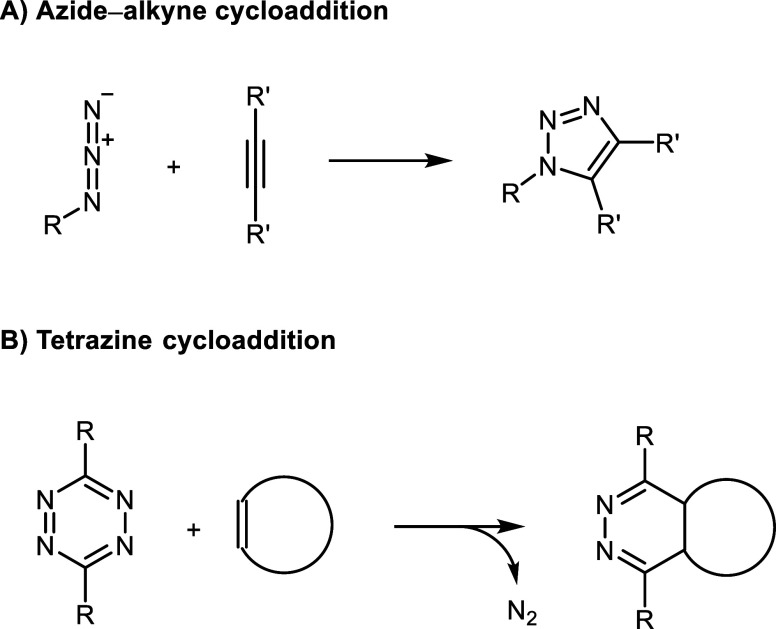
Azide–Alkyne and Tetrazine Click Reactions

Building upon the same strategy of employing highly strained dipolarophiles, inverse electron demand Diels–Alder (IEDDA) reactions have proven particularly valuable for bioorthogonal chemistry due to their exceptionally fast kinetics. In 2008, the use of tetrazines as dienes in [4 + 2] cycloadditions was independently reported by Fox^9^ and Hilderbrand,^10^ for applications in protein functionalization and *in vivo* cell labeling, respectively (Scheme 1B). In general, tetrazine derivatives with the highest reaction rates tend to be the least stable, particularly in the presence of thiols in biological environments. Consequently, the design of new tetrazines that combine high reactivity with improved stability remains an active and important area of research.^11^

At this stage, *trans*-cyclooctene (TCO) and its derivatives represent a highly advantageous class of dienophiles for IEDDA reactions, as their significant ring strain improves the kinetics of the aforementioned cycloadditions. Molecular conformation plays a critical role in their reactivity. While TCO can adopt a relatively stable crown conformation, other derivatives, such as *cis,trans*-cycloocta-1,5-diene or ethyl (*E*)-bicyclo­[6.1.0]­non-4-ene-9-carboxylate, which feature a *cis* bond at the fifth position, are forced to adopt a half-chair conformation. This conformation increases their strain energy, substantially enhancing their reactivity, and thus making them suited for bioorthogonal purposes (Figure 1).^12^

**Figure 1:**
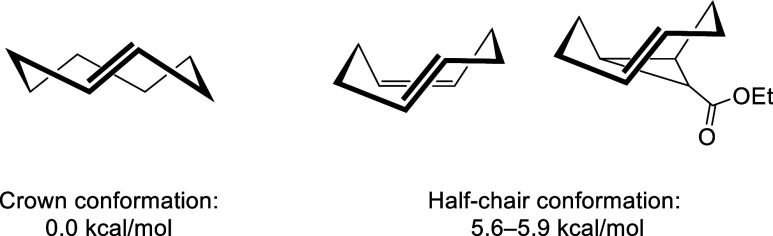
Strain energy differences between crown and half-chair conformations in *trans*-cyclooctene derivatives.

Live-cell and biomolecule imaging represent some of the most successful applications within the broad spectrum of both bioorthogonal and click chemistry.^2^ The use of fluorescent tags to label and track biomolecules remains one of the most effective strategies for studying intracellular processes. However, the challenges associated with removing the excess of fluorescent reagents can prolong the workflow and increase background signals, thereby limiting their utility. In this context, nonfluorescent tags that become fluorescent upon undergoing a click reaction represent a powerful alternative for efficient imaging. In 2003, Bertozzi’s group was once again at the forefront, publishing the use of the recently developed Staudinger ligation to activate a quenched coumarin derivative for specific protein labeling.^13^ Since then, the development of new click reactions with higher turn-on ratios and faster kinetics has remained a central focus of ongoing studies. In addition to the aforementioned Staudinger ligation, copper-catalyzed and strain-promoted azide–alkyne cycloadditions, as well as tetrazine-based cycloadditions, have been used to activate different fluorogenic dyes, such as fluorescein, coumarin, and BODIPY derivatives, for biological applications.^14^ Remarkably, Weissleder et al. reported an 11,000-fold fluorescence enhancement using a quenched coumarin–tetrazine compound activated by TCO^15^ and Bonns et al.^16^ achieved a 13,000-fold increase through a strain-promoted [3 + 2] azide-diazo cycloaddition.

Azo-bonds are well-known fluorescence quenchers that operate through either Förster resonance energy transfer (FRET) mechanisms or non-FRET processes, usually involving photochemical isomerization or rotational relaxation around the nitrogen–nitrogen double bond.^17^ Azo dyes have been widely used in biochemical applications, particularly as photoswitches for *in vivo* photopharmacology^18^ or as fluorescence quenchers in the imaging of hypoxic cancer cells.^19^ Furthermore, azo compounds are highly valuable functional groups in chemical synthesis, particularly for the construction of nitrogen-containing heterocycles *via* cycloaddition reactions, including [2 + 2], [3 + 2] and [4 + 2] annulations.^20^ As a part of our ongoing research in the use of azo compounds,^21^ we have recently reported the first example of an azo-Povarov reaction for the synthesis of cinnoline derivatives (Scheme 2A).^22^ In addition, these cinnoline substrates have demonstrated significant value as bioactive molecules and several derivatives have been reported to exhibit antimicrobial, anti-inflamatory, analgesic, and even antitumor properties.^23^ Therefore, the development of a straightforward click reaction for the synthesis of cinnoline derivatives would represent a noteworthy advancement in this field.

**Scheme 2:**
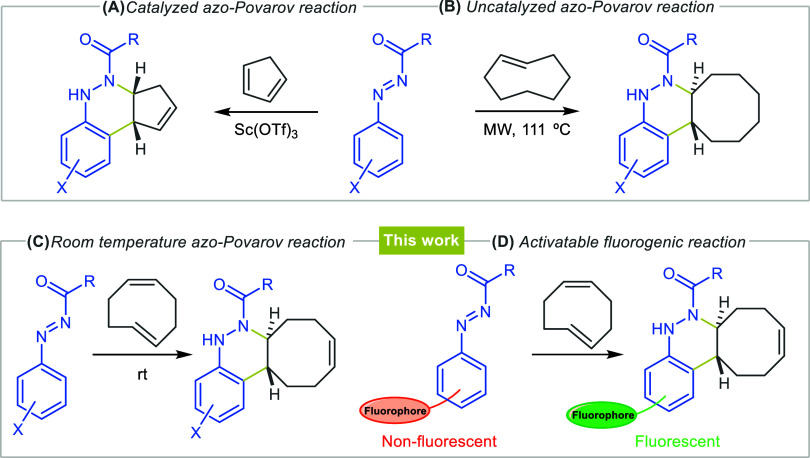
Design and Conceptualization of This Study

Taking into account the considerations mentioned above and, as a continuation of our studies on *N*-carbonyl aryldiazenes, we hypothesized that the use of more strained *cis,trans*-cycloocta-1,5-diene, instead of *trans*-cyclooctene (Scheme 2B),^24^ in the azo-Povarov reaction would lower the reaction temperature to *in vivo* conditions, potentially enabling a biocompatible click reaction (Scheme 2C). Moreover, the use of deactivated fluorophores as starting azo-dienes could lead to a novel activatable fluorogenic reaction (Scheme 2D).

## Results and Discussion

Starting aryldiazenes **2** were synthesized *via* protection of the corresponding arylhydrazines **1**, followed by oxidation of the N–N bond using *N*-bromosuccinimide (NBS). Once the azo compounds were obtained, we began the optimization of the reaction conditions, initially employing the model conditions described in our previous work.^24^ These conditions involve heating ethyl phenyldiazene carboxylate **2a** and *cis,trans*-cycloocta-1,5-diene under microwave irradiation at 111 °C (Table 1, entry 1). As expected, the starting material was consumed significantly faster than in the case of *trans*-cyclooctene (4 h), with the reaction reaching completion in only 30 min, affording the target product in 95% yield. Encouraged by these promising results, we next evaluated the reaction in chloroform at reflux. Under these conditions, the reaction was completed within 2 h (Table 1, entry 2), providing the final cinnoline in an excellent yield (98%). In view of the short reaction times observed, we decided to lower the reaction temperature to 40 °C (entry 3), and subsequently to room temperature (entry 4). Under these conditions, the corresponding cinnoline derivative was obtained in 5 and 7 h, respectively, with both reactions affording the product in 98% yield. At this stage, we aimed to evaluate the efficiency of the reaction under physiological conditions. To this end, we used phosphate-buffered saline (PBS, pH = 7.2) as the solvent instead of CHCl_3_, and the temperature was set to 37 °C (Table 1, entry 5). Under these conditions, the reaction time was increased to 12 h, likely due to the poor solubility of both the starting compound **2a** and the final adduct **3a**. Nevertheless, compound **3a** was obtained in 67% yield, remarkably demonstrating the feasibility of carrying out the reaction in biological media.

**Table 1:**
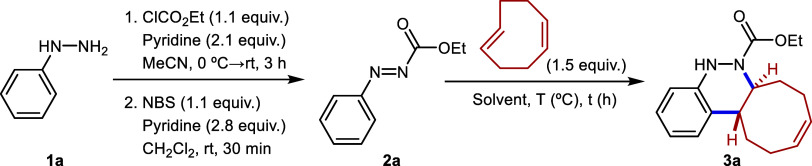
Optimization of the Reaction Conditions for the Azo-Povarov Reaction

entry^a^	solvent	temperature (°C)	time (h)	yield (%)^b^
1	CHCl_3_	111, MW	0.5	95
2	CHCl_3_	61	2	98
3	CHCl_3_	40	5	98
**4**	**CHCl**_**3**_	**rt**	**7**	**98**
5^c^	PBS	37	12	67^d^

a Unless otherwise noted, reactions were performed on a 0.5 mmol scale in a 0.17 M solution of **2a**, with 1.5 equiv of the dienophile. All reactions were carried out under a dry nitrogen atmosphere.

b Isolated yields.

c The reaction was conducted without inert atmosphere.

d Some unreacted aryldiazene **2a** was recovered from the crude reaction.

Next, we focused on the evaluation of the scope of the reaction with various aryldiazenes **2**. For this purpose, the conditions described in entry 4 were selected as optimal for the cycloaddition reaction, due to their mildness, simplicity, and excellent efficiency. Initially, *N*-carbonyl aryldiazenes bearing different substituents on the aromatic ring were used as substrates, as illustrated in Table 2. The presence of electron-donating groups in the *para* position of the aryldiazene, such as Me, OMe, or OCF_3_ (**2b**–**2d**), led to similar reaction rates, as the time required for full conversion remained at 7 h, providing satisfactory yields of 82%, 90%, and 39%, respectively. On the other hand, electron-withdrawing groups such as CF_3_ (**2e**) and halogen substituents such as Br and F (**2f**, **2g**) slightly accelerated the reaction, affording the corresponding products (**3e**–**3g**) in yields ranging from 25% to 94% after 3–5 h. A prolonged reaction time of 6 h was also observed for the *ortho-*substituted diene **2h**, likely due to the restricted approach of the diene from one side of the aromatic ring. Despite this, the corresponding cinnoline was isolated in a good 74% yield.

**Table 2:**
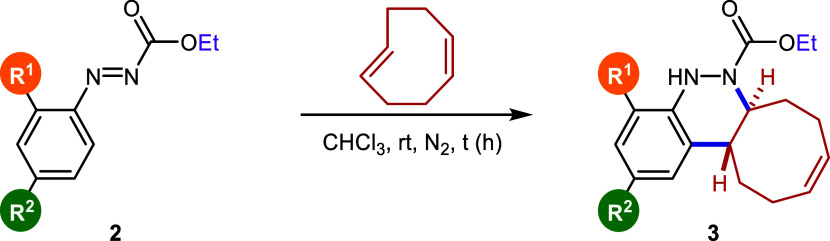

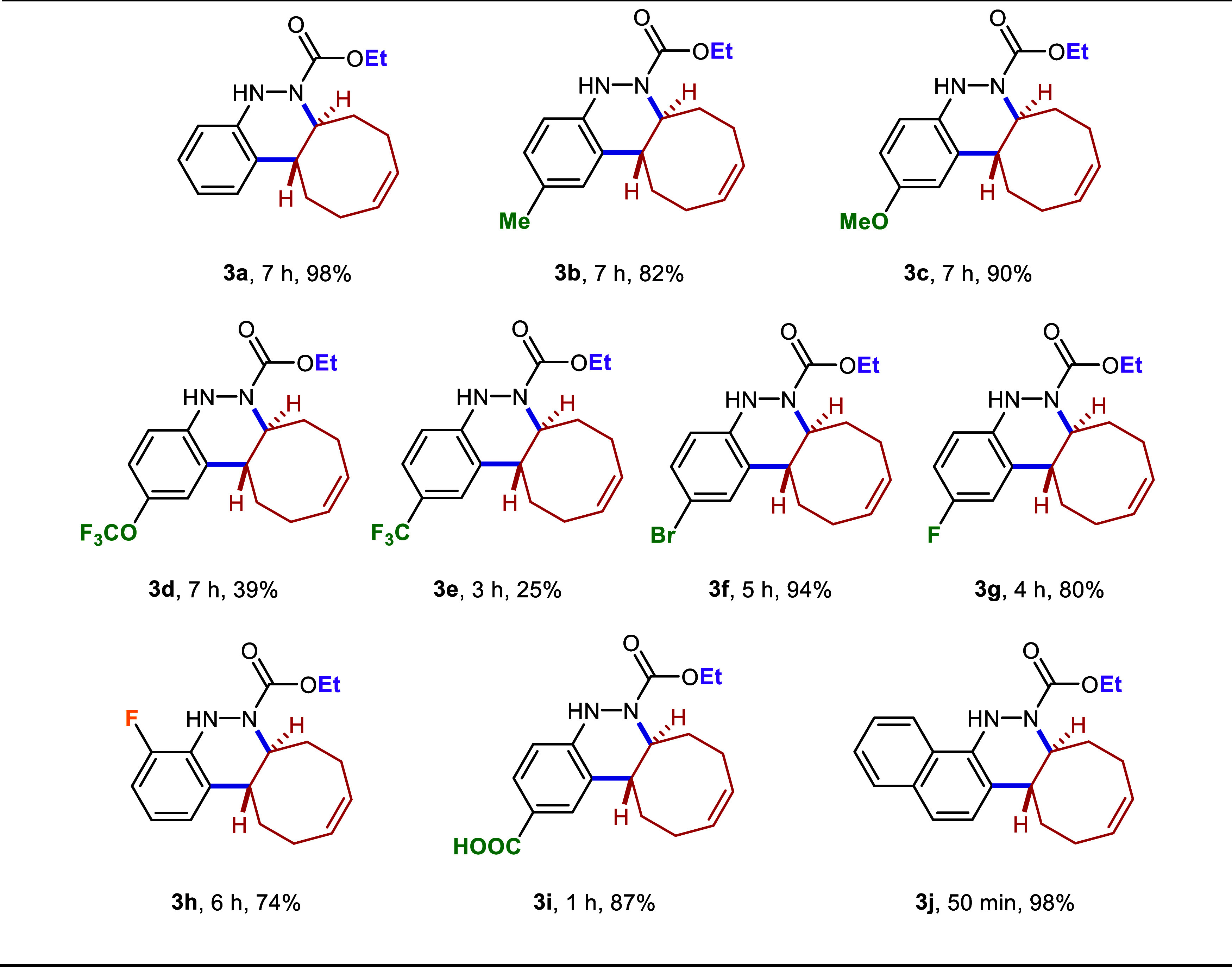
Scope of the Azo-Povarov Reaction with Various Functional Groups in the Aromatic Ring of Aryldiazene Carboxylates **2** ^a^ ^,^ ^b^

a Unless otherwise noted, reactions were performed on a 0.5 mmol scale in a 0.17 M solution of **2**, with 1.5 equiv of the dienophile. All reactions were carried out under a dry nitrogen atmosphere.

b Isolated yield.

Finally, a notable enhancement in the reaction rate was observed when utilizing electron-withdrawing functional groups with a negative mesomeric effect, such as the carboxylic acid **2i** or the naphthyl derivative **2j**. In both cases, the reaction was complete in less than 1 h, affording the corresponding cinnolines **3i** and **3j** in excellent yields (87% and 98%, respectively). These results are consistent with the inverse electron demand nature of the [4 + 2] cycloaddition, in which electron-deficient dienes react faster than the electron-rich ones.

Continuing with the investigation of the scope of the azo-Povarov click reaction, the protecting group on the nitrogen atom of aryldiazenes **2** was modified (Table 3). In this context, phenylhydrazine was protected with different chloroformates, and a subsequent oxidation reaction afforded the corresponding aryldiazenes **2k**–**2o**. *N*-Boc-aryldiazene **2k** required the longest reaction time with *cis,trans*-cycloocta-1,5-diene, taking 2 days to afford derivative **3k** in a good yield of 78%. In contrast, the *N*-Troc-protected azo compound **2l** and the *N*-CO_2_Ph-protected compound **2m** reacted in only 1.5 h and 2h, respectively, furnishing the corresponding cinnolines **3l** and **3m** in excellent yields (82% and 69%, respectively). Finally, aryldiazenes bearing *N*-Cbz and *N*-Alloc protecting groups yielded the final cinnoline products **3n** and **3o** in 71% and 50% yield after 8 and 5 h of reaction time, respectively.

**Table 3:**
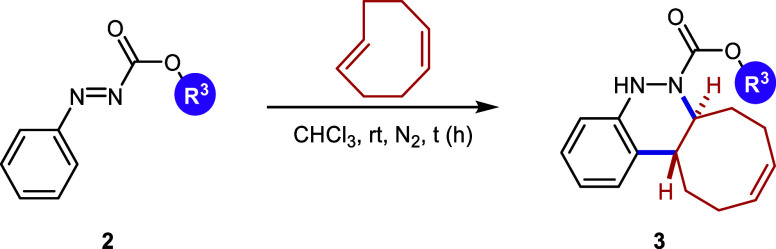

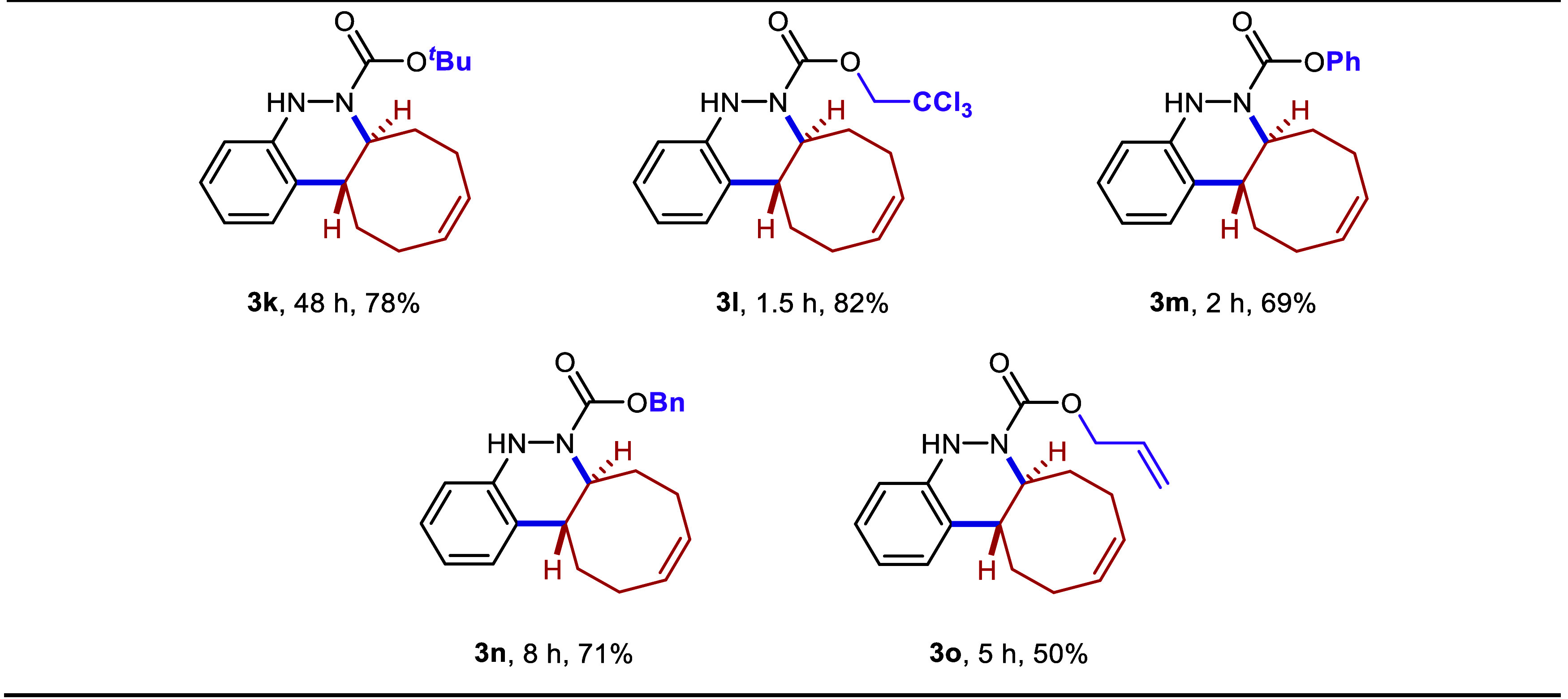
Azo-Povarov Reaction with Different Groups (R^3^) at the Nitrogen Atom of *N*-Aryldiazene Carboxylates **2** ^a^ ^,^ ^b^

a Unless otherwise noted, reactions were performed on a 0.5 mmol scale in a 0.17 M solution of **2**, with 1.5 equiv of the dienophile. All reactions were carried out under a dry nitrogen atmosphere.

b Isolated yield.

Moreover, acetyl chloride was used in order to functionalize phenylhydrazine, and the resulting compound was then oxidized to furnish *N*-acetyl aryldiazene **2p**,^25^ with the aim of studying its reactivity in the present click reaction. The starting material was fully consumed after only 1 h at room temperature. However, to our surprise, it afforded cinnoline derivative **4** in a poor yield of only 8%. We deduce that compound **4** arises from the oxidation of an unstable intermediate **3p**. The instability of both compounds **2p** and **3p** would account for the low yield observed in the cycloaddition reaction (Scheme 3).

**Scheme 3:**
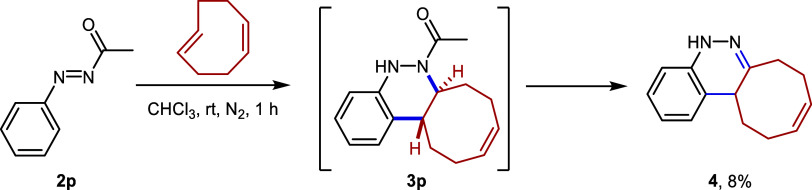
Azo-Povarov Reaction Using *N*-Acetyl Aryldiazene **2p**

In order to extend the scope of the novel click reaction to a different dienophile, we selected a cyclooctene derivative with a ring strain energy comparable to that of *cis,trans*-cycloocta-1,5-diene. In this context, the presence of a *cis* configuration in position 5 of the ring was identified as a key structural feature. Accordingly, we synthesized ethyl (1R*,8S*,9r*,*E*)-bicyclo­[6.1.0]­non-4-ene-9-carboxylate (see SI) and studied its reactivity toward aryldiazene **2a** (Table 4).

**Table 4:**
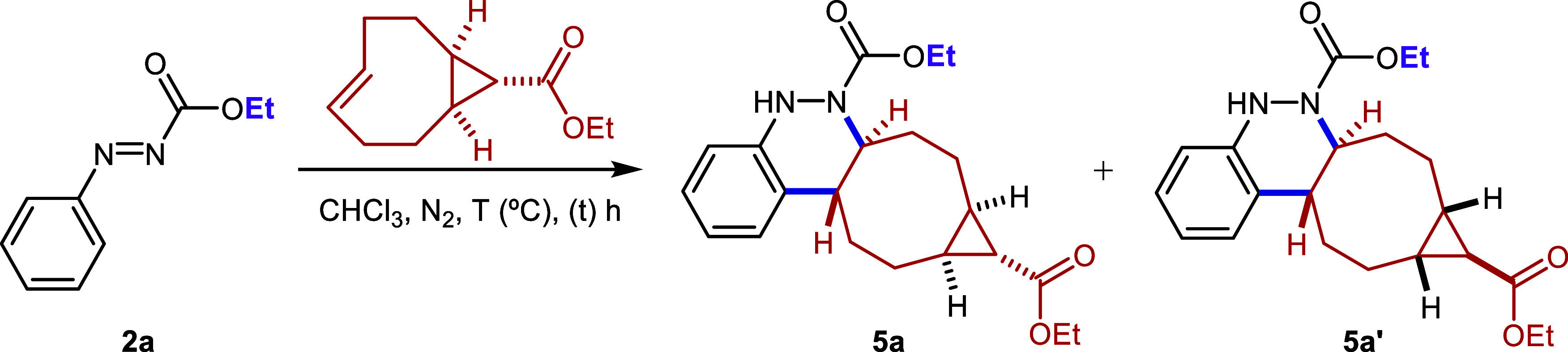
Azo-Povarov Reaction of *N*-Carbonyl Aryldiazene **2a** and Ethyl (1R*,8S*,9r*,*E*)-Bicyclo­[6.1.0]­non-4-ene-9-carboxylate

entry^a^	temperature (°C)	time (h)	yield (%)^b^
1	111, MW	0.5	93
2	61	3	99
3	rt	8	99

a Unless otherwise noted, reactions were performed on a 0.5 mmol scale in a 0.17 M solution of **2a**, with 1.5 equiv of the dienophile. All reactions were carried out under a dry nitrogen atmosphere.

b Isolated yields.

The reaction proceeded similarly to that of *cis,trans*-cycloocta-1,5-diene under all tested conditions. Notably, product **5a** was obtained in excellent yield (93%) after only 0.5 h upon microwave irradiation at 111 °C (Table 4, entry 1). Quantitative yields were also achieved under refluxing chloroform and at room temperature after 3 and 8 h (entries 2 and 3, respectively). In all cases, compound **5a** was obtained as an inseparable mixture of diastereomers (**5a** and **5a**’) in a 65:35 ratio, although the major and minor components could not be definitively assigned.

Taken together with earlier reports in the literature, this example may help clarify the mechanistic picture of the azo-Povarov reaction. While prior studies often point toward a stepwise pathway, our findings suggest a different scenario. In particular, the involvement of a nonactivated 2π component, combined with the complete stereoselectivity observed in bond formation, is more consistent with a concerted [4 + 2] process. Overall, the operative mechanism appears to be strongly influenced by the electronic properties of the reacting partners. Thus, both stepwise and concerted pathways may be viable when nucleophilic 2π systems are involved, whereas nonactivated alkenes seem to favor an exclusively concerted route.

The robustness of the described click reaction was proved by scaling up the model reaction to 5 mmol of the starting azo compound **2a** (0.89 g). This gram-scale experiment afforded 1.43 g of cinnoline derivative **3a** in >99% yield, validating the high efficiency of the cycloaddition with full atom economy, regardless of the reaction scale (Scheme 4a). The synthetic utility of the novel procedure was further demonstrated through several transformations carried out on final products **3**, highlighting their potential as versatile building blocks. First, the *N*-Boc-protected cinnoline **3k** was deprotected using a 2:3 mixture of TFA and CH_2_Cl_2_ at room temperature, affording bis ammonium salt **6** in quantitative yield. An attempt to isolate the free diamine by treatment with NaHCO_3_ led to the formation of unstable cinnoline **7**, which was completely oxidized to compound **4** after 3 days (Scheme 4b). On the other hand, model product **3a** was subjected to Ni-Raney-catalyzed hydrogenation, which not only reduced the double bond of the cyclooctene moiety but also cleaved the N–N bond, affording free amine **8** in quantitative yield after 24 h of reaction. The resulting amine was subsequently protected with ethyl chloroformate to afford compound **9** in 86% yield after 1.5 h (Scheme 4c).

**Scheme 4:**
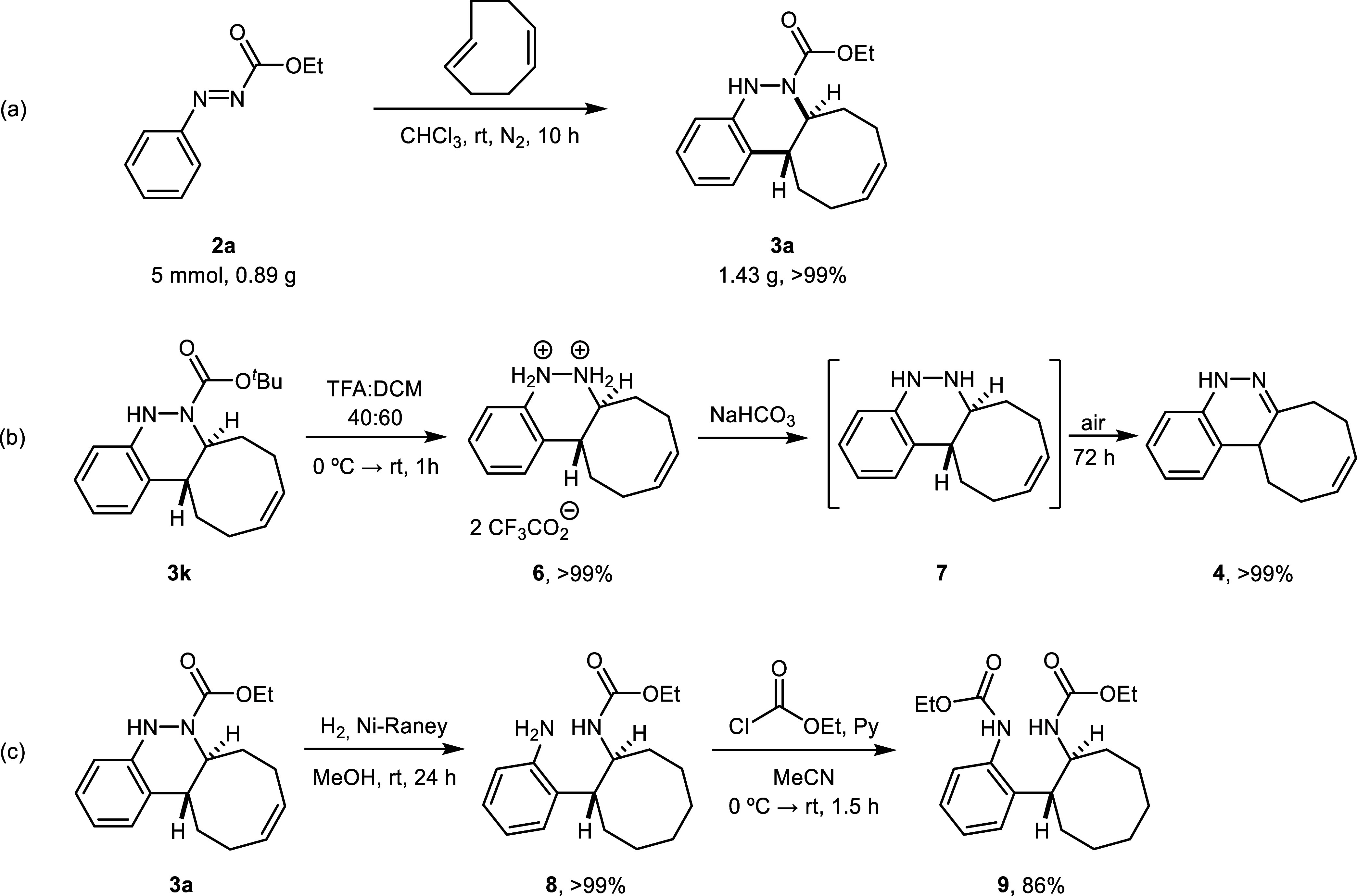
Gram-Scale Synthesis and Derivatization of Compounds **3**

Finally, we focused our efforts on demonstrating the utility of the novel click reaction described above as a potential bioorthogonal transformation. To this end, we aimed to evaluate the efficiency of the fluorescence turn-on effect that our cycloaddition could exhibit. For this purpose, we based our research on two different caged-fluorophores, which we hypothesized to be quenched due to the presence of the azo bond in the starting materials. These fluorophores were expected to be reactivated upon undergoing the rapid [4 + 2] azo-Povarov cycloaddition with *cis, trans*-cycloocta-1,5-diene.

Fluorescein derivative **10** was used to prepare caged azo-fluorescein conjugate **11** by coupling diazenyl benzoic acid **2i** with the ethylenediamine linker of **10** *via* amide bond formation (see Experimental Section). The reactivity of the resulting diene **11** with *cis*,*trans*-cycloocta-1,5-diene was then evaluated in a 1:1 MeCN:H_2_O mixture at 37 °C. Full conversion was achieved after 30 min, affording the final derivative **12** in 85% yield (Scheme 5).

**Scheme 5:**
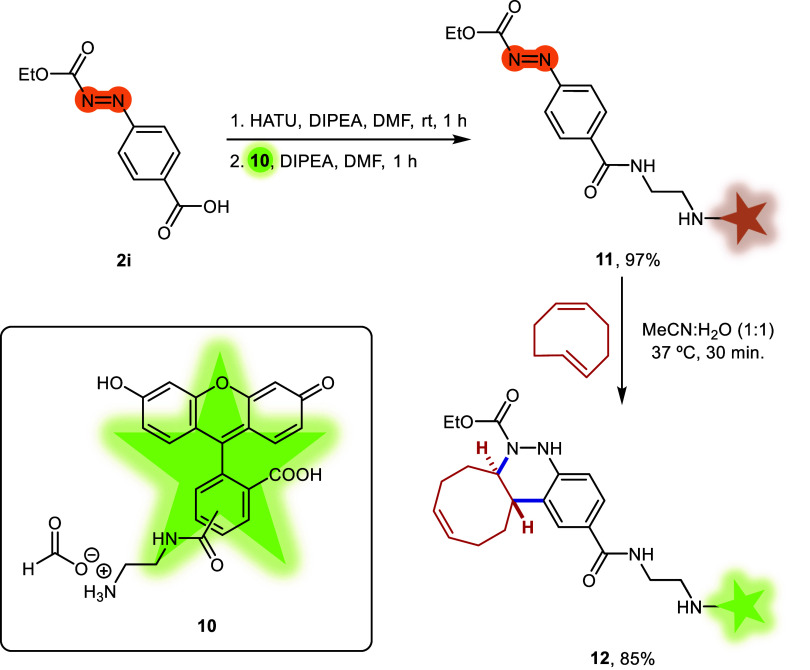
Fluorescence Turn-On *via* Azo-Povarov Reaction of Azo-Fluorescein **11** with *cis,trans*-Cycloocta-1,5-diene

Next, we evaluated the fluorescence properties of compound **12** at a 5 μM concentration in PBS buffer solution (phosphate buffer 50 mM, NaCl 150 mM, pH = 7.4) containing 0.5% DMSO. The cinnoline-fluorescein derivative **12** showed a maximum excitation wavelength at 498 nm and a maximum emission at 523 nm (Figure 2A). We then compared the fluorescence intensity of compound **12** with that of azo-fluorescein **11** at identical wavelengths, and observed a 3-fold increase (Figure 2B). This result suggests that the azo bond slightly quenches the fluorescence, likely due to the relatively long distance between the quencher and the fluorophore.

**Figure 2:**
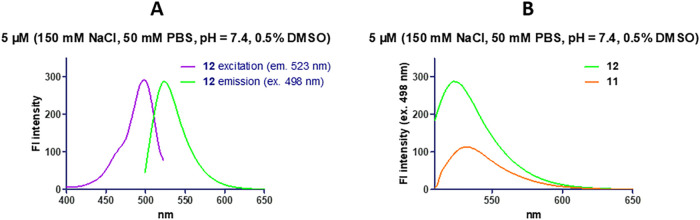
Photocharacterization of derivative **12** and comparison of its fluorescence emission with that of azo compound **11**.

In an effort to obtain a more efficiently caged fluorophore, we designed and synthesized the azo-coumarin derivative **14**, starting from 7-hydroxycoumarin derivative **13**, with the N=N quencher directly attached to the fluorophore core (see Experimental Section). Once the starting material was prepared, we studied its azo-Povarov cycloaddition in chloroform at room temperature (Table 5, entry 1). The reaction reached completion after 20 min, affording the desired product **15** with a modest 62% conversion. Switching to a MeCN:H_2_O solvent mixture at 37 °C accelerates the consumption of the starting material, being completely consumed within only 10 min; gratifyingly, the conversion remains comparable (57%, entry 2). In both crude reactions, the ^1^H NMR spectra revealed unstable impurities, likely due to a mixture of diastereomers **16**. These byproducts presumably result from a light-mediated [2 + 2] cycloaddition between coumarin **14** and *cis,trans*-cycloocta-1,5-diene. Considering these findings, we carried out the azo-Povarov cycloaddition in MeCN:H_2_O at 37 °C under complete darkness. To our delight, the reaction was completed after only 5 min, which we attribute to the fact that only a single addition of the cyclooctene occurs under these conditions, leading to faster consumption of the starting material and affording cinnoline **15** with an excellent 85% conversion (Table 5, entry 3).

**Table 5:**
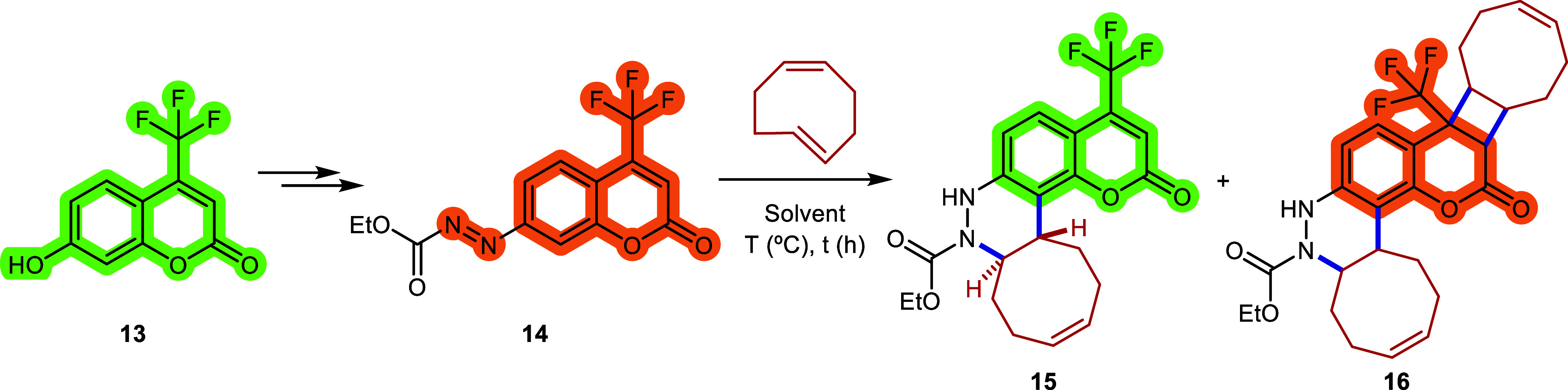
Azo-Povarov Reaction of Azo-Coumarin **14** with *cis,trans*-Cycloocta-1,5-diene

entry^a^	solvent	[**14**] (M)	T^a^ (°C)	time (min)^b^	light	conversion to compound **15** (%)^c^
1	CHCl_3_	0.1	r.t.	20	Under visible light exposure	62
2	MeCN/H_2_O (1:1)	0.03	37	10	Under visible light exposure	57
3	MeCN/H_2_O (1:1)	0.03	37	5	In the darkness	85

a Reactions were performed on a 0.05 mmol scale with 2 equiv of the dienophile.

b Reaction times reported correspond to the time required for complete consumption of the starting material **14**.

c Conversion was calculated based on the ratio of compounds **15** and **16** in the crude reaction mixture as determined by ^1^H NMR.

Encouraged by this excellent result, we studied the fluorescence properties of cinnoline **15** and compared them with those of the caged azo-coumarin **14** in different solvents, using 10 μM concentrations. The maximum excitation wavelength of compound **15** ranged from 360 to 375 nm, depending on the solvent, while the maximum emission varied between 521 and 560 nm (Figure 3A). As reported in previous studies,^26^ the fluorescence of coumarin derivatives is strongly affected by the solvent polarity, exhibiting intense signals in nonpolar solvents such as CHCl_3_ or dioxane, and lower intensities in more polar solvents such as DMF or MeCN.

**Figure 3:**
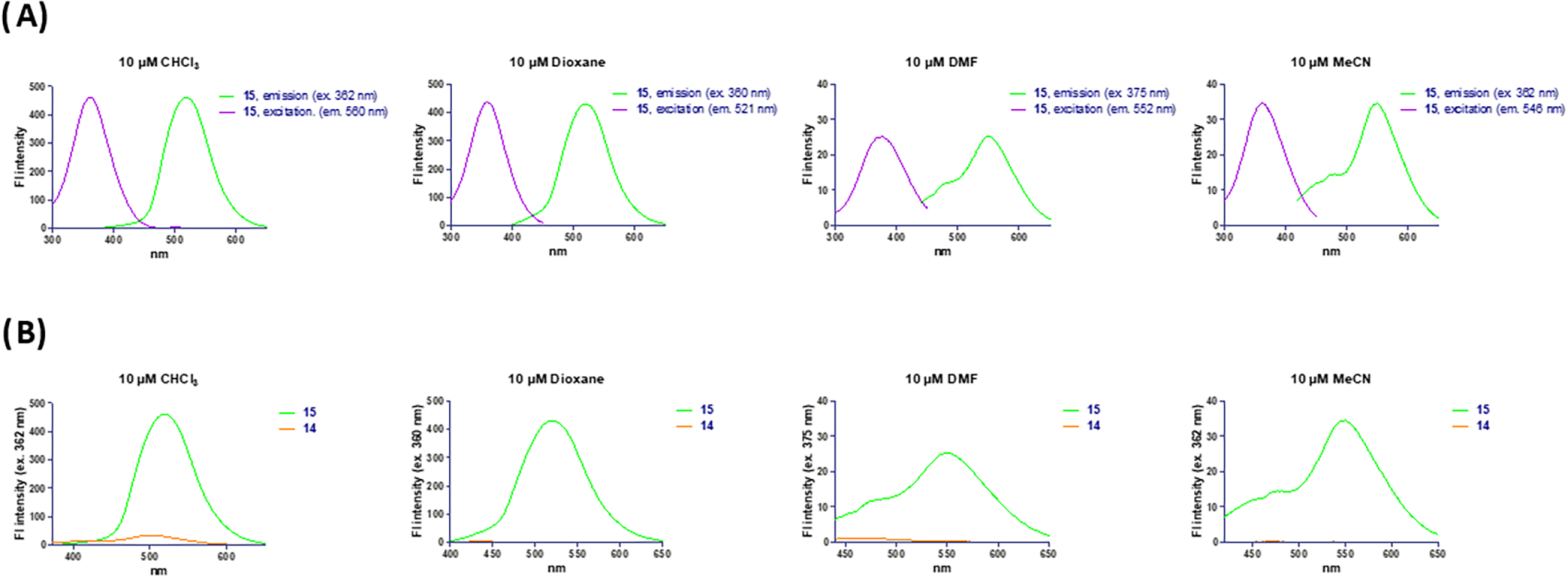
Photocharacterization of coumarin derivative **15** and comparison of the fluorescence emission with that of azo compound **14**.

Using the optimal excitation wavelengths, the emission intensity of the coumarin **15** was compared with that of the caged precursor **14** (Figure 3B), and the fluorescent turn-on ratio of the azo-Povarov reaction was calculated for each solvent. In chloroform and DMF, the emission was increased by 21- and 52-fold, respectively, while in acetonitrile, the emission was 165 times higher. Remarkably, in dioxane, an outstanding 881-fold increase in emission intensity was observed, highlighting the high efficiency of fluorescence quenching by the azo group. Moreover, the absorption spectra of compound **15** in the aforementioned solvents showed only a slight positive solvatochromic effect in DMF (see Figure S1 in SI).

## Conclusions

In summary, we have developed an innovative, straightforward, and uncatalyzed click reaction that enables the quantitative synthesis of a broad range of cinnoline derivatives at room temperature and in short reaction times, by exploring the high reactivity of *cis,trans*-cycloocta-1,5-diene in the azo-Povarov reaction using *N*-carbonyl aryldiazenes. This protocol efficiently accommodates *N*-aryldiazenes bearing either electron-donating groups (*e.g.*, Me, OMe, OCF_3_), electron-withdrawing groups (*e.g.*, CF_3_), or halogen substituents (*e.g.*, Br, F) at the *para* position of the aromatic ring, affording cycloocta­[*c*]­cinnolines in moderate to excellent yields. Notably, the electronic nature and positional variation (2- or 4-substitution) of the substituents on the aryl ring exhibit minimal impact on reaction efficiency, underscoring the generality of this synthetic method for accessing the cinnoline scaffold. The gram-scale synthetic version and subsequent postfunctionalization of the final products **3** highlight the practical applicability and scalability of the developed methodology. Finally, the efficient fluorescence quenching by the azo group was confirmed using a coumarin-based probe, which exhibited an 881-fold increase in fluorescence emission upon activation with the aforementioned dienophile.

## Experimental Section

### General Information

Solvents for extraction and chromatography were of reagent grade. All solvents used in reactions were freshly distilled and dried over 3 Å molecular sieves before use. Unless otherwise mentioned, all other solvents and reagents were purchased from commercial suppliers and were either recrystallized or distilled as necessary, or used without further purification. All reactions were carried out under a dry nitrogen atmosphere. Photoisomerization reactions were performed using a multilamp photoreactor (Model MLU18) equipped with Model 3020 lamps (254 nm) (Photochemical Reactors Limited, Reading RG4 PA, UK). Reaction progress was monitored by analytical thin-layer chromatography (TLC) performed on precoated Merck silica gel 60 F_254_ TLC aluminum plates, with spot visualized using VL-6C UV lamps (λ = 254 nm), ninhydrin stain, or potassium permanganate stain. Melting points are uncorrected. ^1^H (400 or 500 MHz), ^13^C (100 or 125 MHz), ^19^F (282, 376, or 565 MHz) and ^31^P (162 MHz) spectra were recorded on a Bruker Avance 400 (400 MHz), Bruker AV III HD 500 MHz (500 MHz) or Bruker AV III NEO 600 (600 MHz) spectrometers, as appropriate, using CDCl_3_, (CD_3_)_2_CO, (CD_3_)_2_SO or CD_3_OD as solvents, as specified below. Chemical shifts (δ) are reported in parts per million (ppm) with the internal signals at 7.24 ppm (CDCl_3_), 2.05 ppm (acetone-d_6_), 2.50 (DMSO-*d*_6_), and 3.37 ppm (CD_3_OD) as standards for ^1^H NMR. Chemical shifts (δ_C_, δ_F_, and δ_P_) are reported in parts per million (ppm), with the internal signals at 77.16 ppm (CDCl_3_), 206.3 and 29.8 ppm (acetone-d_6_), 39.5 ppm (DMSO-*d*_6_), and 49.0 ppm (CD_3_OD) as standards for ^13^C NMR. The external fluorotrichloromethane (CCl_3_F) at 0.0 ppm was used as the standard for ^19^F NMR, and that of phosphoric acid (H_3_PO_4_) at 0.0 ppm for ^31^P NMR. All coupling constants (*J*) values are given in Hz. ^19^F, ^31^P, and ^13^C NMR spectra were recorded in a broadband decoupled mode from hydrogen nuclei. Distortionless Enhanced Polarization Transfer (DEPT) experiments were used to support peak assignments for ^13^C NMR. Structural assignments were made with additional information from gCOSY, gHSQC, and gHMBC experiments. Multiplicity abbreviations are reported as follows: q= singlet, d = doublet, t = triplet, q = quartet, m = multiplet, dd = double doublet, dt = double triplet, dq = double quartet, bs *=* broad singlet. Infrared (IR) spectra were recorded on a Nicolet iS10 FTIR spectrophotometer (Thermo Scientific) using the Smart iTR accessory. Absorbance frequencies are reported at the maximum intensity in cm^–1^. FTIR spectra were recorded using neat solids or oils. High-resolution mass spectra (HRMS) were acquired by positive-ion electrospray ionization (ESI) method using a time-of-flight Q-TOF system. Data are reported as *m*/*z* values. Fluorescence measurements were performed on a Jasco FP-6500 spectrofluorometer equipped with a Thermo Haake WKL 26 water recirculator at 20 °C, using 1400 μL fluorescence cuvettes (Hellma Analytics, 104F-QS). A 3 nm excitation bandwidth was used; emission bandwidth was set at 10 nm for compounds **11** and **12**, and 5 nm for compounds **14** and **15**. Chromatographic purification was carried out by flash chromatography using commercial grades of silica gel (particle size <230 mesh) under pressure. *N*-Boc-protected phenylhydrazine and *N*’-phenylacetohydrazide are commercially available. All other functionalized hydrazines and *N*-carbonyl aryldiazenes **2** were prepared according to literature procedures.^22,24^

### Synthetic Procedures and Characterization Data

#### Synthesis and Spectral Data of *N*-Carbonyl Aryldiazene **2i**

##### 4-(2-(Ethoxycarbonyl)­hydrazineyl)­benzoic acid (**1i**)

Following a modified literature procedure,^22,24^ a stirred solution of 4-hydrazinylbenzoic acid (7.62 g, 50 mmol) and pyridine (12.5 mL, 155 mmol) in CH_3_CN (100 mL) was cooled to 0 °C, and ethyl chloroformate (5.3 mL, 55 mmol) was added dropwise. The reaction mixture was stirred for 15 min at 0 °C and then for 5 h at room temperature. AcOEt (100 mL) was added, and the compound was extracted with aqueous NaOH (0.1M, 3 × 100 mL). The combined layers were acidified with aqueous HCl (2M, 15 mL), and the product was extracted with AcOEt (5 × 100 mL). The combined organic phases were washed with brine (2 × 50 mL), dried over anhydrous MgSO_4_, filtered, and concentrated under vacuum to yield 3.18 g (28%) of a yellow solid, which was used in the next step without further purification. Mp: 201–204 °C. FTIR (neat) *v*_max_ (cm^–1^) 3308, 2362, 1682. ^1^H NMR (400 MHz, (CD_3_)_2_CO): δ 10.69 (bs, 1H, COOH), 8.34 (bs, 1H, NH), 7.88 (d, ^3^ *J*_HH_ = 8.9 Hz, 2H, 2 × CH_ar_), 7.57 (bs, 1H, NH), 6.85 (d, ^3^ *J*_HH_ = 8.9 Hz, 2H, 2 × CH_ar_), 4.12 (q, ^3^ *J*_HH_ = 7.1 Hz, 2H, CH_2_O), 1.20 (t, ^3^ *J*_HH_ = 7.1 Hz, 3H, CH_3_). ^13^C­{^1^H} NMR (100 MHz, (CD_3_)_2_CO): δ 163.5 (COOH), 157.5 (C=O), 155.7 (C_quat_N), 133.1 (2 × CH_ar_), 119.6 (C_quat_), 112.1 (2 × CH_ar_), 61.8 (CH_2_O), 14.9 (CH_3_). HRMS (ESI) *m*/*z*: [M + H]^+^ Calcd for C_10_H_13_N_2_O_4_ 225.0875; Found 225.0869.
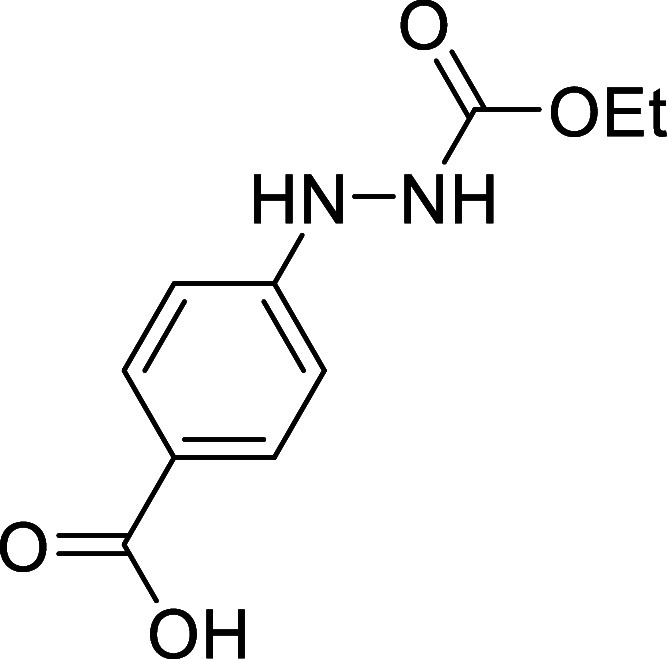


##### 4-((Ethoxycarbonyl)­diazenyl)­benzoic acid (**2i**)

Following a modified literature procedure, pyridine (3.1 mL, 38 mmol) was added to a stirred solution of the previously prepared 4-(2-(ethoxycarbonyl)­hydrazineyl)­benzoic acid (2.24 g, 10 mmol) in CH_2_Cl_2_ (70 mL). *N*-Bromosuccinimide (NBS; 2.00 g, 11 mmol) was then added portionwise. The reaction mixture was stirred for 30 min, and subsequently washed with aqueous HCl (1.5M, 100 mL), an aqueous solution of sodium thiosulfate (1.5%, 50 mL), and brine (100 mL). The organic phase was dried over anhydrous MgSO_4_, filtered, and concentrated to dryness in vacuum. The crude product was purified by flash-column chromatography (SiO_2_, hexanes/AcOEt 9:1) to afford 1.92 g (86%) of **2i** as a red solid. Mp: 183–184 °C. FTIR (neat) *v*_max_ (cm^–1^) 2996, 1752, 1684. ^1^H NMR (400 MHz, (CD_3_)_2_CO): δ 8.26 (d, ^3^ *J*_HH_ = 8.7 Hz, 2H, 2 × CH_ar_), 7.99 (d, ^3^ *J*_HH_ = 8.7 Hz, 2H, 2 × CH_ar_), 4.51 (q, ^3^ *J*_HH_ = 7.1 Hz, 2H, CH_2_O), 1.43 (t, ^3^ *J*_HH_ = 7.1 Hz, 3H, CH_3_). ^13^C­{1H} NMR (100 MHz, (CD_3_)_2_CO) δ 166.6 (COOH), 162.3 (C=O), 154.7 (C_quat_N), 135.6 (C_quat_), 131.8 (CH_ar_), 123.9 (CH_ar_), 65.3 (CH_2_O), 14.3 (CH_3_). Although a satisfactory HRMS could not be obtained, the presence of compound **2i** is evident from the formation of compound **3i** after the reaction with *cis*,*trans*-cycloocta-1,5-diene, which was fully characterized.
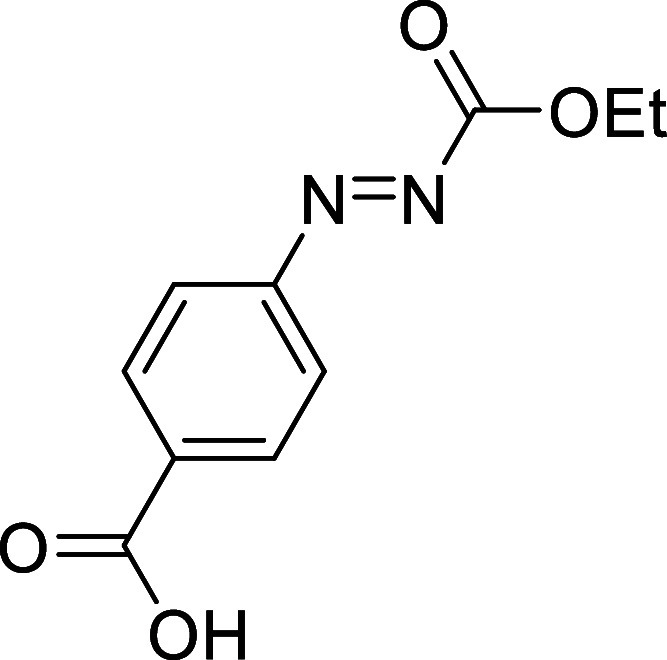


#### Synthesis of *cis*,*trans*-Cycloocta-1,5-diene **IV**

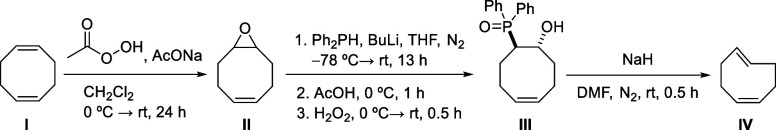



##### Synthesis of (*Z*)-9-Oxabicyclo­[6.1.0]­non-4-ene (**II**)

Following a modified literature procedure,^27^ peracetic acid (15%, 45 mL, 100 mmol) was added dropwise to a suspension of sodium acetate (8.3 g, 100 mmol) and *cis*,*cis*-cycloocta-1,5-diene (12.4 mL, 100 mmol) at 0 °C. The reaction mixture was then stirred at room temperature for 24 h. After this time, it was cooled to 0 °C and quenched with aqueous NaOH (40%) to reach pH ∼ 9. The resulting mixture was extracted with Et_2_O (4 × 80 mL), dried over anhydrous MgSO_4_, filtered, and the solvent was eliminated under reduced pressure at 0 °C to yield 8.06 g (65%) of **II** as a colorless oil, which was used in the next reaction step without further purification. Spectral data were consistent with those previously reported in the literature.^28^ 
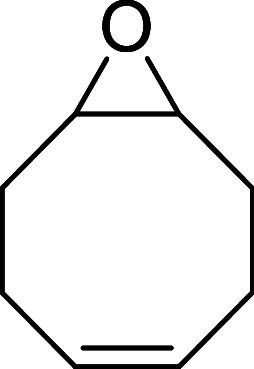


##### Synthesis of ((1R*,8R*,*Z*)-8-Hydroxycyclooct-4-en-1-yl)­diphenylphosphine oxide (**III**)

Following a modified literature procedure,^29^ ^*n*^BuLi (1.6 M, 46.9 mL, 75 mmol) was added dropwise to a solution of **II** (6.21 g, 50 mmol) and Ph_2_PH (11.17 g, 60 mmol) in degassed THF (200 mL) at −78 °C. The mixture was stirred at the same temperature for 1 h. It was then allowed to warm to room temperature and stirred for an additional 13 h. Next, AcOH (5.2 mL, 90 mmol) was added dropwise at 0 °C, and the mixture was stirred for 30 min. H_2_O_2_ (30%, 9.2 mL, 90 mmol) was then added dropwise at 0 °C, and the reaction mixture was stirred at room temperature for 3 h. The resulting mixture was extracted with CHCl_3_ (4 × 150 mL), and the combined organic layers were washed with brine (2 × 100 mL), dried over anhydrous MgSO_4_, filtered, and the solvent was eliminated under vacuum. The crude product was precipitated from toluene to afford 8.32 g (51%) of **III** as a white solid. Mp: 170–172 °C. FTIR (neat) *v*_max_ (cm^–1^) 3234, 3051, 2929, 1176. ^1^H NMR (400 MHz, (CDCl_3_)): δ 7.75–7.64 (m, 4H, 4 × CH_ar_), 7.50–7.40 (m, 6H, 6 × CH_ar_), 5.85 (m, 1H, =CH), 5.47 (m, 1H, =CH), 4.69 (d, ^3^ *J*_HH_ = 3.5 Hz, 1H, OH), 4.11 (m, 1H, CHOH), 3.03 (m, 1H, CHP), 2.42–2.31 (m, 2H, CH_2_), 2.25 (m, 1H, CH_2_), 2.11–1.96 (m, 2H, CH_2_), 1.72–1.59 (m, 2H, CH_2_), 1.41 (m, 1H, CH_2_). ^13^C­{1H} NMR (100 MHz, (CDCl_3_)): δ 134.2 (^1^ *J*_CP_ = 98.7 Hz, C_quat_P), 132.1 (=CH), 131.8 (d, ^1^ *J*_CP_ = 95.9 Hz, C_quat_P), 131.6 (d, ^3^ *J*_CP_ = 9.0 Hz, 2 × CH_ar_), 131.5 (d, ^4^ *J*_CP_ = 2.8 Hz, CH_ar_), 131.5 (d, ^4^ *J*_CP_ = 2.7 Hz, CH_ar_), 130.6 (^3^ *J*_CP_ = 9.1 Hz, 2 × CH_ar_), 128.6 (d, ^2^ *J*_CP_ = 11.6 Hz, 2 × CH_ar_), 128.4 (d, ^2^ *J*_CP_ = 11.4 Hz, 2 × CH_ar_), 127.0 (=CH), 73.0 (d, ^2^ *J*_CP_ = 5.4 Hz, CHOH), 41.4 (d, ^1^ *J*_CP_ = 71.0 Hz, CHP), 36.3 (d, ^3^ *J*_CP_ = 7.5 Hz, CH_2_), 25.3 (CH_2_), 24.2 (d, ^2^ *J*_CP_ = 16.7 Hz, CH_2_), 23.6 (CH_2_). ^31^P NMR (162 MHz, CDCl_3_): δ 41.8. HRMS (ESI) *m*/*z*: [M + H]^+^ Calcd for C_20_H_24_O_2_P 327.1514; Found 327.1495.
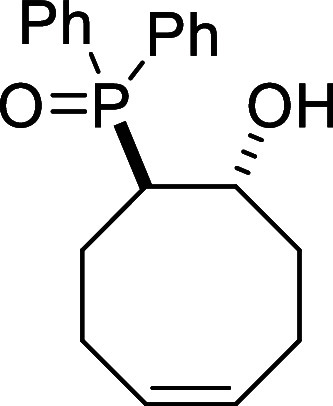


##### Synthesis of *cis*,*trans*-Cycloocta-1,5-diene (**IV**)

Following a modified literature procedure,^29^ NaH (60%, 1.00 g, 25 mmol) was washed with toluene (20 mL) and pentane (4 × 20 mL), and suspended in degassed DMF (20 mL). A solution of **III** (3.26 g, 10 mmol) in degassed DMF (20 mL) was added dropwise, and the resulting mixture was stirred vigorously at room temperature for 30 min. The reaction was quenched with ice, and the product was extracted with pentane (100 mL), washed with water (2 × 50 mL), dried over anhydrous MgSO_4_, filtered, and the solvent was eliminated under vacuum at 0 °C to afford 0.93 g (86%) of **IV** as a colorless liquid. Spectral data were consistent with those previously reported in the literature.^30^ 
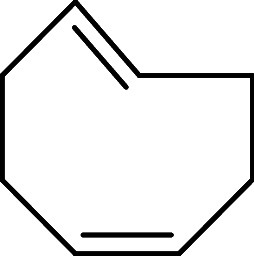


#### Synthesis of Ethyl (1R*,8S*,9r*,*E*)-bicyclo­[6.1.0]­non-4-ene-9-carboxylate **VI**

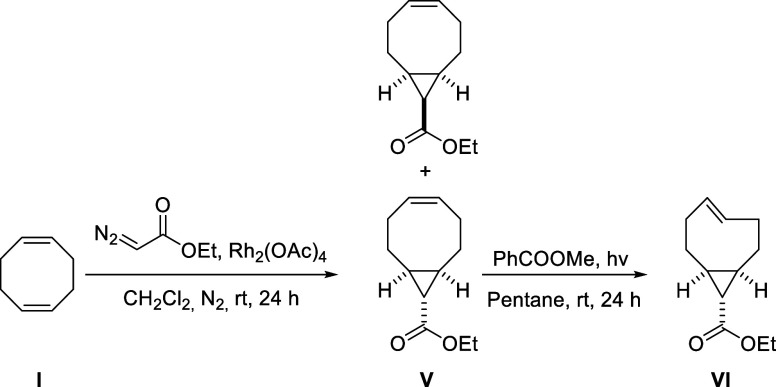



##### Synthesis of Ethyl (1R*,8S*,9r*,*Z*)-Bicyclo­[6.1.0]­non-4-ene-9-carboxylate (**V**)

Following a modified literature procedure,^31^ ethyl diazoacetate (85% in CH_2_Cl_2_, 6.2 mL, 50 mmol) was added *via* syringe pump over 5 h to a mixture of rhodium­(II) acetate (110 mg, 0.25 mmol) and *cis*,*cis*-cycloocta-1,5-diene (53 mL, 425 mmol) at room temperature. The reaction mixture was then stirred for an additional 16 h. The crude product was purified by flash-column chromatography (SiO_2_, 100% hexanes) to obtain 3.21 g (33%) of **V** and 4.98 g (51%) of the *syn* diastereomer as two separated colorless oils. Spectral data were consistent with those reported in the literature.^32^ 
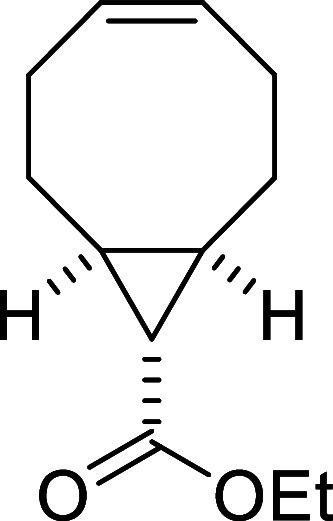


##### Synthesis of Ethyl (1R*,8S*,9r*,*E*)-Bicyclo­[6.1.0]­non-4-ene-9-carboxylate (**VI**)

A solution of **V** (3.98 g, 20.5 mmol) and methyl benzoate (5.1 mL, 40.96 mmol) in pentane (10 mL) was irradiated at 254 nm for 24 h. The crude product was extracted with an aqueous solution of AgNO_3_ (15%, 25 mL), and the aqueous phase was washed with pentane (20 mL). Aqueous NH_4_OH (30%, 20 mL) was then added to the aqueous phase, and the product was extracted with pentane (3 × 30 mL), dried over anhydrous MgSO_4_, filtered, and the solvent was eliminated under vacuum to obtain 214 mg (5%) of **VI** as a colorless oil. ^1^H NMR (400 MHz, CDCl_3_): δ 5.76 (m, =CH), 5.26 (m, =CH), 4.05 (q, ^3^ *J*_HH_ = 7.1 Hz, 2H, CH_2_O), 2.39–2.27 (m, 2H, CH_2_), 2.00–1.85 (m, 4H, CH_2_ + 2 × CH), 1.78 (dd seen as t, ^3^ *J*_HH_ = 9.0 Hz, 1H, CH), 1.71 (m, 1H, CH_2_), 1.46 (m, 1H, CH_2_), 1.22 (t, ^3^ *J*_HH_ = 7.1 Hz, 2H, CH_3_), 1.12 (m, 1H, CH_2_), 1.00 (m, 1H, CH_2_). ^13^C­{1H} NMR (100 MHz, CDCl_3_): δ 172.0 (C=O), 137.6 (=CH), 132.2 (=CH), 59.8 (CH_2_O), 33.5 (CH_2_), 33.0 (CH_2_), 27.0 (CH_2_), 25.8 (CH_2_), 24.4 (CH), 23.4 (CH), 20.8 (CH), 14.3 (CH_3_). A satisfactory HRMS could not be obtained due to decomposition.
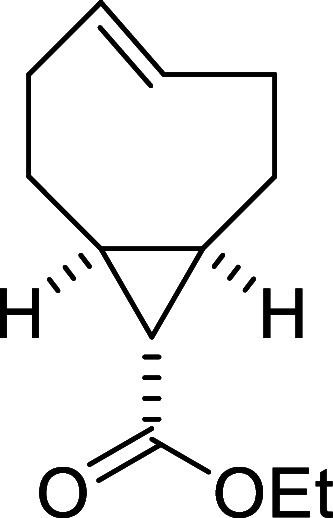


#### General Procedure and Spectral Data for the Synthesis of Functionalized Cinnoline Derivatives **3–5**

Under a nitrogen atmosphere, the corresponding dienophile (0.75 mmol, 1.5 equiv) was added to a solution of the corresponding *N*-carbonyl aryldiazene **2** (0.5 mmol, 1 equiv) in CHCl_3_ (1 mL). The reaction mixture was stirred at room temperature for 1 to 48 h. After completion, the crude reaction mixture was purified by column chromatography, as described for each individual case below, to afford the corresponding cinnoline derivatives **3**, **4**, and **5**.

##### Ethyl (6aS*,12aR*,*Z*)-6a,7,8,11,12,12a-Hexahydrocycloocta­[*c*]­cinnoline-6­(5*H*)-carboxylate (**3a**)

The general procedure was followed using aryldiazene carboxylate **2a** (89 mg, 0.5 mmol) and *cis*,*trans*-cycloocta-1,5-diene (81 mg, 0.75 mmol). After stirring for 7 h at room temperature, the crude reaction mixture was purified by flash-column chromatography (SiO_2_, hexanes/AcOEt 98:2) to afford 141 mg (98%) of **3a** as a brown solid. Mp: 113–115 °C. FTIR (neat) *v*_max_ (cm^–1^) 3315, 3016, 2934, 2858, 1694. ^1^H NMR (400 MHz, CDCl_3_): δ 7.29 (d, ^3^ *J*_HH_ = 7.6 Hz, 1H, CH_ar_), 7.04 (dd seen as t, ^3^ *J*_HH_ = 7.6 Hz, 1H, CH_ar_), 6.91 (dd seen as t, ^3^ *J*_HH_ = 7.6 Hz, 1H, CH_ar_), 6.81 (d, ^3^ *J*_HH_ = 7.6 Hz, 1H, CH_ar_), 6.28 (bs, 1H, NH), 5.83–5.68 (m, 2H, 2 × =CH), 4.44 (ddd seen as broad d, ^3^ *J*_HH_ = 11.6 Hz, 1H, CHN), 4.10 (q, ^3^ *J*_HH_ = 7.1 Hz, 2H, CH_2_O), 2.74 (ddd seen as dt, ^3^ *J*_HH_ = 11.6 Hz, ^3^ *J*_HH_ = 3.8 Hz, 1H, CH), 2.53 (m, 1H, CH_2_), 2.42–2.24 (m, 4H, 2 × CH_2_), 2.14 (m, 1H, CH_2_), 1.79 (m, 1H, CH_2_), 1.58 (m, 1H, CH_2_), 1.20 (t, ^3^ *J*_HH_ = 7.1 Hz, 3H, CH_3_). ^13^C­{1H} NMR (100 MHz, CDCl_3_): δ 155.0 (C=O), 147.3 (C_quat_N), 130.0 (=CH), 129.6 (C_quat_), 129.5 (=CH), 125.7 (CH_ar_), 125.5 (CH_ar_), 121.8 (CH_ar_), 114.4 (CH_ar_), 61.8 (CH_2_O), 59.1 (CHN), 37.7 (CH), 33.0 (CH_2_), 29.0 (CH_2_), 24.1 (CH_2_), 23.5 (CH_2_), 14.4 (CH_3_). HRMS (ESI) *m*/*z*: [M + H]^+^ Calcd for C_17_H_23_N_2_O_2_ 287.1760; Found 287.1746.
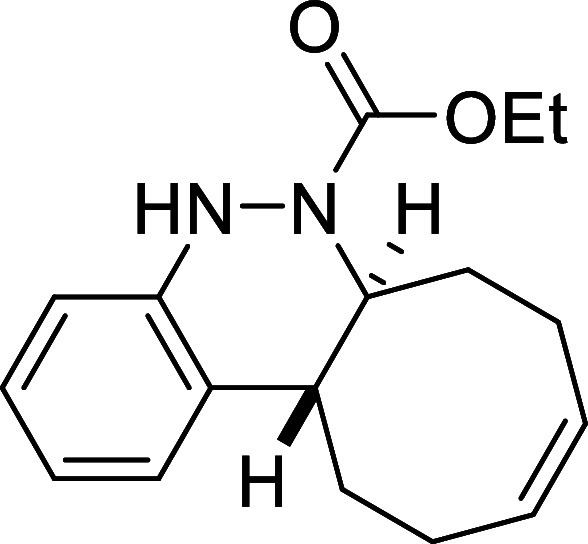


##### Ethyl (6aS*,12aR*,*Z*)-2-Methyl-6a,7,8,11,12,12a-hexahydrocycloocta­[*c*]­cinnoline-6­(5*H*)-carboxylate (**3b**)

The general procedure was followed using aryldiazene carboxylate **2b** (96 mg, 0.5 mmol) and *cis*,*trans*-cycloocta-1,5-diene (81 mg, 0.75 mmol). After stirring for 7 h at room temperature, the crude reaction mixture was purified by flash-column chromatography (SiO_2_, hexanes/AcOEt 98:2) to afford 123 mg (82%) of **3b** as an orange solid. Mp: 110–112 °C. FTIR (neat) *v*_max_ (cm^–1^) 3324, 3017, 2929, 2858, 1687. ^1^H NMR (400 MHz, CDCl_3_): δ 7.12 (s, 1H, CH_ar_), 6.86 (d, ^3^ *J*_HH_ = 7.9 Hz, 1H, CH_ar_), 6.73 (d, ^3^ *J*_HH_ = 7.9 Hz, 1H, CH_ar_), 6.21 (bs, 1H, NH), 5.83–5.68 (m, 2H, 2 × =CH), 4.42 (ddd seen as broad d, ^3^ *J*_HH_ = 11.6 Hz, 1H, CHN), 4.10 (q, ^3^ *J*_HH_ = 7.2 Hz, 2H, CH_2_O), 2.73 (ddd seen as dt, ^3^ *J*_HH_ = 11.6 Hz, ^3^ *J*_HH_ = 3.9 Hz, 1H, CH), 2.54 (m, 1H, CH_2_), 2.42–2.29 (m, 4H, 2 × CH_2_), 2.27 (s, 3H, CH_3_), 2.13 (m, 1H, CH_2_), 1.79 (m, 1H, CH_2_), 1.58 (m, 1H, CH_2_), 1.23 (t, ^3^ *J*_HH_ = 7.2 Hz, 3H, CH_3_). ^13^C­{1H} NMR (100 MHz, CDCl_3_): δ 155.0 (C=O), 144.9 (C_quat_N), 131.0 (C_quat_), 130.0 (=CH), 129.5 (=CH), 129.5 (C_quat_), 126.4 (CH_ar_), 126.0 (CH_ar_), 114.4 (CH_ar_), 61.8 (CH_2_O), 58.9 (CHN), 37.6 (CH), 33.1 (CH_2_), 29.1 (CH_2_), 24.2 (CH_2_), 23.5 (CH_2_), 21.0 (CH_3_), 14.4 (CH_3_). HRMS (ESI) *m*/*z*: [M + H]^+^ Calcd for C_18_H_25_N_2_O_2_ 301.1916; Found 301.1894.
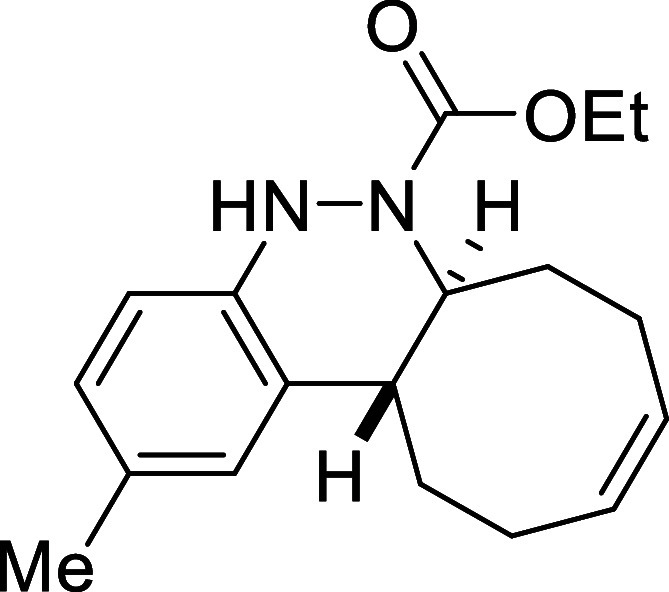


##### Ethyl (6aS*,12aR*,*Z*)-2-Methoxy-6a,7,8,11,12,12a-hexahydrocycloocta­[*c*]­cinnoline-6­(5*H*)-carboxylate (**3c**)

The general procedure was followed using aryldiazene carboxylate **2c** (104 mg, 0.5 mmol) and *cis*,*trans*-cycloocta-1,5-diene (81 mg, 0.75 mmol). After stirring for 7 h at room temperature, the crude reaction mixture was purified by flash-column chromatography (Al_2_O_3_, hexanes/AcOEt 95:5) to afford 142 mg (90%) of **3c** as a brown solid. Mp: 82–84 °C. FTIR (neat) *v*_max_ (cm^–1^) 3333, 2935, 2857, 1692. ^1^H NMR (400 MHz, CDCl_3_): δ 6.87 (d, ^4^ *J*_HH_ = 2.6 Hz, 1H, CH_ar_), 6.74 (d, ^3^ *J*_HH_ = 8.5 Hz, 1H, CH_ar_), 6.59 (dd, ^3^ *J*_HH_ = 8.5 Hz, ^4^ *J*_HH_ = 2.6 Hz, 1H, CH_ar_), 6.03 (bs, 1H, NH), 5.82–5.65 (m, 2H, 2 × =CH), 4.36 (ddd seen as broad d, ^3^ *J*_HH_ = 11.3 Hz, 1H, CHN), 4.08 (q, ^3^ *J*_HH_ = 7.1 Hz, 2H, CH_2_O), 3.73 (s, 3H, CH_3_), 2.72 (ddd seen as dt, ^3^ *J*_HH_ = 11.3 Hz, ^3^ *J*_HH_ = 3.9 Hz, 1H, CH), 2.51 (m, 1H, CH_2_), 2.41–2.21 (m, 4H, 2 × CH_2_), 2.10 (m, 1H, CH_2_), 1.77 (m, 1H, CH_2_), 1.57 (m, 1H, CH_2_), 1.19 (t, ^3^ *J*_HH_ = 7.1 Hz, 3H, CH_3_). ^13^C­{1H} NMR (100 MHz, CDCl_3_): δ 155.3 (C=O), 155.1 (C_quat_O), 141.1 (C_quat_N), 131.5 (C_quat_), 130.2 (=CH), 129.6 (=CH), 115.4 (CH_ar_), 112.8 (CH_ar_), 110.2 (CH_ar_), 62.0 (CH_2_O), 58.8 (CHN), 55.6 (CH_3_O), 38.0 (CH), 33.3 (CH_2_), 29.2 (CH_2_), 24.2 (CH_2_), 23.6 (CH_2_), 14.6 (CH_3_). HRMS (ESI) *m*/*z*: [M + H]^+^ Calcd for C_18_H_25_N_2_O_3_ 317.1865; Found 317.1853.
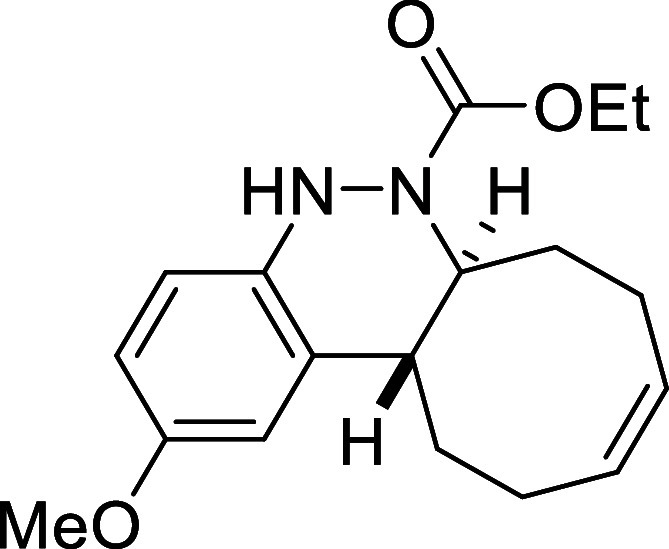


##### Ethyl (6aS*,12aR*,*Z*)-2-(Trifluoromethoxy)-6a,7,8,11,12,12a-hexahydrocycloocta­[*c*]­cinnoline-6­(5*H*)-carboxylate (**3d**)

The general procedure was followed using aryldiazene carboxylate **2d** (131 mg, 0.5 mmol) and *cis*,*trans*-cycloocta-1,5-diene (81 mg, 0.75 mmol). After stirring for 7 h at room temperature, the crude reaction mixture was purified by flash-column chromatography (SiO_2_, hexanes/AcOEt 98:2) to afford 72 mg (39%) of **3d** as a brown solid. Mp: 90–91 °C. FTIR (neat) *v*_max_ (cm^–1^) 3315, 2938, 2858, 1690. ^1^H NMR (400 MHz, CDCl_3_): δ 7.13 (s, 1H, CH_ar_), 6.90 (d, ^3^ *J*_HH_ = 8.5 Hz, 1H, CH_ar_), 6.78 (d, ^3^ *J*_HH_ = 8.5 Hz, 1H, CH_ar_), 6.28 (bs, 1H, NH), 5.82–5.66 (m, 2H, 2 × =CH), 4.40 (ddd seen as broad d, ^3^ *J*_HH_ = 11.6 Hz, 1H, CHN), 4.13–4.07 (m, 2H, CH_2_O), 2.72 (ddd seen as dt, ^3^ *J*_HH_ = 11.6 Hz, ^3^ *J*_HH_ = 3.9 Hz, 1H, CH), 2.51 (m, 1H, CH_2_), 2.41–2.17 (m, 4H, 2 × CH_2_), 2.09 (m, 1H, CH_2_), 1.78 (m, 1H, CH_2_), 1.57 (m, 1H, CH_2_), 1.19 (dd seen as t, ^3^ *J*_HH_ = 7.1 Hz, 3H, CH_3_). ^13^C­{1H} NMR (100 MHz, CDCl_3_): δ 155.2 (C=O), 146.3 (C_quat_N), 144.0 (q, ^3^ *J*_CF_ = 2.0 Hz, C_quat_O), 131.6 (C_quat_), 130.2 (=CH), 129.5 (=CH), 120.5 (q, ^1^ *J*_CF_ = 256.3 Hz, CF_3_), 119.5 (CH_ar_), 118.6 (CH_ar_), 115.1 (CH_ar_), 62.2 (CH_2_O), 58.9 (CHN), 38.0 (CH), 33.1 (CH_2_), 29.0 (CH_2_), 24.0 (CH_2_), 23.5 (CH_2_), 14.5 (CH_3_). ^19^F NMR (376 MHz, CDCl_3_): δ −58.2. HRMS (ESI) *m*/*z*: [M + H]^+^ Calcd for C_18_H_22_F_3_N_2_O_3_ 371.1583; Found 371.1569.
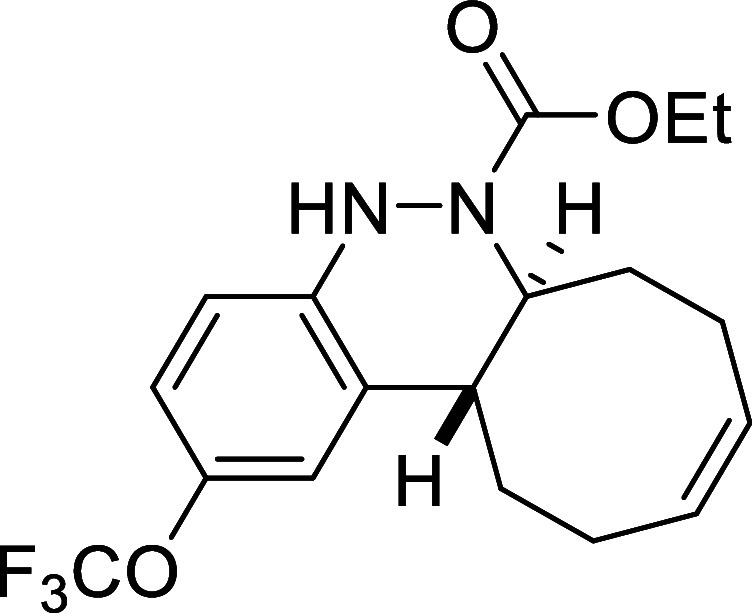


##### Ethyl (6aS*,12aR*,*Z*)-2-(Trifluoromethyl)-6a,7,8,11,12,12a-hexahydrocycloocta­[*c*]­cinnoline-6­(5*H*)-carboxylate (**3e**)

The general procedure was followed using aryldiazene carboxylate **2e** (123 mg, 0.5 mmol) and *cis*,*trans*-cycloocta-1,5-diene (81 mg, 0.75 mmol). After stirring for 3 h at room temperature, the crude reaction mixture was purified by flash-column chromatography (SiO_2_, hexanes/AcOEt 98:2) to afford 44 mg (25%) of **3e** as a yellow solid. Mp: 108–110 °C. FTIR (neat) *v*_max_ (cm^–1^) 3322, 2928, 2860, 1991. ^1^H NMR (400 MHz, CDCl_3_): δ 7.51 (s, 1H, CH_ar_), 7.29 (d, ^3^ *J*_HH_ = 8.2 Hz, 1H, CH_ar_), 6.86 (d, ^3^ *J*_HH_ = 8.2 Hz, 1H, CH_ar_), 6.39 (bs, 1H, NH), 5.82–5.67 (m, 2H, 2 × =CH), 4.45 (ddd seen as broad d, ^3^ *J*_HH_ = 11.6 Hz, 1H, CHN), 4.10 (q, ^3^ *J*_HH_ = 7.1 Hz, 2H, CH_2_O), 2.74 (ddd seen as dt, ^3^ *J*_HH_ = 11.6, ^3^ *J*_HH_ = 3.7 Hz, 1H, CH), 2.52 (m, 1H, CH_2_), 2.41–2.23 (m, 4H, 2 × CH_2_), 2.09 (m, 1H, CH_2_), 1.78 (m, 1H, CH_2_), 1.60 (m, 1H, CH_2_), 1.20 (t, ^3^ *J*_HH_ = 7.1 Hz, 3H, CH_3_). ^13^C­{1H} NMR (100 MHz, CDCl_3_): δ 155.2 (C=O), 150.5 (C_quat_N), 130.2 (=CH), 130.1 (C_quat_), 129.6 (=CH), 124.5 (q, ^1^ *J*_CF_ = 271.2 Hz, CF_3_), 123.9 (q, ^2^ *J*_CF_ = 32.3 Hz, C_quat_), 123.2 (q, ^3^ *J*_CF_ = 3.7 Hz, CH_ar_), 123.0 (q, ^3^ *J*_CF_ = 3.9 Hz, CH_ar_), 114.3 (CH_ar_), 62.3 (CH_2_O), 59.3 (CHN), 37.9 (CH), 33.1 (CH_2_), 29.0 (CH_2_), 24.1 (CH_2_), 23.6 (CH_2_), 14.5 (CH_3_). ^19^F NMR (376 MHz, CDCl_3_): δ −61.6. HRMS (ESI) *m*/*z*: [M + H]^+^ Calcd for C_18_H_22_F_3_N_2_O_2_ 355.1633; Found 355.1610.
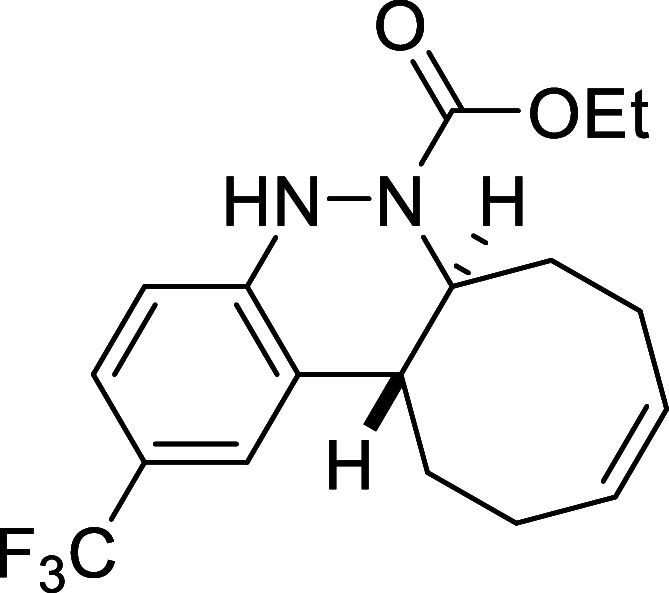


##### Ethyl (6aS*,12aR*,*Z*)-2-Bromo-6a,7,8,11,12,12a-hexahydrocycloocta­[*c*]­cinnoline-6­(5*H*)-carboxylate (**3f**)

The general procedure was followed using aryldiazene carboxylate **2f** (129 mg, 0.5 mmol) and *cis*,*trans*-cycloocta-1,5-diene (81 mg, 0.75 mmol). After stirring for 5 h at room temperature, the crude reaction mixture was purified by flash-column chromatography (SiO_2_, hexanes/AcOEt 98:2) to afford 171 mg (94%) of **3f** as a brown solid. Mp: 139–141 °C. FTIR (neat) *v*_max_ (cm^–1^) 3298, 2982, 2935, 2856, 1677. ^1^H NMR (400 MHz, CDCl_3_): δ 7.38 (m, 1H, CH_ar_), 7.13 (m, 1H, CH_ar_), 6.67 (d, ^3^ *J*_HH_ = 8.3 Hz, 1H, CH_ar_), 6.23 (bs, 1H, NH), 5.80–5.64 (m, 2H, 2 × =CH), 4.39 (ddd seen as broad d, ^3^ *J*_HH_ = 11.4 Hz, 1H, CHN), 4.08 (q, ^3^ *J*_HH_ = 7.1 Hz, 2H, CH_2_O), 2.69 (ddd seen as dt, ^3^ *J*_HH_ = 11.4 Hz, ^3^ *J*_HH_ = 3.9 Hz, 1H, CH), 2.49 (m, 1H, CH_2_), 2.39–2.16 (m, 4H, 2 × CH_2_), 2.07 (m, 1H, CH_2_), 1.76 (m, 1H, CH_2_), 1.55 (m, 1H, CH_2_), 1.18 (t, ^3^ *J*_HH_ = 7.1 Hz, 3H, CH_3_). ^13^C­{1H} NMR (100 MHz, CDCl_3_): δ 155.2 (C=O), 146.6 (C_quat_N), 132.1 (C_quat_), 130.1 (=CH), 129.4 (=CH), 128.9 (CH_ar_), 128.3 (CH_ar_), 116.0 (CH_ar_), 114.5 (C_quat_Br), 62.1 (CH_2_O), 59.0 (CHN), 37.8 (CH), 33.1 (CH_2_), 28.9 (CH_2_), 24.0 (CH_2_), 23.5 (CH_2_), 14.5 (CH_3_). HRMS (ESI) *m*/*z*: [M + H]^+^ Calcd for C_17_H_22_BrN_2_O_2_ 365.0865; Found 365.0848.
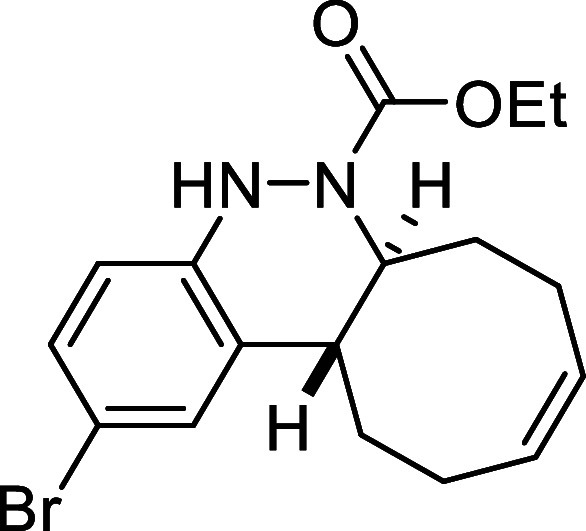


##### Ethyl (6aS*,12aR*,*Z*)-2-Fluoro-6a,7,8,11,12,12a-hexahydrocycloocta­[*c*]­cinnoline-6­(5*H*)-carboxylate (**3g**)

The general procedure was followed using aryldiazene carboxylate **2g** (98 mg, 0.5 mmol) and *cis*,*trans*-cycloocta-1,5-diene (81 mg, 0.75 mmol). After stirring for 4 h at room temperature, the crude reaction mixture was purified by flash-column chromatography (SiO_2_, hexanes/AcOEt 97:3) to afford 121 mg (80%) of **3g** as a brown solid. Mp: 131–133 °C. FTIR (neat) *v*_max_ (cm^–1^) 3296, 2931, 2858, 1673. ^1^H NMR (400 MHz, CDCl_3_): δ 6.98 (d, ^3^ *J*_HH_ = 9.8 Hz, 1H, CH_ar_), 6.75–6.68 (m, 2H, 2 × CH_ar_), 6.20 (bs, 1H, NH), 5.78 (m, 1H, =CH), 5.67 (m, 1H, =CH), 4.37 (ddd seen as broad d, ^3^ *J*_HH_ = 11.6 Hz, 1H, CHN), 4.10–4.04 (m, 2H, CH_2_O), 2.68 (m, 1H, CH), 2.50 (m, 1H, CH_2_), 2.39–2.03 (m, 5H, 2 × CH_2_ + 1H from CH_2_), 1.77 (m, 1H, CH_2_), 1.54 (m, 1H, CH_2_), 1.18 (m, 3H, CH_3_). ^13^C­{1H} NMR (100 MHz, CDCl_3_): δ 158.3 (d, ^1^ *J*_CF_ = 238.5 Hz, C_quat_F), 155.2 (C=O), 143.6 (C_quat_N), 131.9 (d, ^3^ *J*_CF_ = 5.0 Hz, C_quat_), 130.1 (=CH), 129.3 (=CH), 115.3 (d, ^3^ *J*_CF_ = 8.2 Hz, CH_ar_), 112.9 (d, ^2^ *J*_CF_ = 23.9 Hz, CH_ar_), 111.8 (d, ^2^ *J*_CF_ = 22.8 Hz, CH_ar_), 61.9 (CH_2_O), 58.7 (CHN), 37.9 (CH), 33.1 (CH_2_), 28.9 (CH_2_), 23.9 (CH_2_), 23.4 (CH_2_), 14.4 (CH_3_). ^19^F NMR (376 MHz, CDCl_3_): δ −121.1. HRMS (ESI) *m*/*z*: [M + H]^+^ Calcd for C_17_H_22_FN_2_O_2_ 305.1665; Found 305.1657.
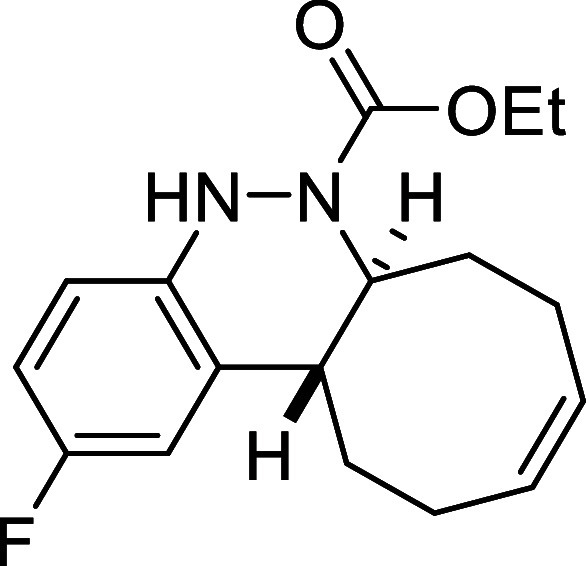


##### Ethyl (6aS*,12aR*,*Z*)-4-Fluoro-6a,7,8,11,12,12a-hexahydrocycloocta­[*c*]­cinnoline-6­(5*H*)-carboxylate (**3h**)

The general procedure was followed using aryldiazene carboxylate **2h** (98 mg, 0.5 mmol) and *cis*,*trans*-cycloocta-1,5-diene (81 mg, 0.75 mmol). After stirring for 6 h at room temperature, the crude reaction mixture was purified by flash-column chromatography (SiO_2_, hexanes/AcOEt 97:3) to afford 112 mg (74%) of **3h** as a white solid. Mp: 98–99 °C. FTIR (neat) *v*_max_ (cm^–1^) 3349, 3019, 2930, 2860, 1705. ^1^H NMR (400 MHz, CDCl_3_): δ 7.04 (d, ^3^ *J*_HH_ = 7.5 Hz, 1H, CH_ar_), 6.88–6.76 (m, 2H, 2 × CH_ar_), 6.61 (bs, 1H, NH), 5.80 (m, 1H, =CH), 5.69 (m, 1H, =CH), 4.45 (ddd seen as broad d, ^3^ *J*_HH_ = 11.6 Hz, 1H, CHN), 4.08 (q, ^3^ *J*_HH_ = 7.1 Hz, 2H, CH_2_O), 2.72 (ddd seen as dt, ^3^ *J*_HH_ = 11.6 Hz, ^3^ *J*_HH_ = 3.8 Hz, 1H, CH), 2.51 (m, 1H, CH_2_), 2.41–2.21 (m, 4H, 2 × CH_2_), 2.11 (m, 1H, CH_2_), 1.80 (m, 1H, CH_2_), 1.59 (m, 1H, CH_2_), 1.18 (t, ^3^ *J*_HH_ = 7.1 Hz, 3H, CH_3_). ^13^C­{1H} NMR (100 MHz, CDCl_3_): δ 155.3 (C=O), 150.4 (d, ^1^ *J*_CF_ = 240.0 Hz, C_quat_F), 135.5 (d, ^2^ *J*_CF_ = 14.5 Hz, C_quat_N), 132.3 (C_quat_), 130.2 (=CH), 129.5 (=CH), 121.2 (d, ^3^ *J*_CF_ = 7.3 Hz, CH_ar_), 121.1 (d, ^4^ *J*_CF_ = 3.2 Hz, CH_ar_), 111.9 (d, ^2^ *J*_CF_ = 18.2 Hz, CH_ar_), 62.1 (CH_2_O), 59.0 (CHN), 37.6 (d, ^4^ *J*_CF_ = 2.6 Hz, CH), 33.1 (CH_2_), 29.2 (CH_2_), 24.1 (CH_2_), 23.5 (CH_2_), 14.3 (CH_3_). ^19^F NMR (376 MHz, CDCl_3_): δ −137.8. ESI- HRMS (ESI) *m*/*z*: [M + H]^+^ Calcd for C_17_H_22_FN_2_O_2_ 305.1665; Found 305.1653.
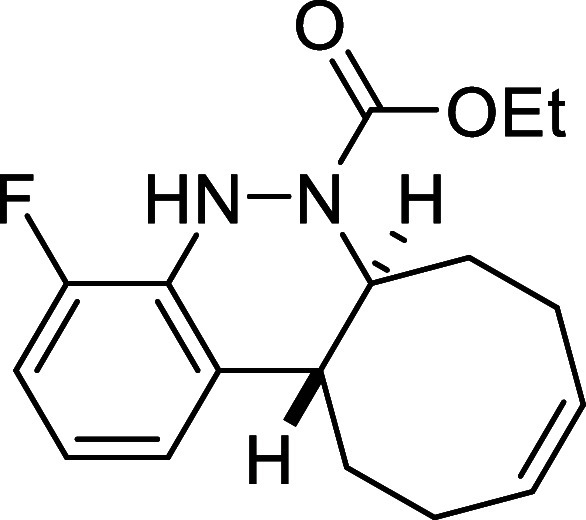


##### (6aS*,12aR*,*Z*)-6-(Ethoxycarbonyl)-5,6,6a,7,8,11,12,12a-octahydrocycloocta­[*c*]­cinnoline-2-carboxylic Acid (**3i**)

The general procedure was followed using aryldiazene carboxylate **2i** (111 mg, 0.5 mmol) and *cis*,*trans*-cycloocta-1,5-diene (81 mg, 0.75 mmol). After stirring for 1 h at room temperature, the crude reaction mixture was purified by recrystallization in hexanes/CH_2_Cl_2_ to afford 143 mg (87%) of **3i** as a white solid. Mp: 188–190 °C. FTIR (neat) *v*_max_ (cm^–1^) 3293, 2982, 2934, 1678. ^1^H NMR (400 MHz, CDCl_3_): δ 12.08 (bs, 1H, COOH), 8.05 (s, 1H, CH_ar_), 7.82 (d, ^3^ *J*_HH_ = 8.2 Hz, 1H, CH_ar_), 6.85 (d, ^3^ *J*_HH_ = 8.2 Hz, 1H, CH_ar_), 6.64 (bs, 1H, NH), 5.82–5.68 (m, 2H, 2 × =CH), 4.46 (ddd seen as broad d, ^3^ *J*_HH_ = 11.2 Hz, 1H, CHN), 4.11 (q, ^3^ *J*_HH_ = 7.1 Hz, 2H, CH_2_O), 2.75 (ddd seen as dt, ^3^ *J*_HH_ = 11.2 Hz, ^3^ *J*_HH_ = 3.7 Hz, 1H, CH), 2.53 (m, 1H, CH_2_), 2.43–2.24 (m, 4H, 2 × CH_2_), 2.08 (m, 1H, CH_2_), 1.78 (m, 1H, CH_2_), 1.61 (m, 1H, CH_2_), 1.20 (t, ^3^ *J*_HH_ = 7.1 Hz, 3H, CH_3_). ^13^C­{1H} NMR (100 MHz, CDCl_3_): δ 171.9 (COOH), 155.2 (C=O), 152.4 (C_quat_N), 130.1 (=CH), 129.7 (=CH), 129.2 (C_quat_), 128.6 (CH_ar_), 128.5 (CH_ar_), 122.5 (C_quat_COOH), 114.2 (CH_ar_), 62.4 (CH_2_O), 59.3 (CHN), 37.9 (CH), 33.0 (CH_2_), 29.1 (CH_2_), 24.2 (CH_2_), 23.6 (CH_2_), 14.6 (CH_3_). HRMS (ESI) *m*/*z*: [M + H]^+^ Calcd for C_18_H_23_N_2_O_4_ 331.1658; Found 331.1661.
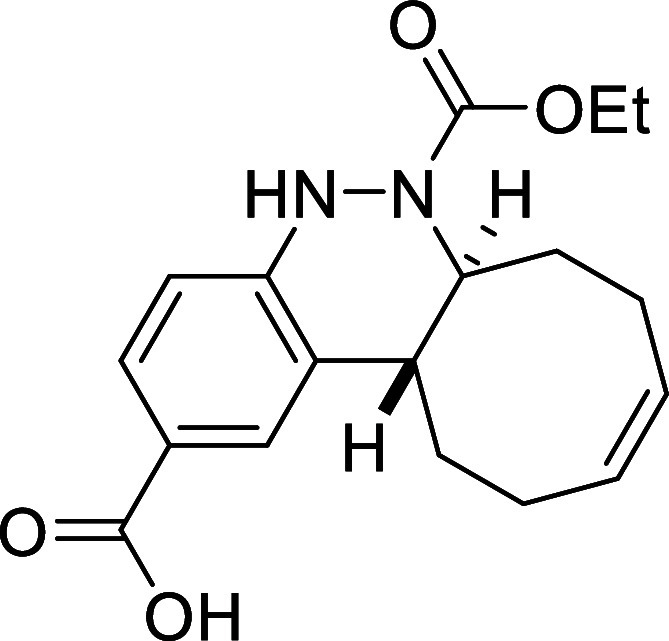


##### Ethyl (6aS*,12aR*,*Z*)-6a,7,8,11,12,12a-Hexahydrobenzo­[*h*]­cycloocta­[*c*]­cinnoline-6­(5*H*)-carboxylate (**3j**)

The general procedure was followed using aryldiazene carboxylate **2j** (114 mg, 0.5 mmol) and *cis*,*trans*-cycloocta-1,5-diene (81 mg, 0.75 mmol). After stirring for 50 min at room temperature, the crude reaction mixture was purified by flash-column chromatography (SiO_2_, hexanes/AcOEt 95:5) to afford 165 mg of **3j** (98%) as a brown oil. FTIR (neat) *v*_max_ (cm^–1^) 3389, 3014, 2930, 2857, 1697. ^1^H NMR (400 MHz, CDCl_3_): δ 7.89 (m, 1H, CH_ar_), 7.78 (d, ^3^ *J*_HH_ = 8.5, Hz, 1H, CH_ar_), 7.50–7.41 (m, 4H, 4 × CH_ar_), 7.11 (bs, 1H, NH), 5.88–5.73 (m, 2H, 2 × =CH), 4.62 (m, 1H, CHN), 4.15–4.04 (m, 2H, CH_2_O), 2.92 (ddd seen as dt, ^3^ *J*_HH_ = 12.2 Hz, ^3^ *J*_HH_ = 3.6, Hz, 1H, CH), 2.60 (m, 1H, CH_2_), 2.48–2.29 (m, 4H, CH_2_), 2.17 (m, 1H, CH_2_), 1.89 (m, 1H, CH_2_), 1.69 (m, 1H, CH_2_), 1.15 (dd seen as t, ^3^ *J*_HH_ = 7.1 Hz, 3H, CH_3_). ^13^C­{1H} NMR (100 MHz, CDCl_3_): δ 155.2 (C=O), 142.1 (C_quat_N), 131.6 (C_quat_), 130.1 (=CH), 129.6 (=CH), 128.0 (CH_ar_), 125.5 (CH_ar_), 125.2 (CH_ar_), 124.1 (C_quat_), 123.8 (CH_ar_), 122.6 (C_quat_), 121.2 (CH_ar_), 119.2 (CH_ar_), 62.0 (CH_2_O), 58.9 (CHN), 38.1 (CH), 32.8 (CH_2_), 29.4 (CH_2_), 24.4 (CH_2_), 23.7 (CH_2_), 14.3 (CH_3_). HRMS (ESI) *m*/*z*: [M + H]^+^ Calcd for C_21_H_25_N_2_O_2_ 337.1916; Found 337.1925.
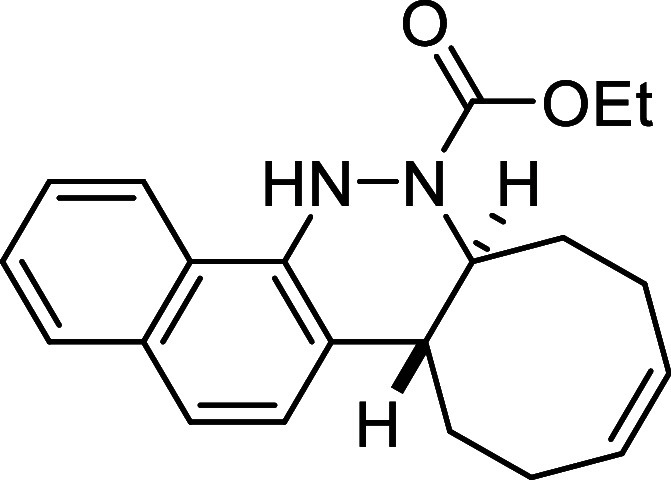


##### *tert*-Butyl (6aS*,12aR*,Z)-6a,7,8,11,12,12a-Hexahydrocycloocta­[*c*]­cinnoline-6­(5*H*)-carboxylate (**3k**)

The general procedure was followed using aryldiazene carboxylate **2k** (103 mg, 0.5 mmol) and *cis*,*trans*-cycloocta-1,5-diene (81 mg, 0.75 mmol). After stirring for 48 h at room temperature, the crude reaction mixture was purified by flash-column chromatography (SiO_2_, hexanes/AcOEt 98:2) to afford 122 mg (78%) of **3k** as a white solid. Mp: 139–140 °C. FTIR (neat) *v*_max_ (cm^–1^) 3361, 2974, 2931, 2857, 1687. ^1^H NMR (400 MHz, CDCl_3_): δ 7.28 (d, ^3^ *J*_HH_ = 7.7 Hz, 1H, CH_ar_), 7.04 (dd seen as t, ^3^ *J*_HH_ = 7.7, 1H, CH_ar_), 6.90 (dd seen as t, ^3^ *J*_HH_ = 7.7 Hz, 1H, CH_ar_), 6.77 (d, ^3^ *J*_HH_ = 7.7 Hz, 1H, CH_ar_), 6.19 (bs, 1H, NH), 5.82–5.68 (m, 2H, 2 × =CH), 4.44 (ddd seen as broad d, ^3^ *J*_HH_ = 11.4 Hz, 1H, CHN), 2.71 (ddd seen as dt, ^3^ *J*_HH_ = 11.4 Hz, ^3^ *J*_HH_ = 3.9 Hz, 1H, CH), 2.52 (m, 1H, CH_2_), 2.39–2.25 (m, 4H, 2 × CH_2_), 2.11 (m, 1H, CH_2_), 1.77 (m, 1H, CH_2_), 1.57 (m, 1H, CH_2_), 1.36 (s, 9H, 3 × CH_3_). ^13^C­{1H} NMR (100 MHz, CDCl_3_): δ 154.6 (C=O), 147.6 (C_quat_N), 130.0 (=CH), 129.7 (C_quat_), 129.6 (=CH), 125.8 (CH_ar_), 125.4 (CH_ar_), 121.6 (CH_ar_), 114.4 (CH_ar_), 80.8 (C_quat_O), 59.0 (CHN), 37.8 (CH), 33.0 (CH_2_), 29.2 (CH_2_), 28.1 (3 × CH_3_), 24.3 (CH_2_), 23.6 (CH_2_). HRMS (ESI) *m*/*z*: [M + Na]^+^ Calcd for C_19_H_26_N_2_NaO_2_ 337.1892; Found 337.1878.
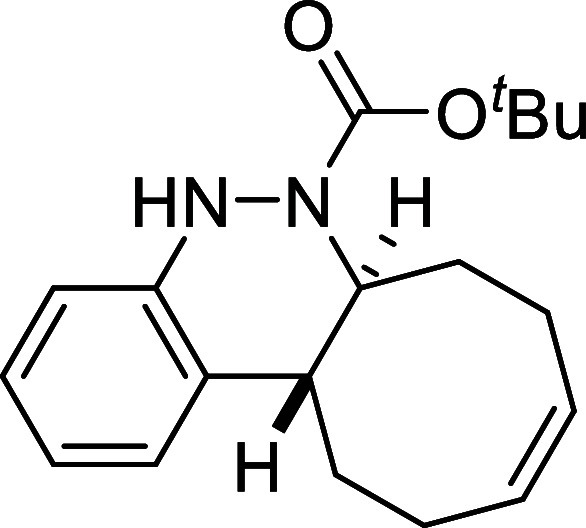


##### 2,2,2-Trichloroethyl (6aS*,12aR*,*Z*)-6a,7,8,11,12,12a-Hexahydrocycloocta­[*c*]­cinnoline-6­(5*H*)-carboxylate (**3l**)

The general procedure was followed using aryldiazene carboxylate **2l** (141 mg, 0.5 mmol) and *cis*,*trans*-cycloocta-1,5-diene (81 mg, 0.75 mmol). After stirring for 1.5 h at room temperature, the crude reaction mixture was purified by flash-column chromatography (SiO_2_, hexanes/AcOEt 98:2) to afford 160 mg (82%) of **3l** as a yellow solid. Mp: 118–120 °C. FTIR (neat) *v*_max_ (cm^–1^) 3321, 3017, 2945, 2858, 1707. ^1^H NMR (400 MHz, CDCl_3_): δ 7.31 (d, ^3^ *J*_HH_ = 7.7 Hz, 1H, CH_ar_), 7.07 (dd seen as t, ^3^ *J*_HH_ = 7.7, Hz, 1H, CH_ar_), 6.95 (dd seen as t, ^3^ *J*_HH_ = 7.7 Hz, 1H, CH_ar_), 6.85 (d, ^3^ *J*_HH_ = 7.7 Hz, 1H, CH_ar_), 6.45 (bs, 1H, NH), 5.84–5.71 (m, 2H, 2 × =CH), 4.88–4.66 (m, 2H, CH_2_O), 4.57 (ddd seen as dt, ^3^ *J*_HH_ = 11.8 Hz, ^3^ *J*_HH_ = 3.4 Hz, 1H, CHN), 2.81 (ddd seen as dt, ^3^ *J*_HH_ = 11.8 Hz, ^3^ *J*_HH_ = 3.8 Hz, 1H, CH), 2.53 (m, 1H, CH_2_), 2.39–2.28 (m, 4H, 2 × CH_2_), 2.20 (m, 1H, CH_2_), 1.87 (m, 1H, CH_2_), 1.62 (m, 1H, CH_2_). ^13^C­{1H} NMR (100 MHz, CDCl_3_): δ 152.8 (C=O), 146.8 (C_quat_N), 129.9 (=CH), 129.6 (=CH), 129.1 (C_quat_), 125.9 (CH_ar_), 125.8 (CH_ar_), 122.2 (CH_ar_), 114.7 (CH_ar_), 95.2 (CCl_3_), 75.1 (CH_2_O), 59.5 (CHN), 37.9 (CH), 33.0 (CH_2_), 29.1 (CH_2_), 24.3 (CH_2_), 23.3 (CH_2_). HRMS (ESI) *m*/*z*: [M + H]^+^ Calcd for C_17_H_20_Cl_3_N_2_O_2_ 389.0590; Found 389.0585.
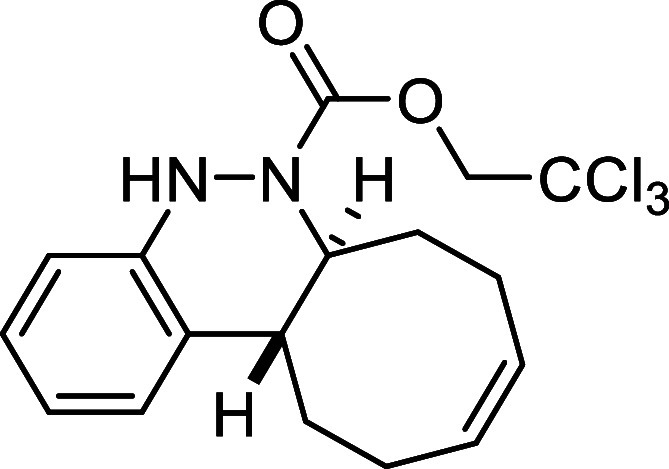


##### Phenyl (6aS*,12aR*,*Z*)-6a,7,8,11,12,12a-Hexahydrocycloocta­[*c*]­cinnoline-6­(5*H*)-carboxylate (**3m**)

The general procedure was followed using aryldiazene carboxylate **2m** (113 mg, 0.5 mmol) and *cis*,*trans*-cycloocta-1,5-diene (81 mg, 0.75 mmol). After stirring for 2 h at room temperature, the crude reaction mixture was purified by flash-column chromatography (SiO_2_, hexanes/AcOEt 98:2) to afford 115 mg (69%) of **3m** as an orange solid. Mp: 102–104 °C. FTIR (neat) *v*_max_ (cm^–1^) 3315, 3016, 2935, 2861, 1709. ^1^H NMR (400 MHz, CDCl_3_): δ 7.38–7.32 (m, 3H, 3 × CH_ar_), 7.20 (m, 1H, CH_ar_), 7.13–6.98 (m, 4H, 4 × CH_ar_), 6.88 (m, 1H, CH_ar_), 6.47 (bs, 1H, NH), 5.88–5.73 (m, 2H, 2 × =CH), 4.62 (m, 1H, CHN), 2.87 (ddd seen as broad d, ^3^ *J*_HH_ = 11.4 Hz, 1H, CH), 2.56 (m, 1H, CH_2_), 2.49–2.23 (m, 5H, 2 × CH_2_ + 1H from CH_2_), 1.96 (m, 1H, CH_2_), 1.68 (m, 1H, CH_2_). ^13^C­{1H} NMR (100 MHz, CDCl_3_): δ 153.2 (C=O), 150.9 (C_quat_O), 147.1 (C_quat_N), 130.0 (=CH), 129.7 (=CH), 129.3 (C_quat_), 129.2 (2 × CH_ar_), 125.9 (CH_ar_), 125.9 (CH_ar_), 125.5 (CH_ar_), 122.3 (CH_ar_), 121.5 (2 × CH_ar_), 114.7 (CH_ar_), 59.9 (CHN), 37.9 (CH), 33.2 (CH_2_), 29.1 (CH_2_), 24.2 (CH_2_), 23.5 (CH_2_). HRMS (ESI) *m*/*z*: [M + H]^+^ Calcd for C_21_H_23_N_2_O_2_ 335.1760; Found 335.1750.
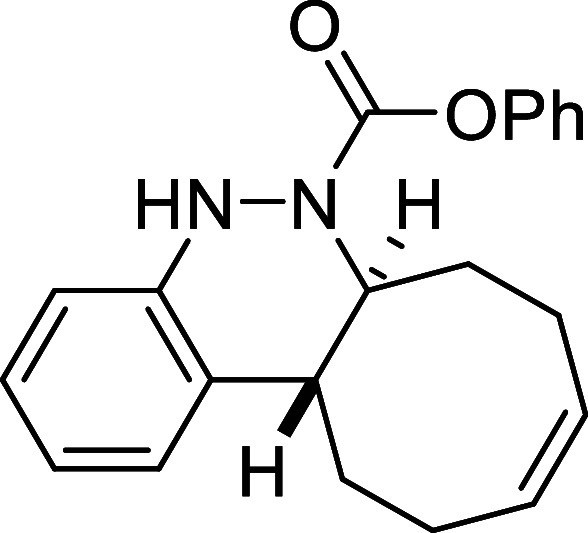


##### Benzyl (6aS*,12aR*,*Z*)-6a,7,8,11,12,12a-Hexahydrocycloocta­[*c*]­cinnoline-6­(5*H*)-carboxylate (**3n**)

The general procedure was followed using aryldiazene carboxylate **2n** (60 mg, 0.25 mmol) and *cis*,*trans*-cycloocta-1,5-diene (41 mg, 0.375 mmol). After stirring for 8 h at room temperature, the crude reaction mixture was purified by flash-column chromatography (SiO_2_, hexanes/AcOEt 96:4) to afford 62 mg (71%) of **3n** as a white solid. Mp: 132–134 °C. FTIR (neat) *v*_max_ (cm^–1^) 3321, 3016, 2940, 2858, 1695. ^1^H NMR (400 MHz, CDCl_3_): δ 7.38–7.25 (m, 6H, 6 × CH_ar_), 7.07 (m, 1H, CH_ar_), 6.95 (dd seen as t, ^3^ *J*_HH_ = 7.6, 1H, CH_ar_), 6.81 (m, 1H, CH_ar_), 6.28 (bs, 1H, NH), 5.84–5.69 (m, 2H, 2 × =CH), 5.15–5.07 (m, 2H, CH_2_O), 4.52 (ddd seen as broad d, ^3^ *J*_HH_ = 11.5 Hz, 1H, CHN), 2.78 (ddd seen as dt, ^3^ *J*_HH_ = 11.5 Hz, ^3^ *J*_HH_ = 3.8 Hz, 1H, CH), 2.53 (m, 1H, CH_2_), 2.42–2.26 (m, 4H, 2 × CH_2_), 2.16 (m, 1H, CH_2_), 1.81 (m, 1H, CH_2_), 1.60 (m, 1H, CH_2_). ^13^C­{1H} NMR (100 MHz, CDCl_3_): δ 155.0 (C=O), 147.4 (C_quat_N), 136.2 (C_quat_), 130.1 (=CH), 129.7 (C_quat_), 129.6 (=CH), 128.4 (2 × CH_ar_), 128.0 (CH_ar_), 127.5 (2 × CH_ar_), 125.9 (CH_ar_), 125.7 (CH_ar_), 122.1 (CH_ar_), 114.6 (CH_ar_), 67.5 (CH_2_O), 59.4 (CHN), 37.8 (CH), 33.1 (CH_2_), 29.1 (CH_2_), 24.3 (CH_2_), 23.5 (CH_2_). HRMS (ESI) *m*/*z*: [M + H]^+^ Calcd for C_22_H_25_N_2_O_2_ 349.1916; Found 349.1918.
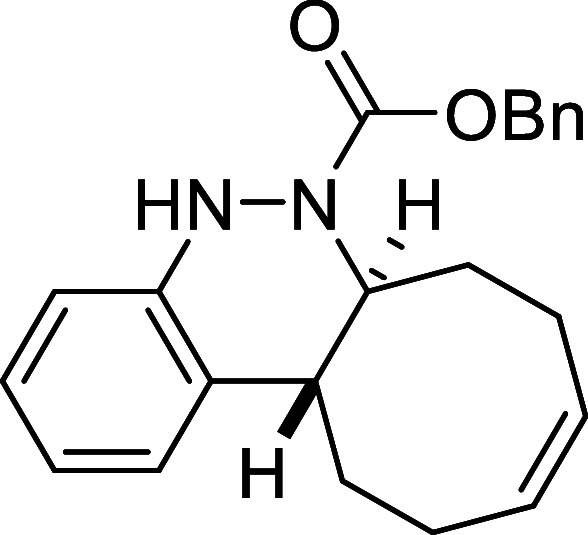


##### Allyl (6aS*,12aR*,*Z*)-6a,7,8,11,12,12a-Hexahydrocycloocta­[*c*]­cinnoline-6­(5*H*)-carboxylate (**3o**)

The general procedure was followed using aryldiazene carboxylate **2o** (95 mg, 0.5 mmol) and *cis*,*trans*-cycloocta-1,5-diene (81 mg, 0.75 mmol). After stirring for 5 h at room temperature, the crude reaction mixture was purified by flash-column chromatography (SiO_2_, hexanes/AcOEt 98:2) to afford 75 mg (50%) of **3o** as a white solid. Mp: 80–81 °C. FTIR (neat) *v*_max_ (cm^–1^) 3310, 3015, 2933, 2858, 1690. ^1^H NMR (400 MHz, CDCl_3_): δ 7.30 (d, ^3^ *J*_HH_ = 7.7 Hz, 1H, CH_ar_), 7.05 (dd seen as t, ^3^ *J*_HH_ = 7.7 Hz, 1H, CH_ar_), 6.92 (dd seen as t, ^3^ *J*_HH_ = 7.7 Hz, 1H, CH_ar_), 6.82 (d, ^3^ *J*_HH_ = 7.7 Hz, 1H, CH_ar_), 6.28 (bs, 1H, NH), 5.90–5.68 (m, 3H, 3× =CH), 5.23–5.15 (m, 2H, =CH_2_), 4.61–4.51 (m, 2H, CH_2_O), 4.47 (m, 1H, CHN), 2.76 (ddd seen as dt, ^3^ *J*_HH_ = 12.1 Hz, ^3^ *J*_HH_ = 3.9 Hz, 1H, CH), 2.53 (m, 1H, CH_2_), 2.42–2.25 (m, 4H, 2 × CH_2_), 2.15 (m, 1H, CH_2_), 1.80 (m, 1H, CH_2_), 1.59 (m, 1H, CH_2_). ^13^C­{1H} NMR (100 MHz, CDCl_3_): δ 154.7 (C=O), 147.3 (C_quat_N), 132.4 (CH=CH_2_), 130.1 (=CH), 129.7 (C_quat_), 129.6 (=CH), 125.8 (CH_ar_), 125.7 (CH_ar_), 122.0 (CH_ar_), 117.2 (=CH_2_), 114.6 (CH_ar_), 66.3 (CH_2_O), 59.3 (CHN), 37.8 (CH), 33.1 (CH_2_), 29.1 (CH_2_), 24.3 (CH_2_), 23.5 (CH_2_). HRMS (ESI) *m*/*z*: [M + H]^+^ Calcd for C_19_H_23_N_2_O_2_ 299.1760; Found 299.1748.
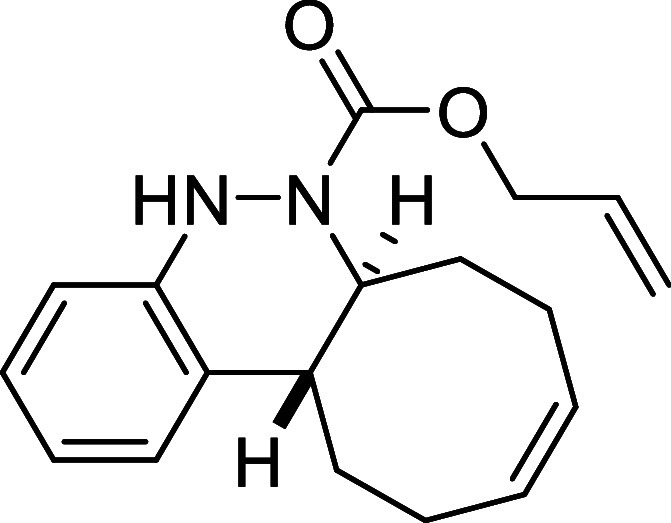


##### (*Z*)-5,7,8,11,12,12a-Hexahydrocycloocta­[*c*]­cinnoline (**4**)

The general procedure was followed using *N*-acetyl aryldiazene **2p** (74 mg, 0.5 mmol) and *cis*,*trans*-cycloocta-1,5-diene (81 mg, 0.75 mmol). After stirring for 1 h at room temperature, the crude reaction mixture was purified by flash-column chromatography (SiO_2_, hexanes/AcOEt 90:10) to afford 8 mg (8%) of **4** as a white solid. Mp: 130–133 °C. FTIR (neat) *v*_max_ (cm^–1^) 3009, 3015, 2928, 1598. ^1^H NMR (400 MHz, CDCl_3_): δ 7.36 (bs, 1H, NH), 7.13 (ddd, ^3^ *J*_HH_ = 7.9 Hz, ^3^ *J*_HH_ = 7.3 Hz, ^4^ *J*_HH_ = 1.5 Hz, 1H, CH_ar_), 7.05 (dd, ^3^ *J*_HH_ = 7.4 Hz, ^4^ *J*_HH_ = 1.5 Hz, 1H, CH_ar_), 6.98 (ddd seen as td, ^3^ *J*_HH_ = 7.4 Hz, ^4^ *J*_HH_ = 1.3 Hz, 1H, CH_ar_), 6.77 (m, 1H, CH_ar_), 5.85–5.71 (m, 2H, 2 × =CH), 3.51 (dd, ^3^ *J*_HH_ = 11.8 Hz, ^3^ *J*_HH_ = 4.3 Hz, 1H, CH), 2.62 (m, 1H, CH_2_), 2.52 (m, 1H, CH_2_), 2.36–2.19 (m, 3H, 2 × CH_2_ + 1H from CH_2_), 2.13 (m, 1H, CH_2_), 1.57 (m, 1H, CH_2_), 1.57 (m, 1H, CH_2_). ^13^C­{1H} NMR (100 MHz, CDCl_3_): δ 150.1 (C=N), 140.3 (C_quat_N), 130.6 (=CH), 129.8 (=CH), 126.5 (CH_ar_), 126.4 (CH_ar_), 122.5 (C_quat_), 122.5 (CH_ar_), 112.1 (CH_ar_) 40.8 (CH), 37.6 (CH_2_), 30.3 (CH_2_), 25.2 (CH_2_), 24.3 (CH_2_). HRMS (ESI) *m*/*z*: [M + H]^+^ Calcd for C_14_H_17_N_2_ 213.1392; Found 213.1394.
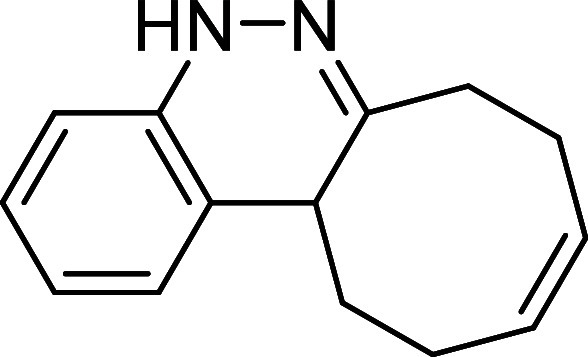


##### Diethyl (6aS*,8aR*,9R*,9aS*,11aR*)-5,6a,7,8,8a,9,9a,10,11,11a-Decahydro-6*H*-cyclopropa­[5,6]­cycloocta­[1,2-*c*]­cinnoline-6,9-dicarboxylate (**5a**) and Diethyl (6aS*,8aS*,9S*,9aR*,11aR*)-5,6a,7,8,8a,9,9a,10,11,11a-Decahydro-6*H*-cyclopropa­[5,6]­cycloocta­[1,2-*c*]­cinnoline-6,9-dicarboxylate (**5a′**)

The general procedure was followed using aryldiazene carboxylate **2a** (89 mg, 0.5 mmol) and ethyl (1R*,8S*,9r*,*E*)-bicyclo­[6.1.0]­non-4-ene-9-carboxylate **VI** (146 mg, 0.75 mmol). After stirring for 8 h at room temperature, the crude reaction mixture was purified by flash-column chromatography (SiO_2_, hexanes/AcOEt 95:5) to afford 185 mg (>99%) of an inseparable mixture of **5a** and **5a′** as a brown oil. FTIR (neat) *v*_max_ (cm^–1^) 3333, 2966, 2903, 1717, 1692. ^1^H NMR (400 MHz, CDCl_3_): δ 7.23–7.27 (m, 2H, CH_ar,major_ + CH_ar,minor_), 7.05–7.00 (m, 2H, CH_ar,major_ + CH_ar,minor_), 6.92–6.87 (m, 2H, CH_ar,major_ + CH_ar,minor_), 6.82–6.77 (m, 2H, CH_ar,major_ + CH_ar,minor_), 6.24 (bs, 2H, NH_major_ + N*H*_minor_), 4.78 (ddd seen as broad d, ^3^ *J*_HH_ = 12.1 Hz, 1H, CHN_major_), 4.19 (m, 1H, CH*N*_minor_), 4.14–4.05 (m, 8H, 2 × CH_2_O_major_ + 2 × CH_2_ *O*_minor_), 2.89 (ddd seen as dt, ^3^ *J*_HH_ = 12.7 Hz, ^3^ *J*_HH_ = 3.9 Hz, 1H, C*H*_minor_), 2.49 (m, 1H, CH_major_), 2.43 (m, 1H, CH_2,major_), 2.35 (m, 1H, CH_2,minor_), 2.25 (m, 1H, CH_2,major_), 2.20–2.11 (m, 2H, CH_2,major_ + CH_2,minor_), 2.09–1.96 (m, 4H, CH_2 major_ + CH_2,minor_), 1.93–1.78 (m, 5H, 2 × CH_major_ + C*H*_minor_ + CH_2,minor_), 1.76–1.66 (m, 3H, CH_major_ + 2 × C*H*_minor_), 1.61–1.47 (m, 3H, 1H from CH_2,major_ + CH_2,minor_), 1.41–1.29 (m, 2H, CH_2,major_), 1.27–1.23 (m, 6H, CH_3,major_ + CH_3,minor_), 1.20–1.15 (m, 6H, CH_3,major_ + CH_3,minor_). ^13^C­{1H} NMR (100 MHz, CDCl_3_): δ 171.9 (C=*O*_minor_), 171.8 (C=O_major_), 155.3 (C=O_major_), 155.1 (C=*O*_minor_), 147.5 (C_quat_ *N*_minor_), 146.7 (C_quat_ *N*_minor_), 129.5 (C_quat,minor_), 128.1 (C_quat,major_), 127.1 (CH_ar,major_), 125.9 (CH_ar,minor_), 125.8 (CH_ar,major_), 125.7 (CH_ar,minor_), 121.8 (CH_ar,minor_), 121.7 (CH_ar,major_), 114.5 (CH_ar,major_), 114.5 (CH_ar,minor_), 61.9 (CH_2_ *O*_minor_), 61.9 (CH_2_O_major_), 59.8 (CH_2_ *O*_minor_), 59.8 (CH_2_O_major_), 59.2 (CH*N*_minor_), 59.0 (CHN_major_), 39.2 (CH_major_), 38.1 (C*H*_minor_), 35.0 (CH_2,minor_), 33.0 (CH_2,major_), 31.9 (CH_2,major_), 30.9 (CH_2,minor_), 24.2 (CH_major_), 23.9 (CH_major_), 23.7 (C*H*_minor_), 23.5 (C*H*_minor_), 22.9 (CH_2,major_), 21.4 (CH_major_), 20.9 (C*H*_minor_), 20.1 (CH_2,minor_), 18.2 (CH_2,minor_), 17.5 (CH_2,major_), 14.5 (CH_3,minor_), 14.5 (CH_3,major_), 14.2 (CH_3,major_ + CH_3,minor_). HRMS (ESI) *m*/*z*: [M + H]^+^ Calcd for C_21_H_29_N_2_O_4_ 373.2127; Found 373.2104.
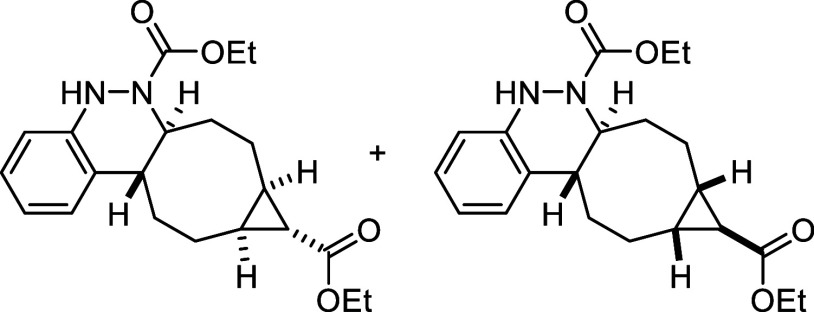


#### Gram-Scale Synthesis of **3a**

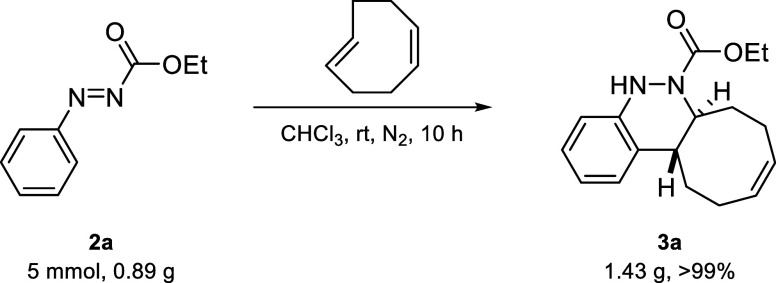



*cis*,*trans*-Cycloocta-1,5-diene (703 mg, 6.5 mmol, 1.3 equiv) was added to a stirred solution of aryldiazene carboxylate **2a** (0.89 g, 5 mmol) in CHCl_3_ (20 mL) under nitrogen atmosphere. The reaction mixture was stirred at room temperature for 10 h. The crude product was purified by flash-column chromatography (SiO_2_, hexanes/AcOEt 93:7) to afford 1.43 g (>99%) of cinnoline **3a** as a brown solid.

#### Synthetic Transformations of Cinnoline Derivatives **3**

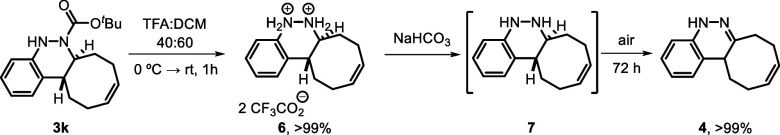



##### (6aS*,12aR*,*Z*)-5,6,6a,7,8,11,12,12a-Octahydrocycloocta­[*c*]­cinnoline bis­(trifluoroacetate) (**6**)

To a 0 °C stirred solution of *N*-Boc-protected cinnoline **3k** (63 mg, 0.2 mmol) in CH_2_Cl_2_ (1.5 mL), trifluoroacetic acid (1 mL) was added dropwise, and the reaction was stirred at room temperature for 1 h. Toluene was added to the reaction mixture, and solvents were eliminated under vacuum, affording 88 mg (>99%) of **6** as a white solid. Mp: 148–149 °C. FTIR (neat) *v*_max_ (cm^–1^) 3021, 2989, 2957, 1672. ^1^H NMR (400 MHz, CDCl_3_): δ 7.09 (dd, ^3^ *J*_HH_ = 7.4 Hz, ^4^ *J*_HH_ = 1.6 Hz, 1H, CH_ar_), 7.05–6.96 (m, 2H, 2 × CH_ar_), 6.67 (dd, ^3^ *J*_HH_ = 7.7, Hz, ^4^ *J*_HH_ = 1.6, Hz, 1H, CH_ar_), 5.80–5.71 (m, 2H, 2 × =CH), 3.63 (m, 1H, CHN), 3.09 (m, 1H, CH), 2.38 (m, 1H, CH_2_), 2.34–2.26 (m, 3H, 2 × CH_2_ + 1H from CH_2_), 2.04 (m, 1H, CH_2_), 1.85 (m, 1H, CH_2_), 1.69 (m, 1H, CH_2_), 1.52 (m, 1H, CH_2_). ^13^C­{1H} NMR (100 MHz, CDCl_3_): δ 140.8 (C_quat_N), 130.1 (=CH), 129.1 (=CH), 129.0 (CH_ar_), 127.7 (C_quat_), 126.8 (CH_ar_), 123.9 (CH_ar_),115.8 (CH_ar_), 61.5 (CHN), 38.8 (CH), 37.7 (CH_2_), 30.1 (CH_2_), 24.8 (CH_2_), 22.3 (CH_2_). ^19^F NMR (376 MHz, CDCl_3_): δ −75.7. HRMS (ESI) *m*/*z*: [M–2­(CF_3_COOH) + H]^+^ Calcd for C_14_H_19_N_2_ 215.1548; Found 215.1548.
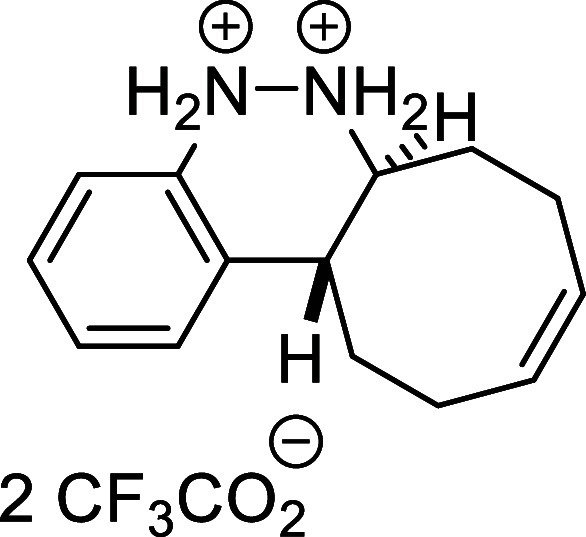


##### (*Z*)-5,7,8,11,12,12a-Hexahydrocycloocta­[*c*]­cinnoline (**4**)


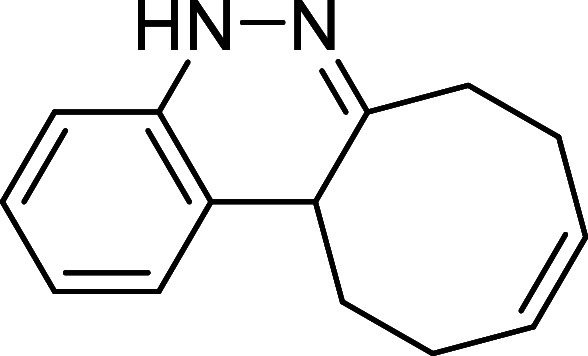
An aqueous solution of NaHCO_3_ (5%, 20 mL) was added to compound **6** (88 mg, 0.2 mmol), and the reaction mixture was stirred at room temperature for 5 min. The crude product was extracted with CH_2_Cl_2_ (30 mL), the organic phase was dried over anhydrous MgSO_4_, filtered, and the solvent was removed under reduced pressure. The unstable intermediate **7** was detected but underwent complete oxidation to the stable compound **4** (42 mg, >99%) after 72 h.
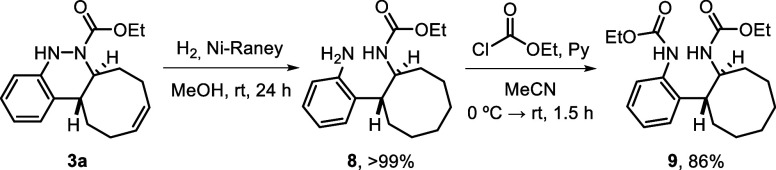


##### Ethyl ((1S*,2R*)-2-(2-Aminophenyl)­cyclooctyl)­carbamate (**8**)

Ni-Raney (50 mg) was added to a solution of cinnoline **3a** (143 mg, 0.5 mmol) in MeOH (15 mL), and the reaction mixture was stirred under a hydrogen atmosphere (3 bar) for 24 h. The mixture was then filtered through a pad of Celite, and the solvent was removed under reduced pressure to afford 145 mg of pure compound **8** (>99%) as a white solid. Mp: 96–98 °C. FTIR (neat) *v*_max_ (cm^–1^) 3363, 3022, 2987, 1693. ^1^H NMR (400 MHz, CDCl_3_): δ 7.07 (d, ^3^ *J*_HH_ = 5.6, Hz, 1H, CH_ar_), 6.98 (m, 1H, CH_ar_), 6.67 (dd seen as t, ^3^ *J*_HH_ = 7.6 Hz, 1H, CH_ar_), 6.65 (dd, ^3^ *J*_HH_ = 7.6 Hz, ^4^ *J*_HH_ = 1.3 Hz, 1H, CH_ar_), 4.97 (bs, 1H, NH), 4.01–3.88 (m, 3H, CH_2_O + CHN), 3.53 (bs, 2H, NH_2_), 2.79 (m, 1H, CH), 2.03–1.48 (m, 12H, 6 × CH_2_), 1.06 (t, ^3^ *J*_HH_ = 7.2, Hz, 3H, CH_3_). ^13^C­{1H} NMR (100 MHz, CDCl_3_): δ 156.0 (C=O), 143.3 (C_quat_N), 130.7 (C_quat_), 126.9 (CH_ar_), 126.6 (CH_ar_), 119.6 (CH_ar_), 116.6 (CH_ar_), 60.2 (CH_2_O), 55.3 (CHN), 41.1 (CH), 31.8 (CH_2_), 30.8 (CH_2_), 27.0 (CH_2_), 26.5 (CH_2_), 25.6 (CH_2_), 24.4 (CH_2_), 14.4 (CH_3_). HRMS (ESI) *m*/*z*: [M + H]^+^ Calcd for C_17_H_27_N_2_O_2_ 291.2073; Found 291.2065.
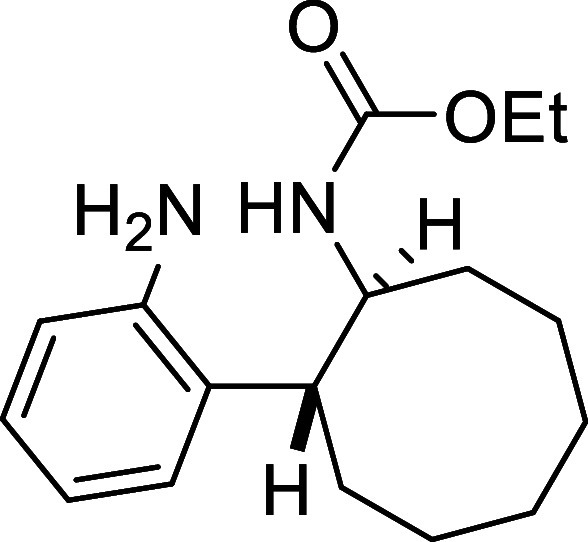


##### Ethyl ((1S*,2R*)-2-(2-((Ethoxycarbonyl)­amino)­phenyl)­cyclooctyl)­carbamate (**9**)

Ethyl chloroformiate (15 μL, 0.26 mmol) was added dropwise to a solution of compound **7** (70 mg, 0.24 mmol) and pyridine (40 μL, 0.50 mmol) in MeCN (2 mL) at 0 °C. The reaction mixture was stirred at room temperature for 1.5 h. The crude product was diluted in CH_2_Cl_2_ (20 mL), washed sequentially with aqueous HCl (2M, 20 mL), saturated aqueous NaHCO_3_ (20 mL), and brine (20 mL). The organic phase was dried over anhydrous MgSO_4_, filtered, and the solvent was removed under reduced pressure. The product was purified by flash-column chromatography (SiO_2_, hexanes/AcOEt 80:20) to yield 75 mg of compound **9** (86%) as a white solid. Mp: 105–106 °C. FTIR (neat) *v*_max_ (cm^–1^) 3271, 3016, 2990, 2924, 1700. ^1^H NMR (400 MHz, CDCl_3_): δ 7.28 (m, 1H, CH_ar_), 7.21–7.11 (m, 3H, 3 × CH_ar_), 6.50 (bs, 1H, NH), 5.86 (bs, 1H, NH), 4.28–4.15 (m, 2H, CH_2_O), 4.03 (bs, 1H, CHN), 3.95–3.88 (m, 2H, CH_2_O), 3.10 (s, 1H, CH), 1.99–1.86 (m, 2H, CH_2_), 1.67–1.52 (m, 10H, 5 × CH_2_), 1.29 (dd seen as t, ^3^ *J*_HH_ = 7.2, Hz, 3H, CH_3_), 1.04 (dd seen as t, ^3^ *J*_HH_ = 7.2, Hz, 3H, CH_3_). ^13^C­{1H} NMR (100 MHz, CDCl_3_): δ 156.2 (C=O), 155.9 (C=O), 142.5 (C_quat_N), 133.7 (C_quat_), 127.5 (CH_ar_), 127.2 (CH_ar_), 126.7 (CH_ar_), 126.5 (CH_ar_), 61.7 (CH_2_O), 60.2 (CH_2_O), 54.7 (CHN), 40.5 (CH), 32.9 (CH_2_), 32.3 (CH_2_), 27.1 (CH_2_), 26.9 (CH_2_), 25.6 (CH_2_), 24.0 (CH_2_), 14.6 (CH_3_), 14.6 (CH_3_). HRMS (ESI) *m*/*z*: [M + H]^+^ Calcd for C_20_H_31_N_2_O_4_ 363.2284; Found 363.2287.
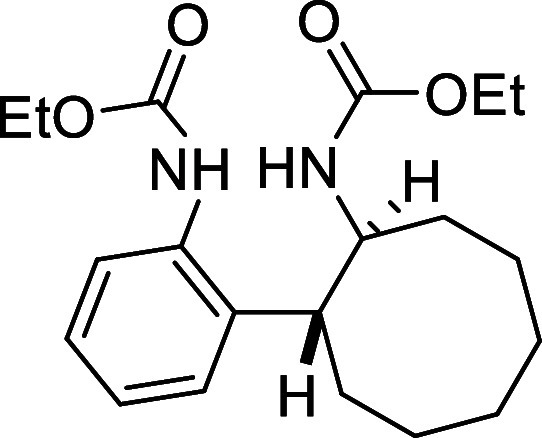


#### General Procedure and Spectral Data for the Synthesis of Fluorescein Derivatives **10–12**

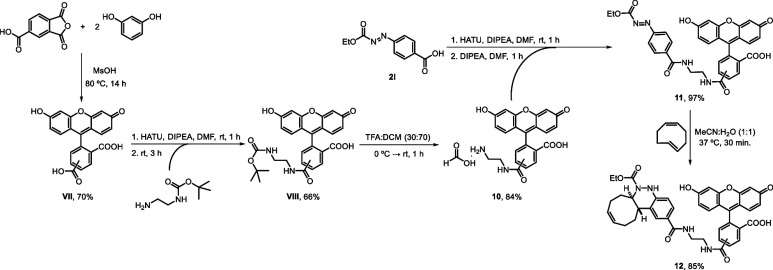



##### 4-(6-Hydroxy-3-oxo-3*H*-xanthen-9-yl)­isophthalic acid and 2-(6-Hydroxy-3-oxo-3*H*-xanthen-9-yl)­terephthalic acid (**VII**)

Following a modified literature procedure,^33^ a solution of 1,2,4-benzenetricarboxylic anhydride (3.84 g, 20 mmol) and resorcinol (4.40 g, 40 mmol) in methanesulfonic acid (20 mL) was heated to 80 °C in a sealed tube, and it was stirred for 14 h. The crude product was precipitated by the addition of H_2_O, then dissolved in aqueous NaOH (2M, 5 mL), and HCl (37%) was added dropwise until pure product **VII** (5.28 g, 70%) precipitated as a brown solid. Spectral data were consistent with those reported in the literature.^33^ 
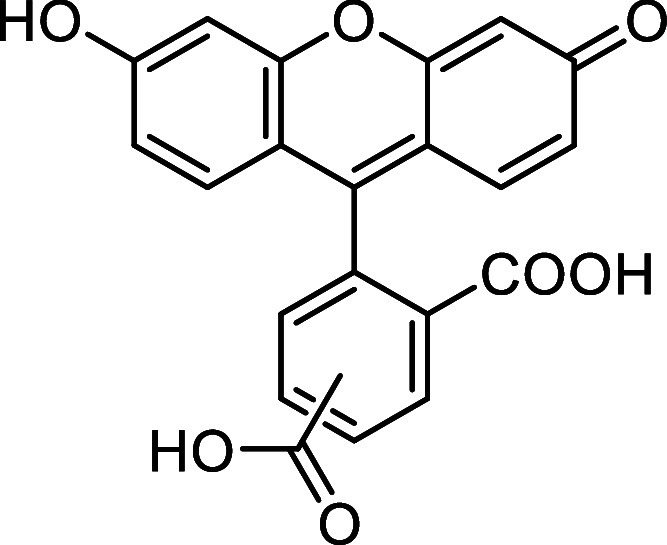


##### 5-((2-((*tert*-Butoxycarbonyl)­amino)­ethyl)­carbamoyl)-2-(6-hydroxy-3-oxo-3*H*-xanthen-9-yl)­benzoic acid and 4-((2-((*tert*-Butoxycarbonyl)­amino)­ethyl)­carbamoyl)-2-(6-hydroxy-3-oxo-3*H*-xanthen-9-yl)­benzoic acid (**VIII**)

DIPEA (0.12 mL, 0.7 mmol) was added to a solution of **VII** (263 mg, 0.7 mmol) and HATU (266 mg, 0.7 mmol) in DMF (15 mL), and the reaction mixture was stirred at room temperature for 1 h. Then, a solution of *N*-Boc-ethylenediamine (112 mg, 0.7 mmol) in DMF (10 mL) and DIPEA (0.12 mL, 0.7 mmol) was added, and the mixture was stirred for an additional 3 h. The crude product was concentrated to dryness under reduced pressure and purified by flash-column chromatography (SiO_2_, CH_2_Cl_2_/MeOH/HCOOH 97:3:0.1), affording 240 mg of **VIII** (66%) as an orange solid. Spectral data were consistent with those reported in the literature.^34^ 
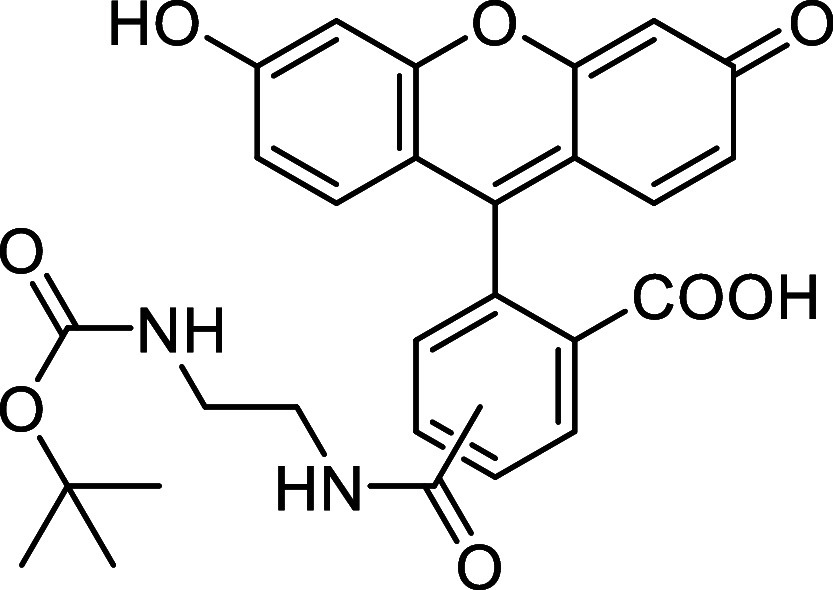


##### 5-((2-Aminoethyl)­carbamoyl)-2-(6-hydroxy-3-oxo-3*H*-xanthen-9-yl)­benzoic acid hydroformate and 4-((2-Aminoethyl)­carbamoyl)-2-(6-hydroxy-3-oxo-3*H*-xanthen-9-yl)­benzoic acid hydroformate (**10**)

TFA (10 mL) was added dropwise to a suspension of compound **VIII** (322 mg, 0.62 mmol) in CH_2_Cl_2_ (15 mL) at 0 °C. After 15 min, the reaction mixture was allowed to warm to room temperature and stirred for 1 h. The crude product was concentrated to dryness under reduced pressure and purified by reverse-phase column chromatography (C18, H_2_O/MeOH, 0.1% HCOOH), affording 242 mg of **10** (84%) as an orange solid. Mp: >400 °C. FTIR (neat) *v*_max_ (cm^–1^) 3380, 3089, 2933, 1744. ^1^H NMR (500 MHz, MeOD): δ 8.55 (d, ^4^ *J*_HH_ = 1.0, Hz, 1H, CH_ar_), 8.31 (dd, ^3^ *J*_HH_ = 8.0 Hz, ^4^ *J*_HH_ = 1.6 Hz, 1H, CH_ar_), 8.23 (dd, ^3^ *J*_HH_ = 8.1 Hz, ^4^ *J*_HH_ = 1.4 Hz, 1H, CH_ar_), 8.15 (dt, ^3^ *J*_HH_ = 8.1 Hz, ^4^ *J*_HH_ = 0.7 Hz, 1H, CH_ar_), 8.14 (m, 1H, HCOOH), 7.24 (d, ^4^ *J*_HH_ = 0.7 Hz, 1H, CH_ar_), 7.38 (d, ^3^ *J*_HH_ = 8.1 Hz, 1H, CH_ar_), 6.79–6.78 (m, 4H, 4 × CH_ar_), 6.70–6.66 (m, 4H, 4 × CH_ar_), 6.63–6.61 (m, 4H, 4 × CH_ar_), 3.79 (t, ^3^ *J*_HH_ = 5.8 Hz, 2H, CH_2_), 3.66 (t, ^3^ *J*_HH_ = 5.8 Hz, 2H, CH_2_), 3.29 (t, ^3^ *J*_HH_ = 5.8 Hz, 2H, CH_2_), 3.17 (t, ^3^ *J*_HH_ = 5.8 Hz, 2H, CH_2_). ^13^C­{1H} NMR (125 MHz, CDCl_3_): δ 170.4 (CO), 169.3 (CO), 169.2 (CO), 164.5 (HCOOH), 163.3 (CO), 162.9 (2 × CO), 162.6 (2 × CO), 161.9 (2 × CO), 161.8 (2 × CO), 156.1 (C_quat_), 154.3 (2 × C_quat_), 154.2 (2 × C_quat_), 154.0 (C_quat_), 141.5 (C_quat_), 137.2 (C_quat_), 135.5 (CH_ar_), 130.7 (CH_ar_), 130.5 (2 × CH_ar_), 130.4 (2 × CH_ar_), 130.2 (2 × CH_ar_), 128.7 (C_quat_), 126.4 (CH_ar_), 125.9 (CH_ar_), 125.4 (CH_ar_), 124.4 (CH_ar_), 119.2 (C_quat_), 116.9 (C_quat_), 114.0 (2 × CH_ar_), 111.0 (C_quat_), 103.7 (2 × CH_ar_), 103.6 (2 × CH_ar_), 40.9 (CH_2_), 40.8 (CH_2_), 38.9 (CH_2_), 38.8 (CH_2_). HRMS (ESI) *m*/*z*: [M – HCOOH + H]^+^ Calcd for C_23_H_19_N_2_O_6_ 419.1238; Found 419.1203.
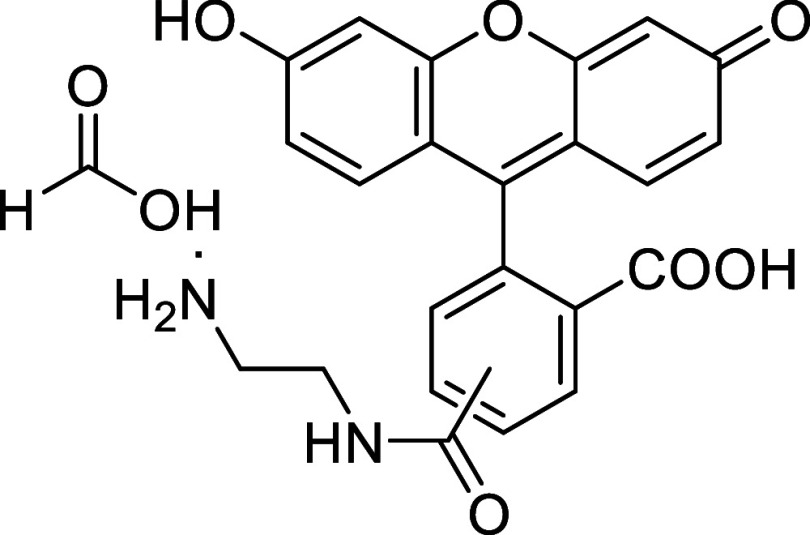


##### 5-((2-(4-((Ethoxycarbonyl)­diazenyl)­benzamido)­ethyl)­carbamoyl)-2-(6-hydroxy-3-oxo-3*H*-xanthen-9-yl)­benzoic acid and 4-((2-(4-((Ethoxycarbonyl)­diazenyl)­benzamido)­ethyl)­carbamoyl)-2-(6-hydroxy-3-oxo-3*H*-xanthen-9-yl)­benzoic acid (**11**)

DIPEA (49 μL, 0.28 mmol) was added to a solution of compound **2i** (62 mg, 0.28 mmol) and HATU (106 mg, 0.28 mmol) in DMF (5 mL), and the reaction mixture was stirred at room temperature for 1 h. Then, a solution of compound **10** (130 mg, 0.28 mmol) in DMF (5 mL) and DIPEA (195 μL, 1.12 mmol) was added, and the reaction mixture was stirred for an additional 1 h. The crude product was concentrated to dryness under vacuum and purified by flash-column chromatography (SiO_2_, CH_2_Cl_2_/EtOH/HCOOH 95:5:0.1), affording 169 mg of **11** (97%) as a red solid. Mp: 343–355 °C (dec.). FTIR (neat) *v*_max_ (cm^–1^) 3275, 2989, 2959, 1744. ^1^H NMR (500 MHz, DMSO-*d*_6_): δ 10.16 (bs, 4H, 4 × OH), 8.98 (t, ^3^ *J*_HH_ = 5.1 Hz, 1H, NH), 8.88 (t, ^3^ *J*_HH_ = 5.1 Hz, 1H, NH), 8.83–8.81 (m, 2H, 2 × NH), 8.47 (s, 1H, CH_ar_), 8.25 (d, ^3^ *J*_HH_ = 8.0 Hz, 1H, CH_ar_), 8.15 (m, 1H, CH_ar_), 8.09–8.07 (m, 3H, 3 × CH_ar_), 7.99–7.96 (m, 4H, 4 × CH_ar_), 7.93–7.92 (m, 2H, 2 × CH_ar_), 7.65 (s, 1H, CH_ar_), 7.38 (d, ^3^ *J*_HH_ = 8.0 Hz, 1H, CH_ar_), 6.69–6.68 (m, 4H, 4 × CH_ar_), 6.60–6.54 (m, 8H, 8 × CH_ar_), 4.49–4.45 (m, 4H, 2 × CH_2_O), 3.53–3.51 (m, 4H, 2 × CH_2_), 3.42–3.40 (m, 4H, H_2_O overlap, 2 × CH_2_), 1.38–1.36 (m, 6H, 2 × CH_3_). ^13^C­{1H} NMR (125 MHz, DMSO-*d*_6_): δ 168.2 (CO), 168.1 (CO), 165.4 (CO), 165.3 (CO), 164.9 (CO), 164.9 (CO), 161.7 (CO), 161.7 (CO), 159.6 (2 × CO), 159.6 (2 × CO), 159.6 (2 × CO), 159.6 (2 × CO), 154.7 (C_quat_), 152.7 (C_quat_), 152.1 (C_quat_), 152.1 (C_quat_), 151.8 (2 × C_quat_), 151.8 (2 × C_quat_), 140.9 (C_quat_), 139.2 (C_quat_), 139.0 (C_quat_), 136.3 (C_quat_), 134.7 (CH_ar_), 129.4 (CH_ar_), 129.3 (2 × CH_ar_), 129.1 (2 × CH_ar_), 128.8 (2 × CH_ar_), 128.7 (2 × CH_ar_), 128.1 (C_quat_), 126.4 (C_quat_), 124.8 (CH_ar_), 124.2 (CH_ar_), 123.3 (CH_ar_), 123.0 (2 × CH_ar_), 123.0 (2 × CH_ar_), 122.3 (CH_ar_), 112.7 (2 × CH_ar_), 112.7 (2 × CH_ar_), 109.1 (C_quat_), 109.0 (C_quat_), 102.3 (2 × CH_ar_), 102.2 (2 × CH_ar_), 64.6 (CH_2_O), 64.6 (CH_2_O), 39.5 (4 × CH_2_, DMSO-*d*_6_ overlap), 14.0 (CH_3_), 14.0 (CH_3_). HRMS (ESI) *m*/*z*: [M + H]^+^ Calcd for C_33_H_27_N_4_O_9_ 623.1771; Found 623.1773.
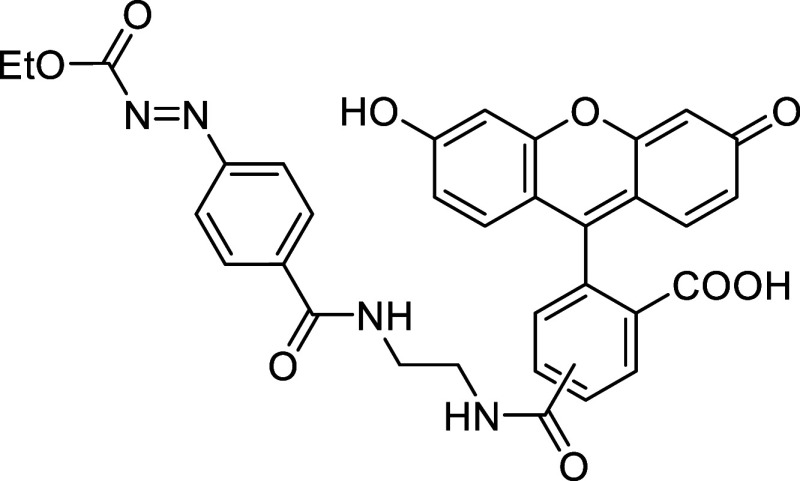


##### 5-((2-((6aR*,12aS*,*Z*)-6-(Ethoxycarbonyl)-5,6,6a,7,8,11,12,12a-octahydrocycloocta­[*c*]­cinnoline-2-carboxamido)­ethyl)­carbamoyl)-2-(6-hydroxy-3-oxo-3*H*-xanthen-9-yl)­benzoic acid and 4-((2-((6aR*,12aS*, *Z*)-6-(Ethoxycarbonyl)-5,6,6a,7,8,11,12,12a-octahydrocycloocta­[*c*]­cinnoline-2-carboxamido)­ethyl)­carbamoyl)-2-(6-hydroxy-3-oxo-3*H*-xanthen-9-yl)­benzoic acid (**12**)

*cis*,*trans*-Cycloocta-1,5-diene (8 mg, 0.075 mmol, 1.5 equiv) was added to a solution of compound **11** (30 mg, 0.05 mmol, 1 equiv) in MeCN/H_2_O (1:1, 1.6 mL), and the reaction mixture was stirred at 37 °C for 30 min. The crude product was concentrated to dryness by vacuum and purified by flash-column chromatography (SiO_2_, CH_2_Cl_2/_EtOH/HCOOH 94:6:0.1), affording 31 mg of **12** (85%) as an orange solid. Mp: 281–292 °C. FTIR (neat) *v*_max_ (cm^–1^) 3342, 3193, 2991, 2965, 1762. ^1^H NMR (500 MHz, DMSO-*d*_6_): δ 10.16 (bs, 4H, 4 × OH), 8.94 (t, ^3^ *J*_HH_ = 5.0 Hz, 1H, NH), 8.82 (t, ^3^ *J*_HH_ = 5.0 Hz, 1H, NH), 8.46 (s, 1H, CH_ar_), 8.43 (t, ^3^ *J*_HH_ = 5.0 Hz, 1H, NH), 8.38 (t, ^3^ *J*_HH_ = 5.0 Hz, 1H, NH), 8.24 (d, ^3^ *J*_HH_ = 8.0 Hz, 1H, CH_ar_), 8.16 (d, ^3^ *J*_HH_ = 8.0 Hz, 1H, CH_ar_), 8.08 (d, ^3^ *J*_HH_ = 8.0 Hz, 1H, CH_ar_), 7.96–7.94 (m, 2H, 2 × NH), 7.80 (s, 1H, CH_ar_), 7.74 (s, 1H, CH_ar_), 7.66 (s, 1H, CH_ar_), 7.56 (d, ^3^ *J*_HH_ = 8.1 Hz, 1H, CH_ar_), 7.47 (d, ^3^ *J*_HH_ = 8.4 Hz, 1H, CH_ar_), 7.37 (d, ^3^ *J*_HH_ = 8.1 Hz, 1H, CH_ar_), 6.96 (d, ^3^ *J*_HH_ = 8.2 Hz, 1H, CH_ar_), 6.92 (d, ^3^ *J*_HH_ = 8.2 Hz, 1H, CH_ar_), 6.69–6.68 (m, 4H, 4 × CH_ar_), 6.63–6.46 (m, 8H, 8 × CH_ar_), 5.78–5.69 (m, 4H, 4× =CH), 4.46–4.45 (m, 2H, 2 × CHN), 4.00–3.92 (m, 4H, 2 × CH_2_O), 3.47–3.42 (m, 8H, H_2_O overlap, 4 × CH_2_N), 2.61–2.56 (m, 2H, 2 × CH), 2.44–2.37 (m, 4H, 2 × CH_2_), 2.31–2.26 (m, 6H, 3 × CH_2_), 2.03–1.98 (m, 2H, CH_2_), 1.66–1.63 (m, 2H, CH_2_), 1.41–1.33 (m, 2H, CH_2_), 1.08–1.05 (m, 6H, 2 × CH_3_). ^13^C­{1H} NMR (125 MHz, DMSO-*d*_6_): δ 168.2 (CO), 168.1 (CO), 166.5 (CO), 166.4 (CO), 165.0 (CO), 164.9 (CO), 159.6 (2 × CO), 159.6 (2 × CO), 159.6 (2 × CO), 159.6 (2 × CO), 156.0 (CO), 156.0 (CO), 154.7 (C_quat_), 152.7 (C_quat_), 151.8 (2 × C_quat_), 151.8 (2 × C_quat_), 151.1 (C_quat_), 151.1 (C_quat_), 140.8 (C_quat_), 136.4 (C_quat_), 134.7 (CH_ar_), 130.0 (=CH), 130.0 (=CH), 129.8 (=CH), 129.8 (=CH), 129.4 (CH_ar_), 129.2 (2 × CH_ar_), 129.1 (2 × CH_ar_), 128.1 (C_quat_), 127.9 (C_quat_), 127.9 (C_quat_), 126.8 (C_quat_), 126.6 (C_quat_), 126.4 (C_quat_), 125.2 (CH_ar_), 125.2 (CH_ar_), 125.1 (CH_ar_), 125.0 (CH_ar_), 124.8 (CH_ar_), 124.2 (CH_ar_), 123.4 (CH_ar_), 122.4 (CH_ar_), 114.0 (CH_ar_), 114.0 (CH_ar_), 112.7 (2 × CH_ar_), 112.7 (2 × CH_ar_), 109.1 (C_quat_), 109.1 (C_quat_), 102.3 (2 × CH_ar_), 102.2 (2 × CH_ar_), 61.5 (CH_2_O), 61.5 (CH_2_O), 59.5 (CHN), 59.5 (CHN), 38.9 (CH_2_N), 38.9 (CH_2_N), 38.8 (CH_2_N), 38.8 (CH_2_N), 37.9 (CH), 37.9 (CH), 32.6 (CH_2_), 32.6 (CH_2_), 28.9 (CH_2_), 28.9 (CH_2_), 24.2 (CH_2_), 24.2 (CH_2_), 23.0 (CH_2_), 23.0 (CH_2_), 14.4 (CH_3_), 14.4 (CH_3_). HRMS (ESI) *m*/*z*: [M + H]^+^ Calcd for C_41_H_39_N_4_O_9_ 731.2709; Found 731.2712.
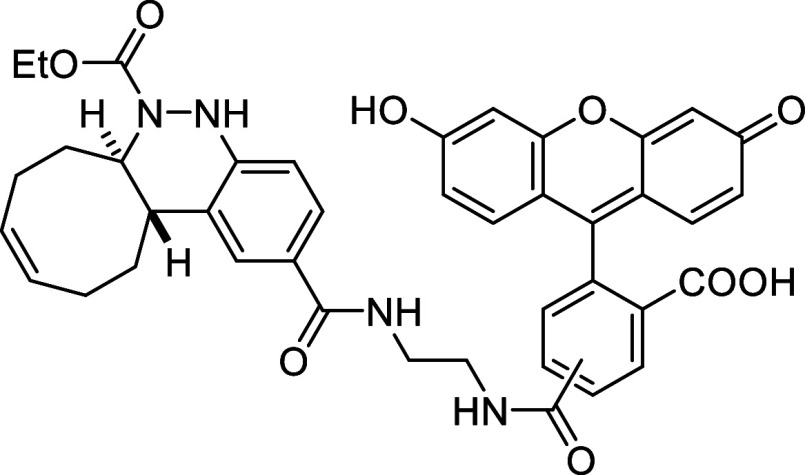


#### General Procedures and Spectral Data for the Synthesis of Coumarin Derivatives **14** and **15**

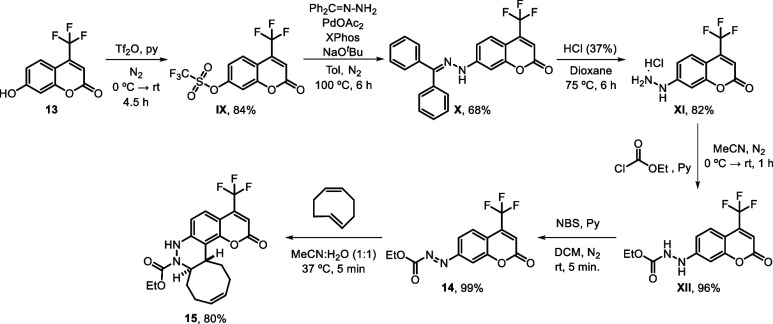



##### 2-Oxo-4-(trifluoromethyl)-2*H*-chromen-7-yl-trifluoromethanesulfonate (**IX**)

Following a modified literature procedure,^35^ trifluoromethanesulfonic anhydride (0.4 mL, 2.4 mmol) was added dropwise to a solution of 7-hydroxy-4-(trifluoromethyl)­coumarin **13** (0.51 g, 2.2 mmol) in pyridine (5 mL) at 0 °C, and the reaction mixture was stirred at room temperature for 4.5 h. The crude product was dissolved in AcOEt (30 mL) and washed with aqueous HCl (5%, 5 × 40 mL) and brine (40 mL). The organic phase was dried over anhydrous MgSO_4_, filtered, and the solvent was removed under vacuum to obtain 668 mg of pure compound **IX** (84%) as a white solid. Spectral data were consistent with those previously reported in the literature.^35^ 
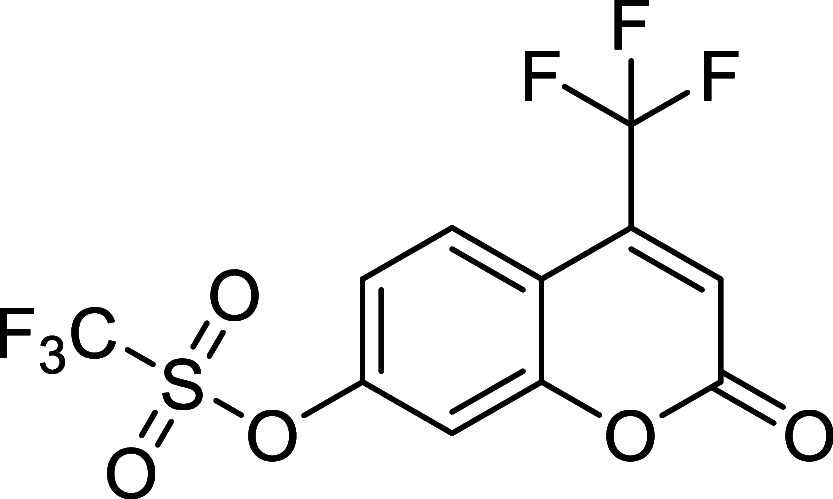


##### 7-(2-(Diphenylmethylene)­hydrazineyl)-4-(trifluoromethyl)-2*H*-chromen-2-one (**X**)

Benzophenone hydrazone (331 mg, 1.62 mmol), XPhos (54 mg, 0.11 mmol), and Pd­(OAc)_2_ (16 mg, 0.07 mmol) were stirred in dry toluene (15 mL) at 100 °C for 5 min. Then, compound **IX** (534 mg, 1.47 mmol) and NaO^*t*^Bu (202 mg, 2.06 mmol) were added, and the reaction mixture was stirred for 6 h at 100 °C. The crude product was purified by flash-column chromatography (SiO_2_, pentane/CH_2_Cl_2_ 50:50), affording 410 mg of **X** (68%) as a yellow solid. Mp: 222–224 °C. FTIR (neat) *v*_max_ (cm^–1^) 3096, 2973, 1720. ^1^H NMR (500 MHz, CDCl_3_): δ 7.82 (bs, 1H, NH), 7.62–7.54 (m, 5H, 5 × CH_ar_), 7.51 (d, ^3^ *J*_HH_ = 8.3 Hz, 1H, CH_ar_), 7.35–7.32 (m, 5H, 5 × CH_ar_), 7.19 (s, 1H, CH_ar_), 6.88 (d, ^3^ *J*_HH_ = 8.3 Hz, 1H, CH_ar_) 6.49 (s, 1H, CH_ar_). ^13^C­{1H} NMR (125 MHz, CDCl_3_): δ 159.9 (C=O), 156.5 (C = N), 148.4 (C_quat_), 148.3 (C_quat_), 141.6 (q, ^2^ *J*_CF_ = 32.5 Hz, C_quat_), 137.3 (C_quat_), 131.8 (C_quat_), 129.9 (2 × CH_ar_), 129.8 (CH_ar_), 129.1 (CH_ar_), 128.8 (2 × CH_ar_), 128.4 (2 × CH_ar_), 127.0 (2 × CH_ar_), 126.2 (q, ^4^ *J*_CF_ = 2.7 Hz, CH_ar_), 121.7 (q, ^1^ *J*_CF_ = 275.6 Hz, CF_3_), 110.7 (CH_ar_), 110.4 (q, ^3^ *J*_CF_ = 5.9 Hz, CH_ar_), 106.2 (C_quat_), 100.1 (CH_ar_). ^19^F NMR (282 MHz, CDCl_3_): δ – 64.6. HRMS (ESI) *m*/*z*: [M + H]^+^ Calcd for C_23_H_16_F_3_N_2_O_2_ 409.1158; Found 409.1147.
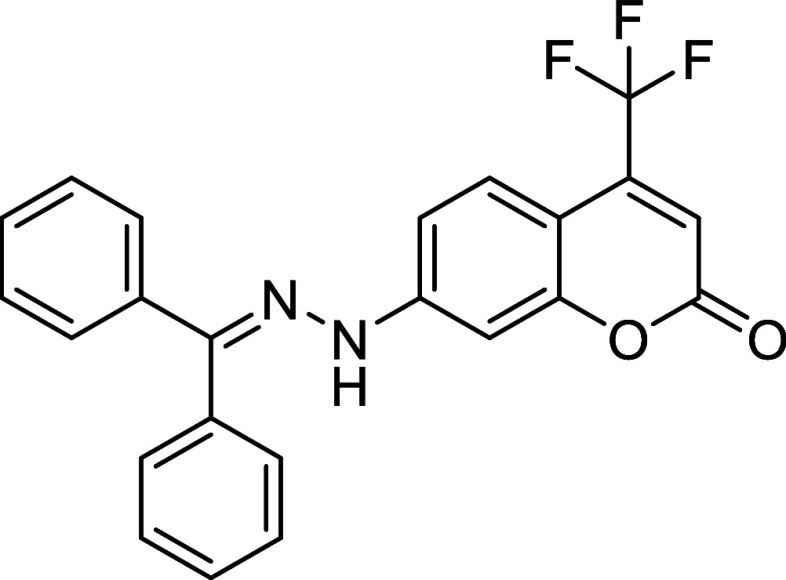


##### 7-Hydrazineyl-4-(trifluoromethyl)-2*H*-chromen-2-one hydrochloride (**XI**)

Aqueous HCl (5%, 20 mL) was added to a solution of **X** (457 mg, 1.12 mmol) in dioxane (36 mL), and the reaction mixture was heated to 75 °C for 6 h. The crude product was concentrated to dryness under vacuum and precipitated from Et_2_O (40 mL), affording 257 mg of **XI** (82%) as a yellow solid. Mp: 237–239 °C. FTIR (neat) *v*_max_ (cm^–1^) 3527, 2920, 2874, 1719. ^1^H NMR (500 MHz, MeOD): δ 7.77 (m, 1H, CH_ar_), 7.06 (dd, ^3^ *J*_HH_ = 8.9 Hz, 2.4 Hz, 1H, CH_ar_), 7.00 (d, ^3^ *J*_HH_ = 2.4 Hz, 1H, CH_ar_), 6.80 (s, 1H, CH_ar_). ^13^C­{1H} NMR (125 MHz, MeOD): δ 160.6 (C=O), 157.4 (C_quat_N), 150.9 (C_quat_O), 142.2 (q, ^2^ *J*_CF_ = 32.6 Hz, C_quat_), 127.6 (q, ^4^ *J*_CF_ = 2.3 Hz, CH_ar_), 123.2 (q, ^1^ *J*_CF_ = 274.6 Hz, CF_3_), 114.1 (q, ^3^ *J*_CF_ = 5.8 Hz, CH_ar_), 112.5 (CH_ar_), 108.7 (C_quat_), 101.7 (CH_ar_). ^19^F NMR (565 MHz, MeOD): δ −66.1. HRMS (ESI) *m*/*z*: [M – HCl + H]^+^ Calcd for C_10_H_8_F_3_N_2_O_2_ 245.0532; Found 245.0527.
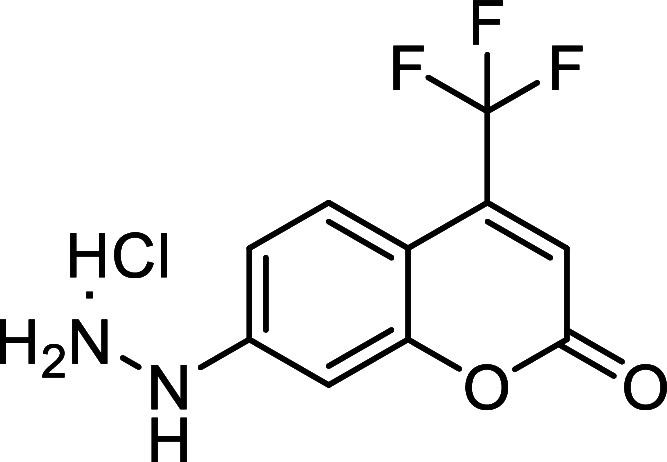


##### Ethyl 2-(2-Oxo-4-(trifluoromethyl)-*2H*-chromen-7-yl)­hydrazine-1-carboxylate (**XII**)

Following the general procedure for hydrazine functionalization, ethyl chloroformiate (84 μL, 0.88 mmol) was added to a suspension of compound **XI** (225 mg, 0.8 mmol) and pyridine (136 μL, 1.68 mmol) in MeCN at 0 °C, and the reaction mixture was stirred at room temperature for 1 h. The mixture was then acidified with aqueous HCl (5 M) to pH = 4. The crude product was extracted with CH_2_Cl_2_ (4 × 20 mL), and the combined organic layers were washed with saturated NaHCO_3_ (25 mL) and brine (25 mL), dried over anhydrous Na_2_SO_4_, filtered, and concentrated to dryness under vacuum, affording 243 mg of **XII** (96%) as a yellow solid. Mp: 222–224 °C. FTIR (neat) *v*_max_ (cm^–1^) 3292, 3115, 2984, 1708. ^1^H NMR (500 MHz, DMSO-*d*_6_): δ 9.37 (bs, 1H, NH), 8.83 (bs, 1H, NH), 7.51 (d, ^3^ *J*_HH_ = 8.8 Hz, 1H, CH_ar_), 6.78 (d, ^3^ *J*_HH_ = 8.8 Hz, 1H, CH_ar_), 6.62 (s, 1H, CH_ar_), 6.56 (s, 1H, CH_ar_), 4.09 (d, ^3^ *J*_HH_ = 6.8 Hz, 2H, CH_2_O), 1.22 (m, 3H, CH_3_). ^13^C­{1H} NMR (125 MHz, DMSO-*d*_6_): δ 159.1 (C=O), 156.5 (C=O), 156.2 (C_quat_N), 153.9 (C_quat_O), 139.9 (q, ^2^ *J*_CF_ = 31.8 Hz, C_quat_), 125.8 (CH_ar_), 121.8 (q, ^1^ *J*_CF_ = 275.5 Hz, CF_3_), 110.0 (q, ^3^ *J*_CF_ = 5.5 Hz, CH_ar_), 109.7 (CH_ar_), 103.9 (C_quat_), 97.2 (CH_ar_), 60.7 (CH_2_O), 14.5 (CH_3_). ^19^F NMR (565 MHz, DMSO-*d*_6_): δ −63.5. HRMS (ESI) *m*/*z*: [M + H]^+^ Calcd for C_13_H_12_F_3_N_2_O_4_ 317.0744; Found 317.0734.
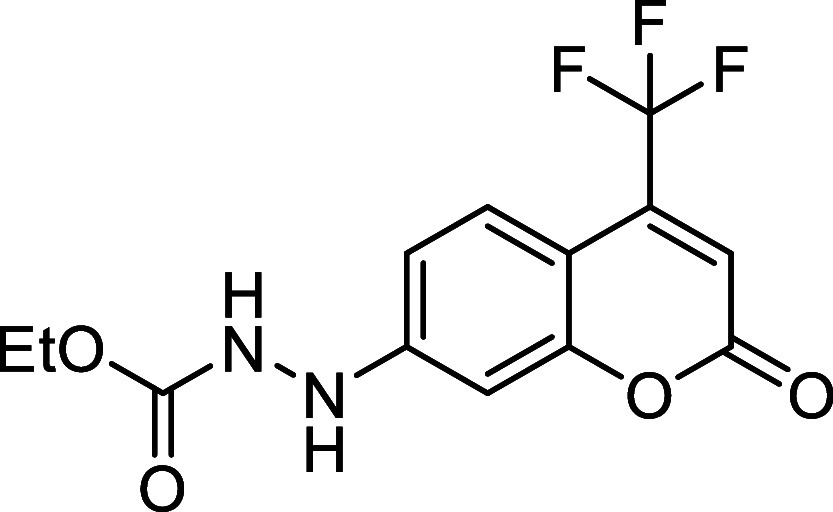


##### Ethyl 2-(2-Oxo-4-(trifluoromethyl)-2*H*-chromen-7-yl)­diazene-1-carboxylate (**14**)

Following the general procedure for hydrazine oxidation, NBS (37 mg, 0.21 mmol) was added to a suspension of compound **XII** (60 mg, 0.19 mmol) and pyridine (43 μL, 0.53 mmol) in CH_2_Cl_2_ (2 mL), and the reaction mixture was stirred at room temperature for 5 min. The crude product was diluted with CH_2_Cl_2_ (15 mL) and washed successively with aqueous HCl (1,5 M, 10 mL), an aqueous solution of Na_2_S_2_O_3_ (1.5%, 10 mL), saturated NaHCO_3_ (10 mL), and brine (10 mL). The organic phase was dried over anhydrous Na_2_SO_4_, filtered, and concentrated under vacuum. The crude product was purified by flash-column chromatography (SiO_2_, pentane/AcOEt 98:2), affording 59 mg of **14** (99%) as a red solid. Mp: 92–93 °C. FTIR (neat) *v*_max_ (cm^–1^) 3094, 2970, 1741. ^1^H NMR (500 MHz, CDCl_3_) δ 7.87 (m, 3H, 3 × CH_ar_), 6.89 (s, 1H, CH_ar_), 4.52 (q, ^3^ *J*_HH_ = 7.2 Hz, 2H, CH_2_O), 1.45 (t, ^3^ *J*_HH_ = 7.2 Hz, 3H, CH_3_). ^13^C­{1H} NMR (125 MHz, CDCl_3_): δ 161.6 (C=O), 158.0 (C=O), 154.7 (C_quat_N), 153.7 (C_quat_O), 140.8 (q, ^2^ *J*_CF_ = 33.4 Hz, C_quat_), 126.5 (^4^ *J*_CF_ = 2.4 Hz, CH_ar_), 121.2 (q, ^1^ *J*_CF_ = 275.6 Hz, CF_3_), 119.7 (CH_ar_), 118.1 (^3^ *J*_CF_ = 5.7 Hz, CH_ar_), 117.1 (C_quat_), 112.5 (CH_ar_), 65.0 (CH_2_O), 14.1 (CH_3_). ^19^F NMR (282 MHz, CDCl_3_): δ −64.7. HRMS (ESI) *m*/*z*: [M + H]^+^ Calcd for C_13_H_10_F_3_N_2_O_4_ 315.0587; Found 315.0578.
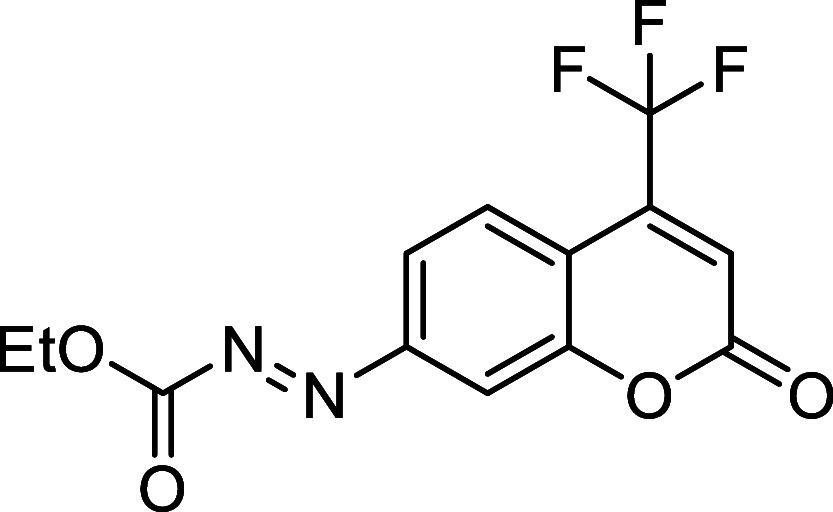


##### Ethyl (8aR*,14aS*,*Z*)-2-Oxo-4-(trifluoromethyl)-2,7,8a,9,10,13,14,14a-octahydro-8*H*-cycloocta­[*c*]­pyrano­[2,3-*f*]­cinnoline-8-carboxylate (**15**)

*cis,trans*-Cycloocta-1,5-diene (11 mg, 0.1 mmol) was added to a solution of **14** (15 mg, 0.05 mmol) in a MeCN/H_2_O mixture (1.6 mL), and the reaction mixture was stirred at 37 °C for 5 min in complete darkness. The crude product was purified by flash-column chromatography (SiO_2_, pentane/CH_2_Cl_2_ 30:70), affording 17 mg of **15** (80%) as a yellow solid. Mp: 145–147 °C. FTIR (neat) *v*_max_ (cm^–1^) 3369, 3215, 2991, 2946, 1740. ^1^H NMR (500 MHz, CDCl_3_): δ 7.41 (d, ^3^ *J*_HH_ = 8.6 Hz, 1H, CH_ar_), 6.81 (bs, 1H, NH), 6.74 (d, ^3^ *J*_HH_ = 8.6 Hz, 1H, CH_ar_), 6.55 (s, 1H, CH_ar_), 5.87 (ddd seen as q, ^3^ *J*_HH_ = 8.8 Hz, 1H, =CH), 5.77 (ddd seen as q, ^3^ *J*_HH_ = 8.8 Hz, 1H, =CH), 4.49 (ddd seen as broad d, ^3^ *J*_HH_ = 11.5 Hz, 1H, CHN), 4.13 (m, 2H, CH_2_O), 3.32 (d, ^3^ *J*_HH_ = 11.5 Hz, 1H, CH), 2.48–2.36 (m, 4H, 2 × CH_2_), 2.31 (m, 1H, CH_2_), 1.97 (m, 1H, CH_2_), 1.81 (m, 1H, CH_2_), 1.37 (m, 1H, CH_2_), 1.22 (dd seen as t, ^3^ *J*_HH_ = 7.1 Hz, 3H, CH_3_). ^13^C­{1H} NMR (125 MHz, CDCl_3_): δ 159.0 (C=O), 155.7 (C=O), 154.1 (C_quat_N), 149.8 (C_quat_O), 142.1 (q, ^2^ *J*_CF_ = 32.4 Hz, C_quat_), 130.6 (=CH), 129.7 (=CH), 123.3 (q, ^4^ *J*_CF_ = 2.1 Hz, CH_ar_), 121.7 (q, ^1^ *J*_CF_ = 175.4 Hz, CF_3_), 115.1 (C_quat_), 111.6 (CH_ar_), 111.2 (q, ^3^ *J*_CF_ = 5.7 Hz, CH_ar_), 107.6 (C_quat_), 62.6 (CH_2_O), 59.5 (CHN), 36.3 (CH), 31.2 (CH_2_), 31.1 (CH_2_), 25.8 (CH_2_), 24.5 (CH_2_), 14.5 (CH_3_). ^19^F NMR (565 MHz, CDCl_3_): δ −64.3. HRMS (ESI) *m*/*z*: [M + H]^+^ Calcd for C_21_H_22_F_3_N_2_O_4_ 423.1532; Found 423.1512.
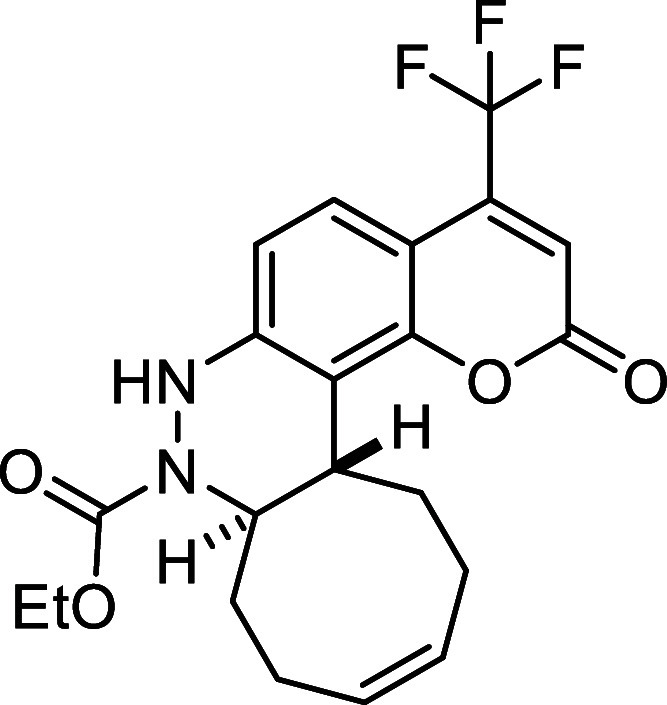


## Supplementary Material



## Data Availability

The data underlying this study are available in the published article and its Supporting Information.

## Notes

Copies of ^1^H, ^13^C, and ^19^F NMR spectra for compounds **1i**, **2i**, **III**, **VI**, **3a**–**o**, **4**, **5a** + **5a′**, **6**, **8**, **9**, **10**, **11**, **12**, **X**, **XI**, **XII, 14**, and **15**, 2D NMR spectra for compounds **11** and **12** (PDF)
