# Supplementary material for: Azo-Povarov Cycloaddition of N‑Carbonyl Aryldiazenes with cis,trans-Cycloocta-1,5-diene as a Fluorogenic Click Reaction for the Synthesis of Cinnoline Derivatives
Source: J Org Chem. 2026 Apr 30;91(19):6646–64. doi: 10.1021/acs.joc.6c00313 (PMC13185131; doi:10.1021/acs.joc.6c00313)
Supplement: Supplementary file 1 [file jo6c00313_si_001.pdf]

Supporting Information

Azo-Povarov Cycloaddition of *N*-Carbonyl Aryldiazenes with *cis,trans*-Cycloocta-1,5-diene as a Fluorogenic Click Reaction for the Synthesis of Cinnoline Derivatives.

Xabier Jiménez-Aberásturi,[a] Javier Vicario,[a] Frank Abendroth,[b] Olalla Vázquez,[b,c]*

Jesús M. de los Santos,[a]*

[a] *Department of Organic Chemistry I, Faculty of Pharmacy and Lascaray Research Center, University of the Basque Country (UPV/EHU), Paseo de la Universidad 7, 01006 Vitoria-Gasteiz, Spain.*

E mail: jesus.delossantos@ehu.eus

[b] *Faculty of Chemistry, and Chemical Biology Division, Marburg University, Hans-Meerwein-Straße 4, 35043 Marburg, Germany*

[c] *Centre for Synthetic Microbiology (SYNMIKRO), Karl-von-Frisch-Straße 14, 35043 Marburg, Germany*

E-mail: olalla.vazquez@staff.uni-marburg.de

Table of contents

NMR spectra of all target compounds.. S2

Absorption spectra of compound **15**.. S47

References.. S47

NMR spectra of all target compounds

^{1}H NMR (400 MHz, $(CD_3)_2CO$) of 4-(2-(ethoxycarbonyl)hydrazineyl)benzoic acid **1i**

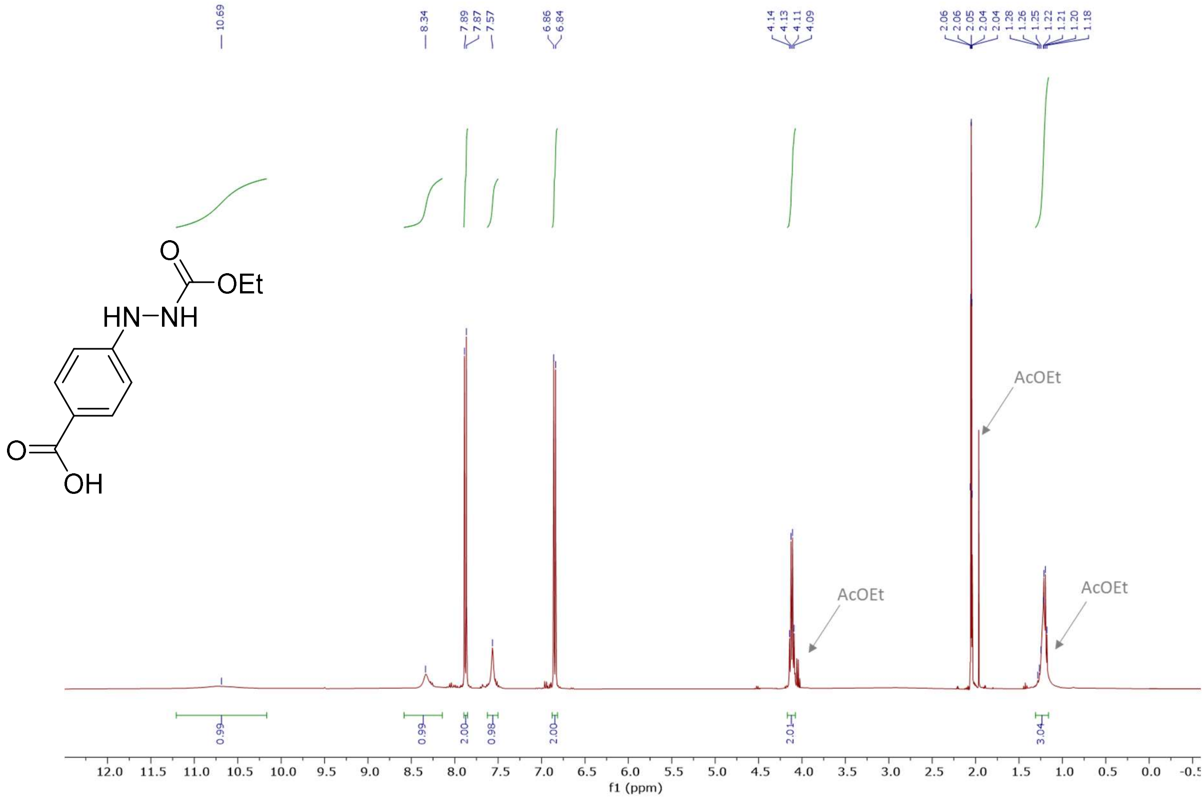

^{13}C{1H} NMR (100 MHz, $(CD_3)_2CO$) of 4-(2-(ethoxycarbonyl)hydrazineyl)benzoic acid **1i**

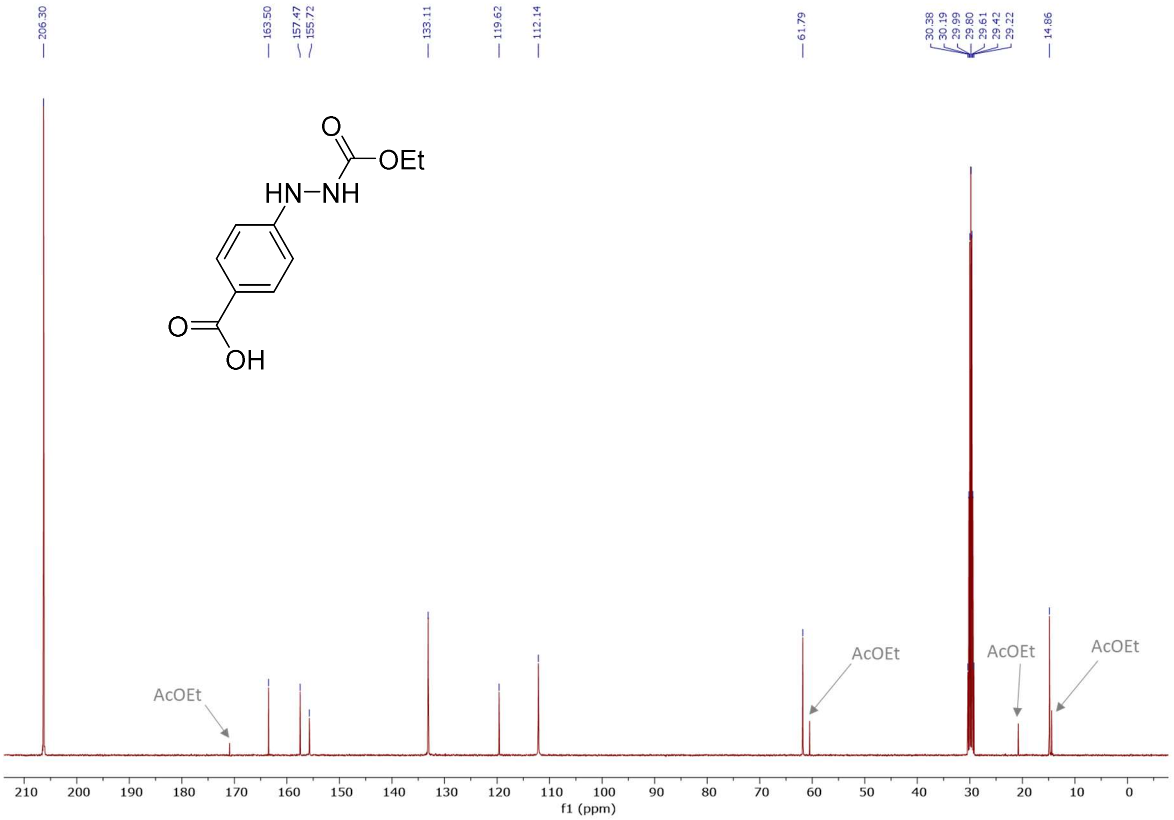

^{1}H NMR (400 MHz, $(CD_3)_2CO$) of aryldiazene carboxylate **2i**

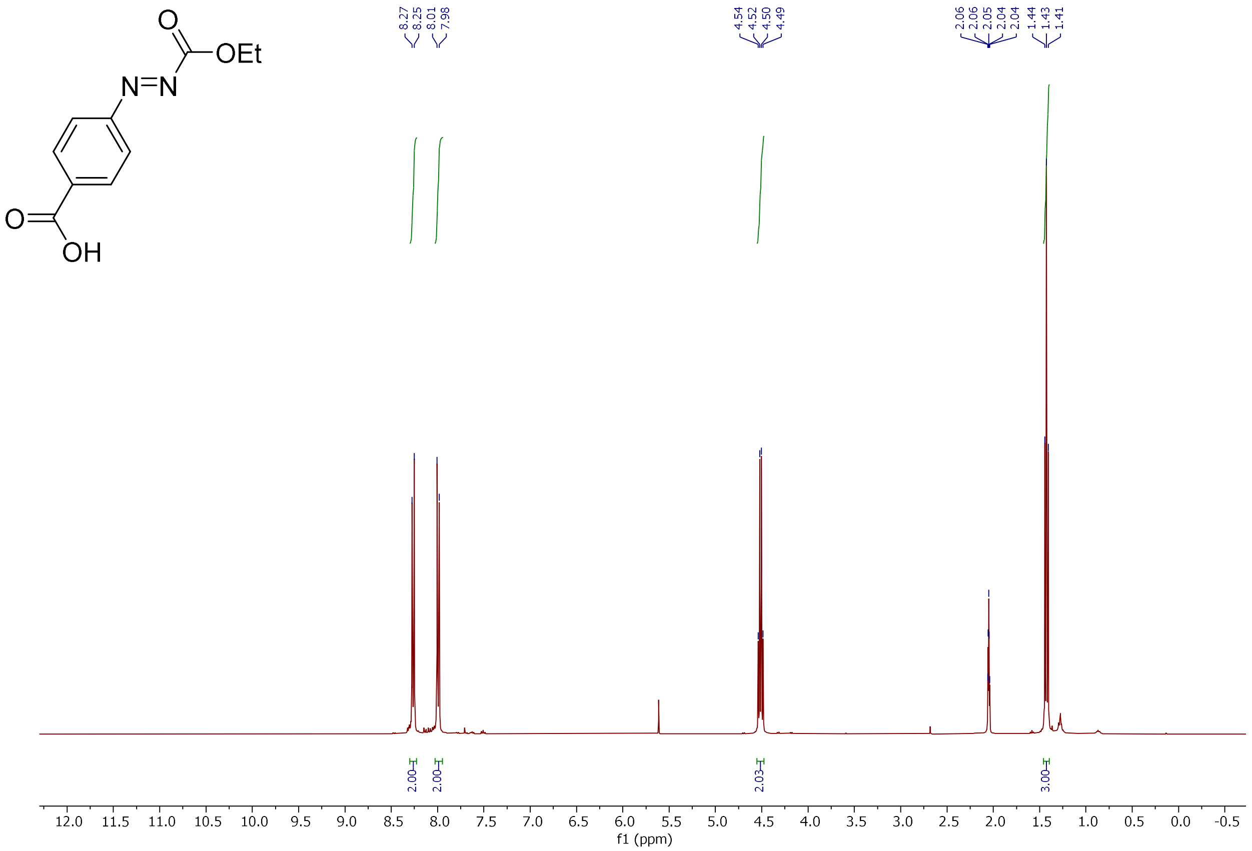

$^{13}C\{1H\}$ NMR (100 MHz, $(CD_3)_2CO$) of aryldiazene carboxylate **2i**

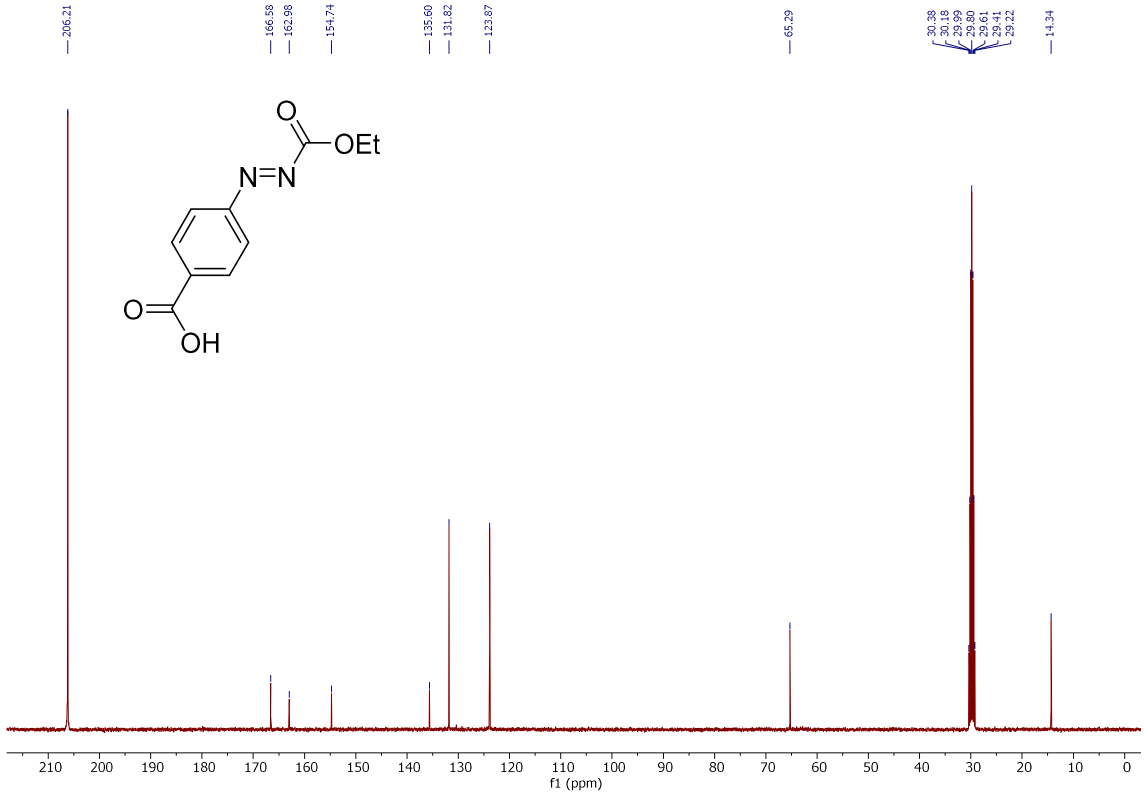

^{1}H NMR (400 MHz, $CDCl_3$) of 8-hydroxycyclooct-4-en-1-yldiphenylphosphine oxide **III**

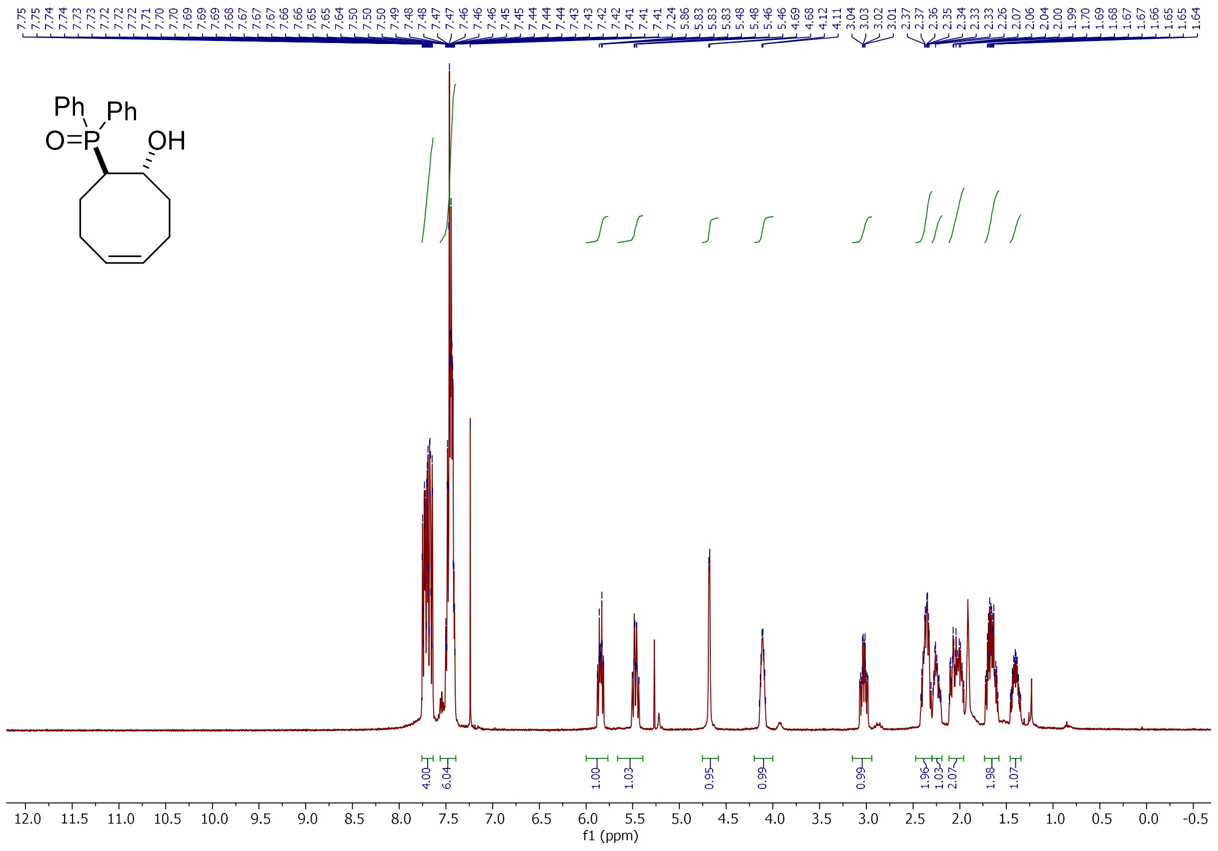

^{13}C{1H} NMR (100 MHz, $CDCl_3$) of 8-hydroxycyclooct-4-en-1-yldiphenylphosphine oxide **III**

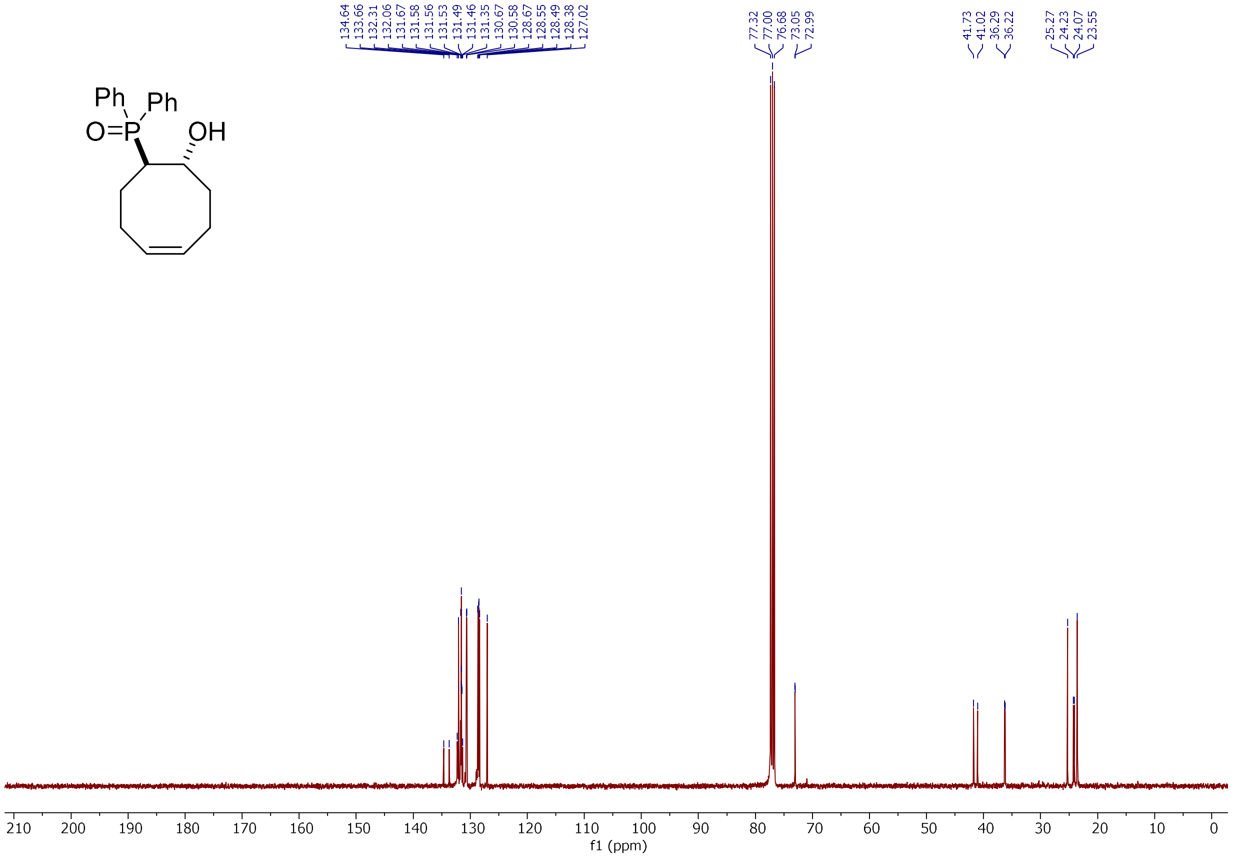

^{31}P NMR (162 MHz, $CDCl_3$) of 8-hydroxycyclooct-4-en-1-yldiphenylphosphine oxide **III**

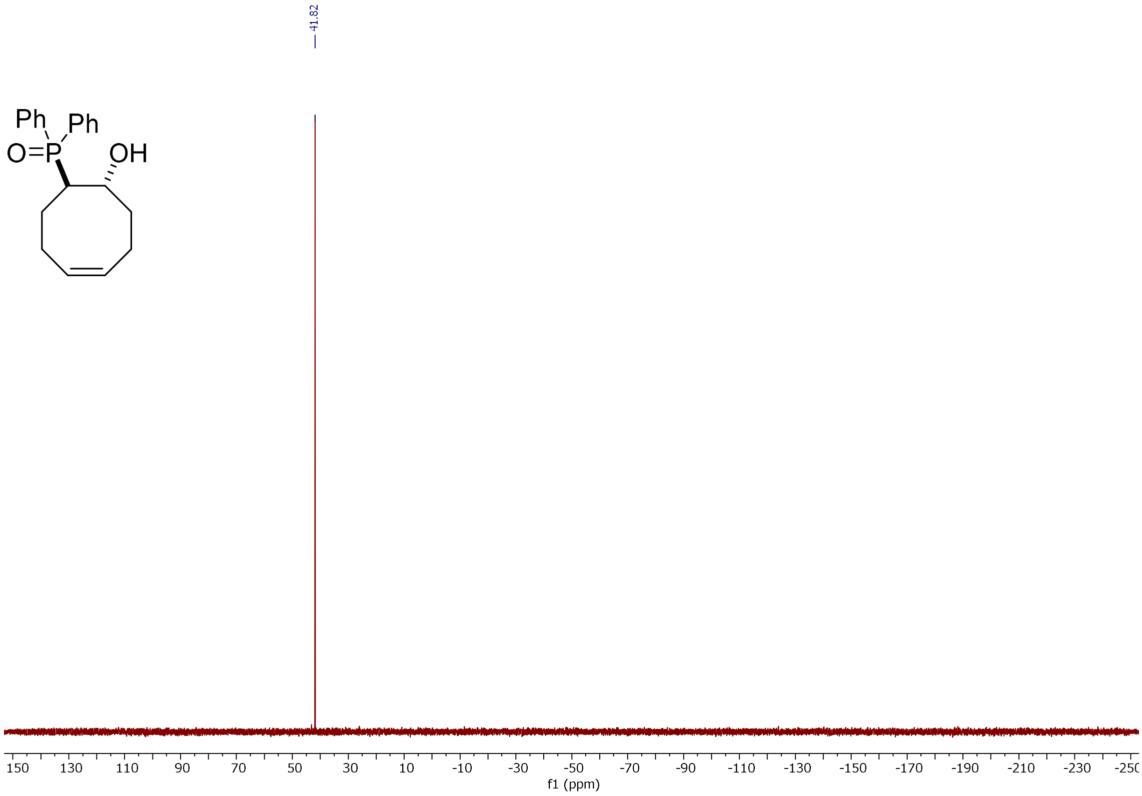

^{1}H NMR (400 MHz, $CDCl_3$) of ethyl (1*R**,8*S**,9*r**,*E*)-bicyclo[6.1.0]non-4-ene-9-carboxylate **VI**

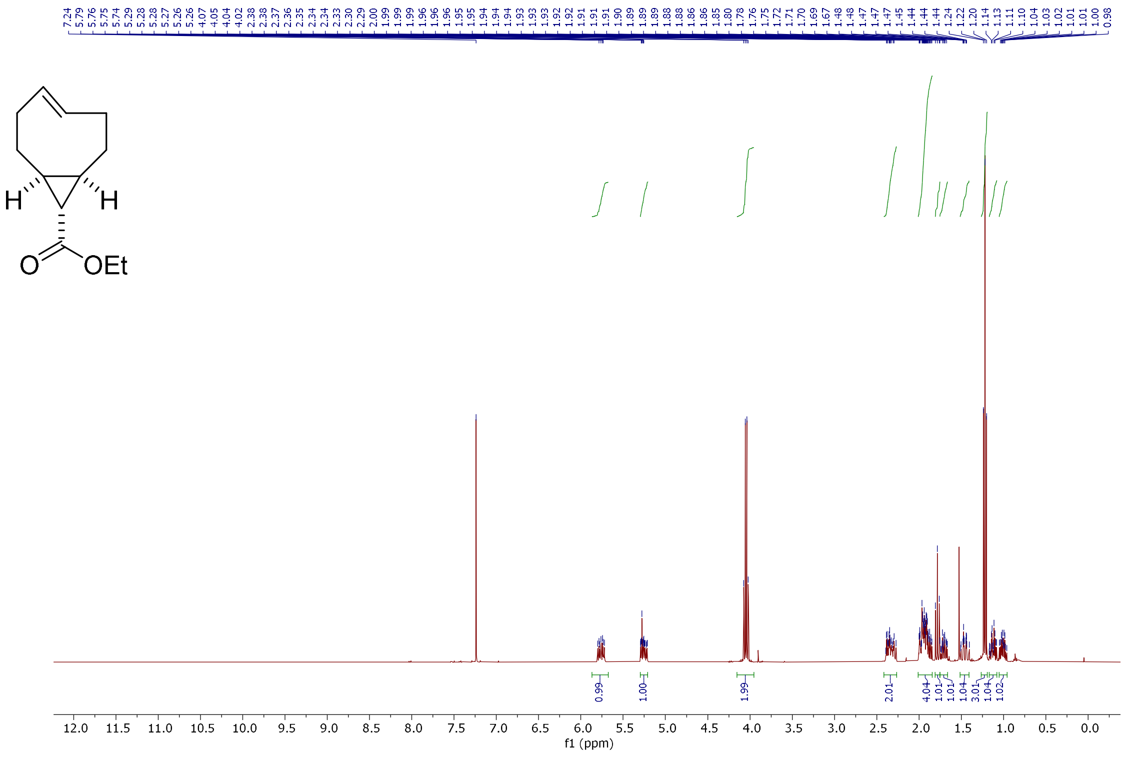

^{13}C{1H} NMR (100 MHz, $(CD_3)_2CO$) of ethyl (1*R**,8*S**,9*r**,*Z*)-bicyclo[6.1.0]non-4-ene-9-carboxylate **VI**

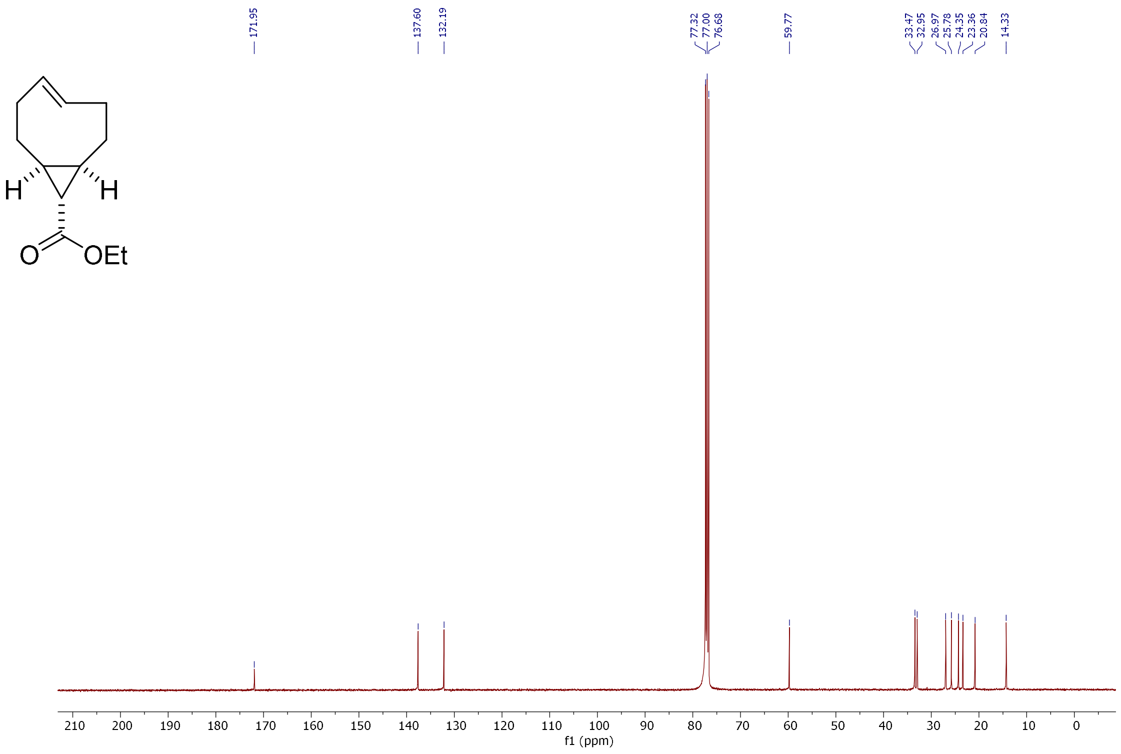

^{1}H NMR (400 MHz, $CDCl_3$) of cinnoline derivative **3a**

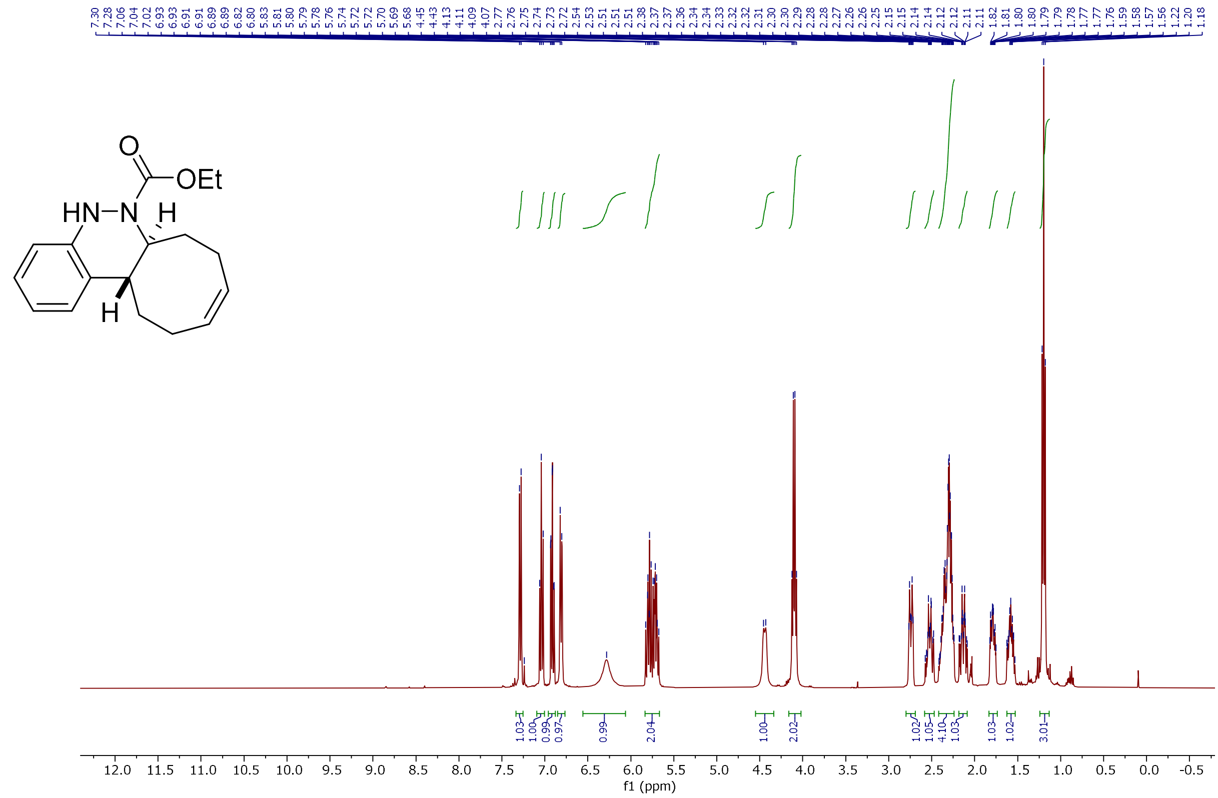

$^{13}C\{1H\}$ NMR (100 MHz, $CDCl_3$) of cinnoline derivative **3a**

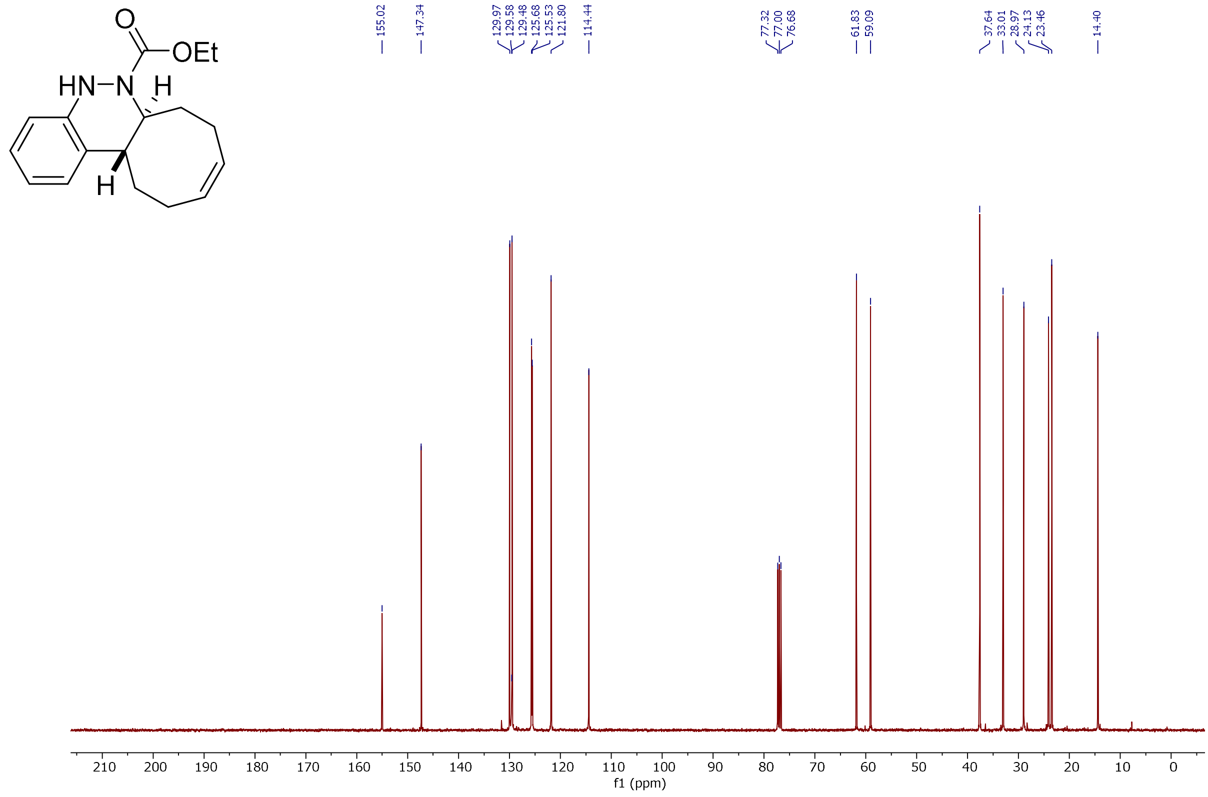

^{1}H NMR (400 MHz, $CDCl_3$) of cinnoline derivative **3b**

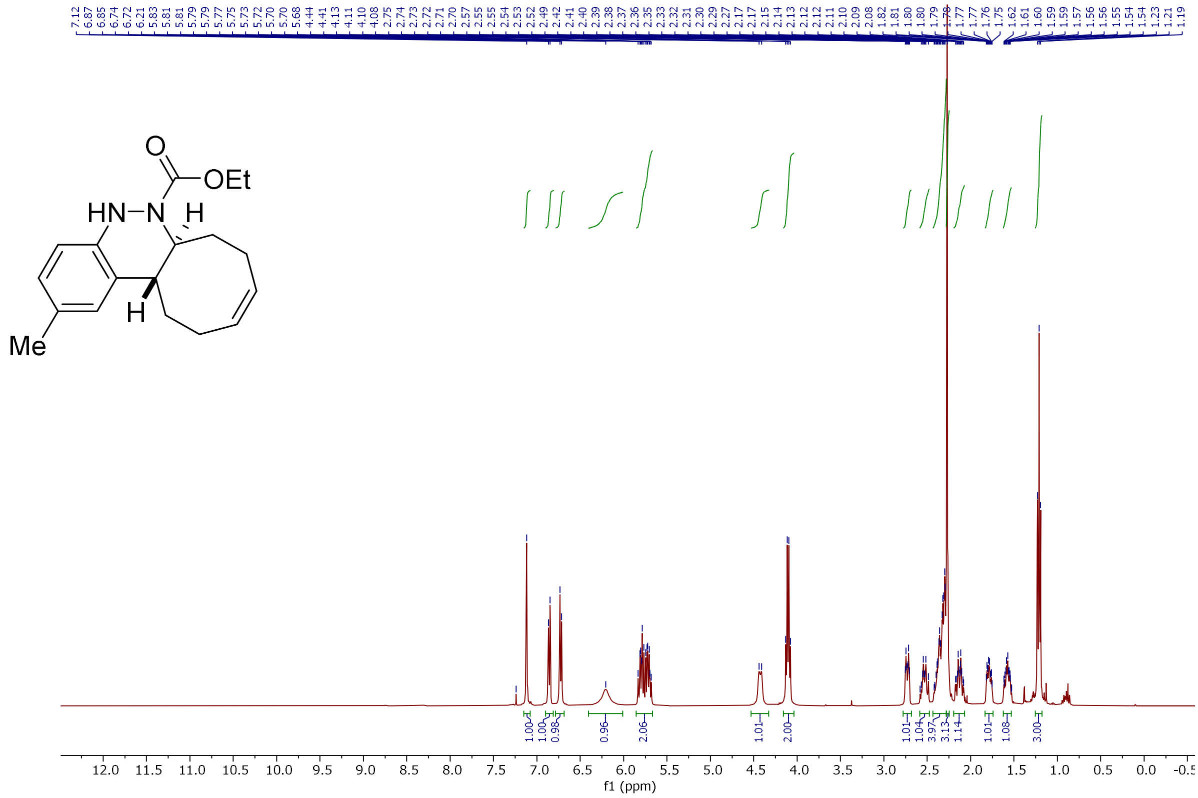

^{13}C{1H} NMR (100 MHz, $CDCl_3$) of cinnoline derivative **3b**

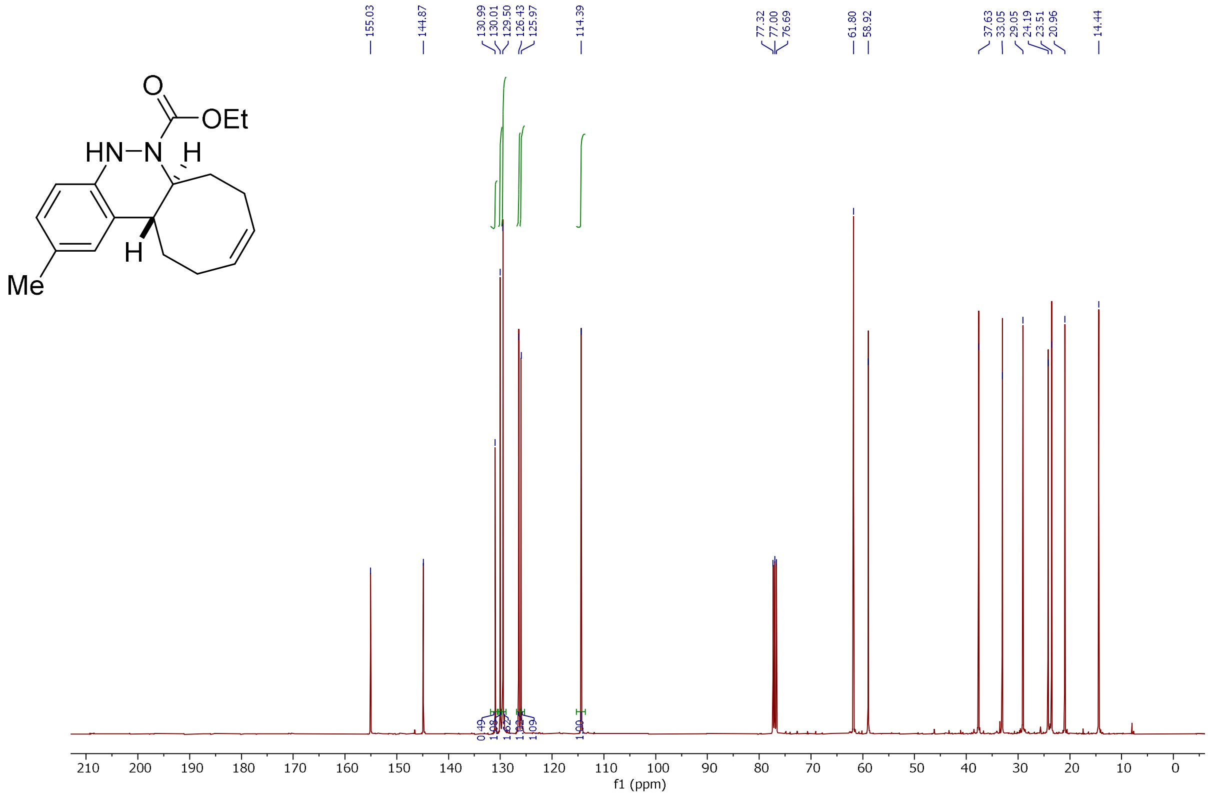

^{1}H NMR (400 MHz, $CDCl_3$) of cinnoline derivative **3c**

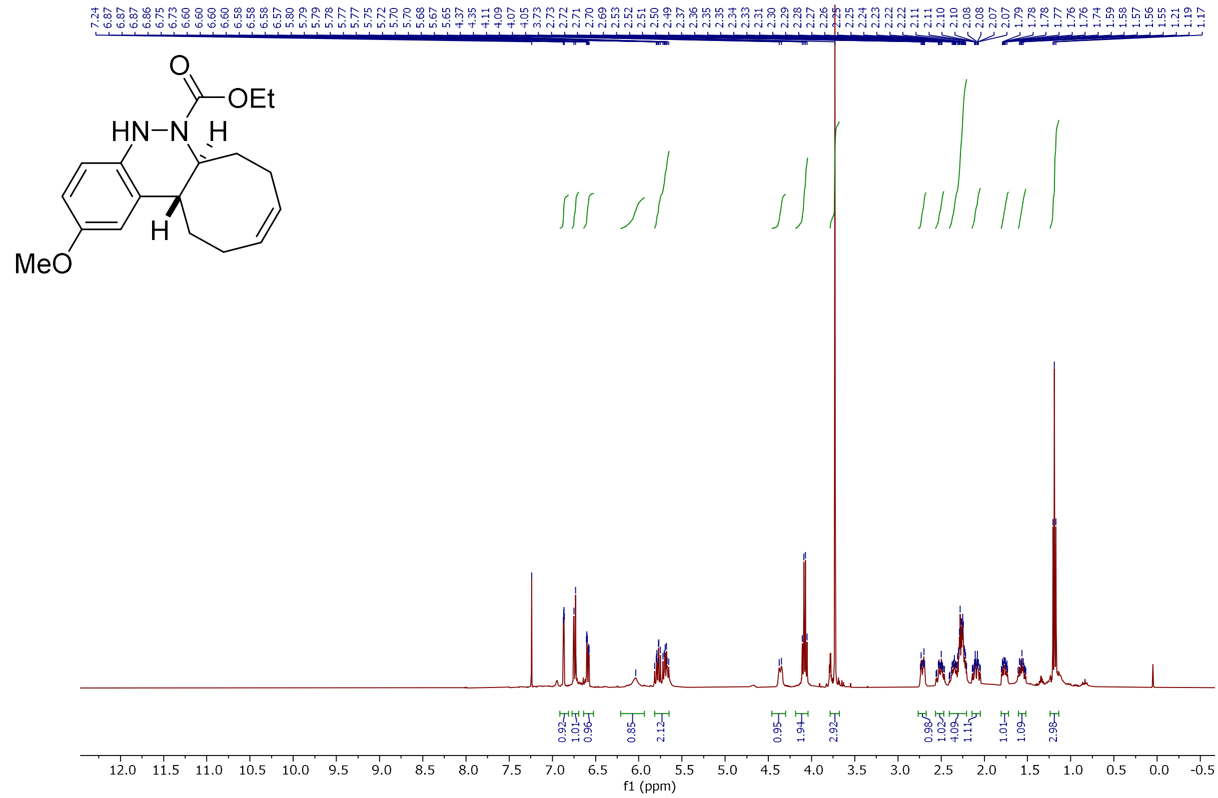

^{13}C{1H} NMR (100 MHz, $CDCl_3$) of cinnoline derivative **3c**

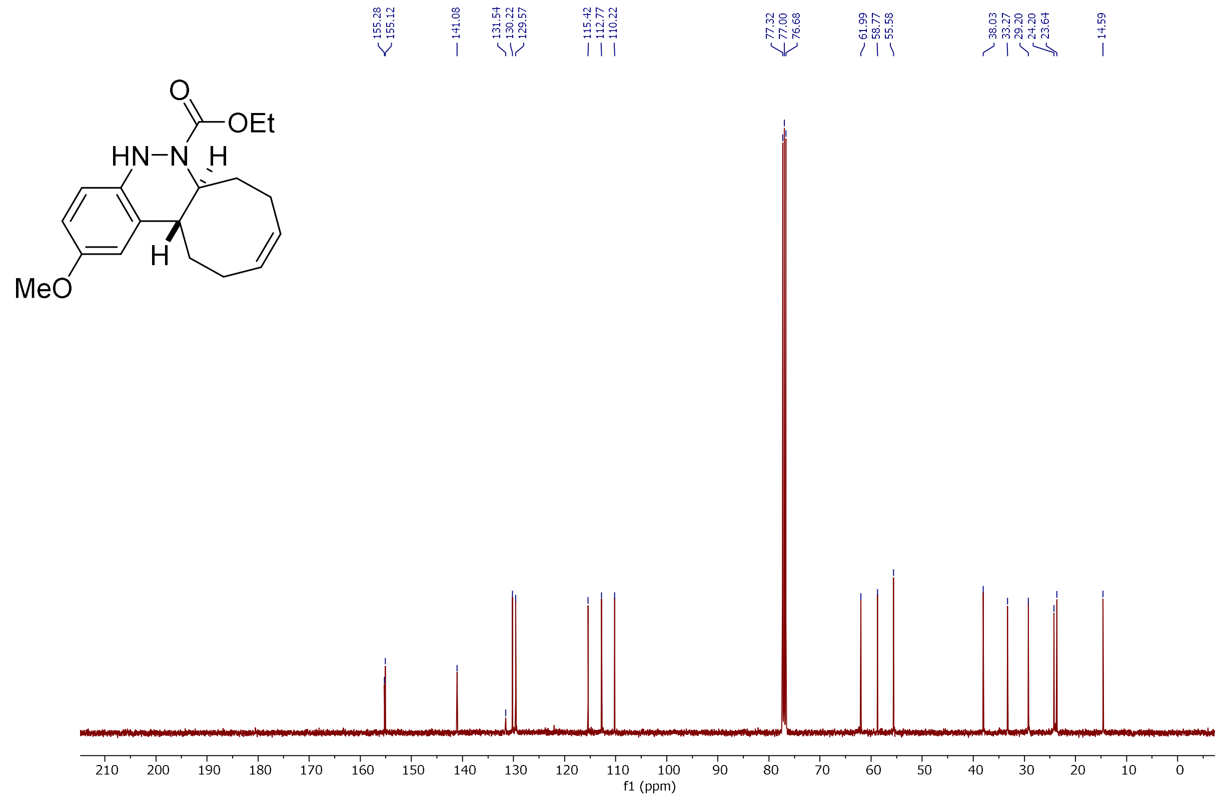

^{1}H NMR (400 MHz, $CDCl_3$) of cinnoline derivative **3d**

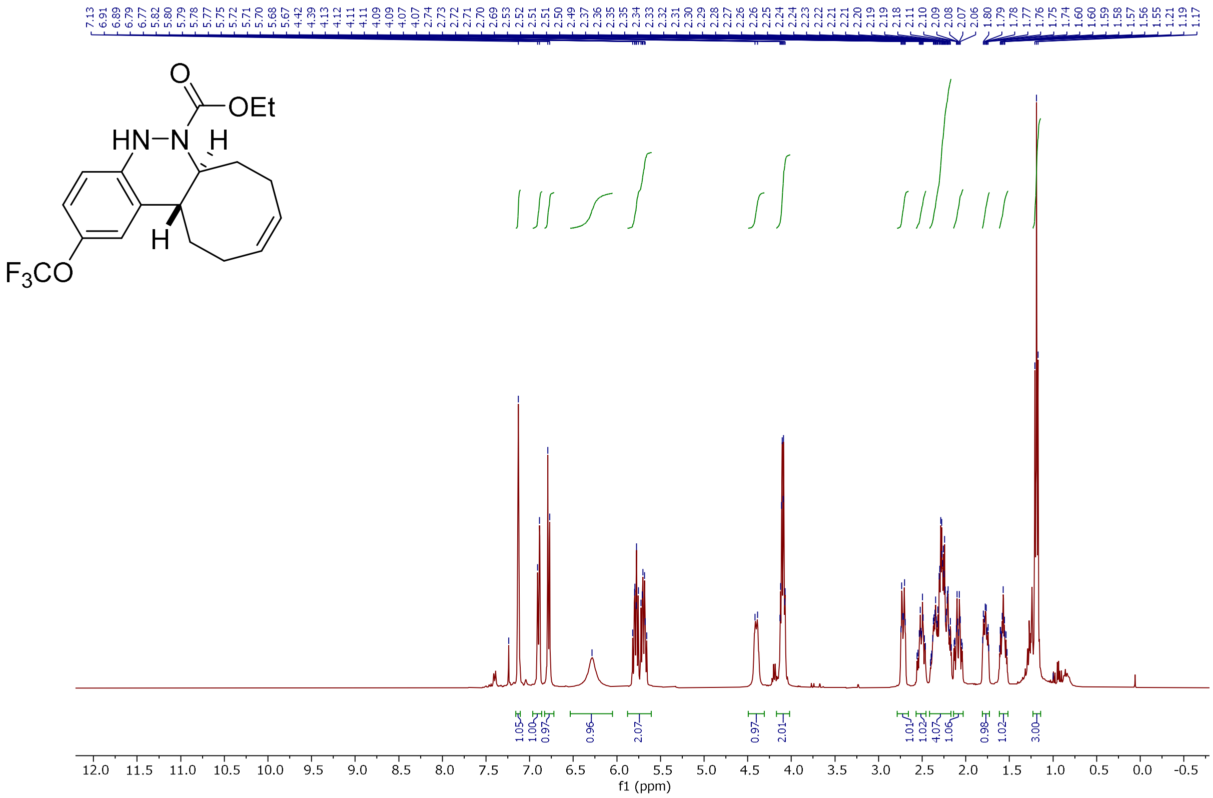

^{13}C{1H} NMR (100 MHz, $CDCl_3$) of cinnoline derivative **3d**

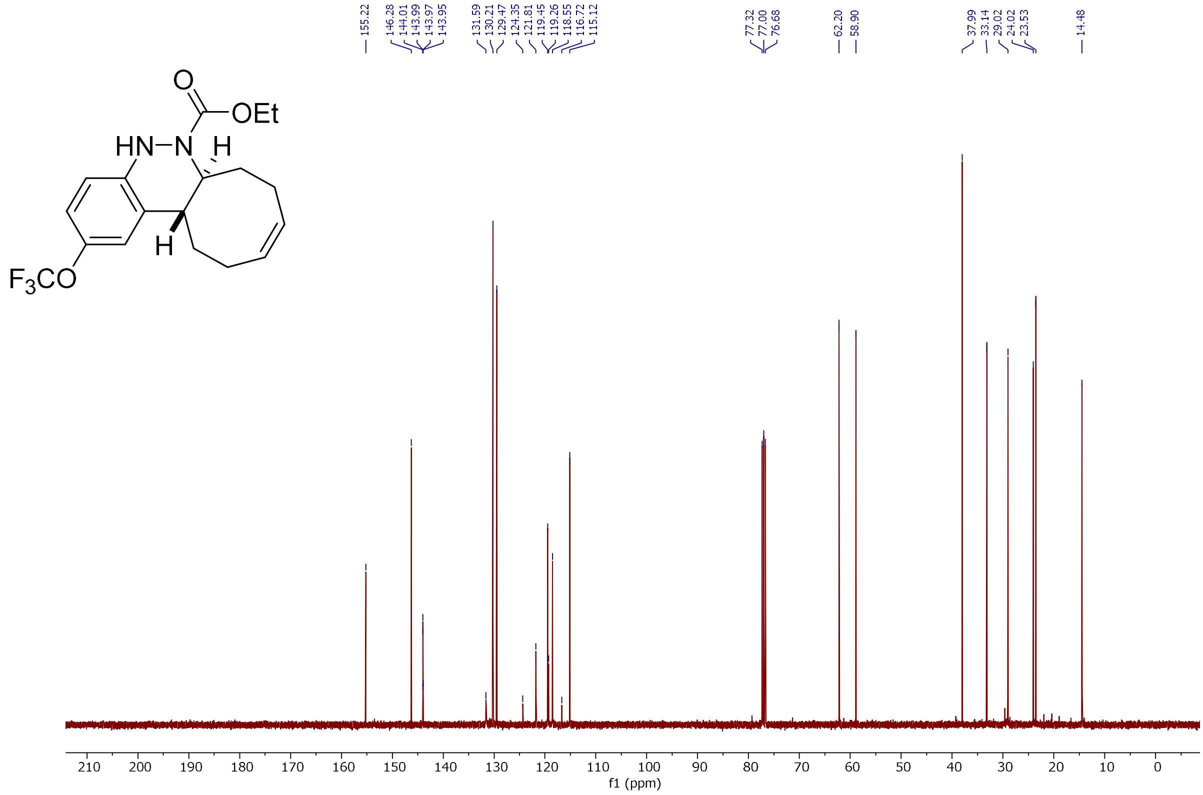

^{19}F (376 MHz, $CDCl_3$) of cinnoline derivative **3d**

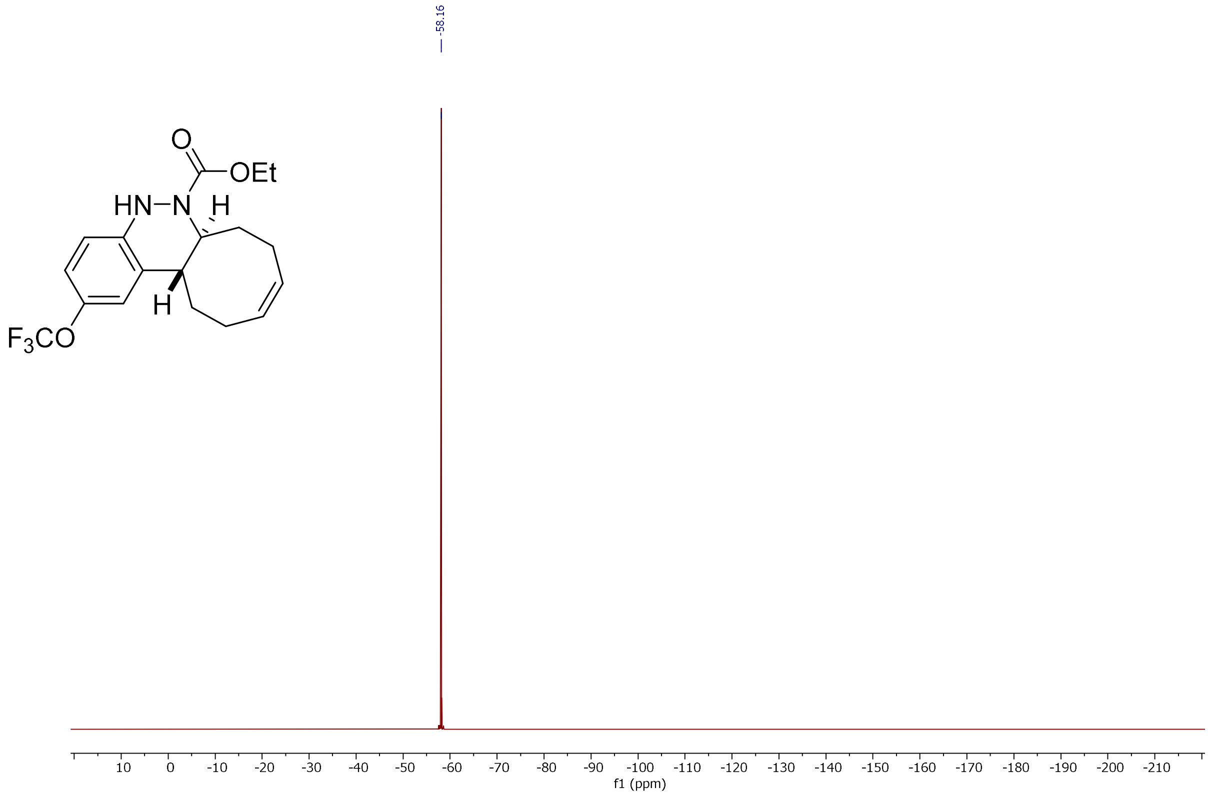

^{1}H NMR (400 MHz, $CDCl_3$) of cinnoline derivative **3e**

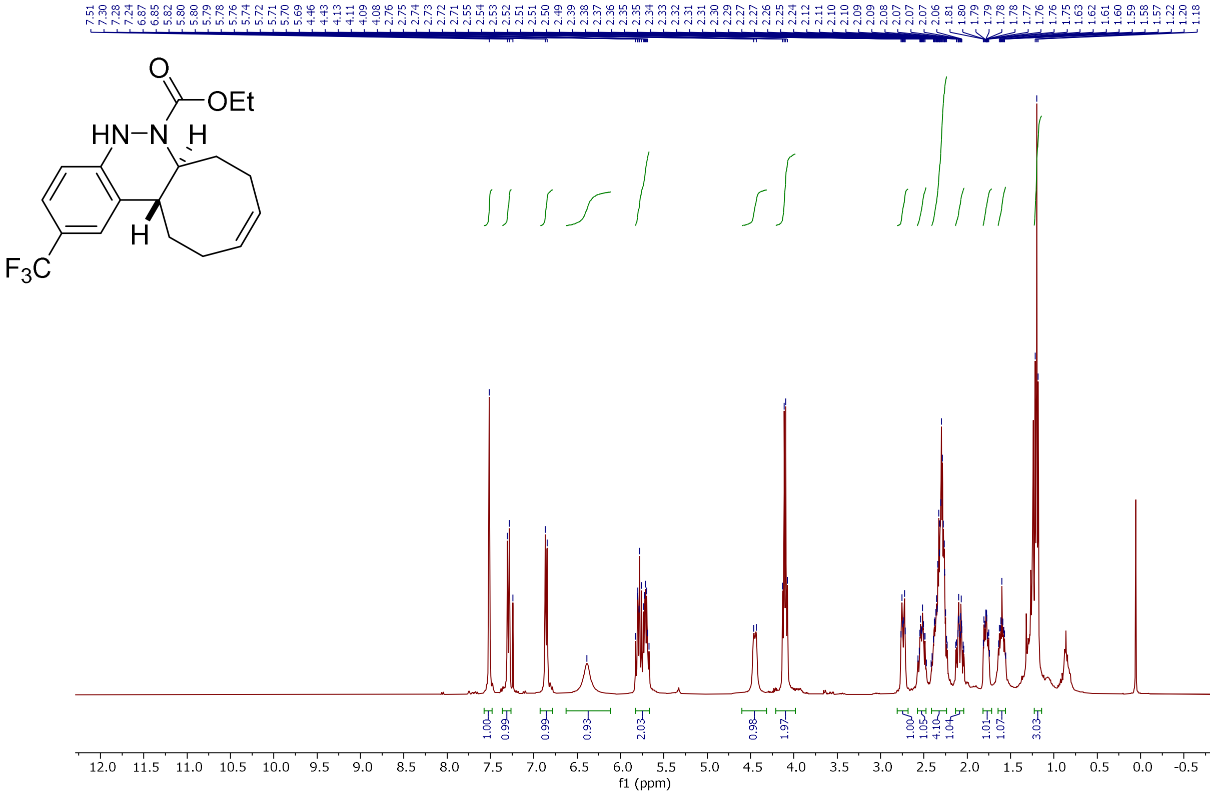

$^{13}C\{1H\}$ NMR (100 MHz, $CDCl_3$) of cinnoline derivative **3e**

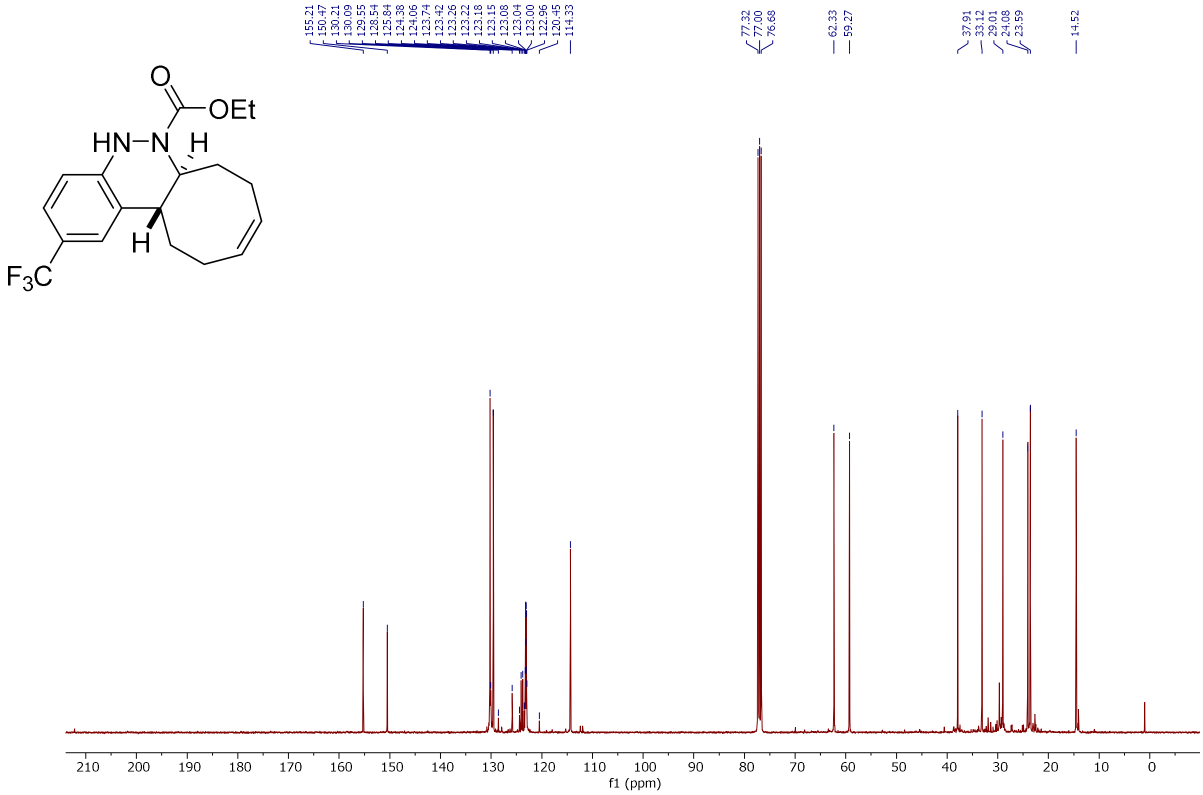

^{19}F (376 MHz, $CDCl_3$) of cinnoline derivative **3e**

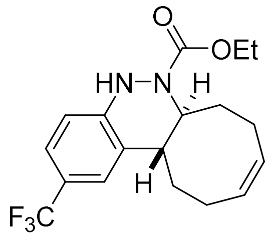

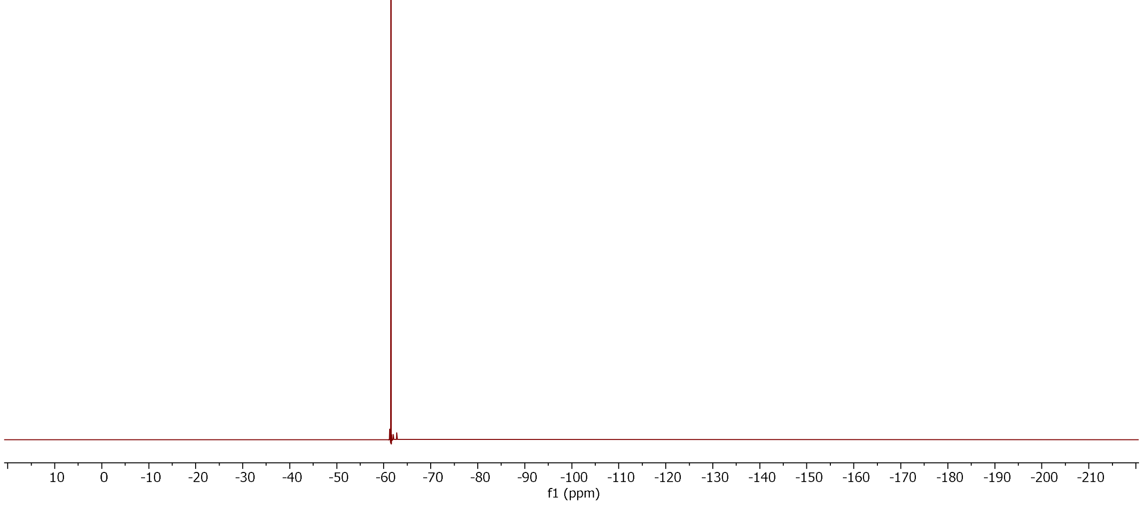

^{1}H NMR (400 MHz, $CDCl_3$) of cinnoline derivative **3f**

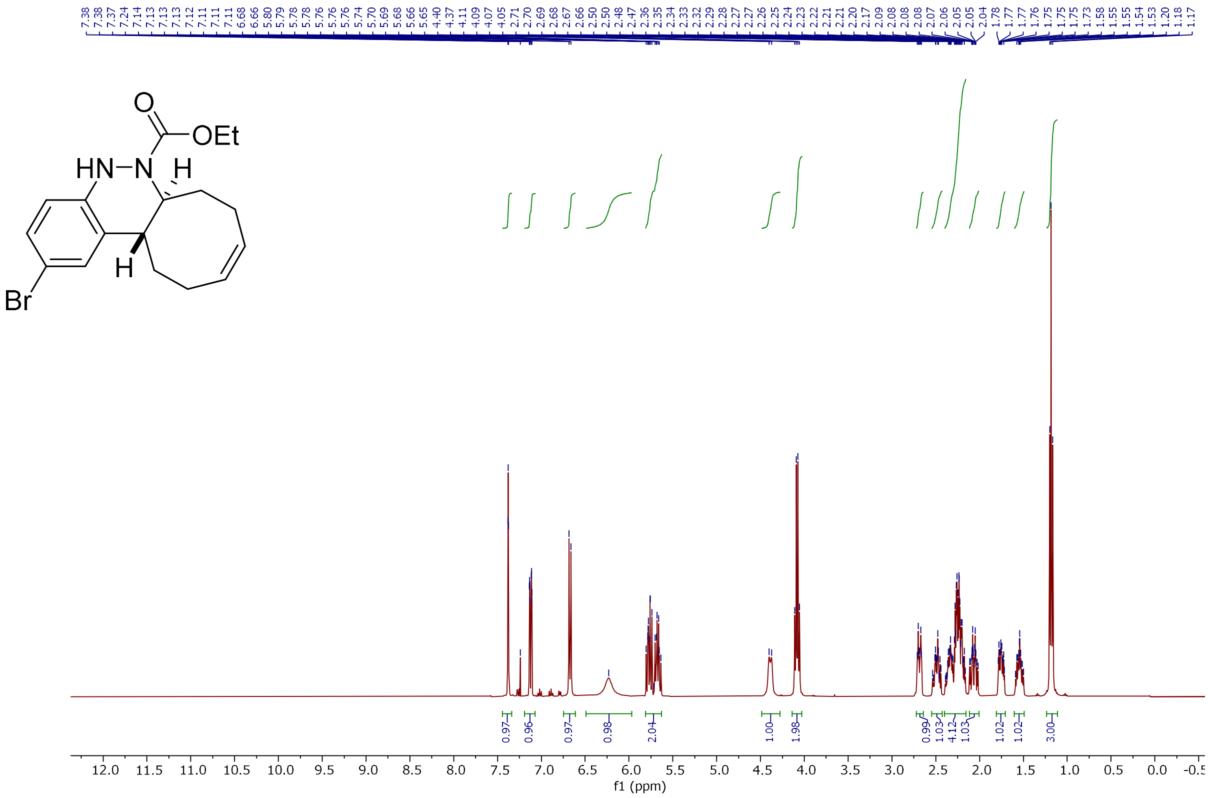

$^{13}C\{1H\}$ NMR (100 MHz, $CDCl_3$) of cinnoline derivative **3f**

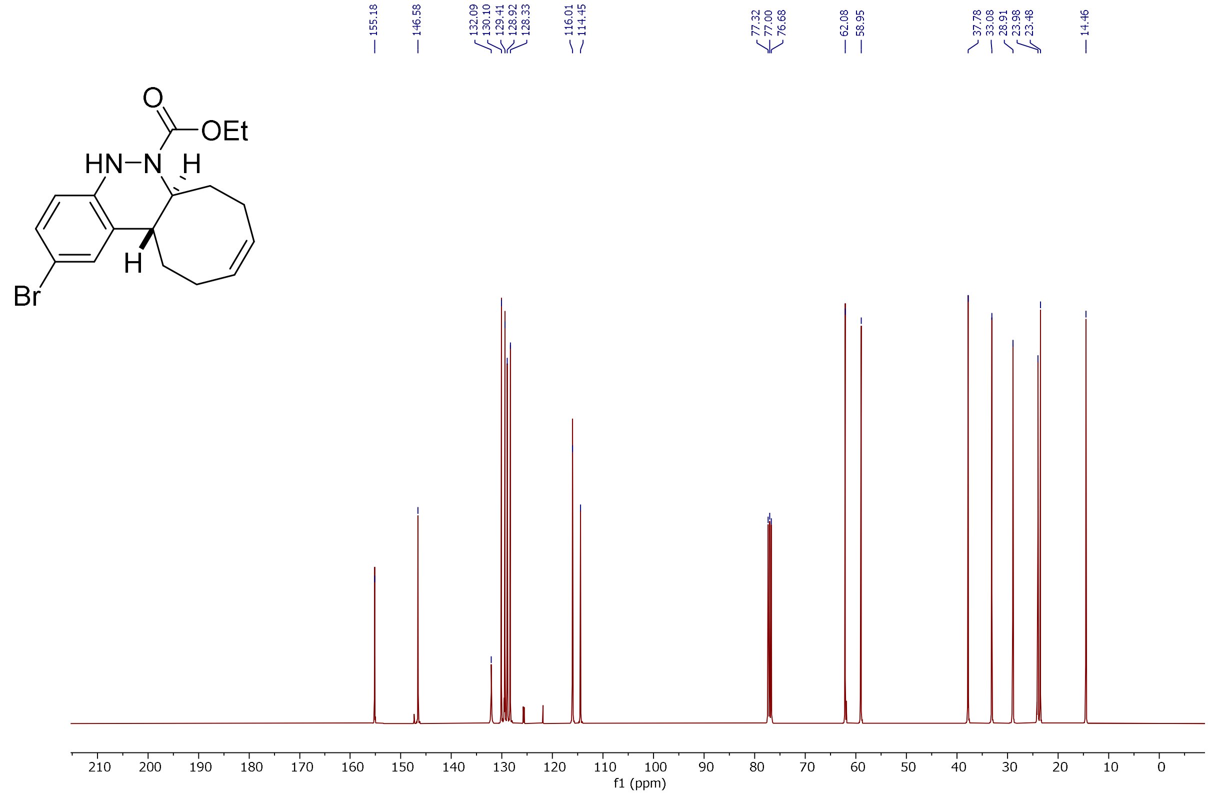

^{1}H NMR (400 MHz, $CDCl_3$) of cinnoline derivative **3g**

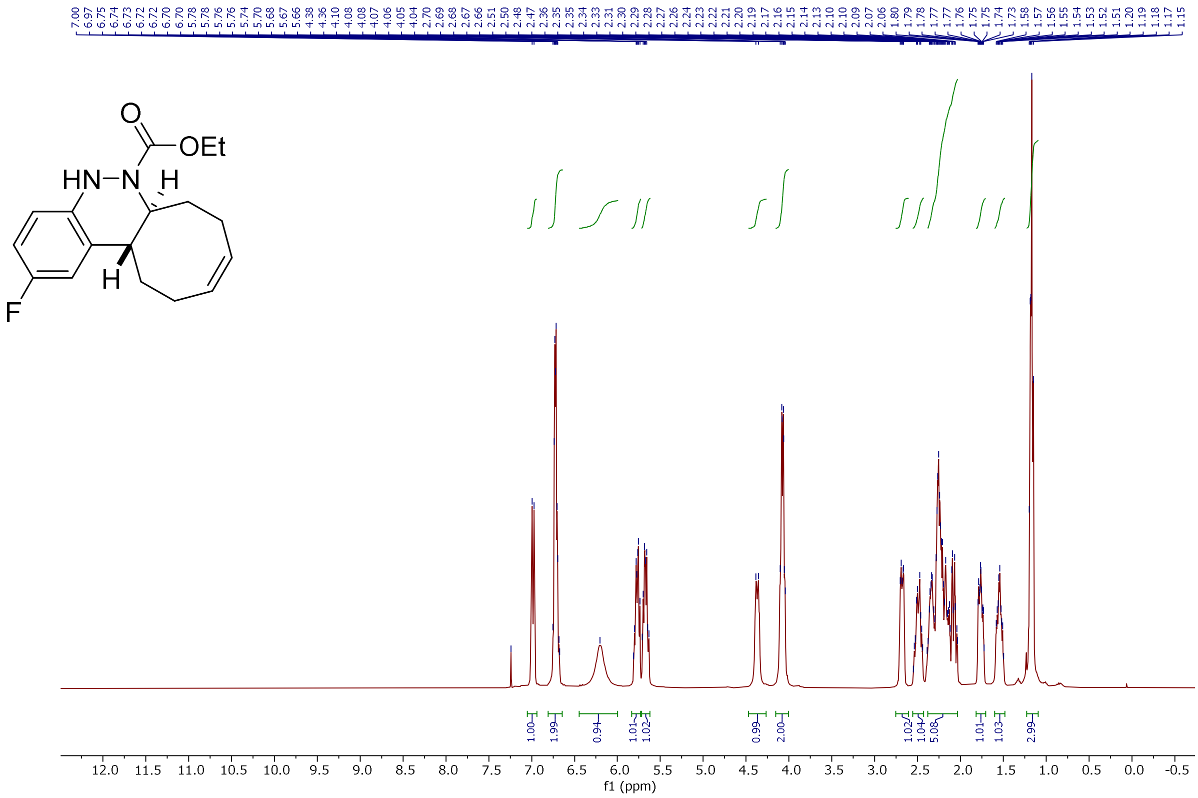

^{13}C{1H} NMR (100 MHz, $CDCl_3$) of cinnoline derivative **3g**

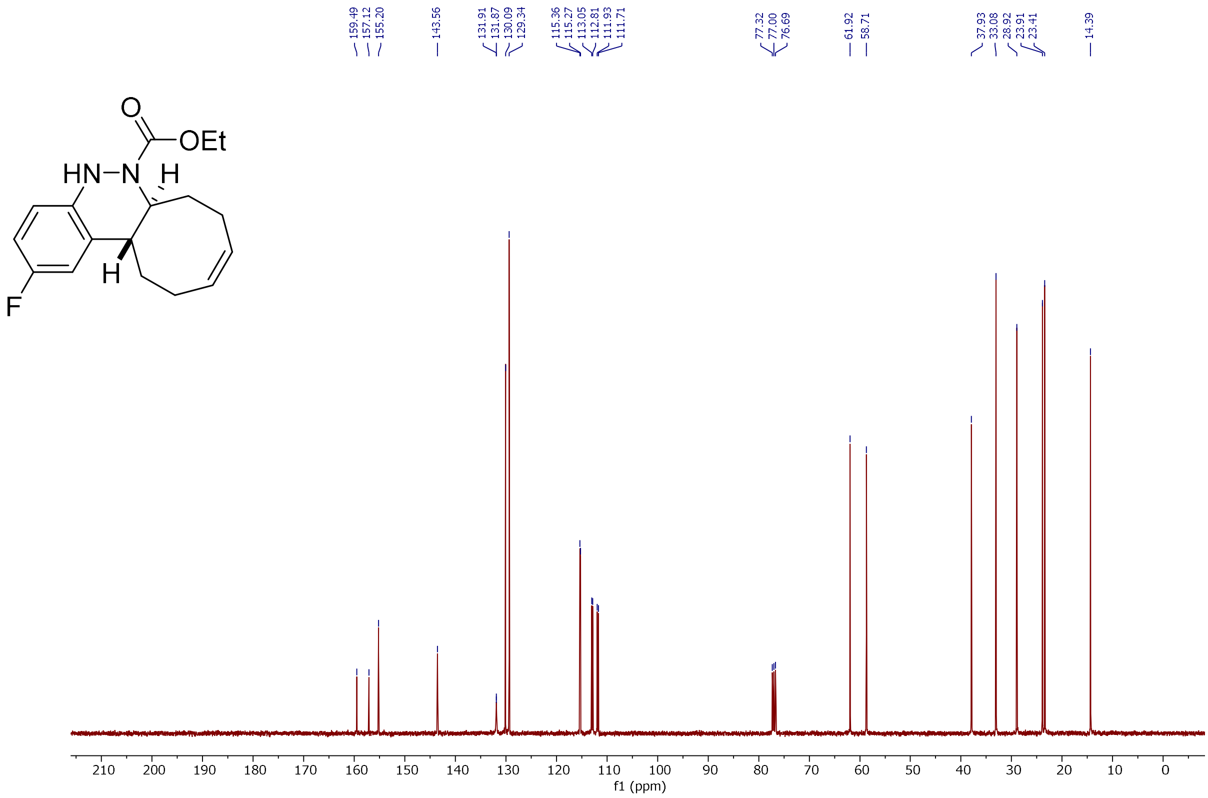

^{19}F (376 MHz, $CDCl_3$) of cinnoline derivative **3g**

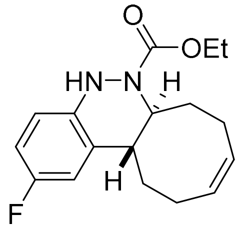

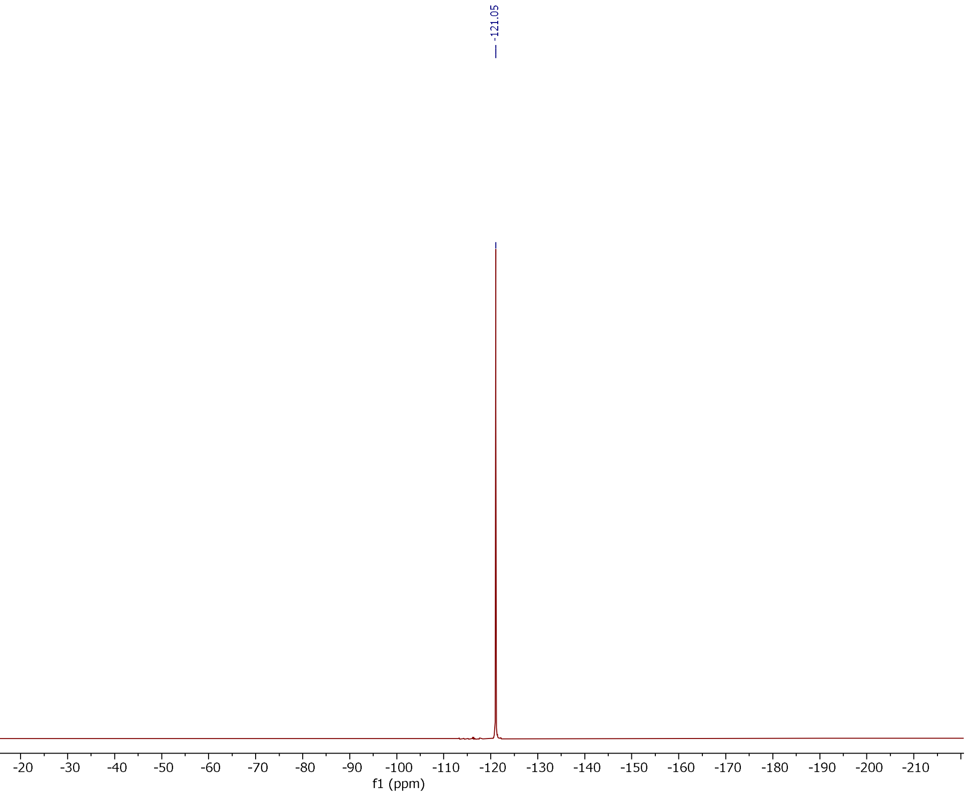

^{1}H NMR (400 MHz, $CDCl_3$) of cinnoline derivative **3h**

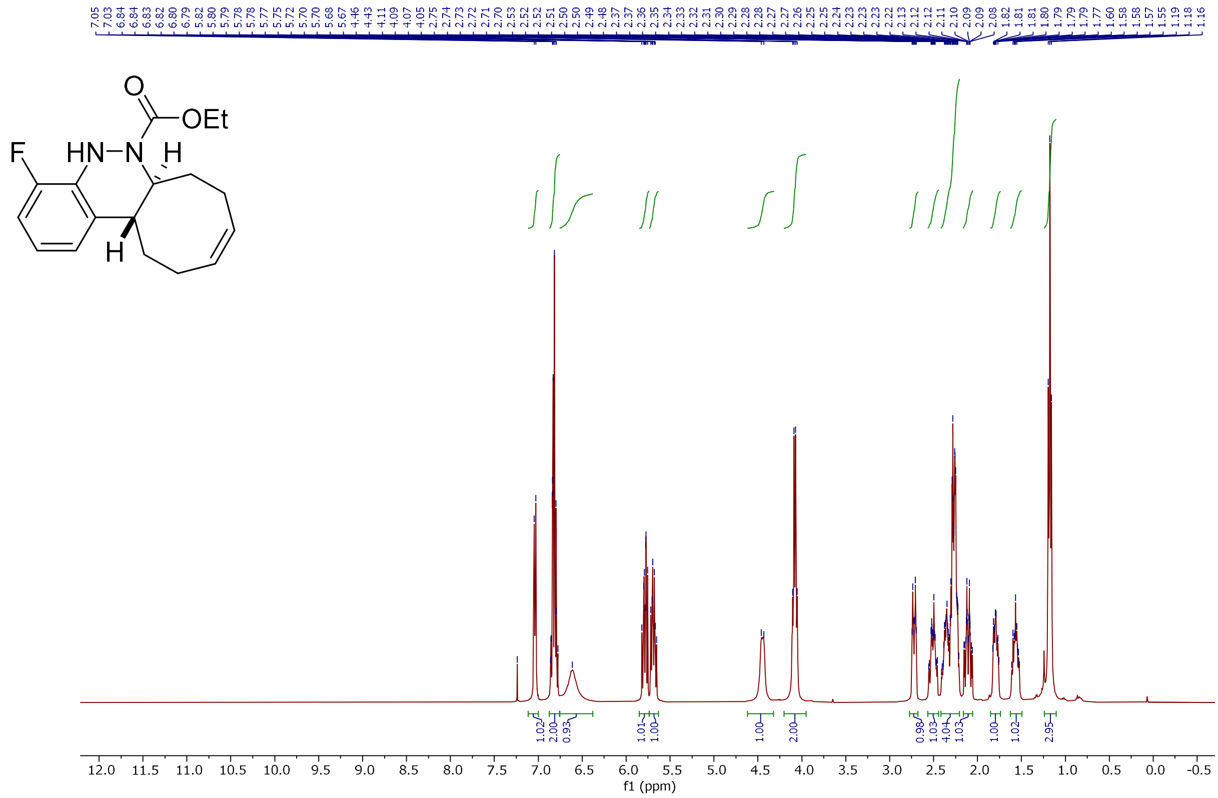

^{13}C{1H} NMR (100 MHz, $CDCl_3$) of cinnoline derivative **3h**

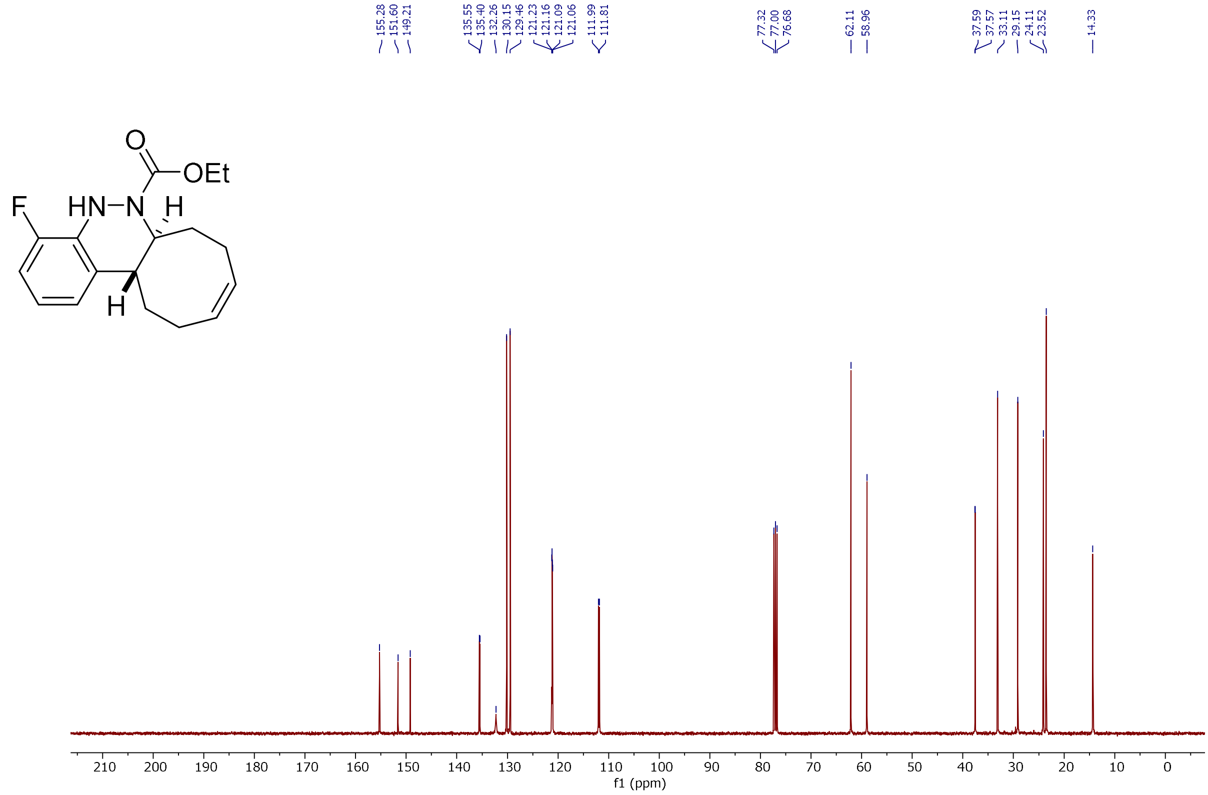

^{19}F (376 MHz, $CDCl_3$) of cinnoline derivative **3h**

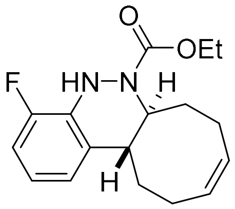

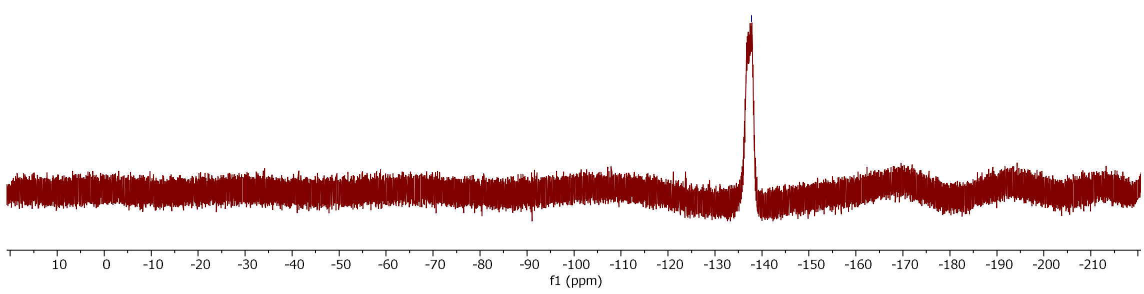

^{1}H NMR (400 MHz, $CDCl_3$) of cinnoline derivative **3i**

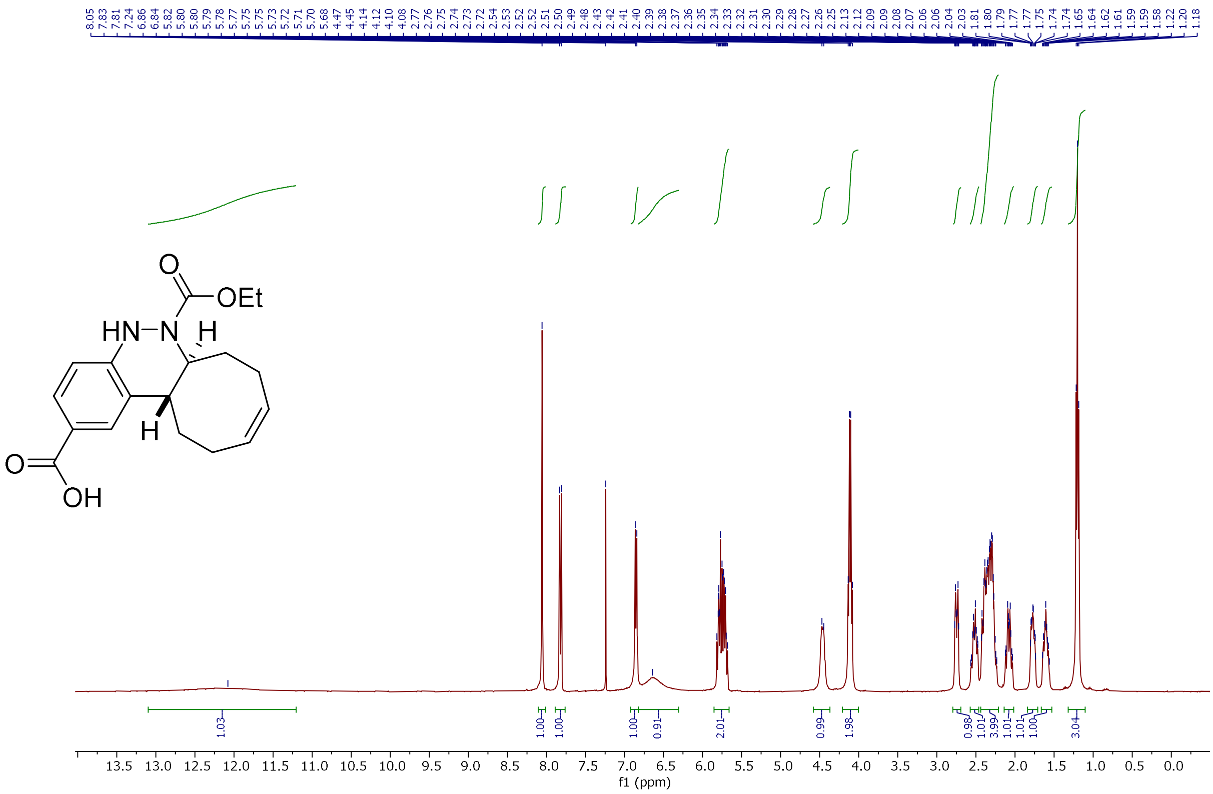

^{13}C{1H} NMR (100 MHz, $CDCl_3$) of cinnoline derivative **3i**

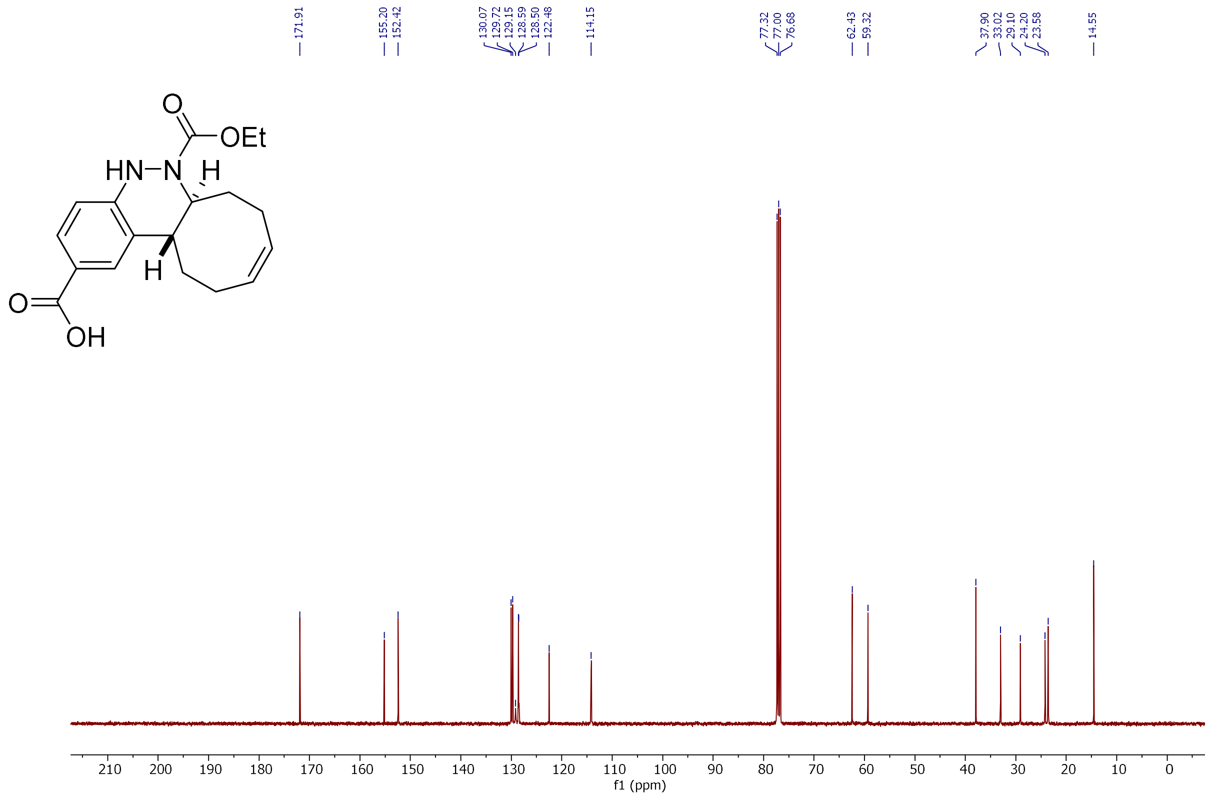

^{1}H NMR (400 MHz, $CDCl_3$) of cinnoline derivative **3j**

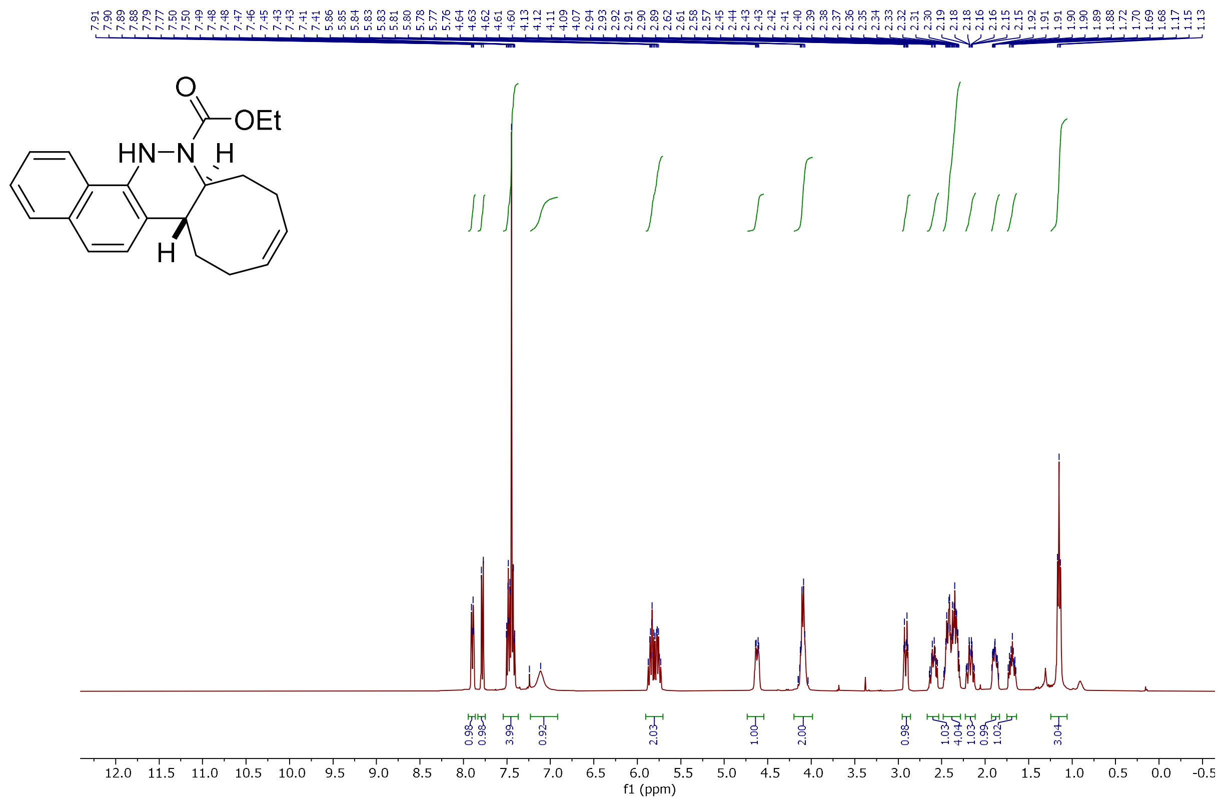

$^{13}C\{1H\}$ NMR (100 MHz, $CDCl_3$) of cinnoline derivative **3j**

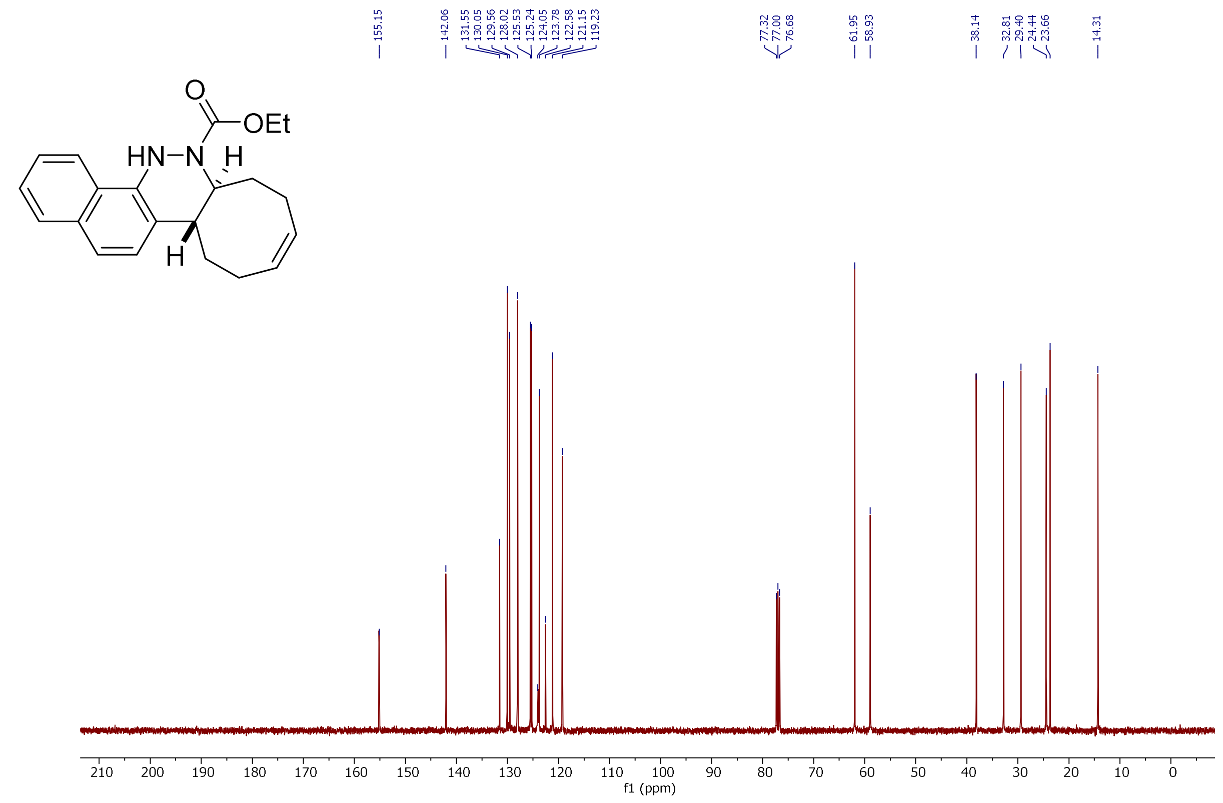

^{1}H NMR (400 MHz, $CDCl_3$) of cinnoline derivative **3k**

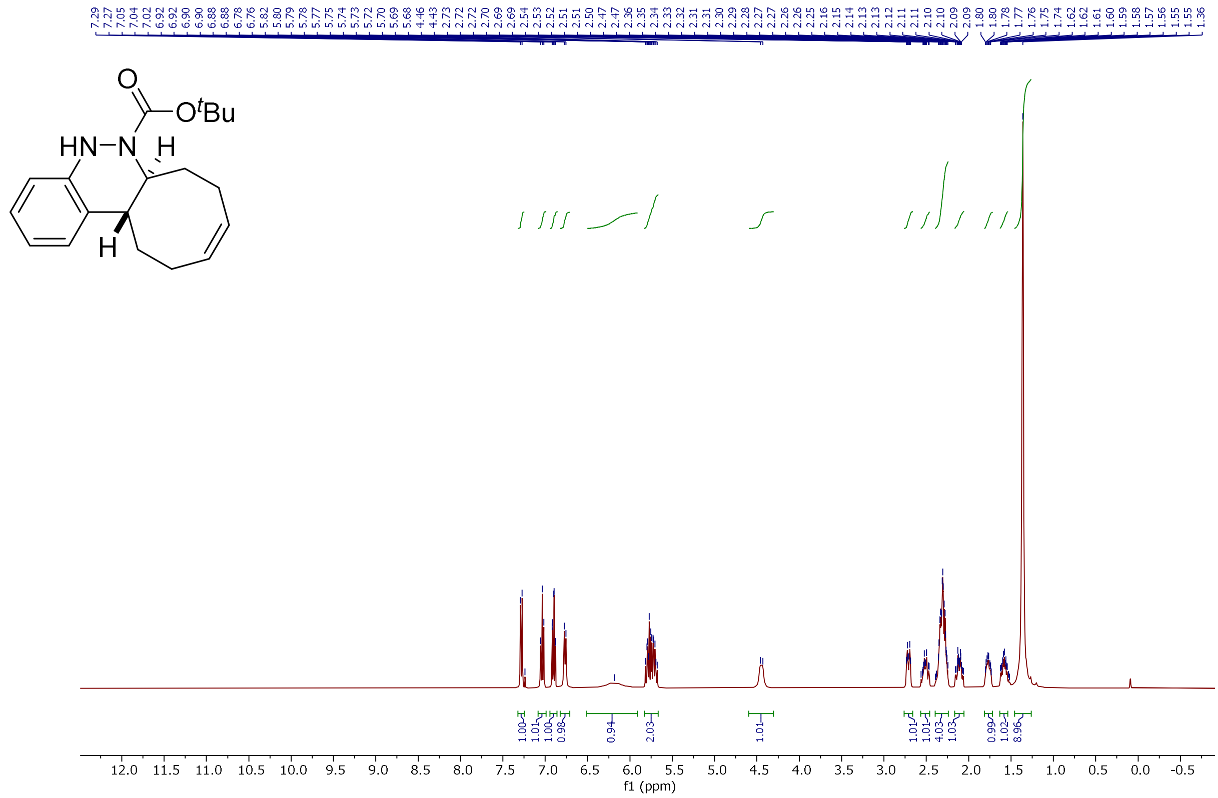

^{13}C{1H} NMR (100 MHz, $CDCl_3$) of cinnoline derivative **3k**

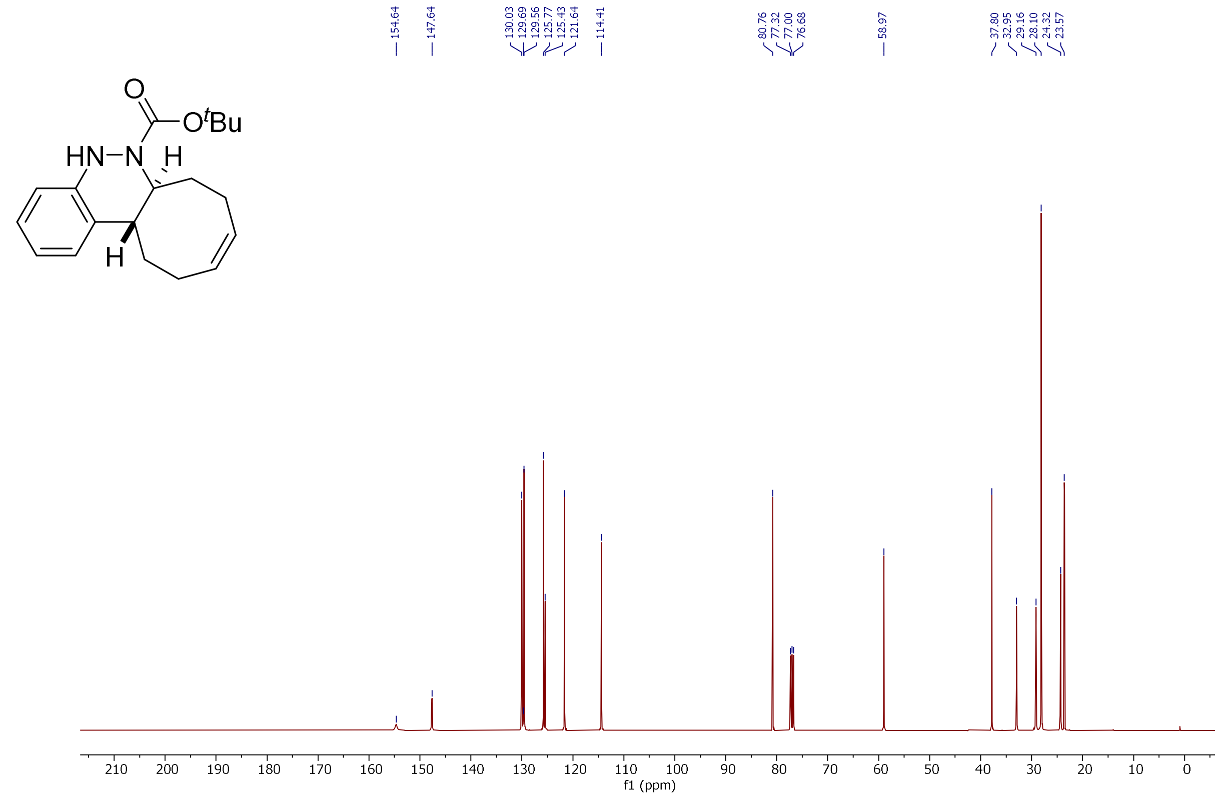

^{1}H NMR (400 MHz, $CDCl_3$) of cinnoline derivative **3l**

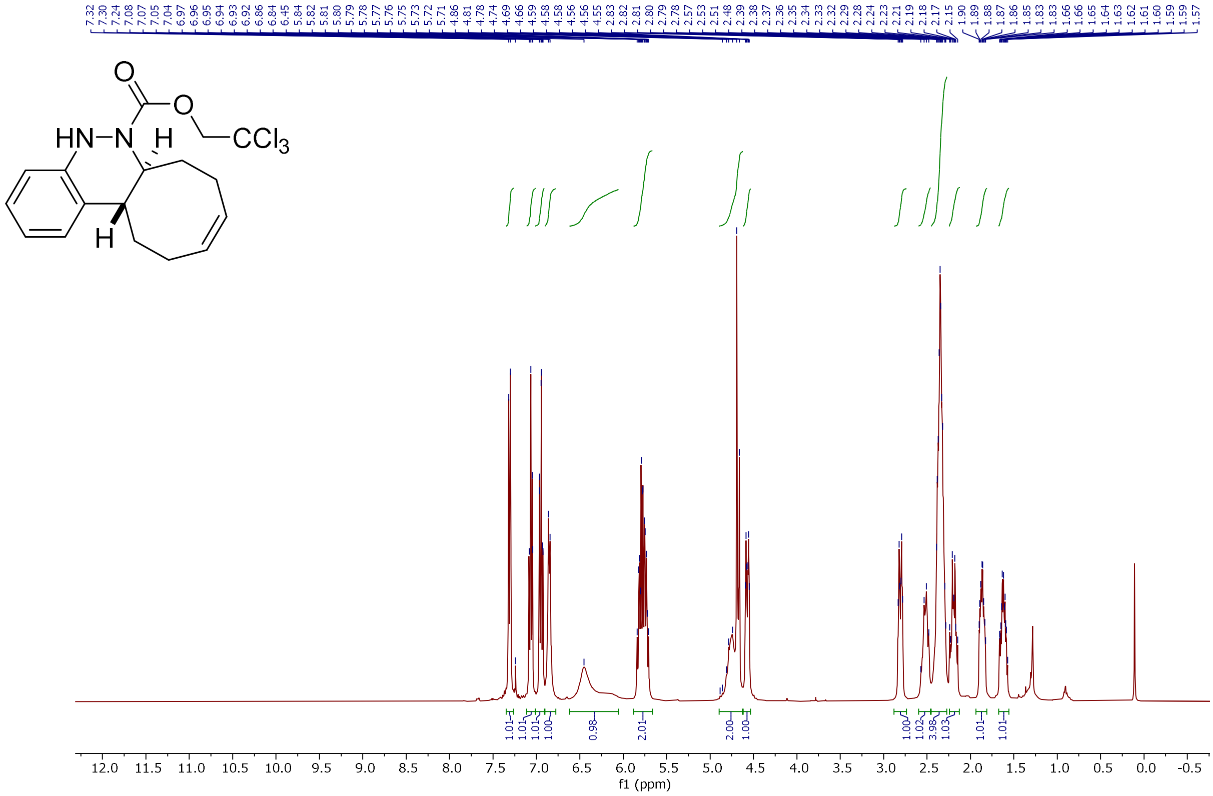

^{13}C{1H} NMR (100 MHz, $CDCl_3$) of cinnoline derivative **3l**

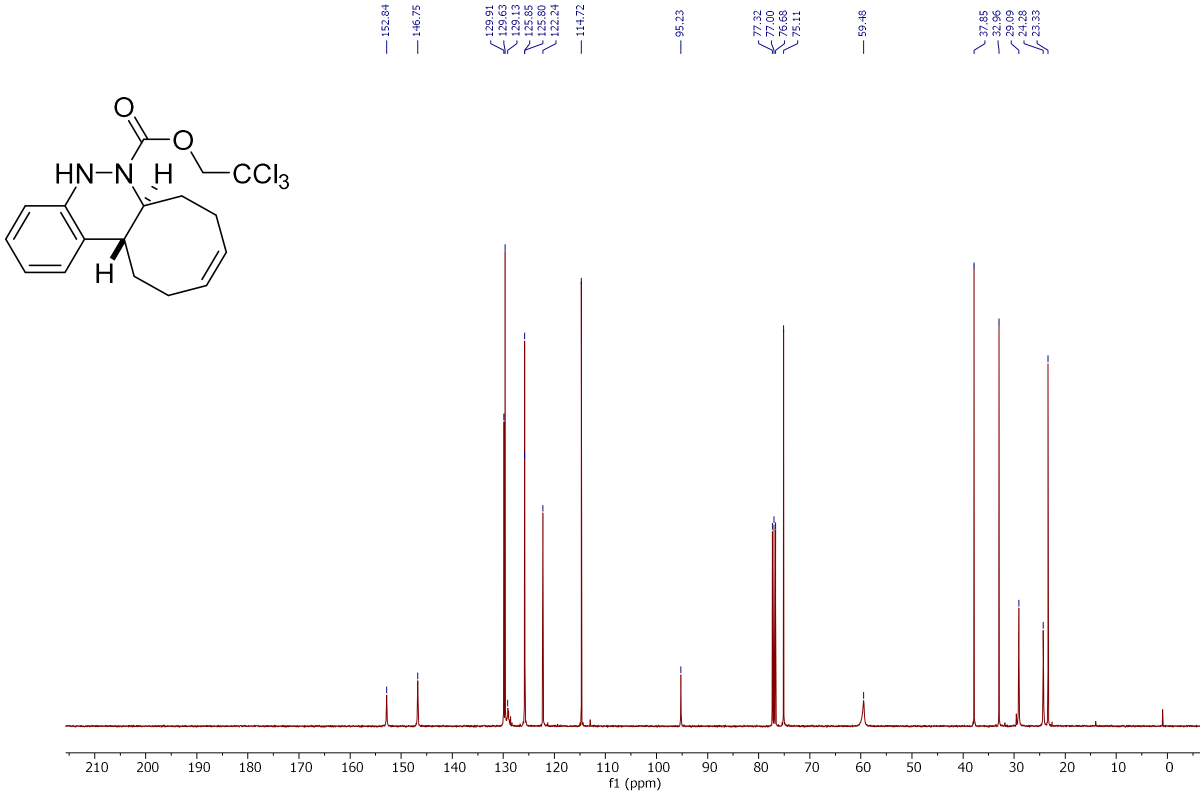

^{1}H NMR (400 MHz, $CDCl_3$) of cinnoline derivative **3m**

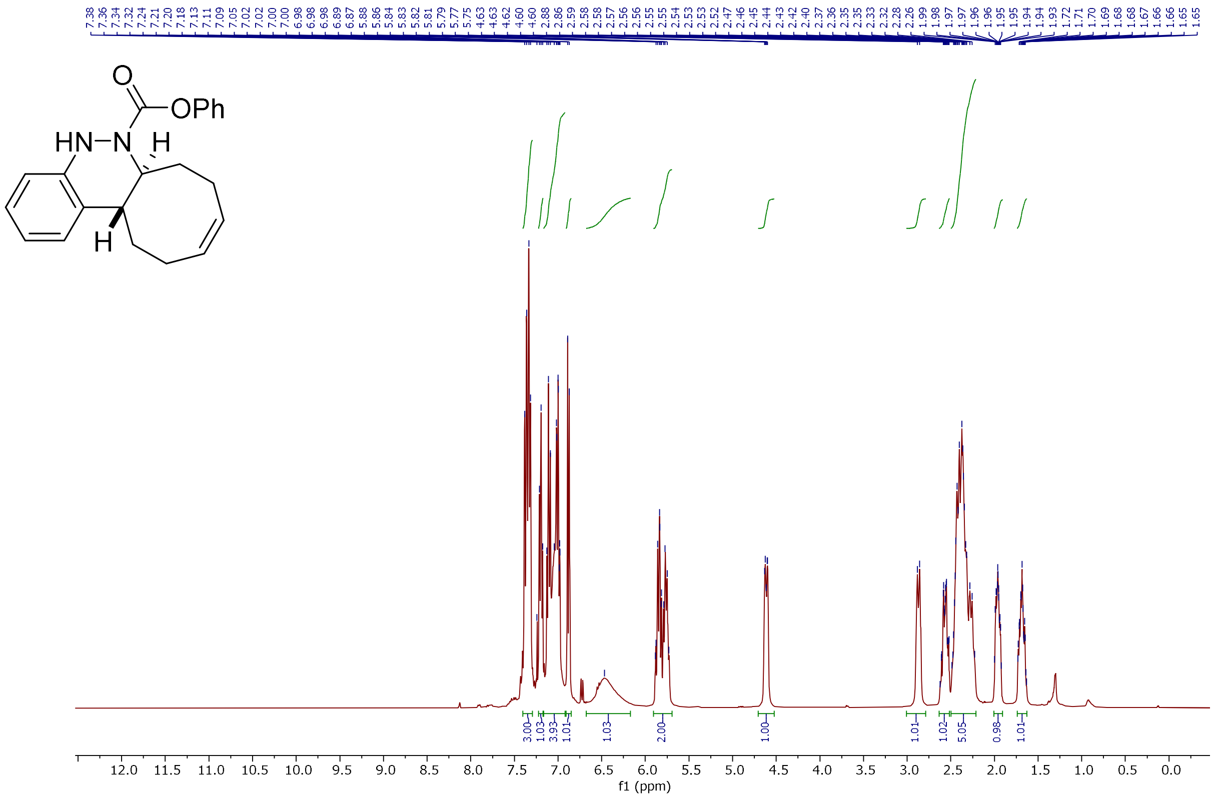

^{13}C{1H} NMR (100 MHz, $CDCl_3$) of cinnoline derivative **3m**

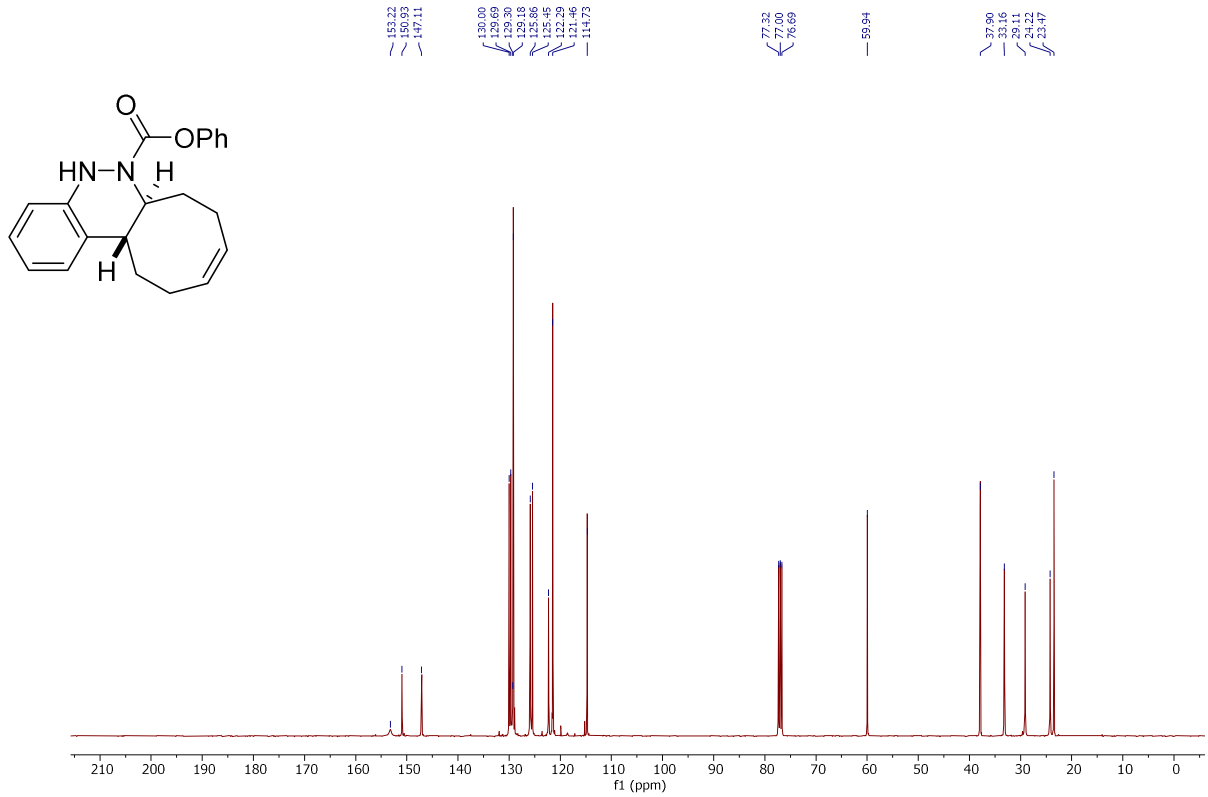

^{1}H NMR (400 MHz, $CDCl_3$) of cinnoline derivative **3n**

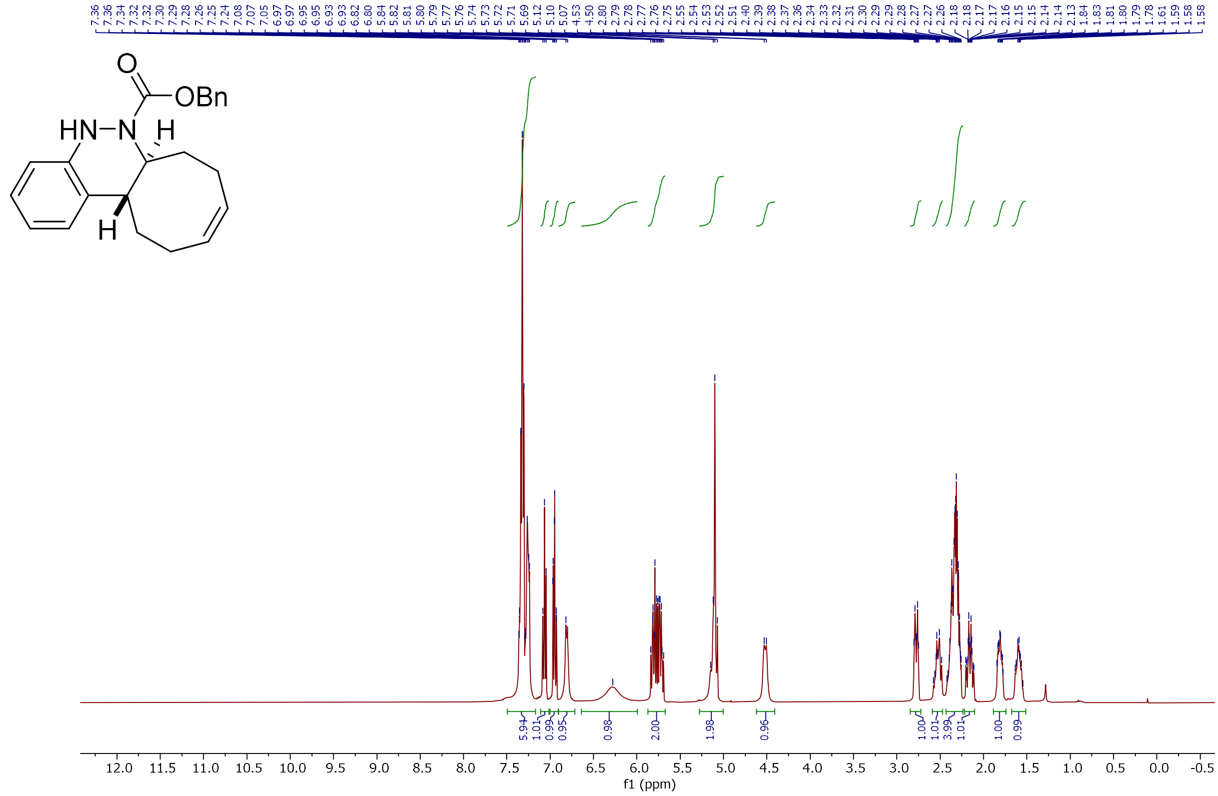

$^{13}C\{1H\}$ NMR (100 MHz, $CDCl_3$) of cinnoline derivative **3n**

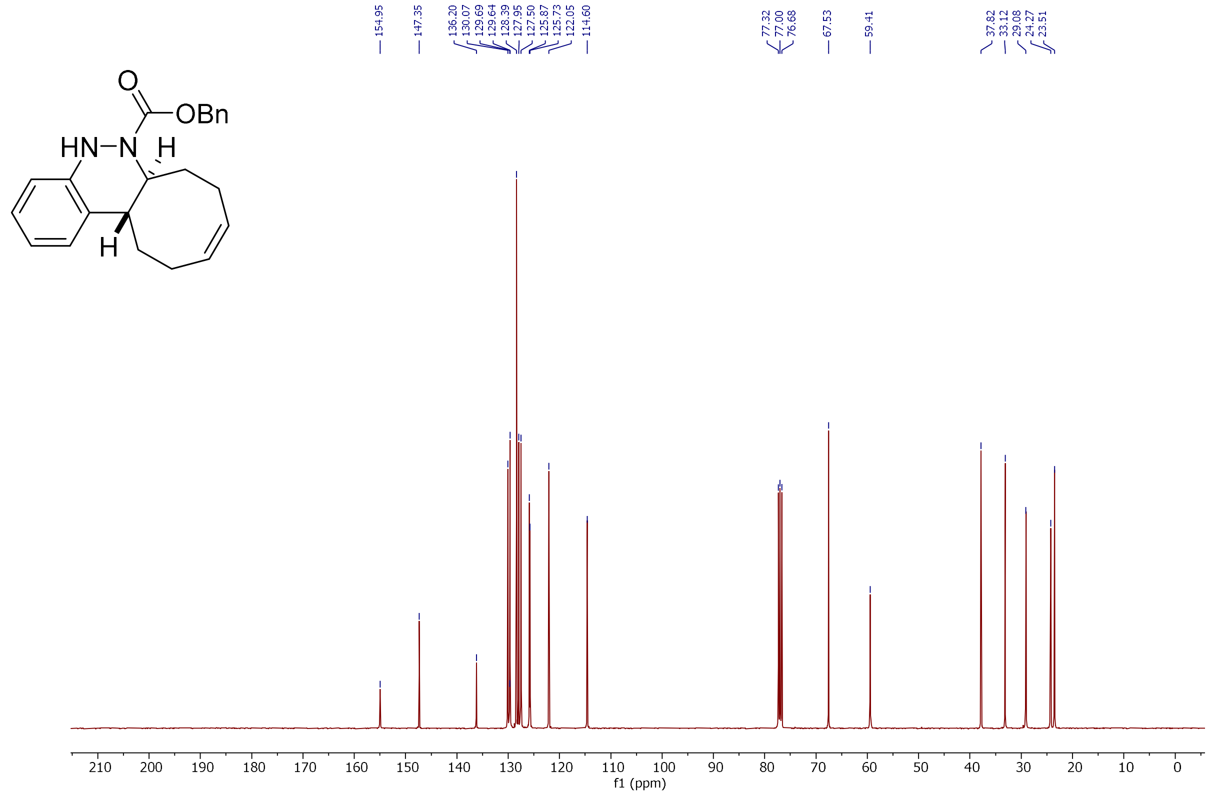

^{1}H NMR (400 MHz, $CDCl_3$) of cinnoline derivative **3o**

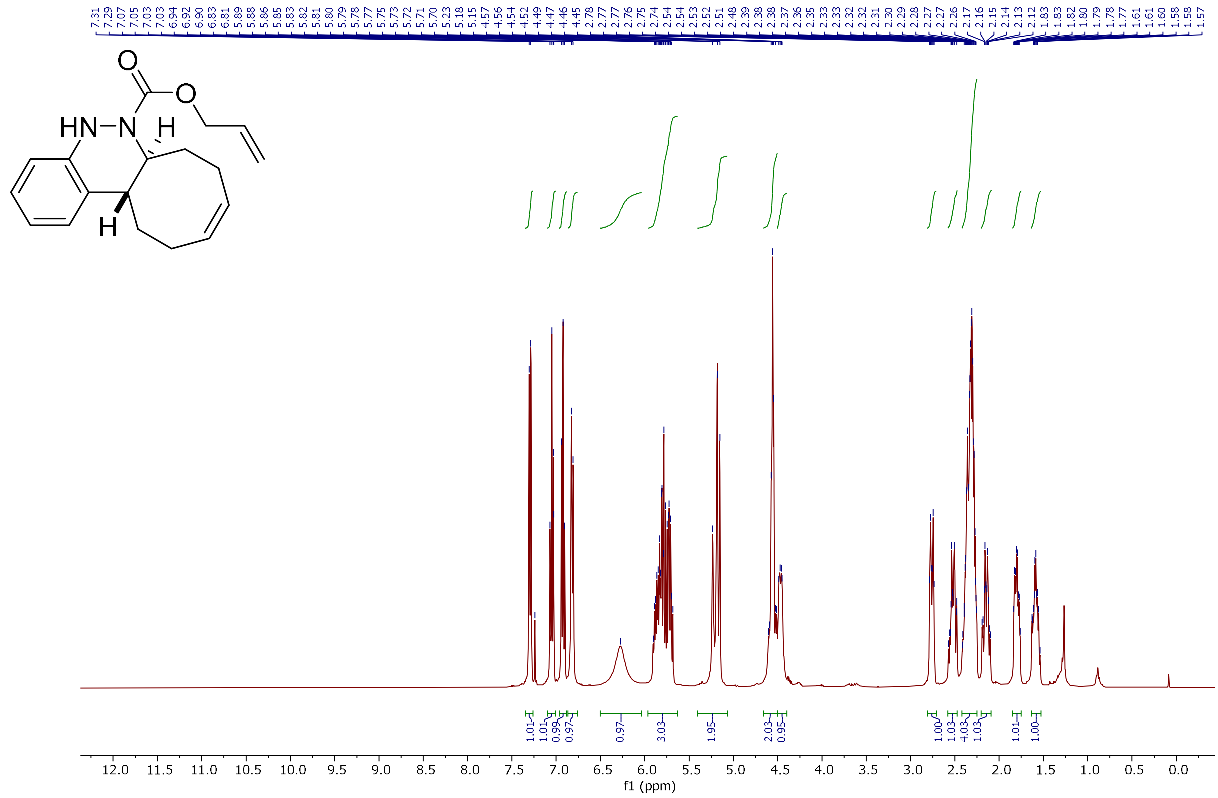

^{13}C{1H} NMR (100 MHz, $CDCl_3$) of cinnoline derivative **3o**

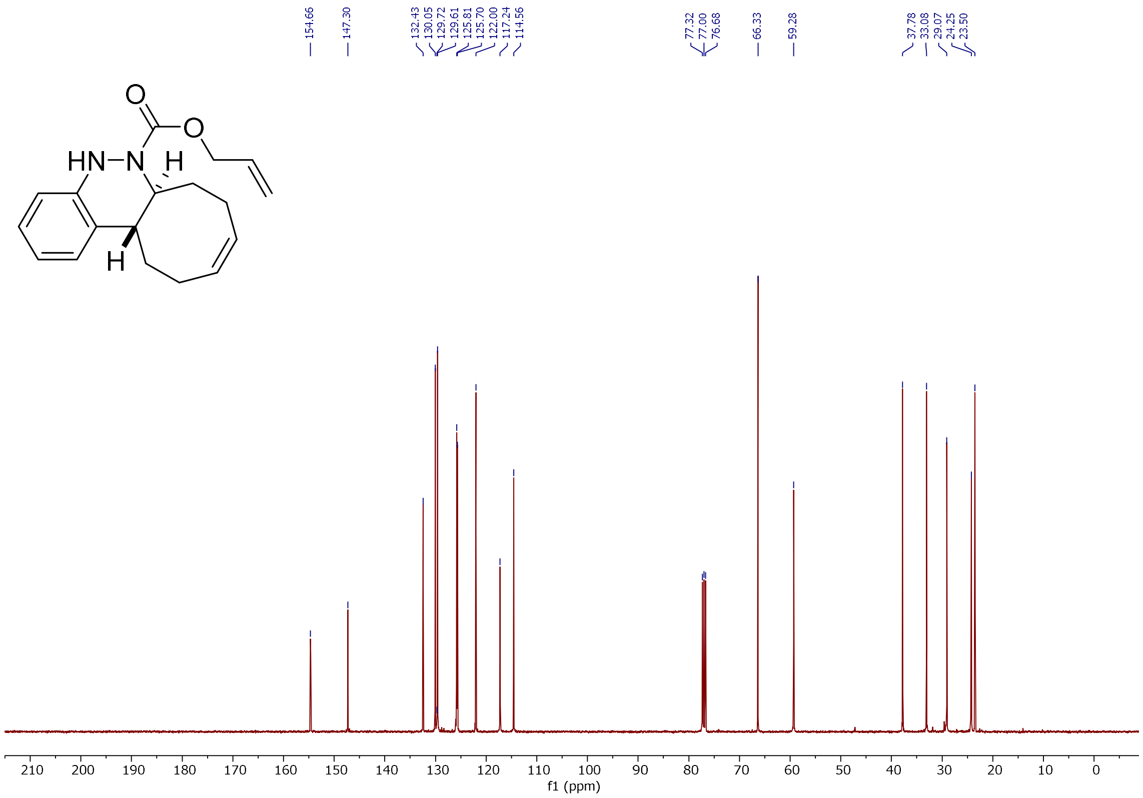

^{1}H NMR (400 MHz, $CDCl_3$) of cinnoline derivative **4**

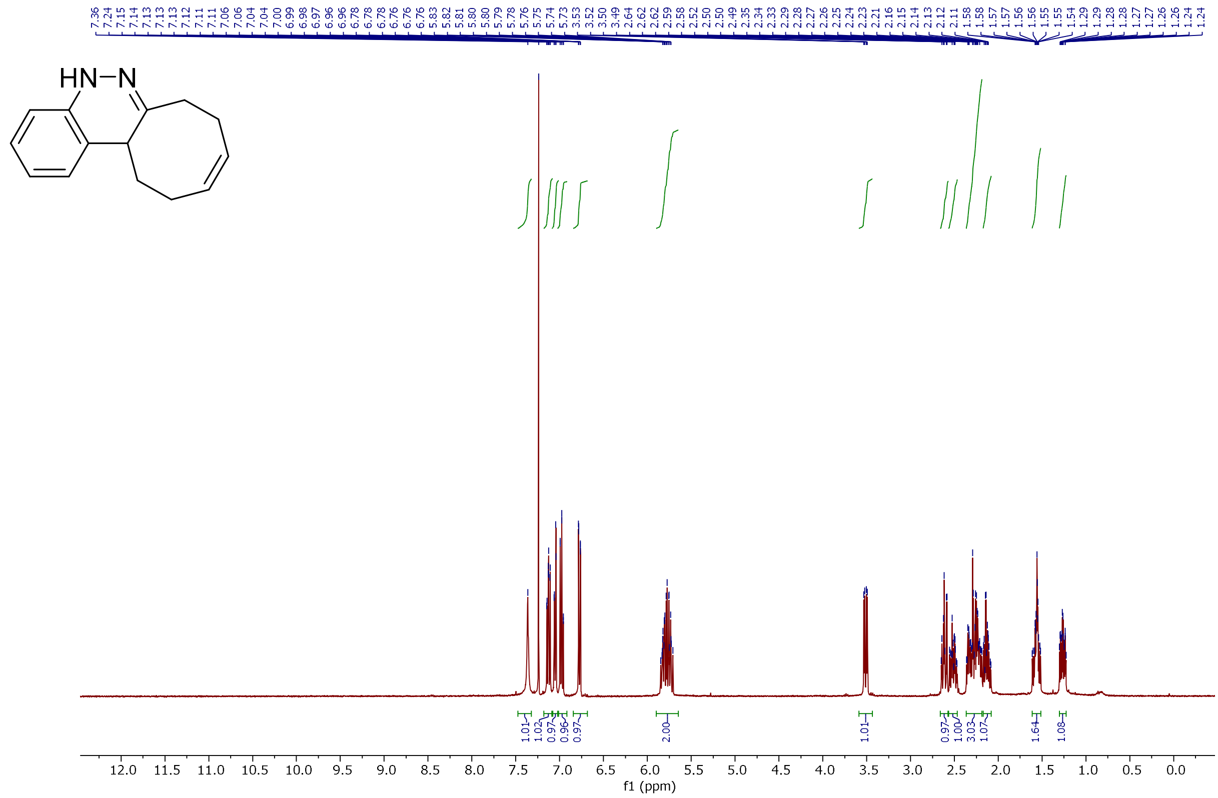

^{13}C{1H} NMR (100 MHz, $CDCl_3$) of cinnoline derivative **4**

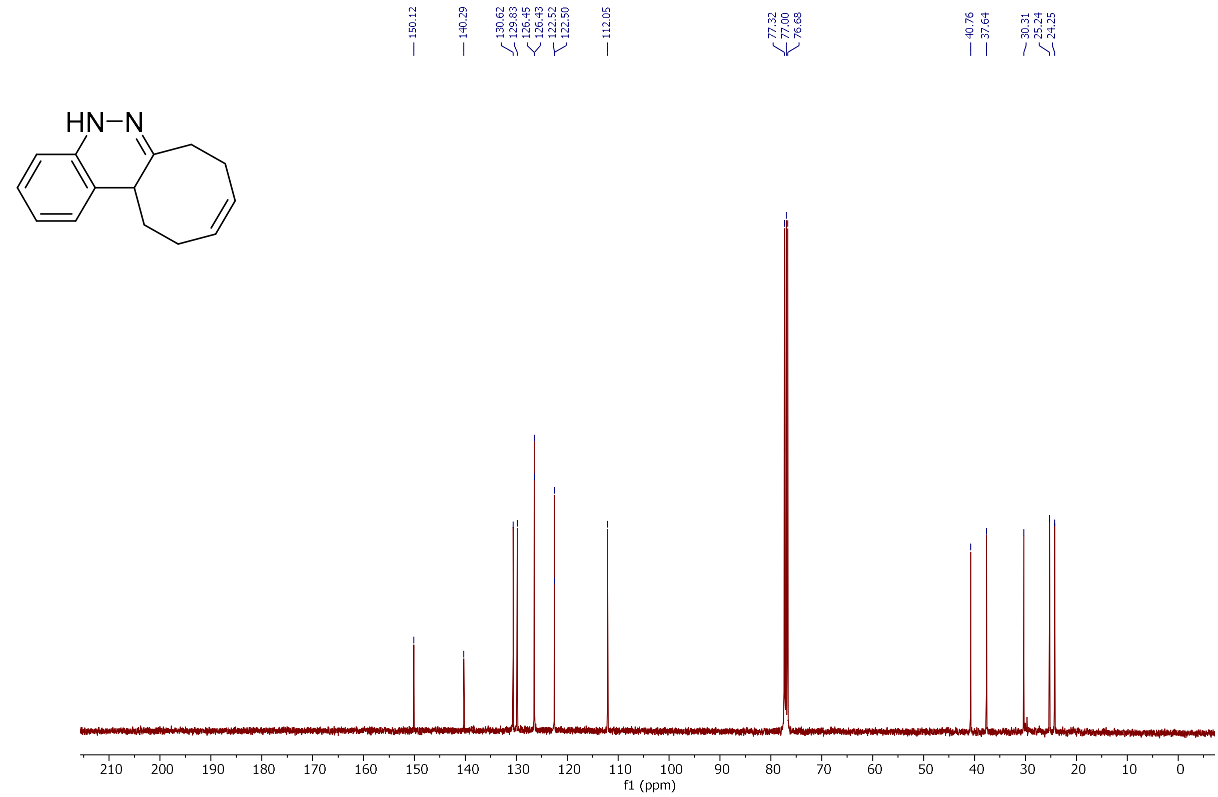

^{1}H NMR (400 MHz, $CDCl_3$) of cinnoline derivatives **5a** and **5a'**

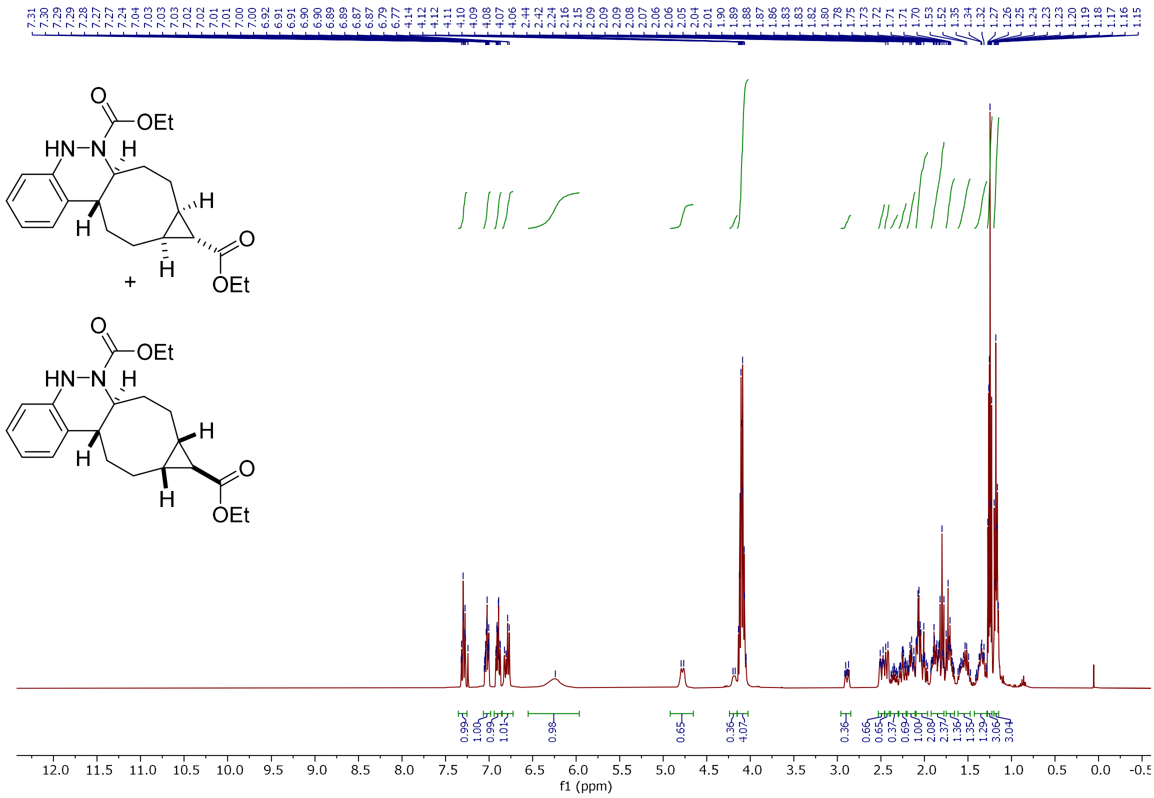

^{13}C{1H} NMR (100 MHz, $CDCl_3$) of cinnoline derivatives **5a** and **5a'**

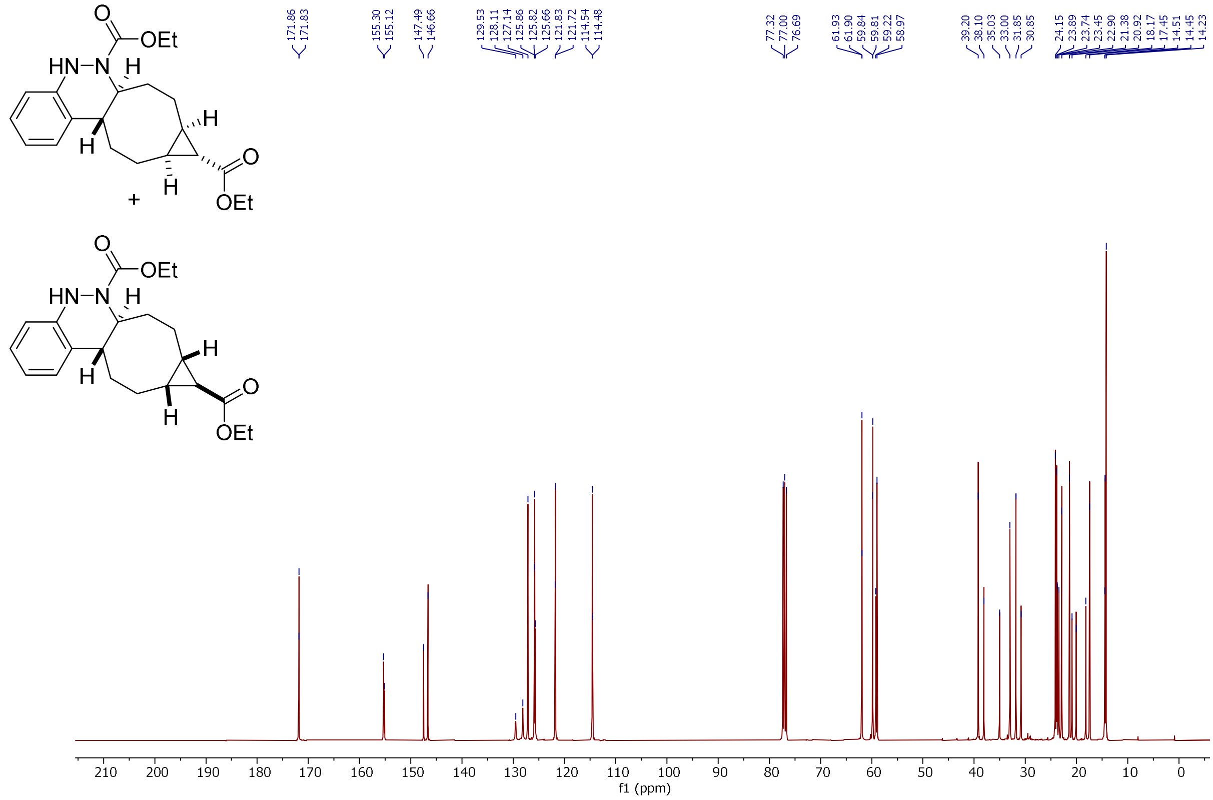

^{1}H NMR (400 MHz, $CDCl_3$) of cinnoline derivative **6**

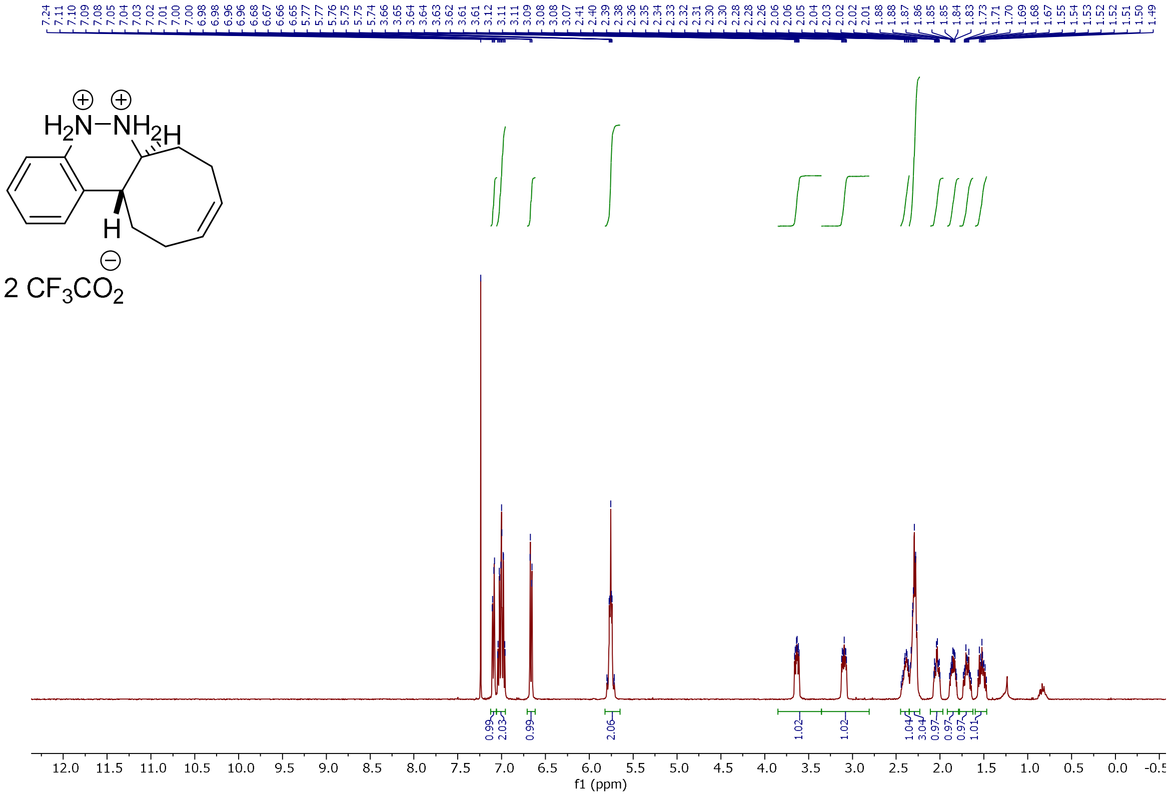

^{13}C{1H} NMR (100 MHz, $CDCl_3$) of cinnoline derivative **6**

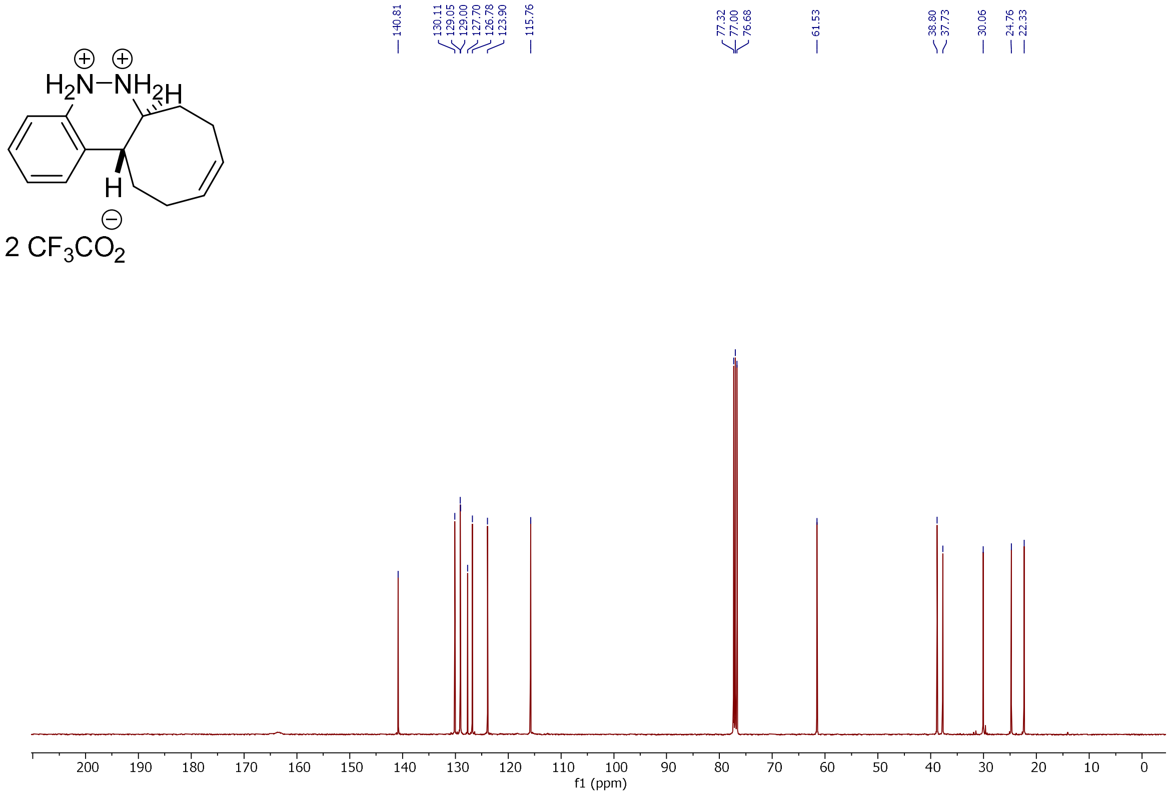

^{19}F (376 MHz, $CDCl_3$) of cinnoline derivative **6**

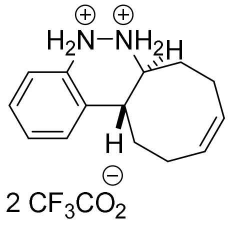

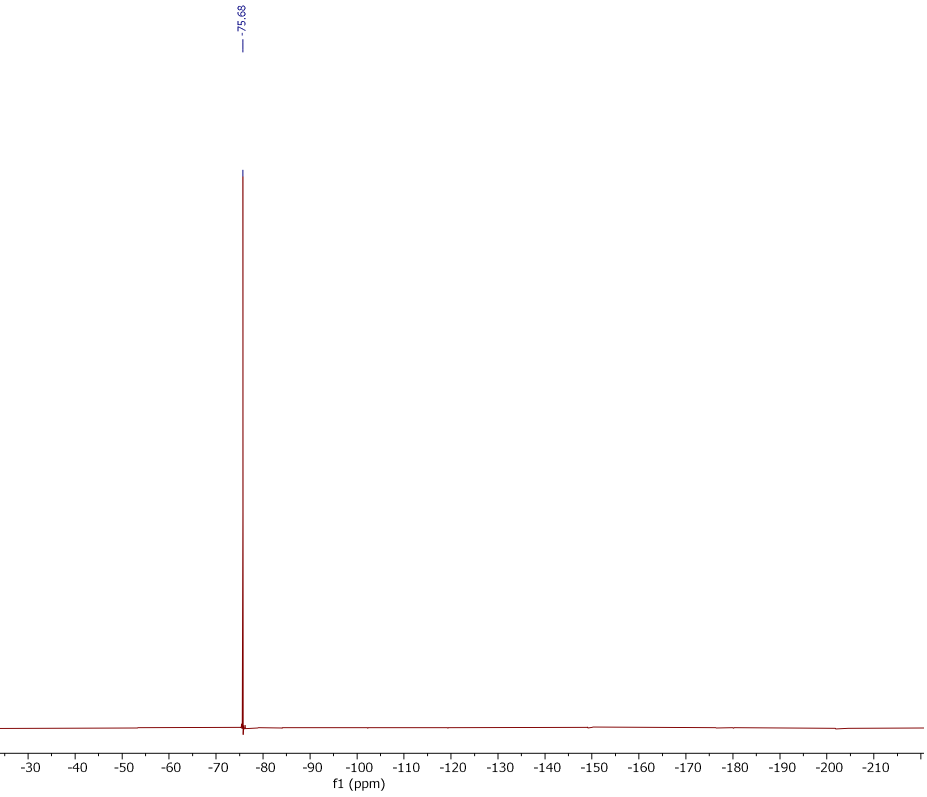

^{1}H NMR (400 MHz, $CDCl_3$) of compound **8**

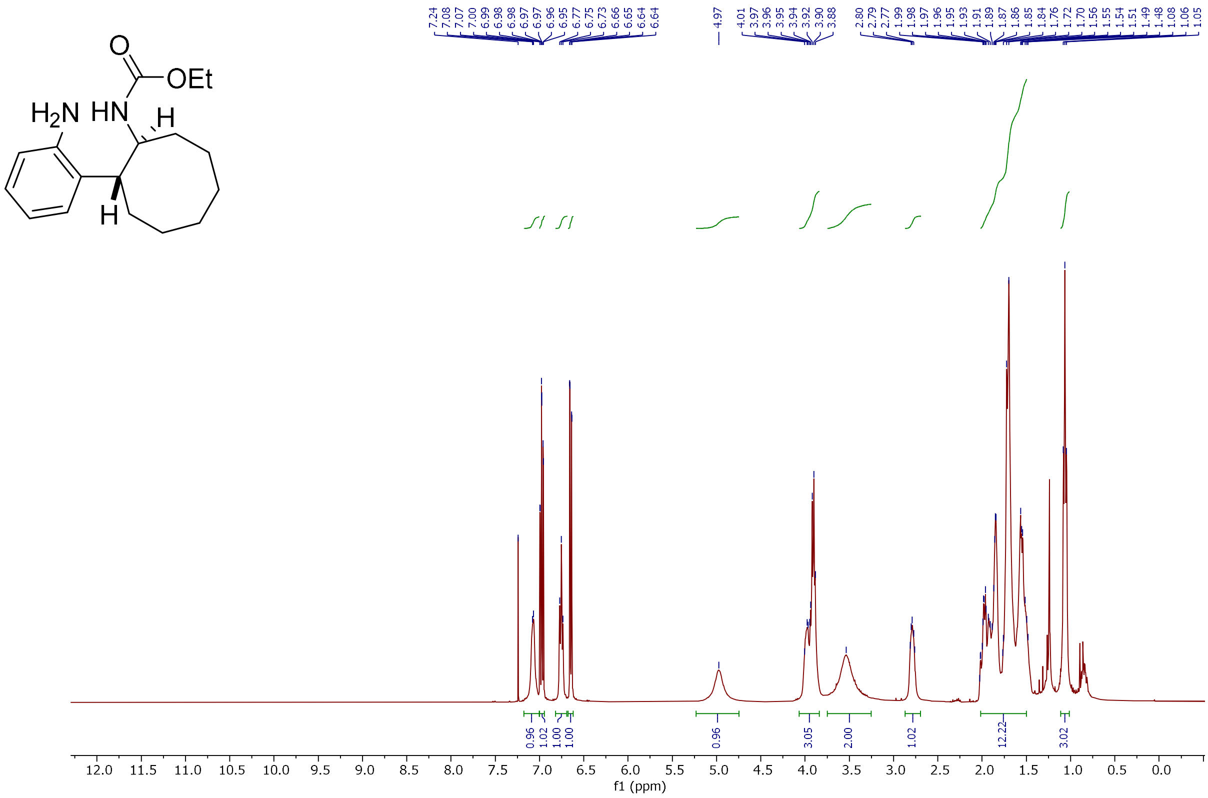

^{13}C{1H} NMR (100 MHz, $CDCl_3$) of compound **8**

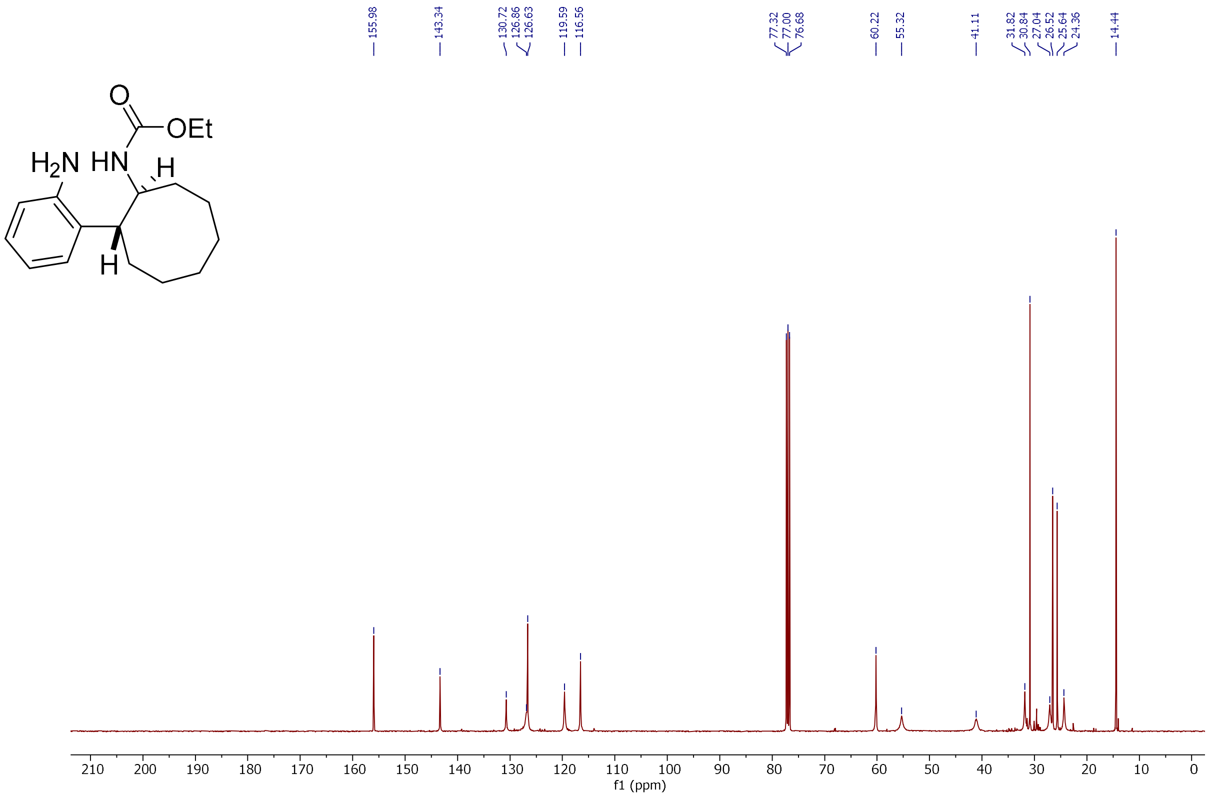

^{1}H NMR (400 MHz, $CDCl_3$) of cinnoline derivative **9**

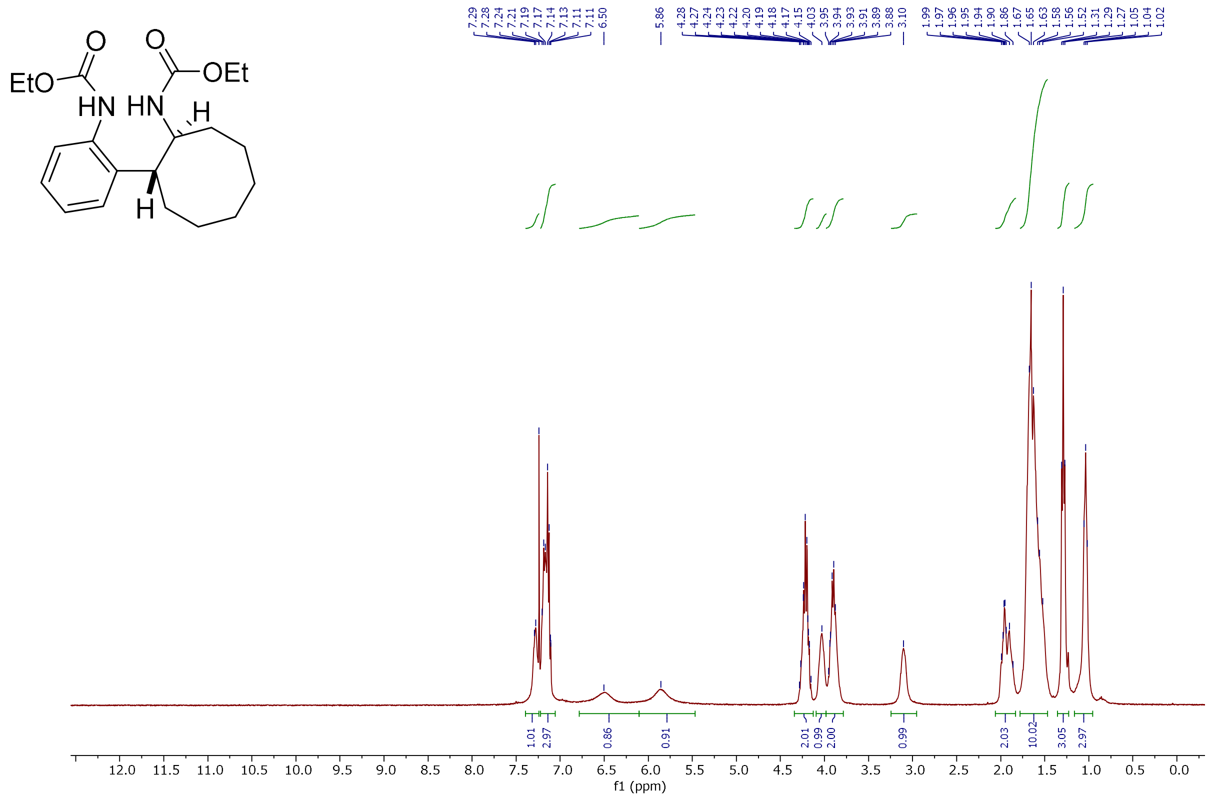

$^{13}C\{1H\}$ NMR (100 MHz, $CDCl_3$) of cinnoline derivative **9**

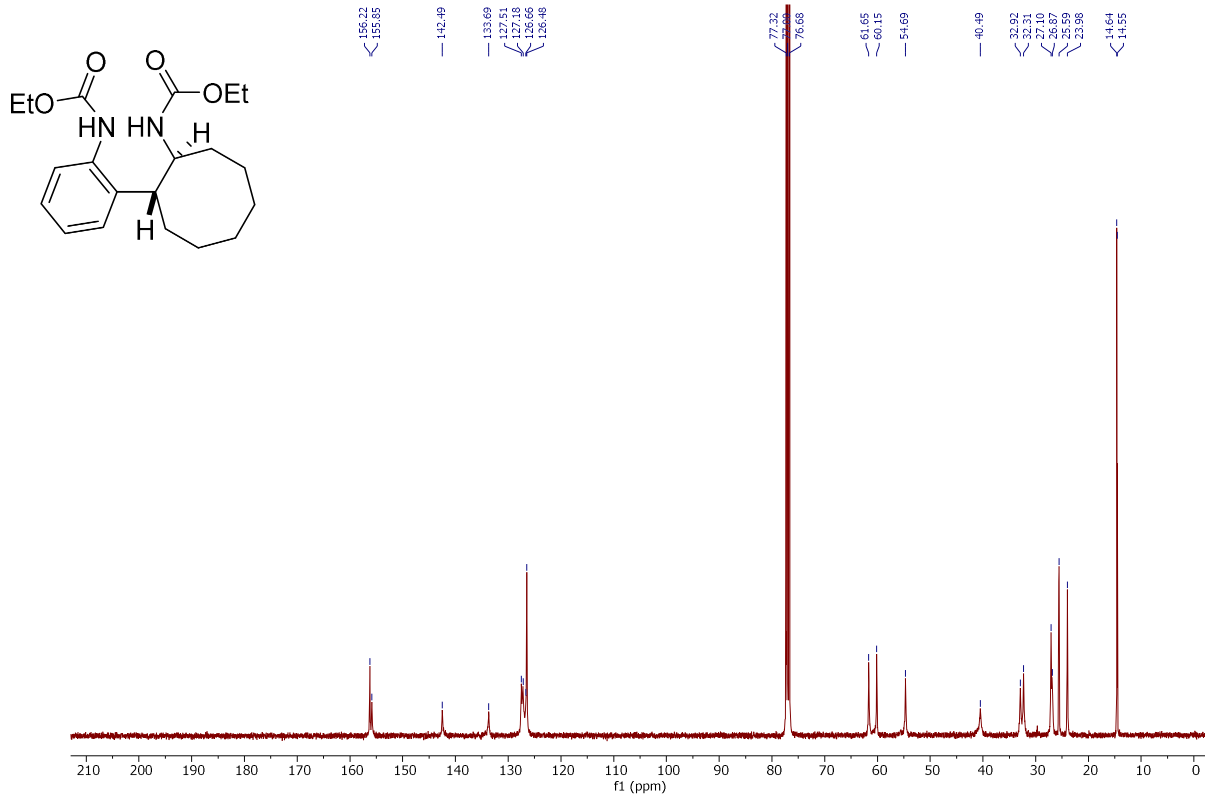

^{1}H NMR (500 MHz, CD_3OD) of fluorescein derivative **10**

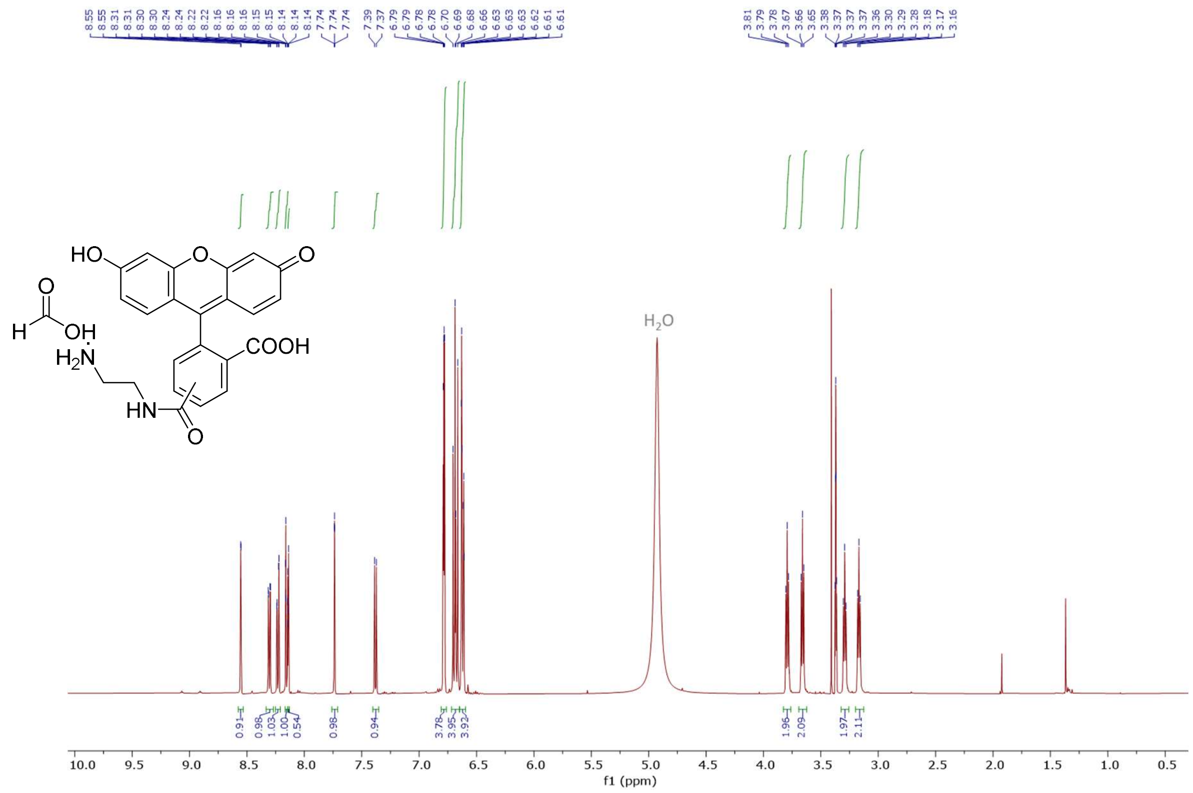

$^{13}C\{1H\}$ NMR (125 MHz, CD_3OD) of fluorescein derivative **10**

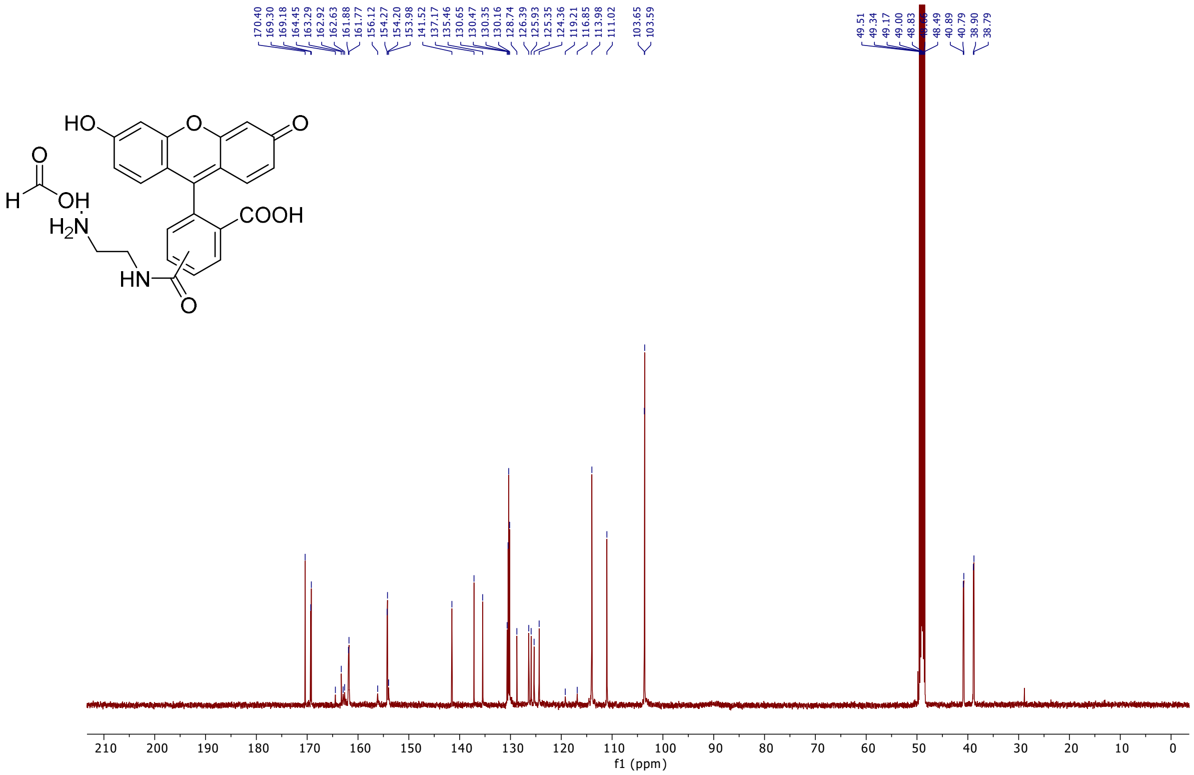

^{1}H NMR (500 MHz, $(CD_3)_2SO$) of fluorescein derivative **11**

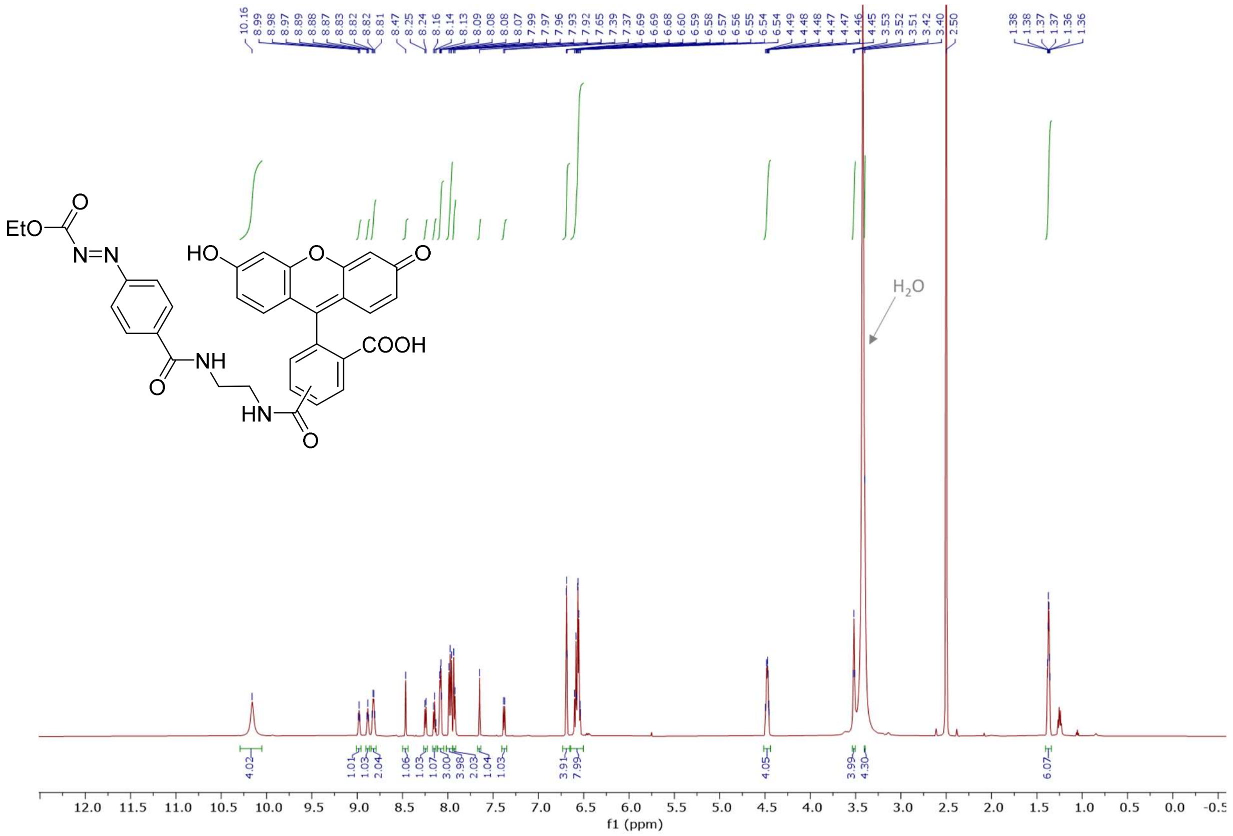

$^{13}C\{1H\}$ NMR (125 MHz, $(CD_3)_2SO$) of fluorescein derivative **11**

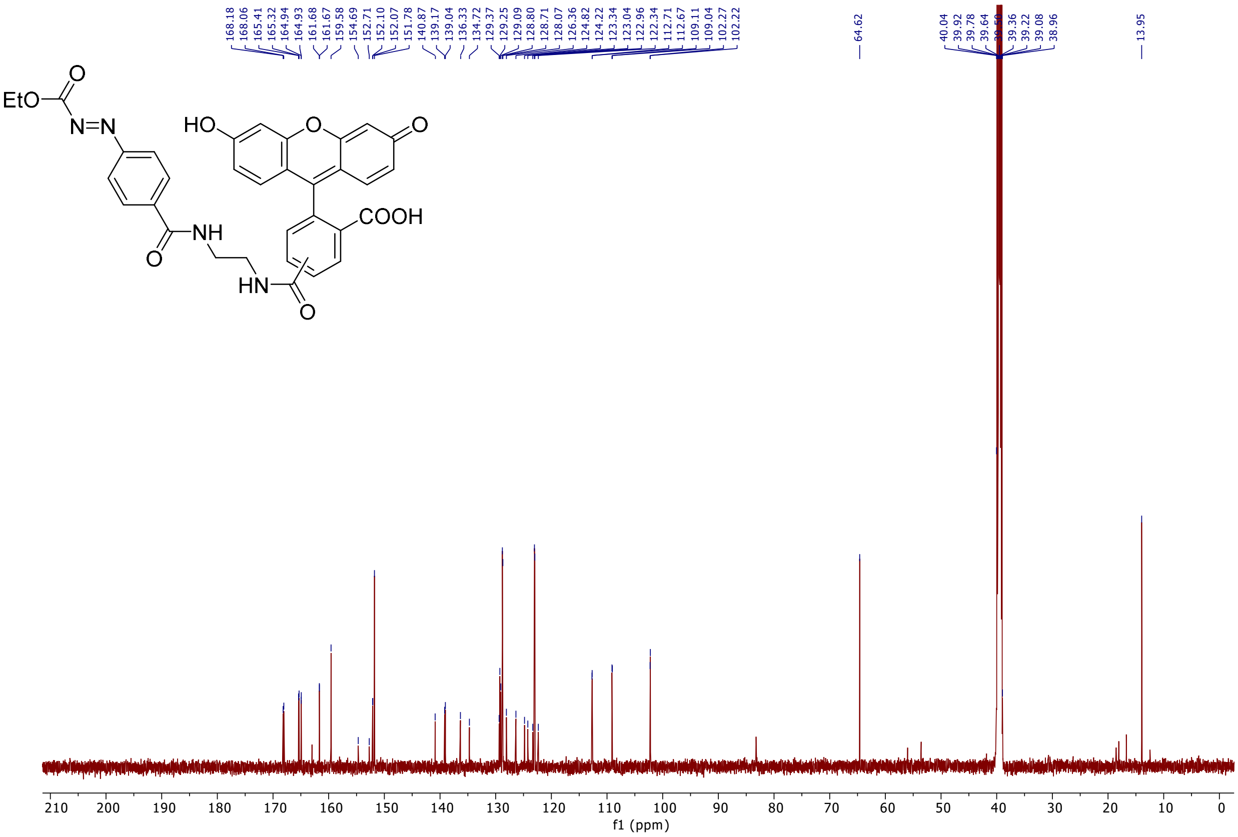

2D HSQC NMR {^{1}H-^{13}C} (125 MHz, $(CD_3)_2SO$) of fluorescein derivative **11**

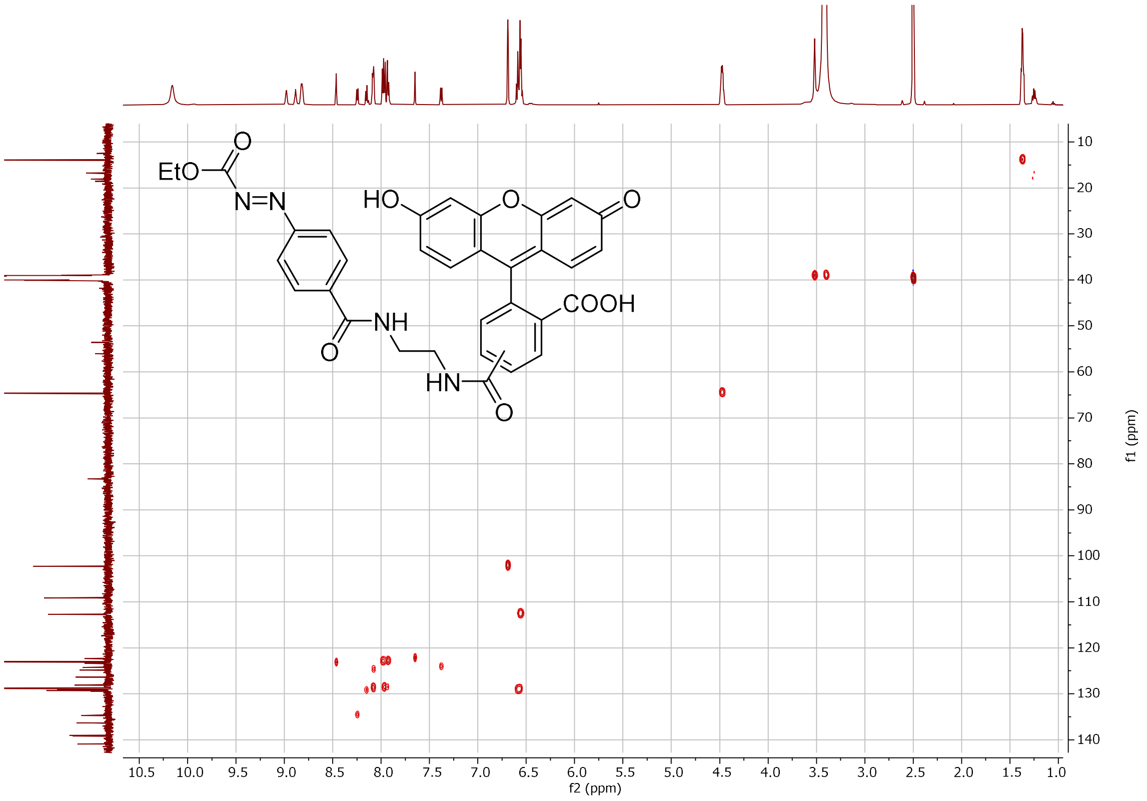

2D HMBC NMR {^{1}H-^{13}C} (125 MHz, $(CD_3)_2SO$) of fluorescein derivative **11**

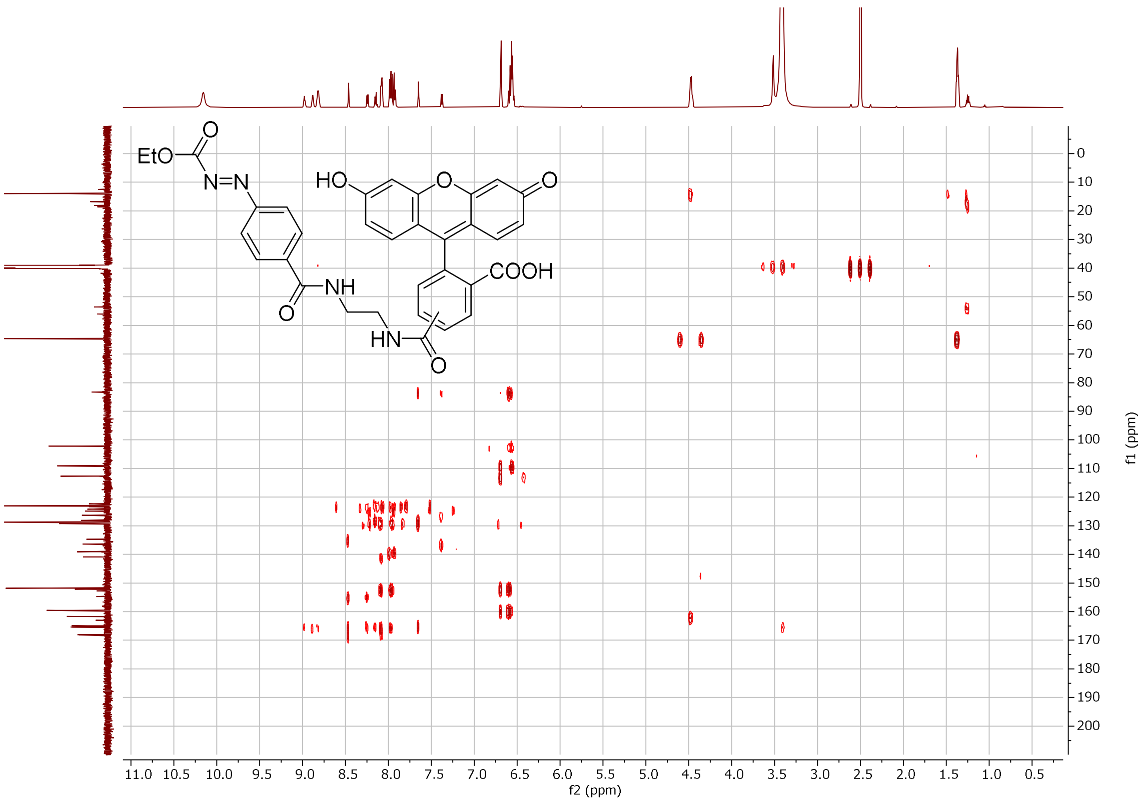

^{1}H NMR (500 MHz, $(CD_3)_2SO$) of fluorescein derivative **12**

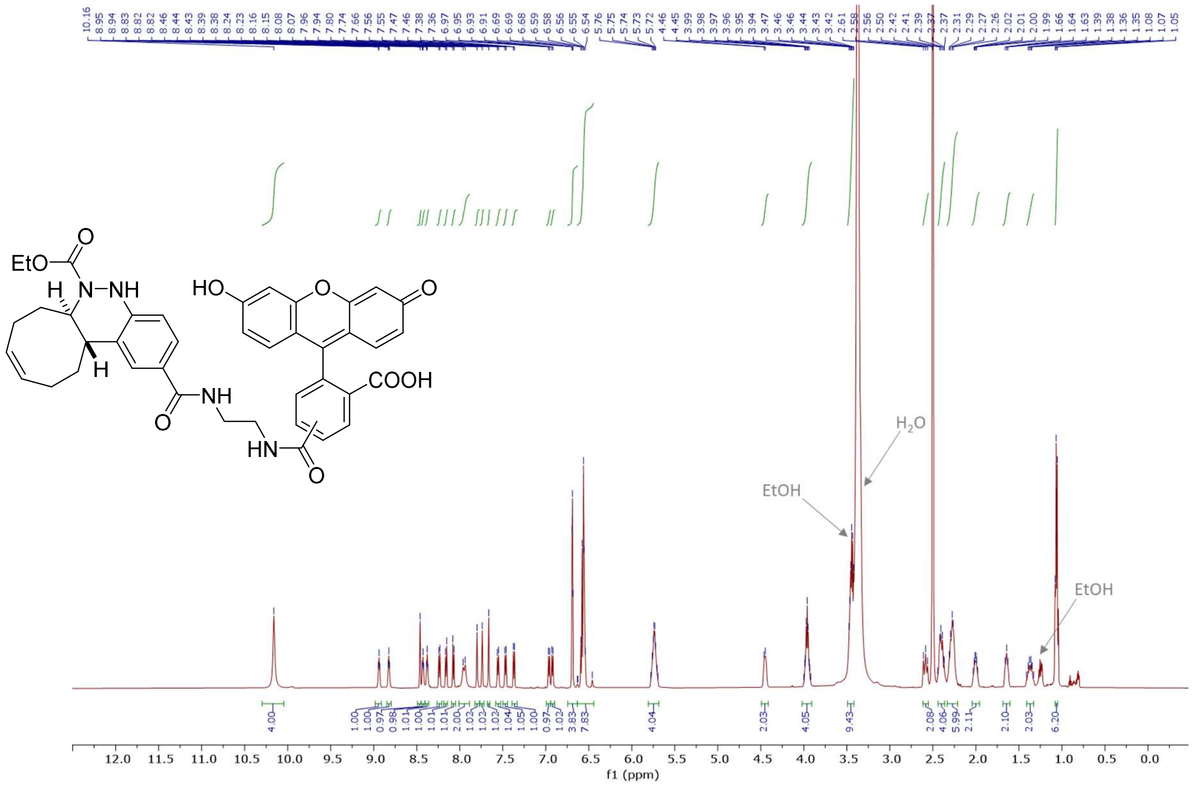

^{13}C{1H} NMR (125 MHz, $(CD_3)_2SO$) of fluorescein derivative **12**

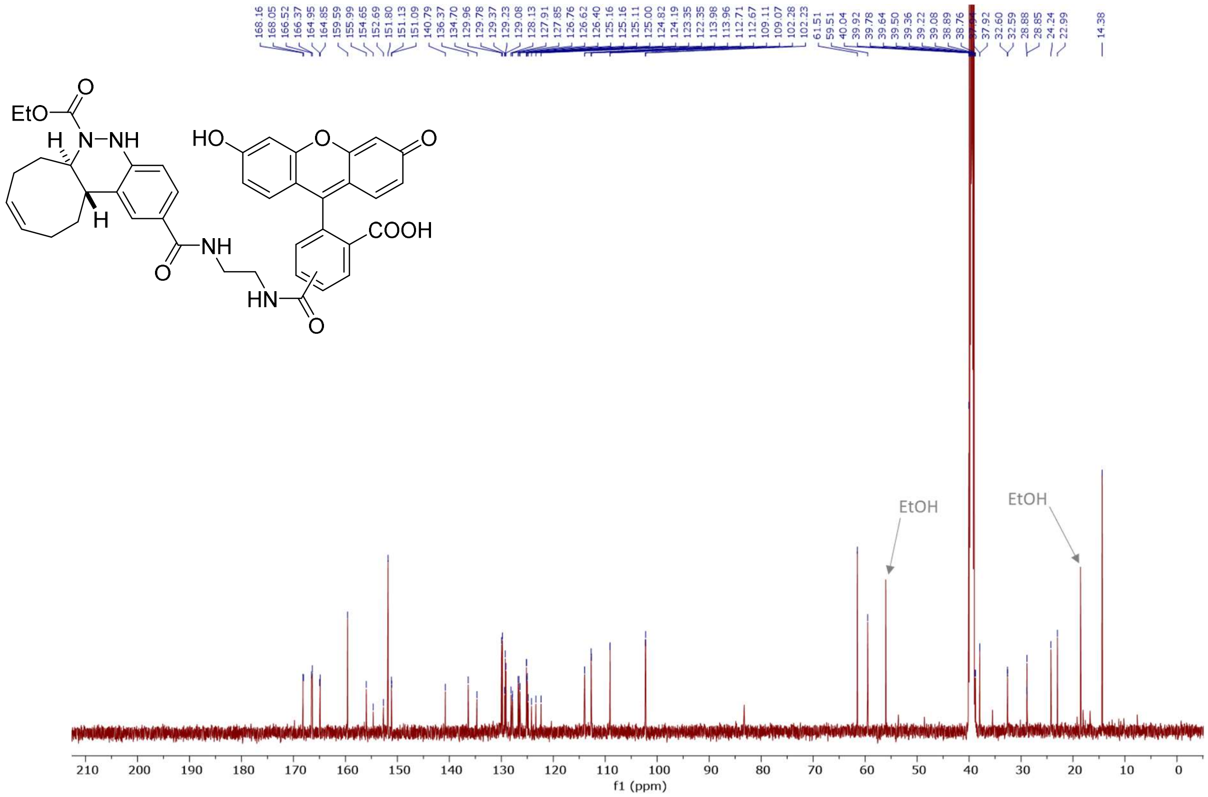

2D HSQC NMR {^{1}H-^{13}C} (125 MHz, $(CD_3)_2SO$) of fluorescein derivative **12**

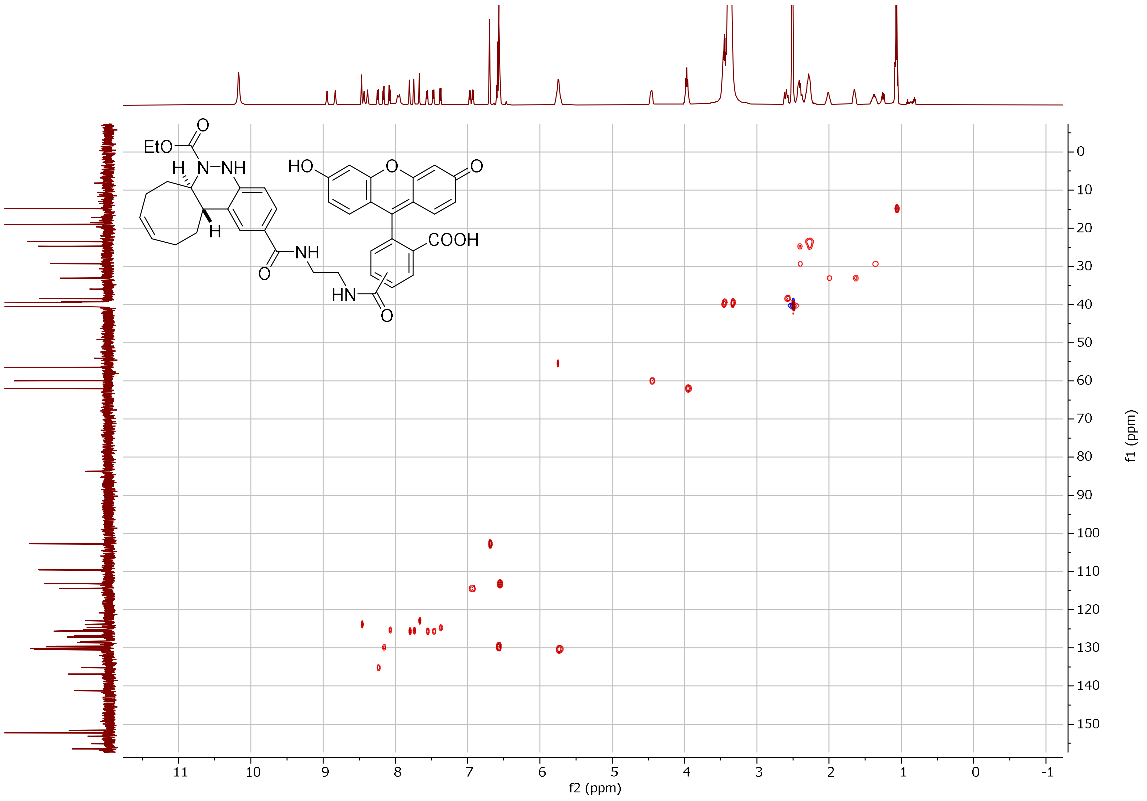

2D HMBC NMR {^{1}H-^{13}C} (125 MHz, $(CD_3)_2SO$) of fluorescein derivative **12**

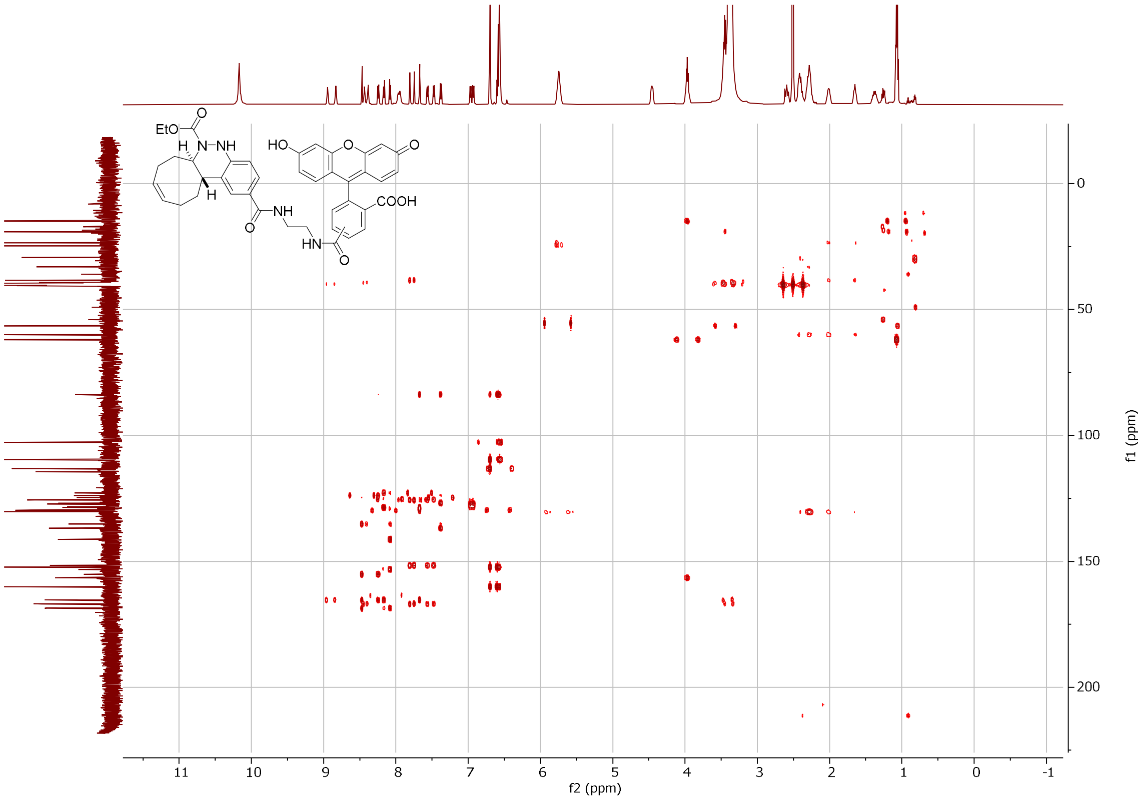

^{1}H NMR (500 MHz, $CDCl_3$) of coumarin derivative **X**

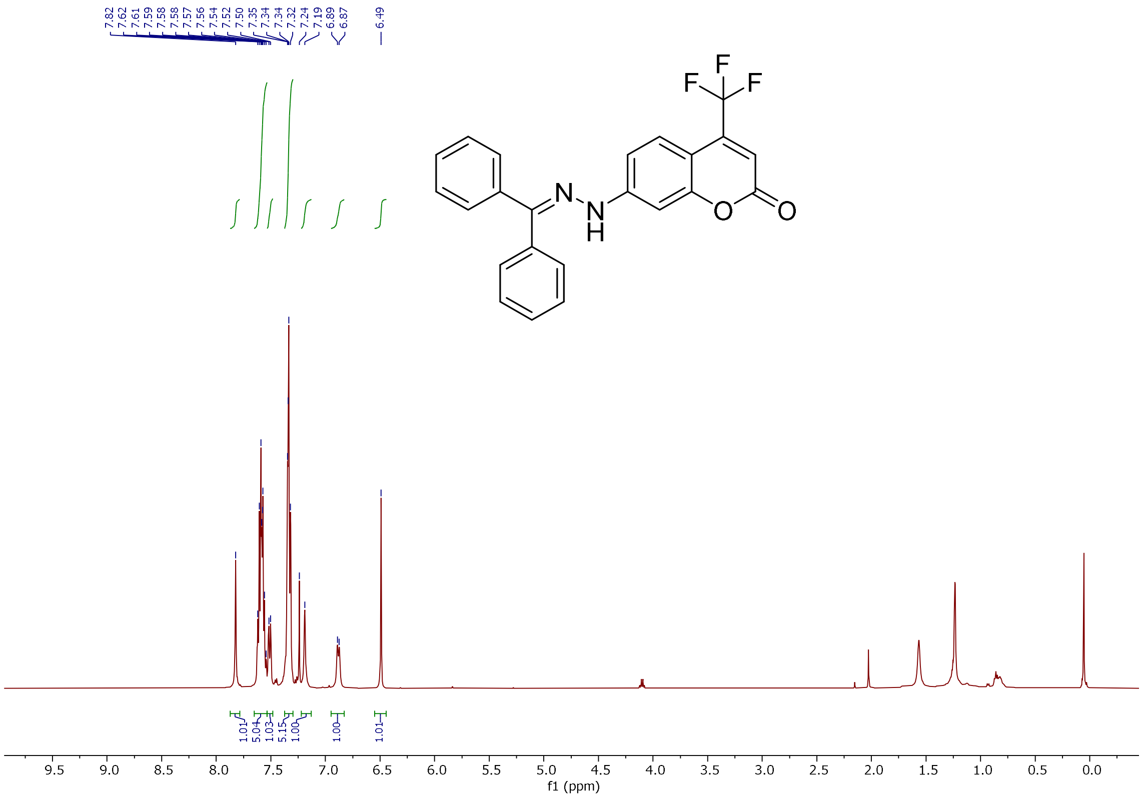

^{13}C{1H} NMR (125 MHz, $CDCl_3$) of coumarin derivative **X**

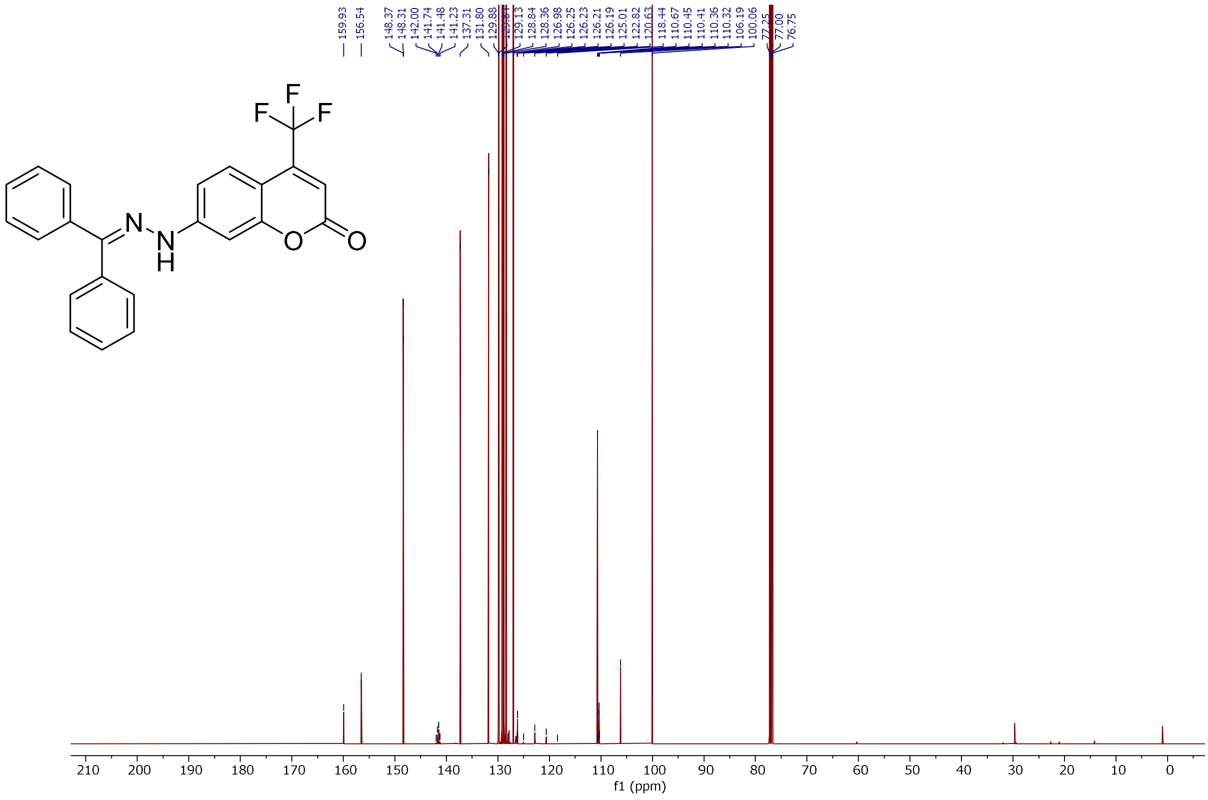

^{19}F (282 MHz, $CDCl_3$) of coumarin derivative **X**

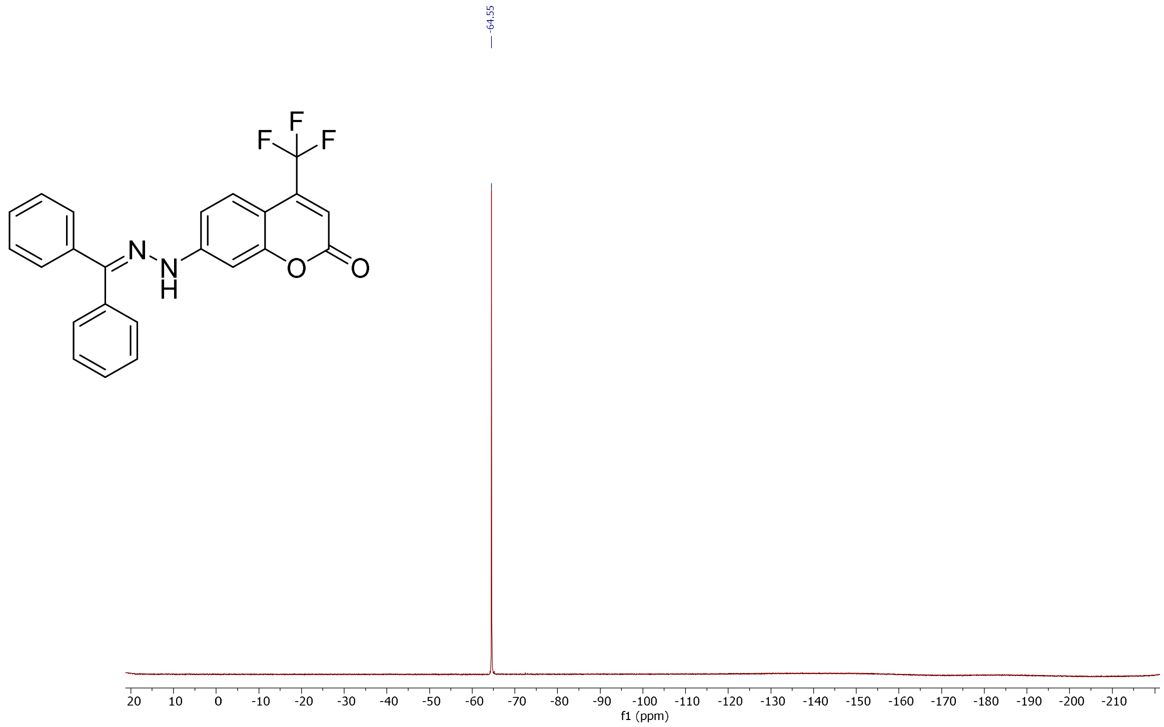

^{1}H NMR (500 MHz, CD_3OD) of coumarin derivative **XI**

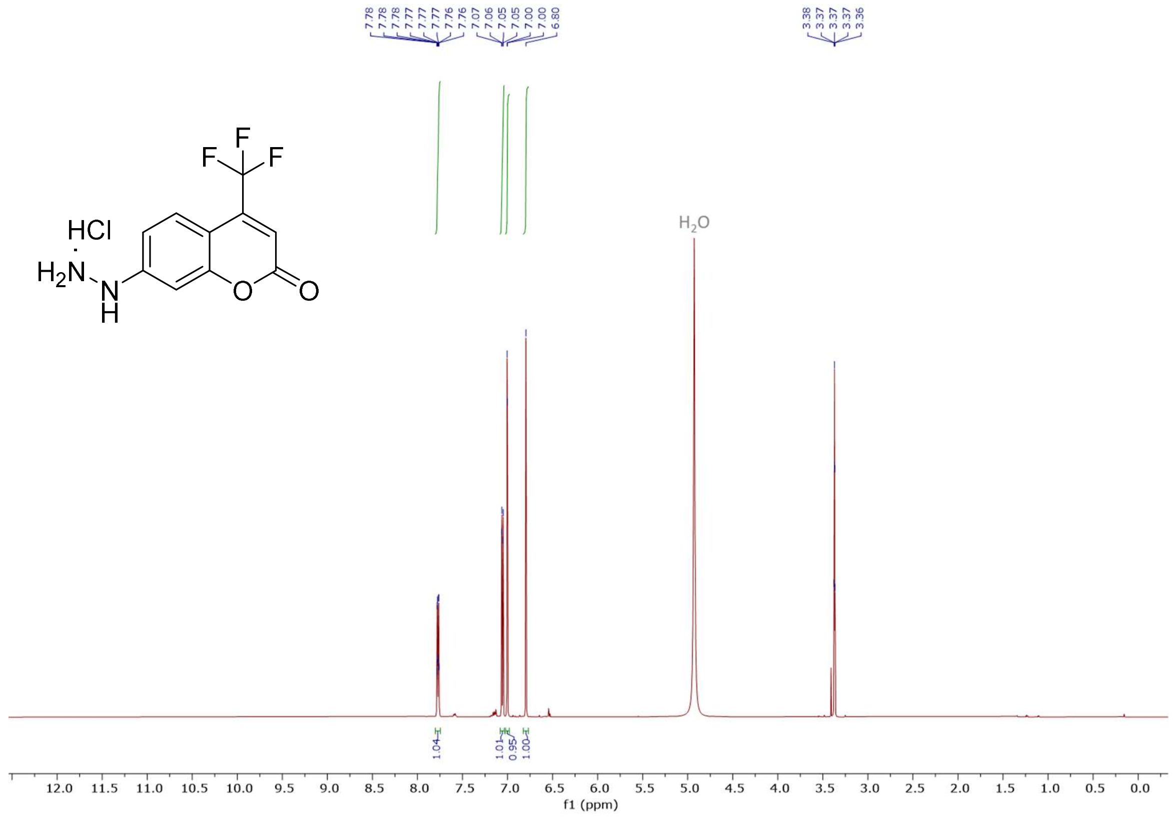

^{13}C{1H} NMR (125 MHz, CD_3OD) of coumarin derivative **XI**

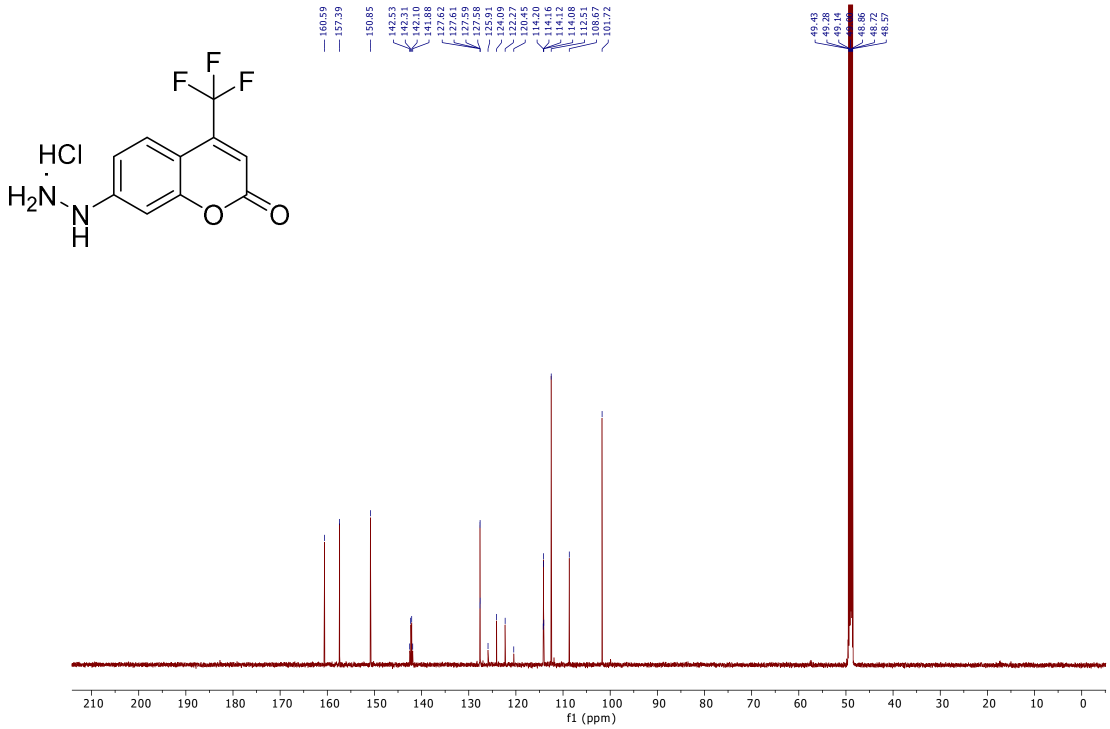

^{19}F (565 MHz, CD_3OD) of coumarin derivative **XI**

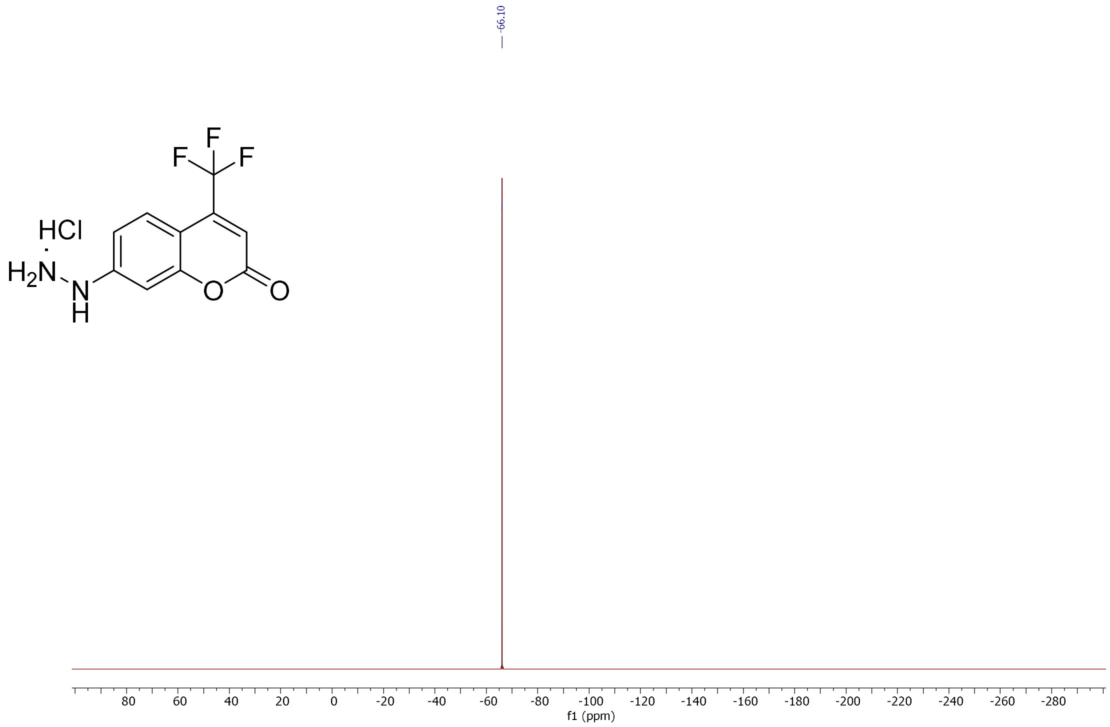

^{1}H NMR (500 MHz, CD_3OD) of coumarin derivative **XII**

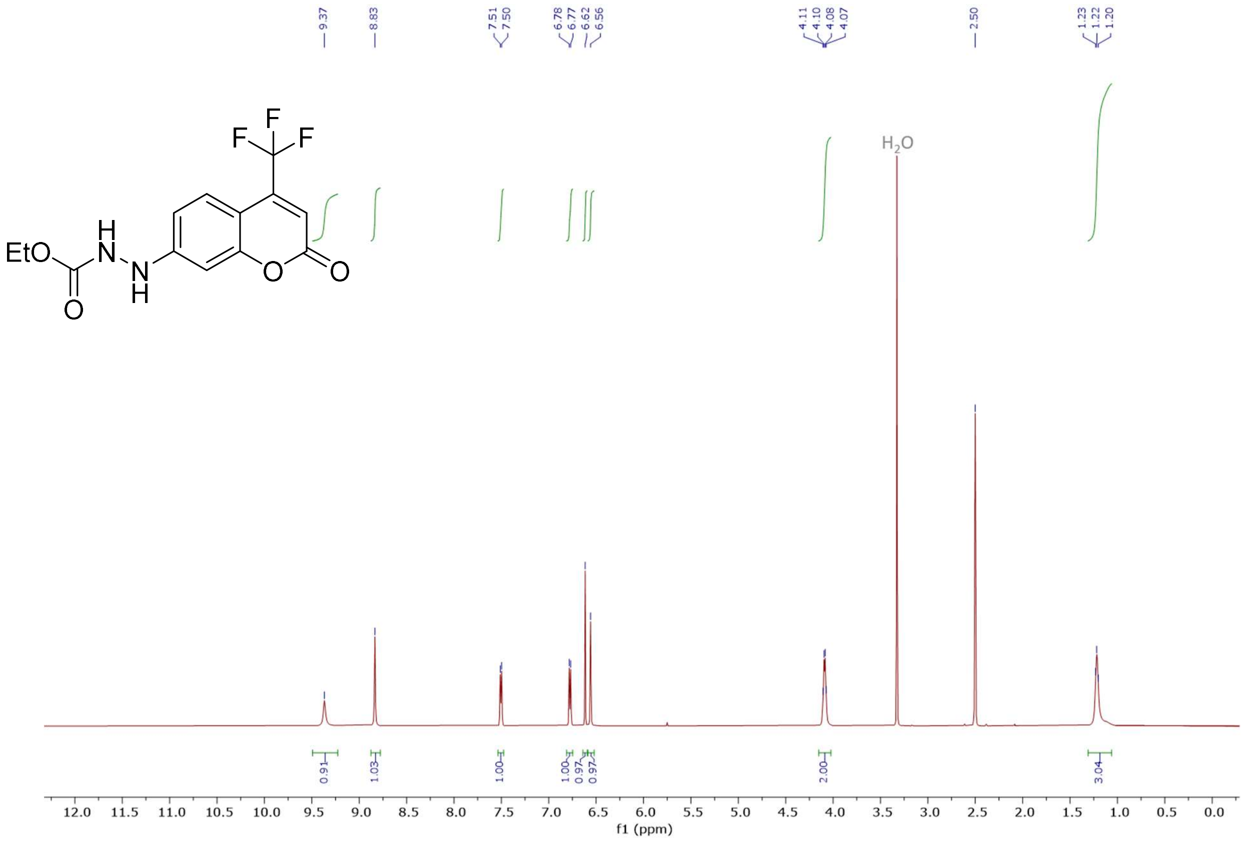

$^{13}C\{1H\}$ NMR (125 MHz, CD_3OD) of coumarin derivative **XII**

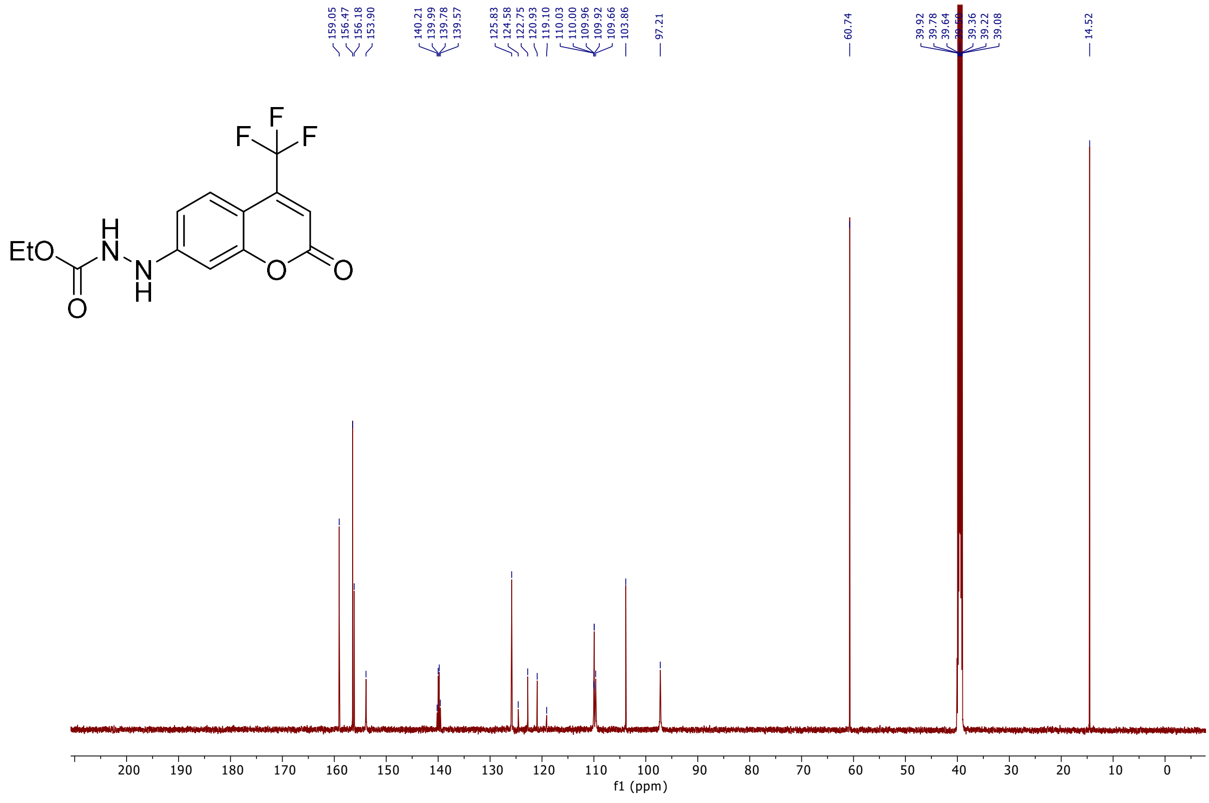

^{19}F (565 MHz, CD_3OD) of coumarin derivative **XII**

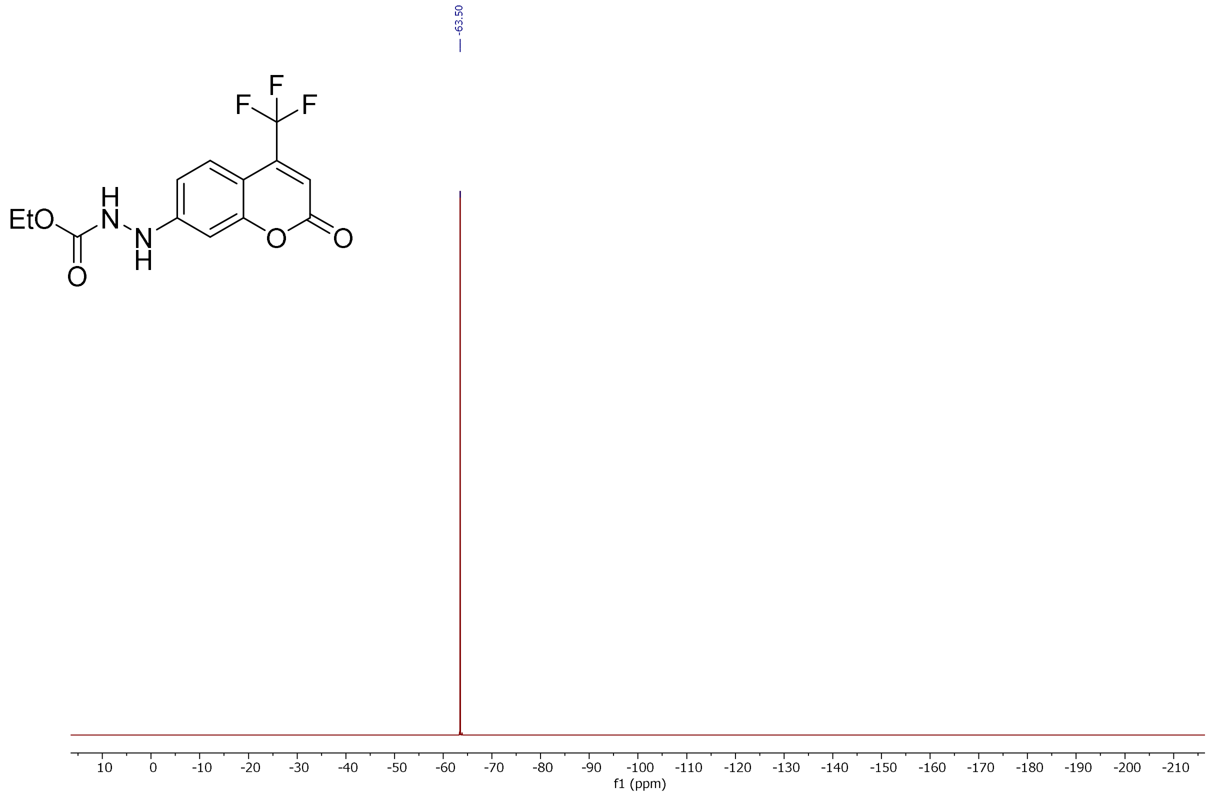

^{1}H NMR (500 MHz, $CDCl_3$) of coumarin derivative **14**

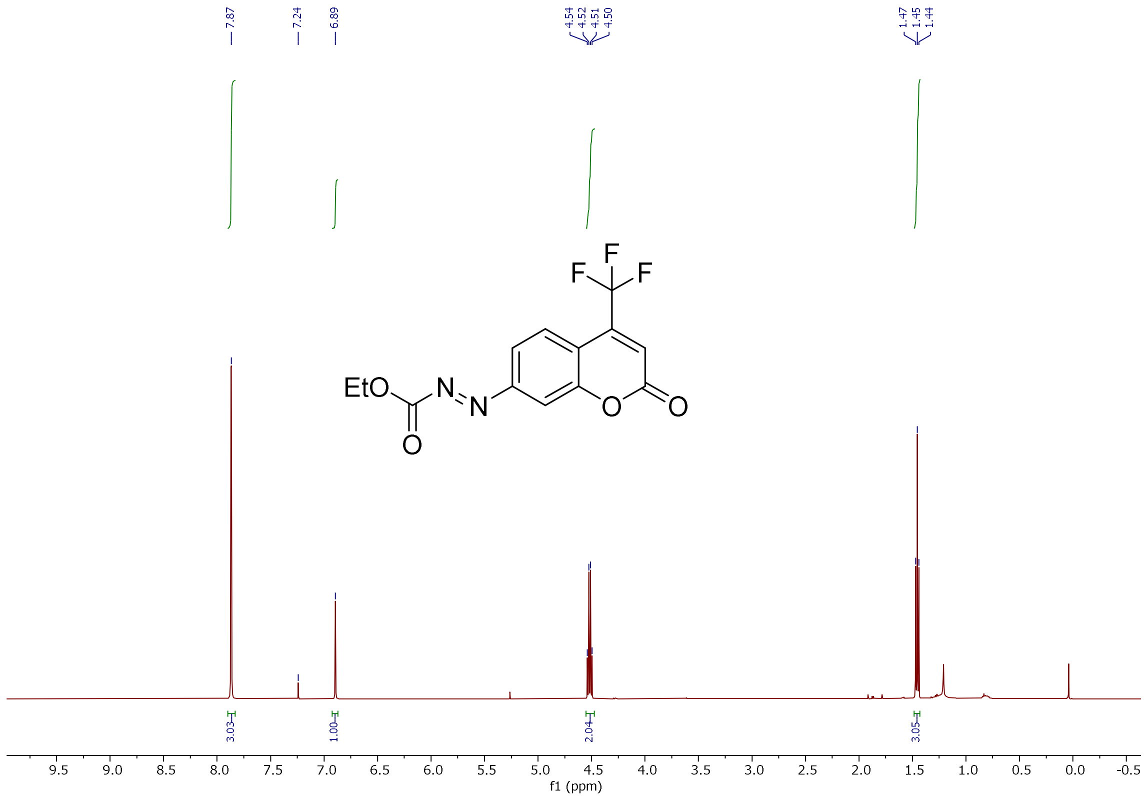

^{13}C{1H} NMR (125 MHz, $CDCl_3OD$) of coumarin derivative **14**

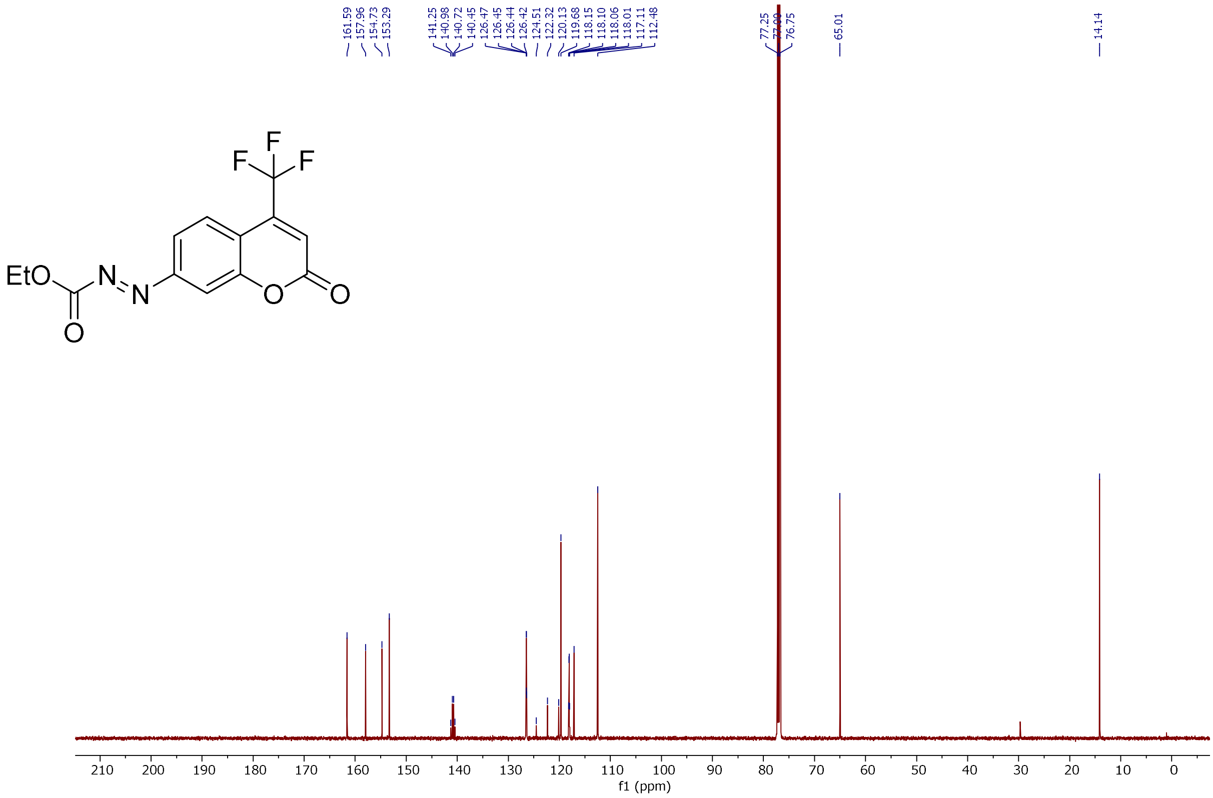

^{19}F (282 MHz, $CDCl_3$) of coumarin derivative **14**

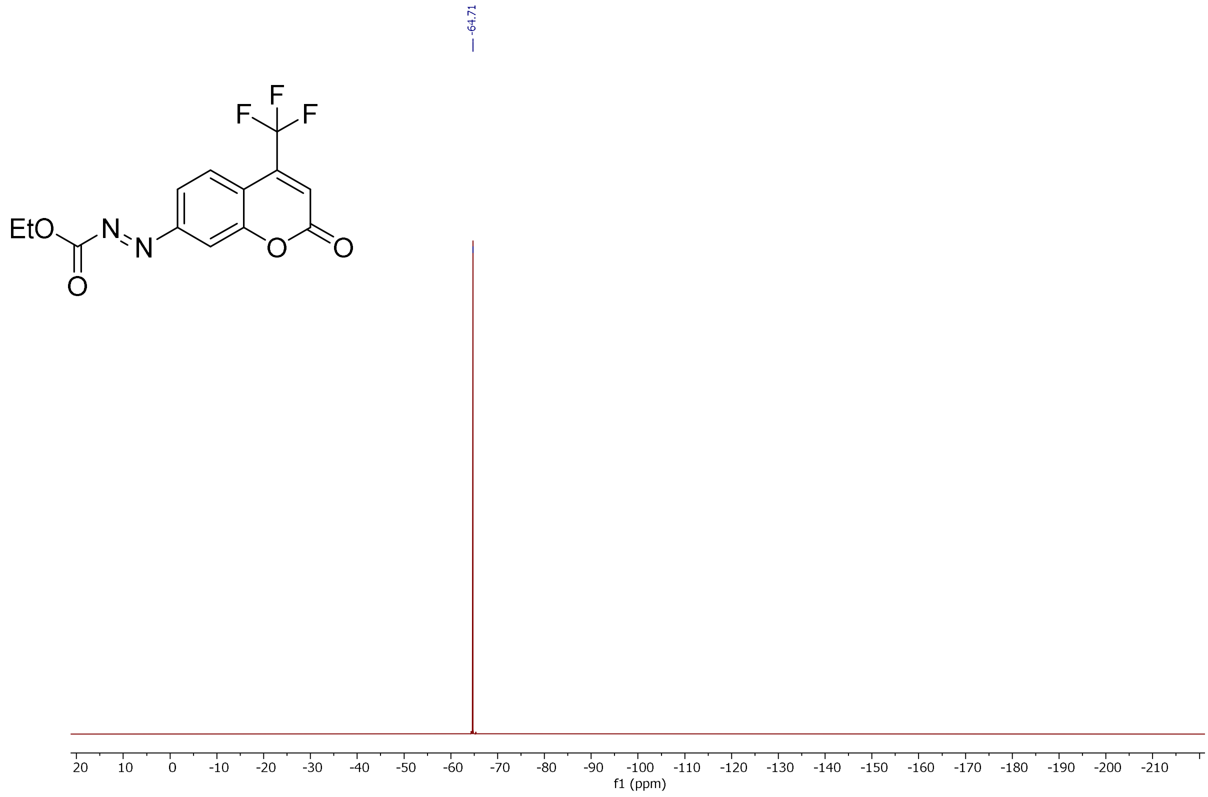

^{1}H NMR (500 MHz, $CDCl_3$) of coumarin derivative **15**

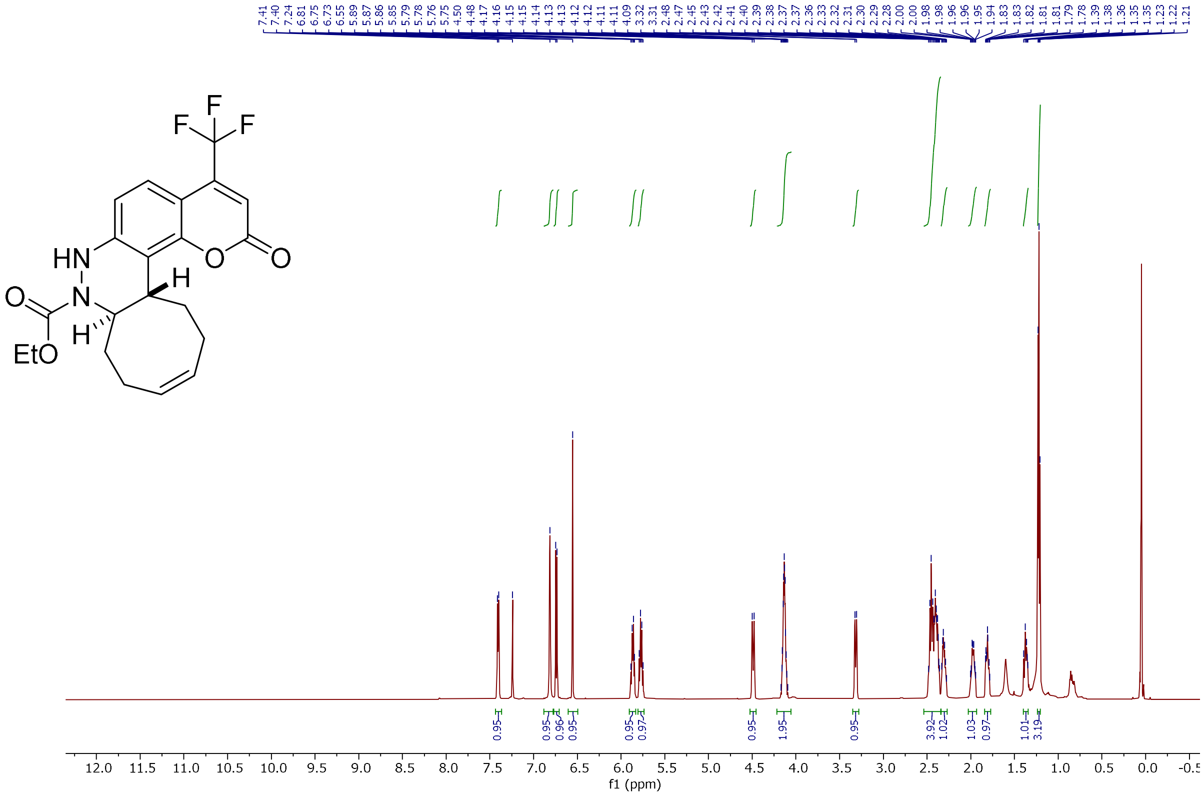

^{13}C{1H} NMR (125 MHz, $CDCl_3OD$) of coumarin derivative **15**

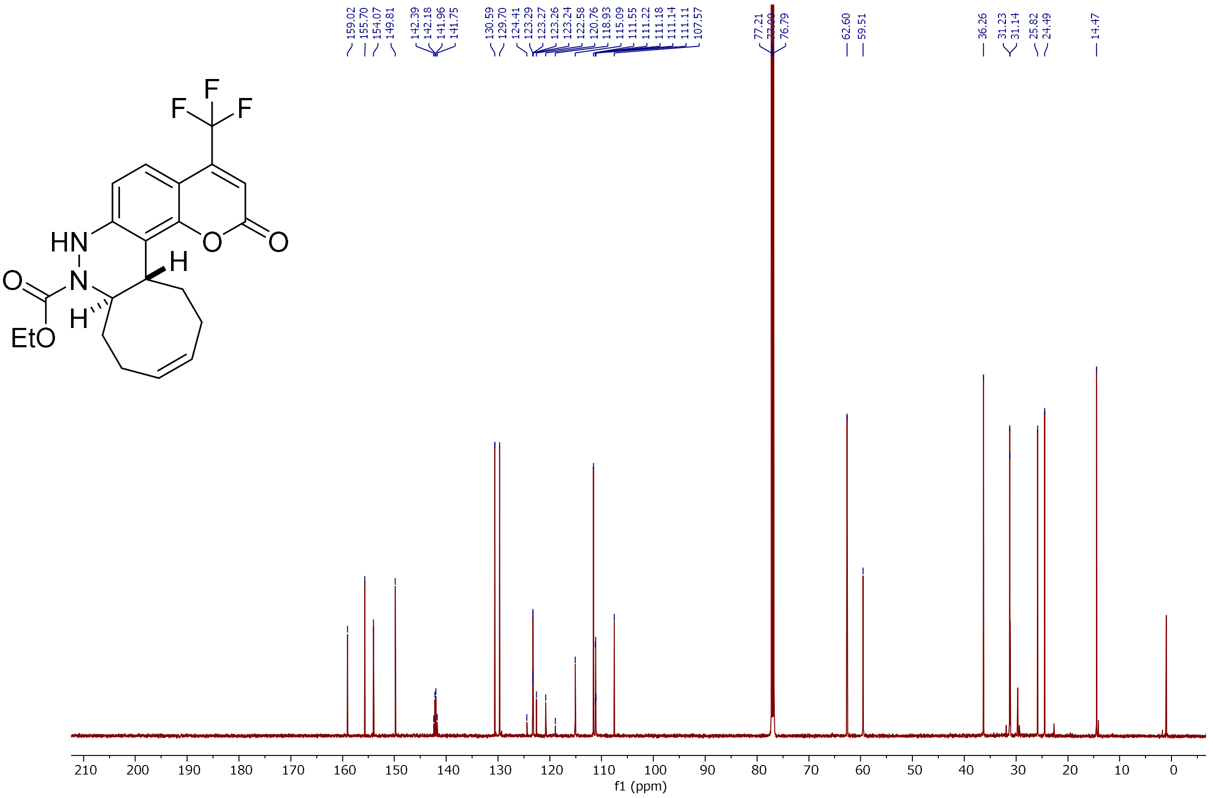

^{19}F (565 MHz, $CDCl_3$) of coumarin derivative **15**

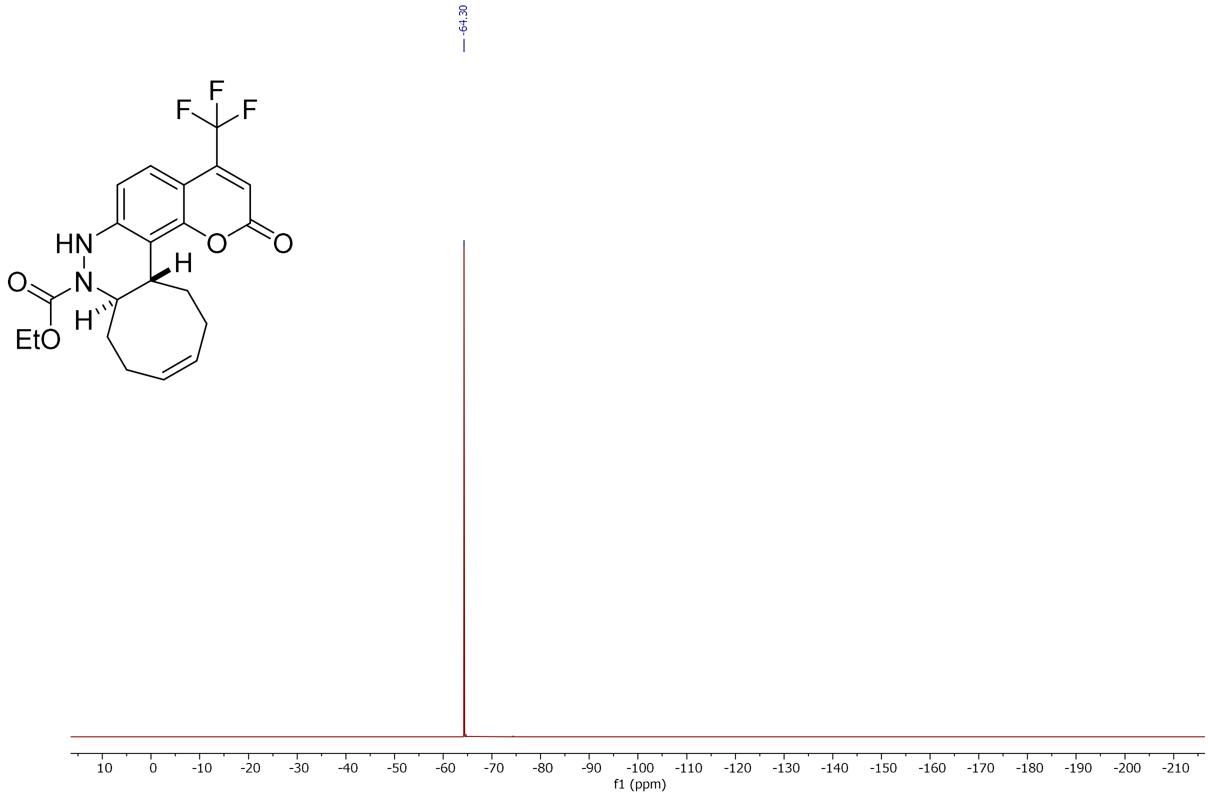

Absorption spectra of compound 15

The absorption spectra of compound **15** in different organic solvents showed only a slight positive solvatochromic effect in DMF (Figure S1), indicating that the Franck–Condon transition is only weakly affected by solvent polarity and that the ground-state electronic distribution remains essentially unchanged.[1] Consequently, the pronounced decrease in fluorescence intensity observed in polar solvents may be attributed to the stabilization of non-emissive excited states or to the enhancement of competing non-radiative decay pathways. This behavior is consistent with the population of twisted intramolecular charge-transfer (TICT) states, solvent-stabilized exciplex, or charge-transfer states.[2]

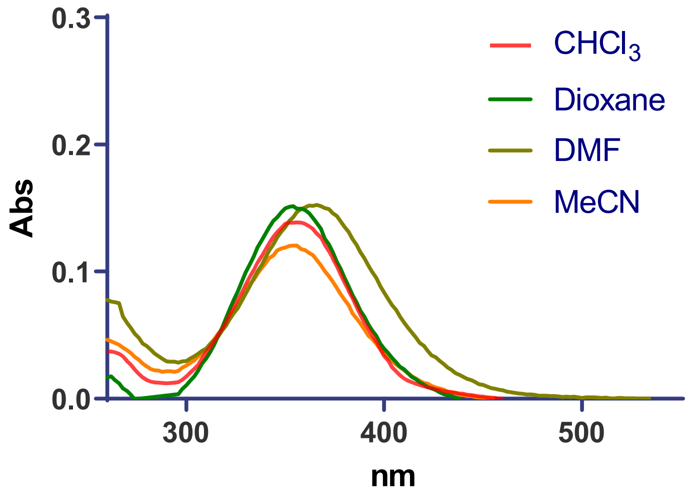

Figure S1. Absorption spectra of compound **15** in different organic solvents.

References

---

1 Gemeda, F. T. A Review on Effect of Solvents on Fluorescent Spectra. *Chem. Sci. Int. J.* **2017**, *18*, 1–12

2 (a) Phukan, S.; Saha, M.; Pal, A. M.; Bhasikuttan, A. C.; Mitra, S. Intramolecular charge transfer in coumarin based donor-acceptor systems: Formation of a new product through planar intermediate. *J. Photochem. Photobiol. A: Chem.* **2015**, *303–304*, 67–79. (b) Basavaraja, J.;

Inamdar, S. R.; Kumar, H. M. S. Solvents effect on the absorption and fluorescence spectra of 7-diethylamino-3-thenoylcoumarin: evaluation and correlation between solvatochromism and solvent polarity parameters. *Spectrochim. Acta A Mol. Biomol. Spectrosc*. **2015**, *137*, 527–534. (c) Debnath, T.; Ghosh, H. N. An Insight of Molecular Twisting of Coumarin Dyes. *ChemistrySelect*, **2020**, *5*, 9461–9476. (d) Mukhtar, A.; Mansha, A.; Asim, S.; Shahzad, A.; Bibi, S. Excited State Complexes of Coumarin Derivatives. *J. Fluoresc.* **2022**, *32*, 1–17.
